# Exploring the mechanism of Celastrol in the treatment of rheumatoid arthritis based on systems pharmacology and multi-omics

**DOI:** 10.1038/s41598-023-48248-5

**Published:** 2024-01-18

**Authors:** Liuting Zeng, Ganpeng Yu, Kailin Yang, Qi He, Wensa Hao, Wang Xiang, Zhiyong Long, Hua Chen, Xiaojun Tang, Lingyun Sun

**Affiliations:** 1 Department of Rheumatology and Immunology, Nanjing Drum Tower Hospital, Chinese Academy of Medical Sciences and Peking Union Medical College, Graduate School of Peking Union Medical College, Nanjing, China; 2 People’s Hospital of Ningxiang City, Ningxiang, China; 3 Key Laboratory of Hunan Province for Integrated Traditional Chinese and Western Medicine on Prevention and Treatment of Cardio-Cerebral Diseases, School of Integrated Chinese and Western Medicine, Hunan University of Chinese Medicine, Changsha, China; 4 Department of Rehabilitation Medicine, Guangzhou Panyu Central Hospital, Guangzhou, China; 5 Institute of Materia Medica, Chinese Academy of Medical Sciences and Peking Union Medical College, Beijing, China; 6 Department of Rheumatology, The First People′s Hospital Changde City, Changde, China; 7 Department of Rheumatology and Immunology, The First Affiliated Hospital of Anhui Medical University, Anhui, China

**Keywords:** Biochemistry, Immunology

## Abstract

To explore the molecular network mechanism of Celastrol in the treatment of rheumatoid arthritis (RA) based on a novel strategy (integrated systems pharmacology, proteomics, transcriptomics and single-cell transcriptomics). Firstly, the potential targets of Celastrol and RA genes were predicted through the database, and the Celastrol-RA targets were obtained by taking the intersection. Then, transcriptomic data and proteomic data of Celastrol treatment of RA were collected. Subsequently, Celastrol-RA targets, differentially expressed genes, and differentially expressed proteins were imported into Metascape for enrichment analysis, and related networks were constructed. Finally, the core targets of Celastrol-RA targets, differentially expressed genes, and differentially expressed proteins were mapped to synoviocytes of RA mice to find potential cell populations for Celastrol therapy. A total of 195 Celastrol-RA targets, 2068 differential genes, 294 differential proteins were obtained. The results of enrichment analysis showed that these targets, genes and proteins were mainly related to extracellular matrix organization, TGF-β signaling pathway, etc. The results of single cell sequencing showed that the main clusters of these targets, genes, and proteins could be mapped to RA synovial cells. For example, Mmp9 was mainly distributed in Hematopoietic cells, especially in Ptprn+fibroblast. The results of molecular docking also suggested that Celastrol could stably combine with molecules predicted by network pharmacology. In conclusion, this study used systems pharmacology, transcriptomics, proteomics, single-cell transcriptomics to reveal that Celastrol may regulate the PI3K/AKT signaling pathway by regulating key targets such as TNF and IL6, and then play an immune regulatory role.

## Introduction

Rheumatoid arthritis (RA) is a common rheumatoid immune disease characterized by immune destruction of the synovium ^1^, which can lead to continuous and progressive destruction of articular cartilage, bone and para-articular tissue, and eventually joint deformity and loss of function ^2^. Patients with RA are all over the world, with a total incidence of 0.5–1%, of which the incidence in inland China is about 0.42%, and female patients are about 4 times that of male patients ^3,4^. American medical statistics analysis shows that the annual direct medical expenses of patients suffering from RA disease are on average 2000 US dollars more than that of patients without RA ^5^. Therefore, RA not only greatly reduces the quality of life and work of patients, but also significantly increases the economic burden of the affected family, resulting in a huge social burden ^6^. RA is an autoimmune disease with unknown etiology, which may be related to genetics, external infections, hormone disorders in the body, and immune imbalances, but its exact pathogenesis is still unclear ^7–9^. The early pathological damage is mainly manifested as immune hypertrophy and hypertrophy of the synovial tissue of the joint capsule and immune-related inflammation ^10^. Among them, a variety of inflammatory cells (CD4+T cells, CD8+T cells, etc.) and inflammatory factors (IL-1α, L-1β, TNF-α and IL-6, etc.) can promote the formation and development of RA. The current clinical treatments mainly include glucocorticoids (such as dexamethasone), disease-modifying antirheumatic drugs (DMARDs) (such as methotrexate) and non-steroidal anti-inflammatory drugs (NSAIDs) (such as diclofenac) ^11–14^. However, some DMARDs have serious side effects and can cause severe gastrointestinal reactions, so there is an urgent need to find drugs with less side effects ^15^.

**Figure 1:**
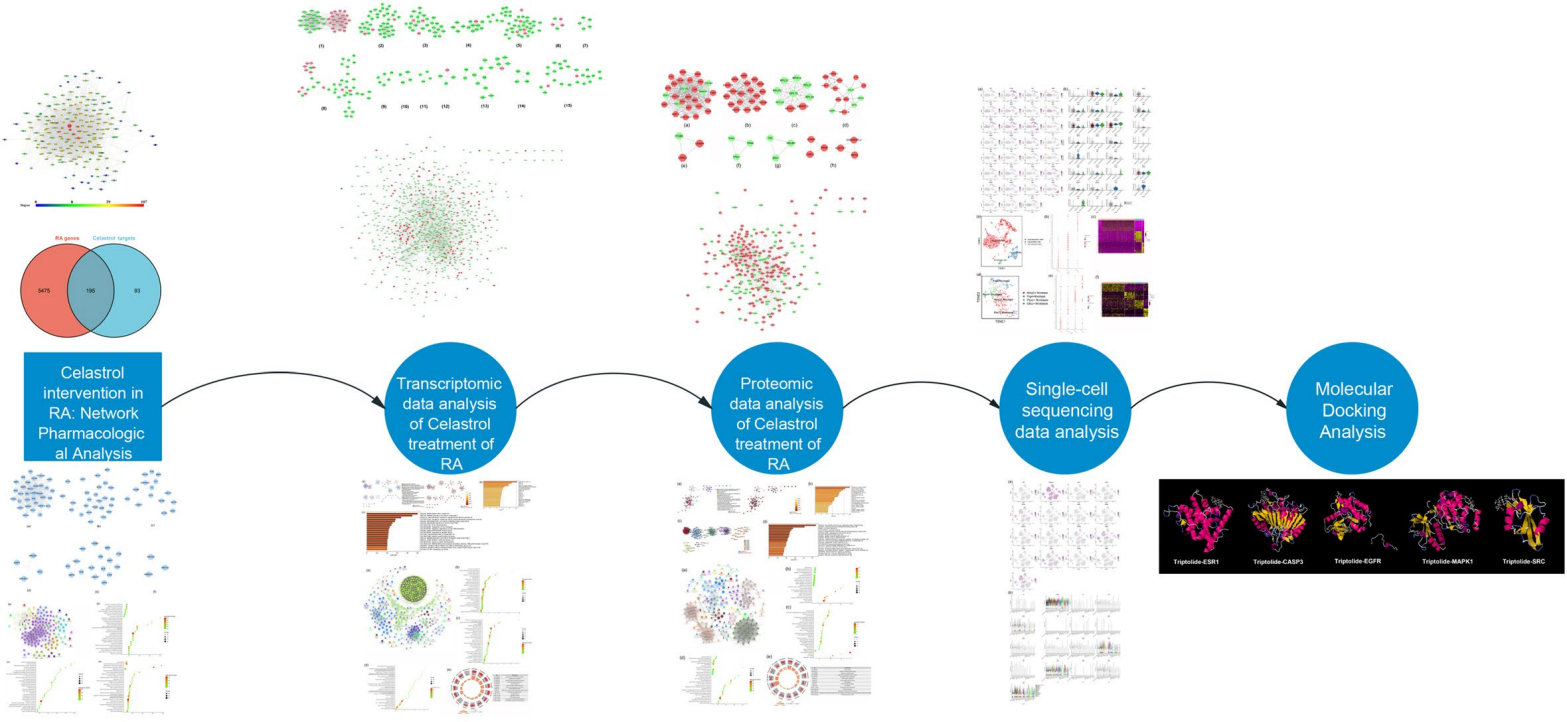
The research processes.

Celastrol is a natural product extracted from the root bark of *Tripterygium wilfordii* Hook. f. ^16^. Celastrol has various pharmacological activities such as anti-inflammatory, antiviral, immune regulation, and anti-tumor ^17–20^. Current research shows that it has the mechanism of inhibiting inflammation and regulating various immune cells (T cells, B cells, macrophages, neutrophils) in the treatment of RA. However, its mechanism on synovial tissue remains to be further revealed. This study explored the molecular network of Celastrol in the treatment of RA through the informatics and transcriptomic strategies of the Department of Chemistry, and further revealed the mechanism and target of Celastrol in the treatment of RA. Through data modeling and network analysis, based on the “disease-gene-target-drug” interaction relationship, network pharmacology constructs related networks from a systematic perspective to explore the mechanism of action and pathogenesis of traditional Chinese medicine (TCM), and provides theoretical data support for the research of TCM ^21^. Its main research methods are high-throughput omics sequencing data, molecular simulation network data and professional computer simulation analysis. It is represented by a variety of omics technologies, which provide important methodological support for explaining the scientific connotation of the pharmacological mechanism of natural plant herbal components intervening in diseases ^22–24^. Our previous research used network pharmacology and other research methods to analyze the mechanism of TCM in the treatment of cardiovascular and cerebrovascular diseases and immune inflammatory diseases. Single-cell RNA sequencing technology is a high-throughput sequencing technology developed in recent years. It can study cellular gene expression and identify cellular heterogeneity at the single-cell level, greatly broadening researchers' understanding of biomolecular mechanisms such as cardiovascular and cerebrovascular, autoimmune diseases, tumor generation, and immune responses ^25^. Our previous studies have systematically revealed the heterogeneity and pathogenesis of autoimmune diseases such as RA and Behcet’s disease through single-cell RNA sequencing, and identified potential new therapeutic targets, providing guidance for the diagnosis and treatment of skin diseases ^26,27^. In this study, single-cell transcriptomics, proteomics and transcriptomics were integrated to develop a new research strategy and explore the molecular network mechanism of Celastrol in the treatment of RA. The research processes were shown in Fig. 1.

## Results

### Celastrol-RA target PPI network analysis

#### Celastrol targets and RA genes

**Figure 2:**
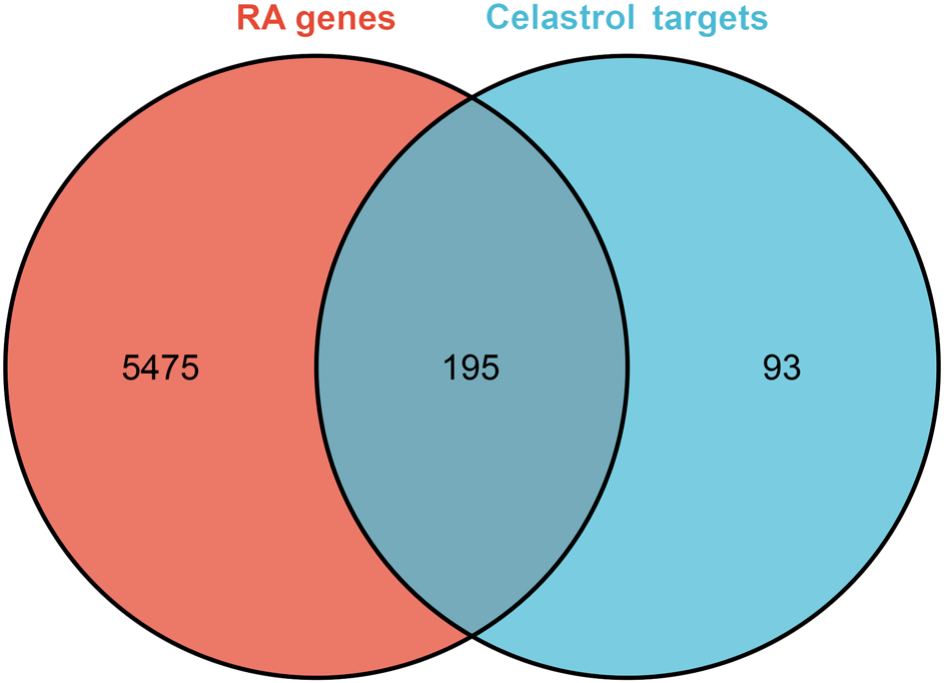
The intersection of Celastrol targets and RA genes.

**Figure 3:**
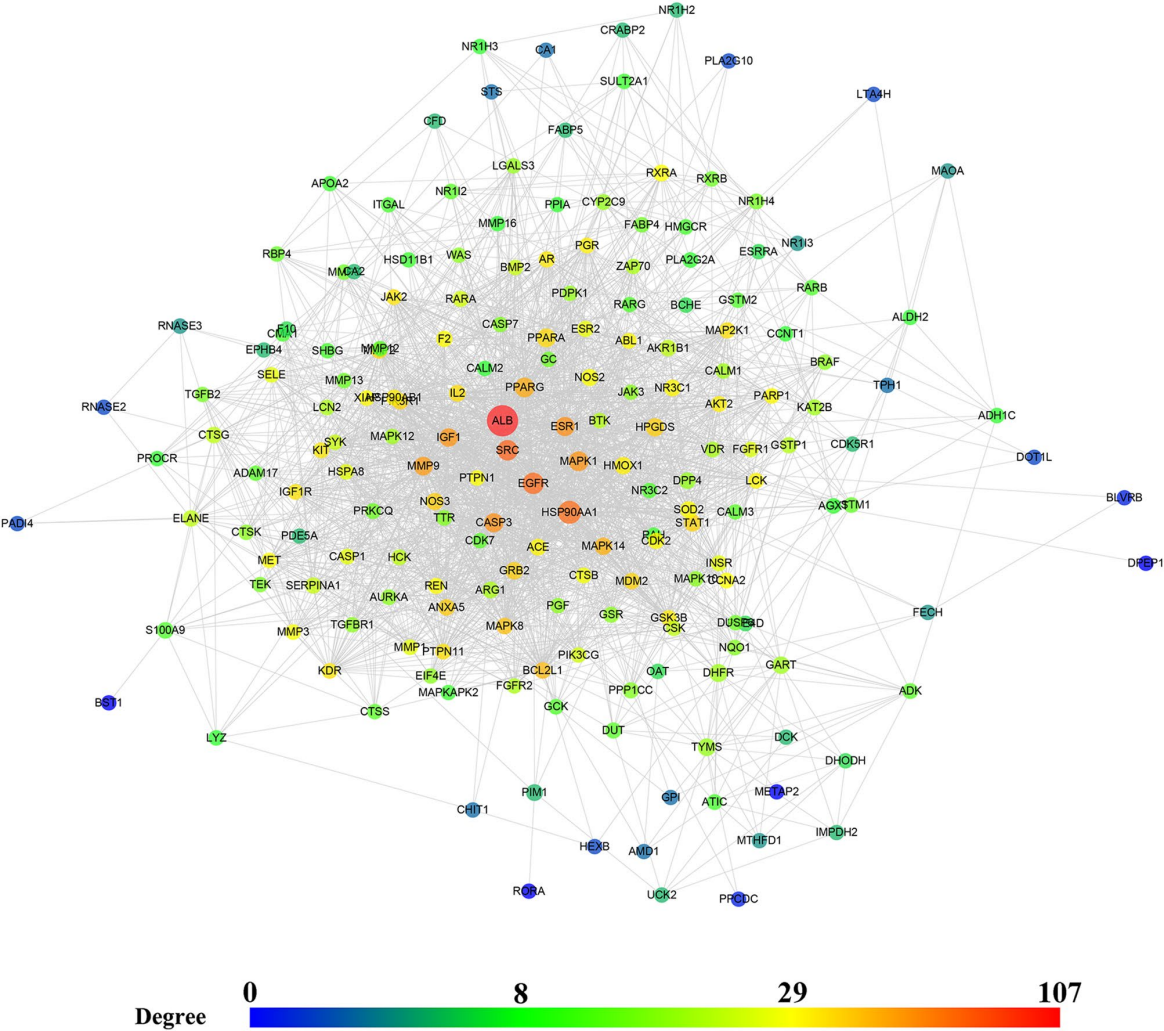
Celastrol-RA target PPI network (The size of nodes is positively correlated with edge betweenness).

**Figure 4:**
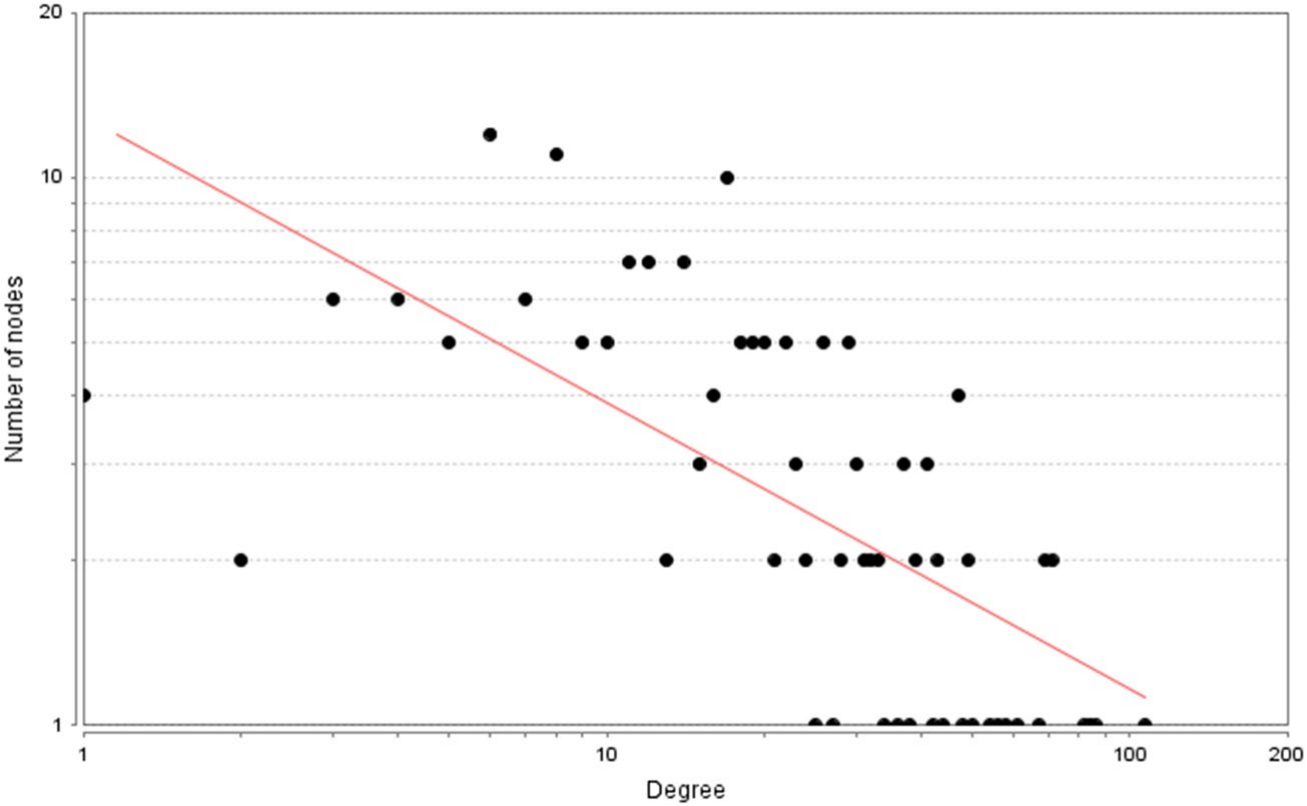
Node degree distribution of Celastrol-RA target PPI network.

**Figure 5:**
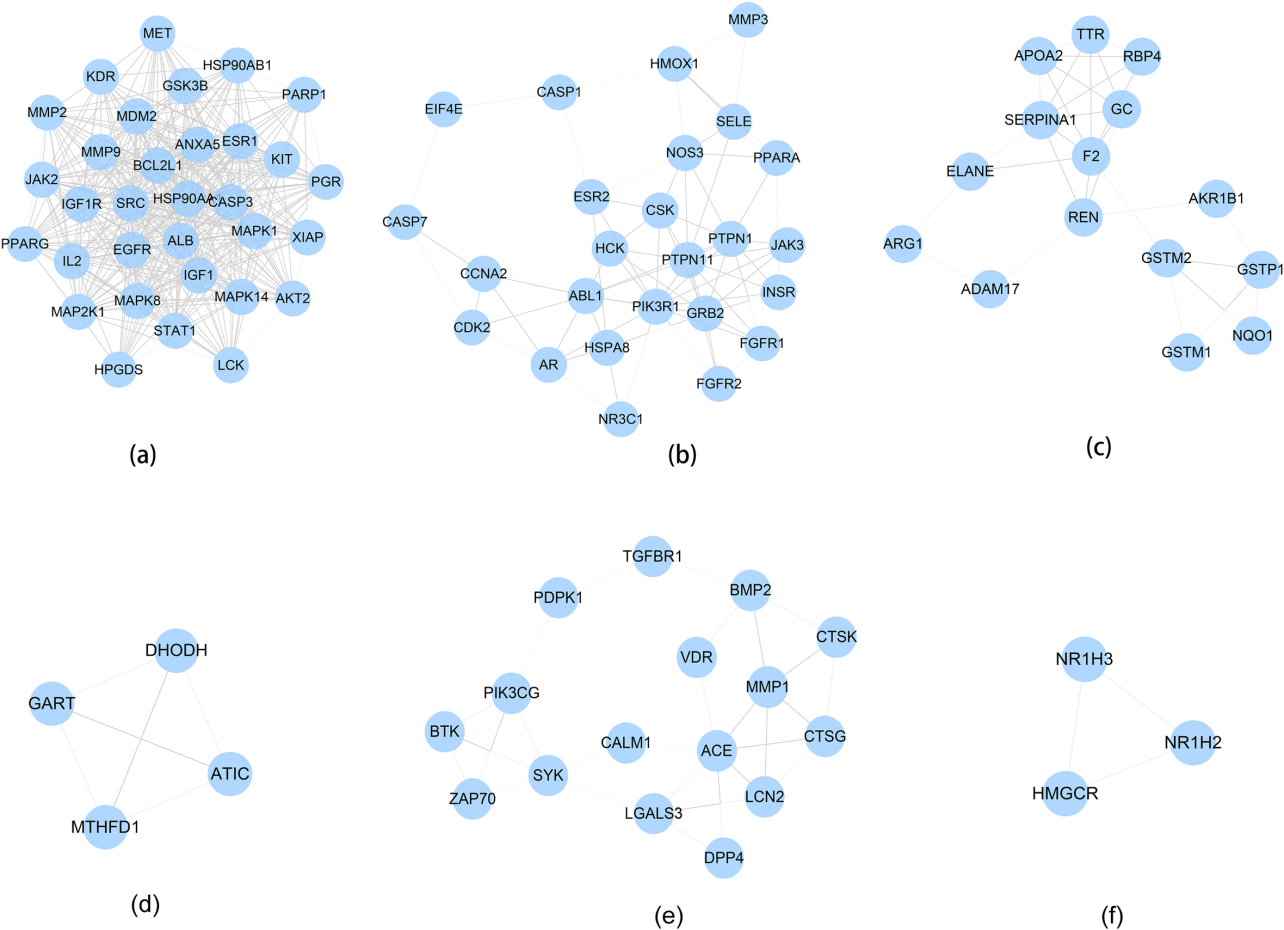
Clusters of Celastrol-RA target PPI network.

A total of 288 Celastrol targets were predicted from Pharmmapper and 5670 RA genes were obtained from the databases. The intersection of the two datasets has a total of 195 targets (that is Celastrol-RA targets) (Fig. 2). The Celastrol-RA targets were input into Cytoscape to construct Celastrol-RA target PPI network. The top 30 targets in Celastrol-RA target PPI network were ALB (107 edges), SRC (86 edges), EGFR (84 edges), HSP90AA1 (82 edges), CASP3 (71 edges), ESR1 (71 edges), IGF1 (69 edges), MAPK1 (69 edges), MMP9 (67 edges), PPARG (61 edges), MAPK14 (58 edges), BCL2L1 (56 edges), MAPK8 (54 edges), ANXA5 (50 edges), GRB2 (49 edges), NOS3 (49 edges), HPGDS (48 edges), STAT1 (47 edges), PIK3R1 (47 edges), MMP2 (47 edges), MDM2 (47 edges), PPARA (44 edges), MAP2K1 (43 edges), IL2 (43 edges), PTPN11 (42 edges), JAK2 (41 edges), IGF1R (41 edges), KDR (41 edges), GSK3B (39 edges), AR (39 edges) (Fig. 3). The topological property of this network was assessed by network analyzer tool, and the result demonstrates this PPI network meets the power-law distribution (R^2^ = 0.441, y = 12.969x^−0.524^) (Fig. 4). Cluster analysis showed that the Celastrol-RA target PPI network was divided into 6 clusters (Fig. 5).

#### Enrichment analysis of Celastrol-RA target PPI network

**Figure 6:**
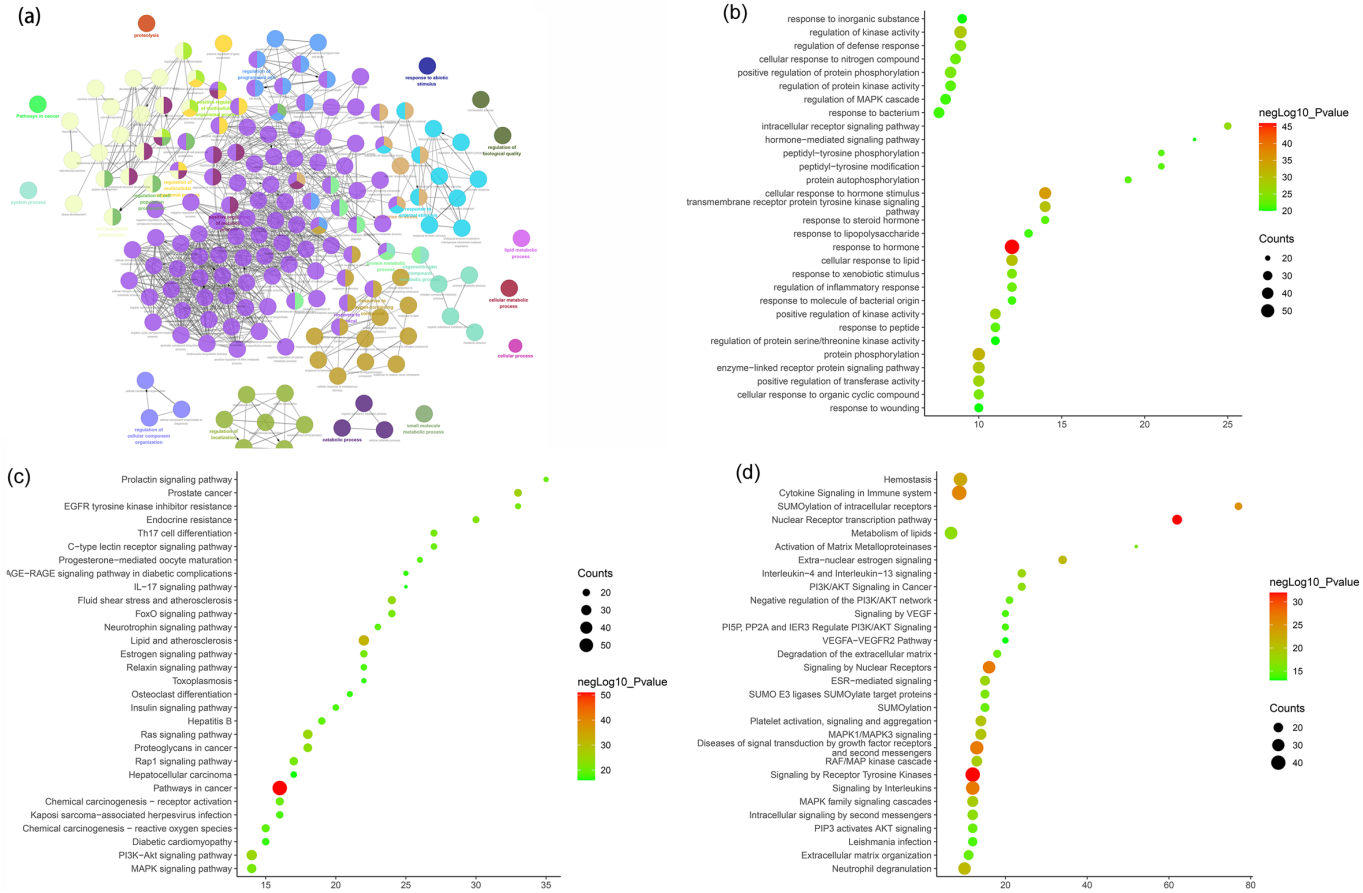
Enrichment analysis of Celastrol-RA target PPI network (**a**: primary enrichment analysis results; **b**: biological processes; **c**: signaling pathways; **d**: Reactome pathway. X-axis stands for fold enrichment).

The biological processes, signaling pathways, Reactome pathways were shown in Fig. 6 and the details of them were shown in Table S1 (see Supplementary Materials).

Through a systematic pharmacological study, it has been discovered that the 195 targets in the Celastrol-RA target PPI network are not independent but exhibit interactive relationships. Enrichment analysis results have shown that the signaling pathways associated with cell proliferation include the PI3K-Akt signaling pathway, Ras signaling pathway, estrogen signaling pathway, MAPK signaling pathway, nuclear receptor transcription pathway, receptor tyrosine kinase signaling pathway, nuclear receptor signaling pathway, growth factor receptor and second messenger signaling pathways, and extracellular estrogen signaling pathway. Additionally, the signaling pathways associated with immune inflammation include Th17 cell differentiation, FoxO signaling pathway, Rap1 signaling pathway, EGFR tyrosine kinase inhibitor resistance, interleukin signaling pathways, cytokine signaling in the immune system. Furthermore, the signaling pathways related to the pathological invasion of fibroblast-like synoviocytes include MAPK1/MAPK3 signaling, signaling and adhesion, RAF/MAP kinase cascade, MAPK family signaling cascades, interleukin-4 and interleukin-13 signaling, neutrophil degranulation, ESR-mediated signaling, second messenger intracellular signaling, lipid metabolism, and activation of matrix metalloproteinases.

RA is characterized by synovial hyperplasia, production of cytokines, chemokines, and autoantibodies such as rheumatoid factor (RF) and anti-citrullinated protein antibodies (ACPA), osteoclast proliferation, angiogenesis, and systemic consequences such as cardiovascular, pulmonary, psychological, and skeletal diseases ^28,29^. This symmetric polyarticular arthritis initially affects freely moving joints such as the shoulder, knee, hip, and hand joints ^29^. RA is typically considered a disease mediated by helper T cells (particularly Th1/Th17 cells), with inflammatory synovium leading to neovascularization and destruction of local joint structures ^30,31^. The synovium usually consists of a relatively cellular structure with intricate internal organization, and in RA, macrophages, plasma cells, dendritic cells, lymphocytes, and immune complexes infiltrate the synovium, forming lymphoid aggregates with germinal centers ^32,33^. RA synovium is enriched with bone marrow cells (monocytes, macrophages, neutrophils, eosinophils, basophils, red blood cells, dendritic cells, and megakaryocytes or platelets) and plasma-like dendritic cells, which produce various cytokines (IL-12, IL-15, IL-18, IL-6, IL-32), HLA class II molecules, and co-stimulatory molecules responsible for T-cell activation and presentation ^34^. Among these, TGF-β, IL-1β, IL-6, IL-21, and IL-23 derived from macrophages and dendritic cells provide the environment for Th17 differentiation, while regulatory T-cell differentiation is suppressed, disturbing the balance within the body ^35^. The synovium harbors a significant number of innate immune cells, such as macrophages, mast cells, and natural killer cells, but the major component is neutrophils. Their maturation and transport from the bone marrow to the synovium are mediated by colony-stimulating factors, including macrophage-CSF, granulocyte-CSF, and granulocyte–macrophage-CSF ^36^. Macrophages are usually considered the main culprits of synovial inflammation, exerting their effects through the release of TNF-α, interleukins (IL-1, IL-6, IL-12, IL-15, IL-18, IL-23), reactive oxygen and nitrogen species, prostaglandins, and matrix-degrading enzymes, as well as phagocytosis and antigen presentation ^37,38^. The activation of these macrophages is achieved through various factors, including Toll-like receptors (TLRs), nucleotide-binding oligomerization domain (NOD) receptors, cytokines, T-cell interactions, immune complexes, lipoprotein particles, liver X receptor agonists, and protease-activating receptors providing an environment rich in proteases ^39,40^. Cytokines and chemokines are proteins that regulate human immune function, and their imbalance can lead to various diseases. The cytokine profile in early RA is characterized by significant expression of IL-14, IL-13, and IL-15, which are derived from T cells and stromal cells, ultimately leading to the development of chronic RA ^41^. In the synovium of RA patients, intracellular signaling pathways actively participate in regulating cytokine and receptor-mediated functions. These pathways include the JAK pathway, spleen tyrosine kinase (Syk), NF-κB pathway, and p38 mitogen-activated protein kinase ^42,43^. Blocking these pathways in RA patients has shown favorable clinical outcomes in the majority of cases.

### Transcriptomic data analysis of Celastrol treatment of RA

#### Differentially expressed genes

**Figure 7:**
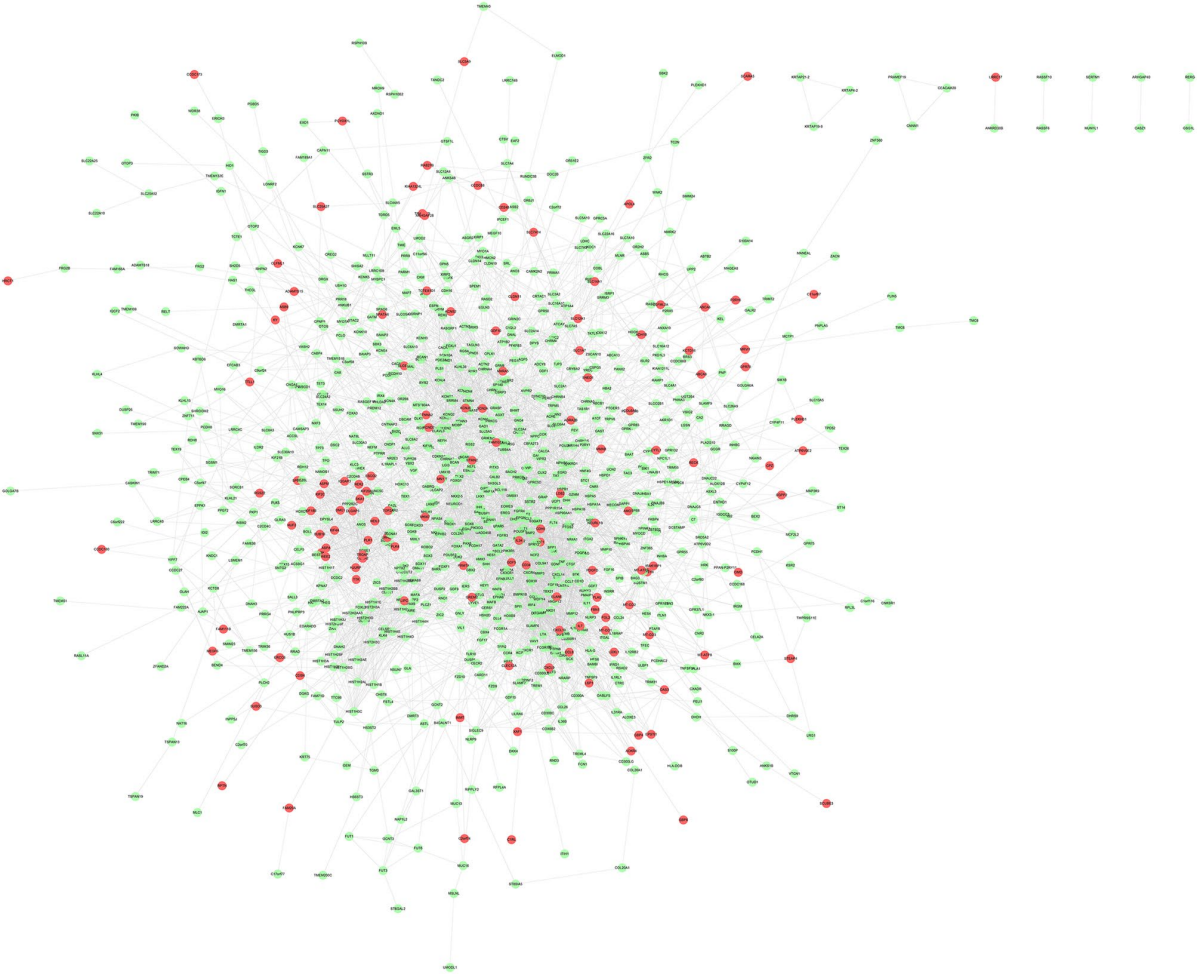
Differentially expressed genes PPI network (Red node stands for up-regulated genes; green node stands for down-regulated genes).

**Figure 8:**
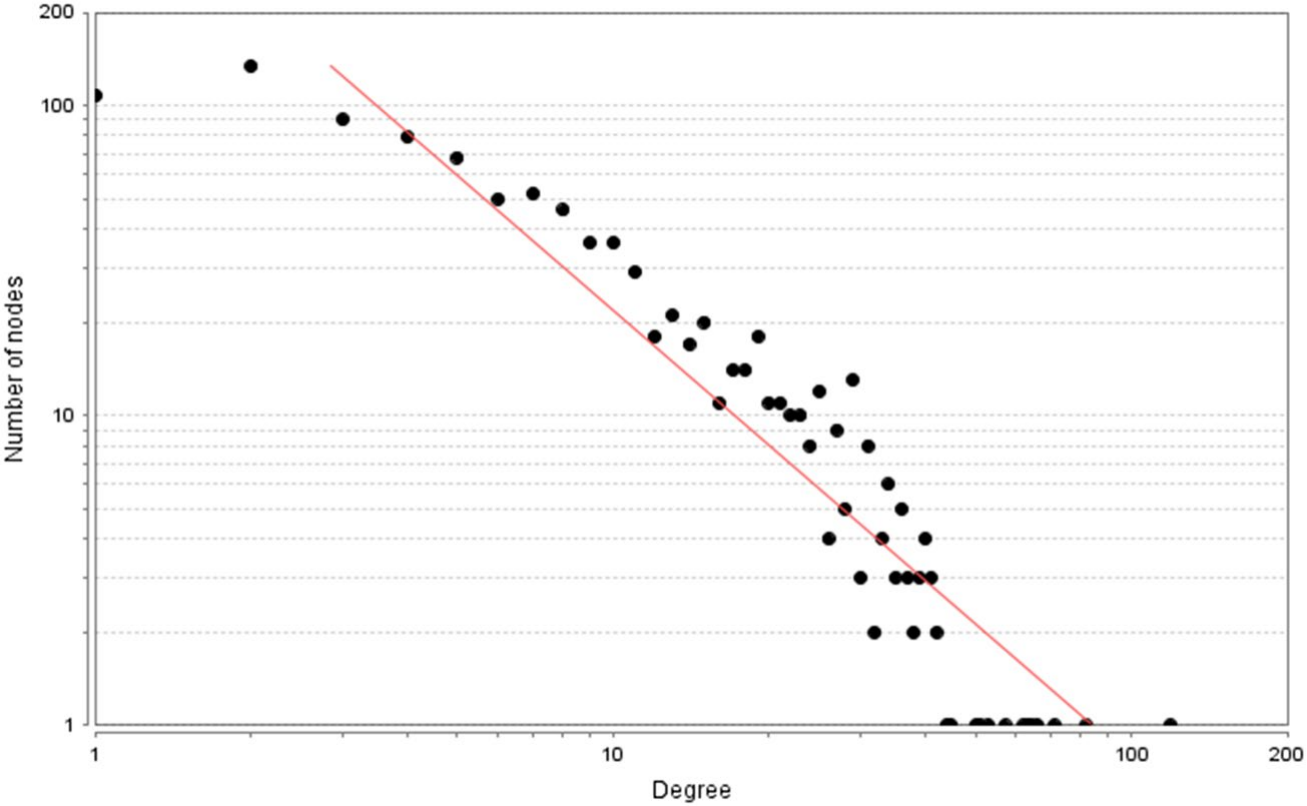
Node degree distribution of Differentially expressed genes PPI network.

**Figure 9:**
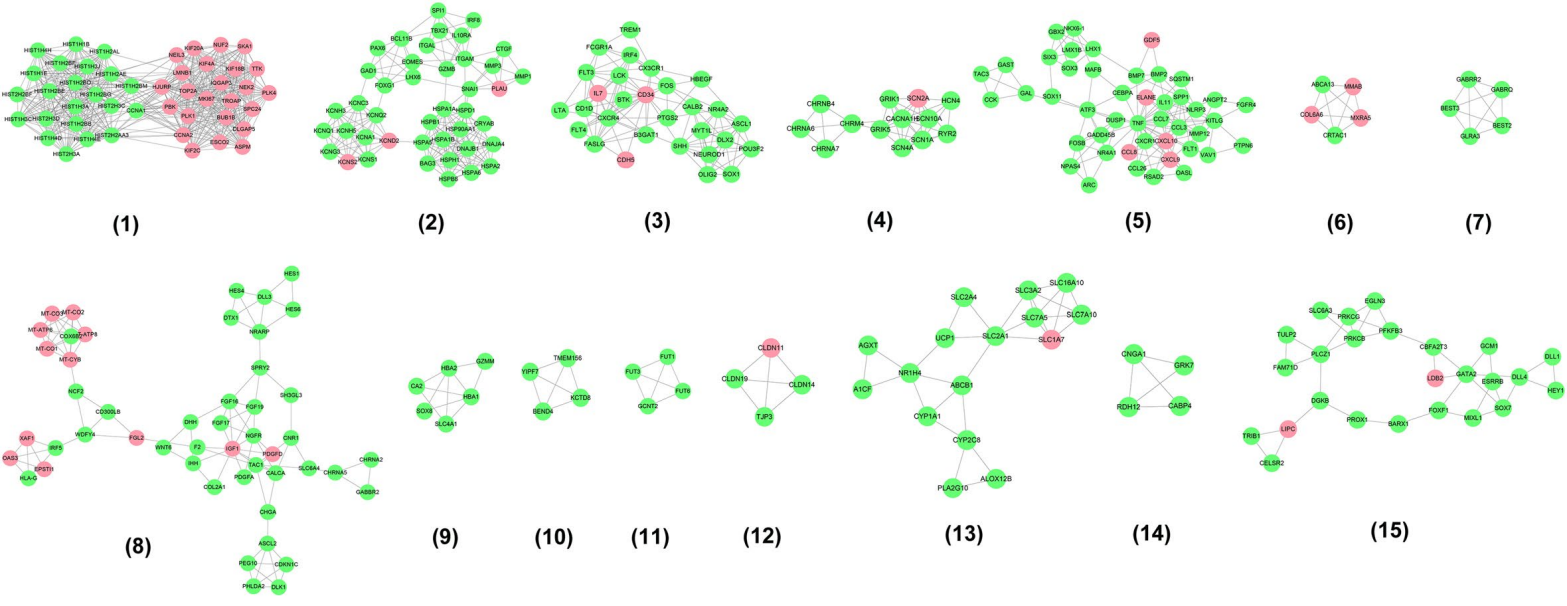
Clusters of differentially expressed genes PPI network (Red node stands for up-regulated genes; green node stands for down-regulated genes).

The results showed that there were 2068 differential genes with log2FC > 3 and < -3 and FDR < 0.05, with 1723 down-regulated genes and 345 up-regulated genes. Those differentially expressed genes were input into Cytoscape to construct differentially expressed genes PPI network (Fig. 7). The topological property of this network was assessed by network analyzer tool, and the result demonstrates this PPI network meets the power-law distribution (R^2^ = 0.878, y = 615.9x^−1.451^) (Fig. 8). Cluster analysis showed that the Celastrol-RA target PPI network was divided into 27 clusters (Fig. 9).

#### Up-regulated genes

**Figure 10:**
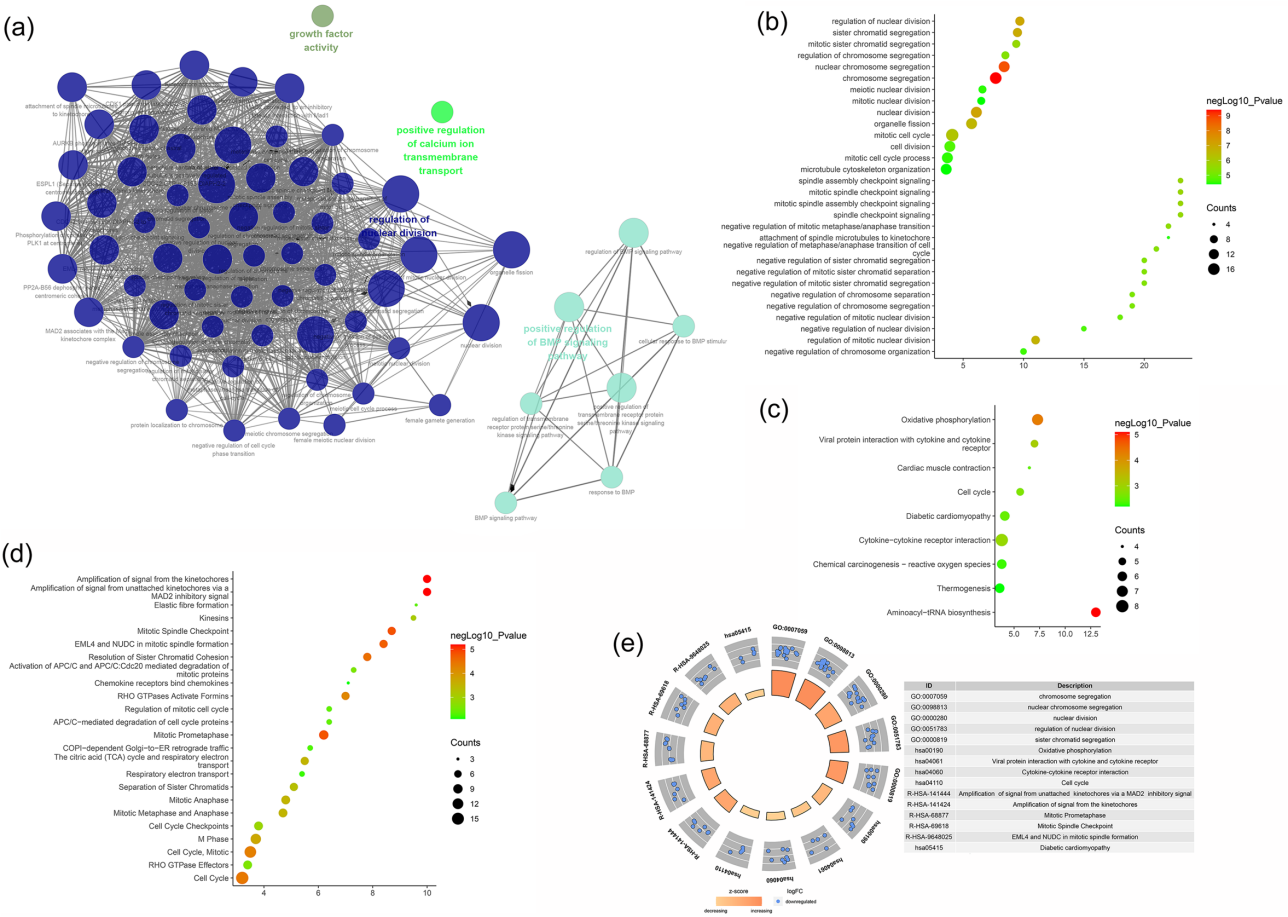
Enrichment analysis of up-regulated genes (**a**: primary enrichment analysis results; **b**: biological processes; **c**: signaling pathways; **d**: Reactome pathway. X-axis stands for fold enrichment. **e**: top 5 enrichment results).

The biological processes, signaling pathways, Reactome pathways were shown in Fig. 10.

#### Down-regulated genes

**Figure 11:**
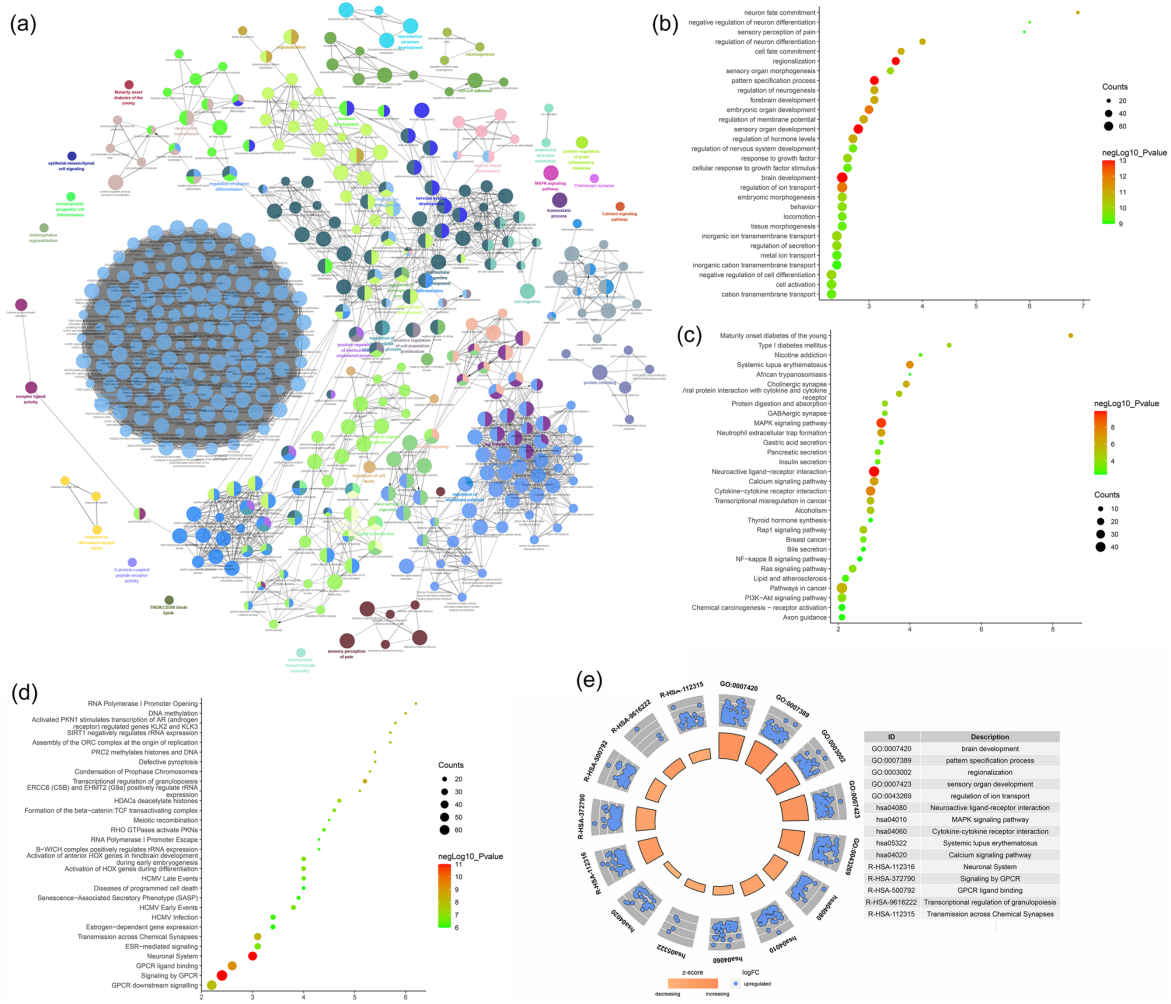
Enrichment analysis of down-regulated genes (**a**: primary enrichment analysis results; **b**: biological processes; **c**: signaling pathways; **d**: Reactome pathway. X-axis stands for fold enrichment. **e**: top 5 enrichment results).

The biological processes, signaling pathways, Reactome pathways were shown in Fig. 11.

#### All genes

**Figure 12:**
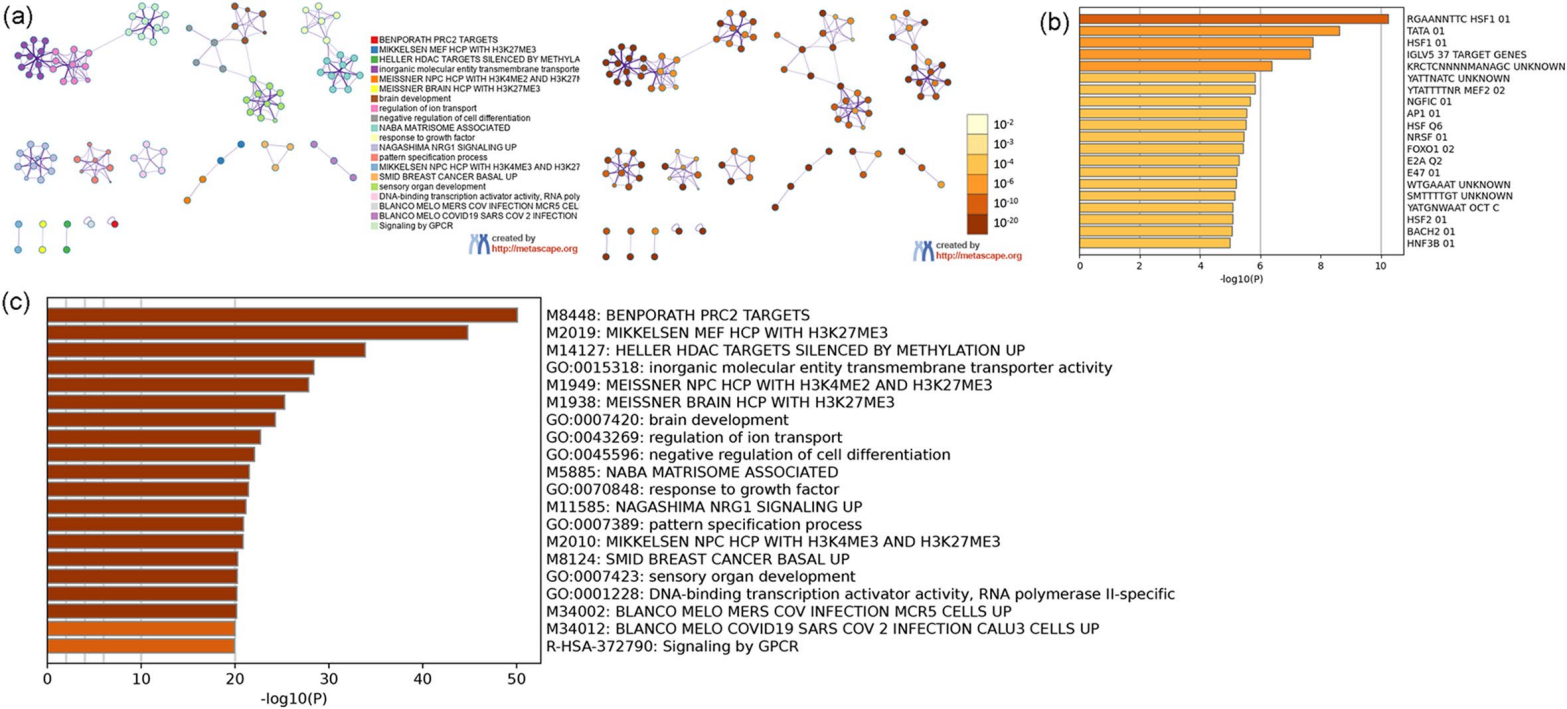
Primary enrichment results (**a**: PPI network colored by cluster and *P*-value; **b**: Heatmap of transcription factor; **c**: Heatmap of enrichment results).

**Figure 13:**
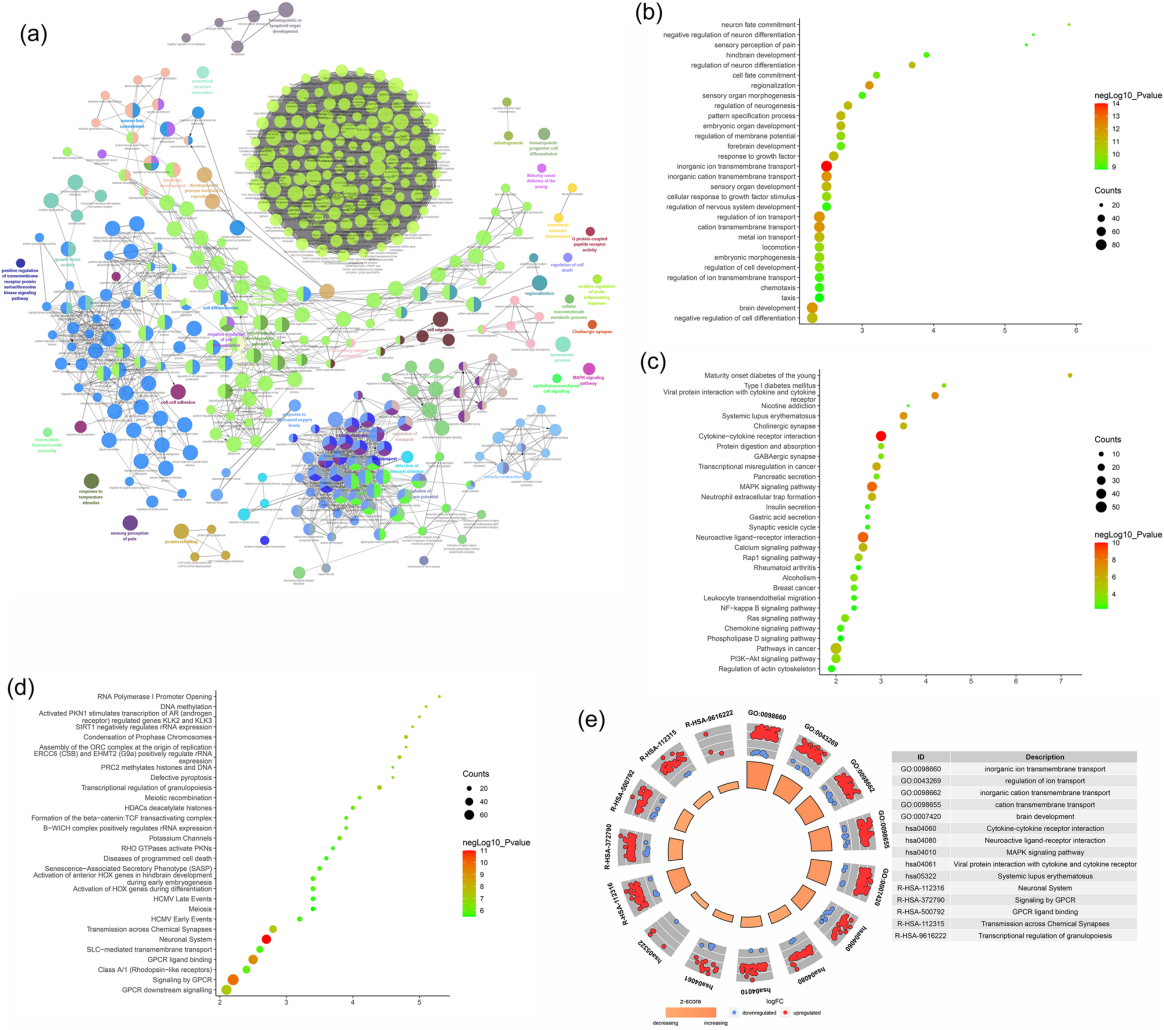
Enrichment analysis of down-regulated genes (**a**: primary enrichment analysis results; **b**: biological processes; **c**: signaling pathways; **d**: Reactome pathway. X-axis stands for fold enrichment. **e**: top 5 enrichment results).

The biological processes, signaling pathways, Reactome pathways were shown in Figs. 12 and 13 and The details were shown in Table S2.

In the pathogenesis and progression of RA, inflammatory cytokines play a crucial role. When the pro-inflammatory effects outweigh the anti-inflammatory effects, it can lead to multi-system immune complications ^44^. For instance, highly activated macrophage-like cells produce various pro-inflammatory cytokines, chemokines, and growth factors, which induce B cells to generate interleukin-6 (IL-6), prostaglandins, and matrix metalloproteinases (MMPs) that contribute to joint immune damage ^11^. Fibroblast-like synoviocytes (FLS) in the synovium, due to their unique invasiveness, abundant production of proteases, and resistance to apoptosis, lead to synovial tissue hyperplasia, triggering inflammation, promoting cartilage erosion and degradation, and causing tissue damage and joint destruction ^45^. Chronically activated T cells in the synovium and joint cavity play crucial roles as initiators and coordinators of the RA inflammatory pathway. They promote the expression of IL-1, tumor necrosis factor (TNF), IL-6, and chemokines in macrophages through cell contact-dependent mechanisms, leading to an imbalance in regulatory T cell (Treg) differentiation, promoting immune-inflammatory reactions, and exacerbating RA pathology ^45,46^. Neutrophils act as bridges between FLS and T cell responses, with activated neutrophils in the inflammatory tissue expressing various pro-inflammatory cytokines and chemokines. These inflammatory factors further promote the migration of neutrophils from the circulation, thereby exacerbating inflammation and cartilage destruction ^47^. Moreover, these inflammatory factors regulate RA inflammation through signaling pathways such as JAK/STAT, NF-κB, and MAPK, leading to dysregulation of immune-inflammatory reactions, imbalance of dendritic cells, mast cells, natural killer cells, macrophages, Th1/Th2, and Th17/Treg ratios, and contributing to the progression and development of RA pathology ^48–50^.

PPI analysis in transcriptomics reveals that celastrol, an intervention in RA, downregulates key targets associated with inflammation and immune regulation, such as TNF, CXCR4, SPI1, PTGS2, ASCL1, IRF4, and CCL3. Other targets are involved in cell proliferation, angiogenesis, bone remodeling, fibrosis, including FOS, ITGAM, SHH, HSP90AA1, PAX6, GAD1, KCNA1, FOXG1, LCK, EGR2, EOMES, SPP1, BMP2, and FLT3. TNF is especially well-known as a target in RA, with the most important downstream signaling pathway mediated by NF-κB activation, participating in inflammation, anti-apoptosis, and immune responses. TNF-α can activate the NF-κB signaling pathway to exert pro-inflammatory effects ^51^. Additionally, TNF-α has other functions. For example, in RA, TNF-α promotes the activation of leukocytes and endothelial cells, cascading cytokine and chemokine responses, angiogenesis, and activation of nociceptive receptors ^52^. Studies have found that TNF-α regulates osteoclastogenesis and inhibits osteoblast differentiation, disrupting the balance between osteoclasts and osteoblasts, resulting in joint damage ^53^. Moreover, in synovial cells obtained from RA patients, TNF-α blockade significantly reduces the production of other pro-inflammatory factors, such as IL-1, IL-6, IL-8, and granulocyte–macrophage colony-stimulating factor (GM-CSF), exerting anti-inflammatory and anti-rheumatic effects ^54^. In recent years, the successful application of TNF-α monoclonal antibodies (e.g., infliximab, etanercept) in clinical practice has further demonstrated the importance of TNF-α. Blocking TNF-α can effectively disrupt signal transmission, improve synovial hypoxia, synovial inflammation, and inhibit angiogenesis ^55^.

### Proteomic data analysis of Celastrol treatment of RA

#### Differentially expressed proteins

**Figure 14:**
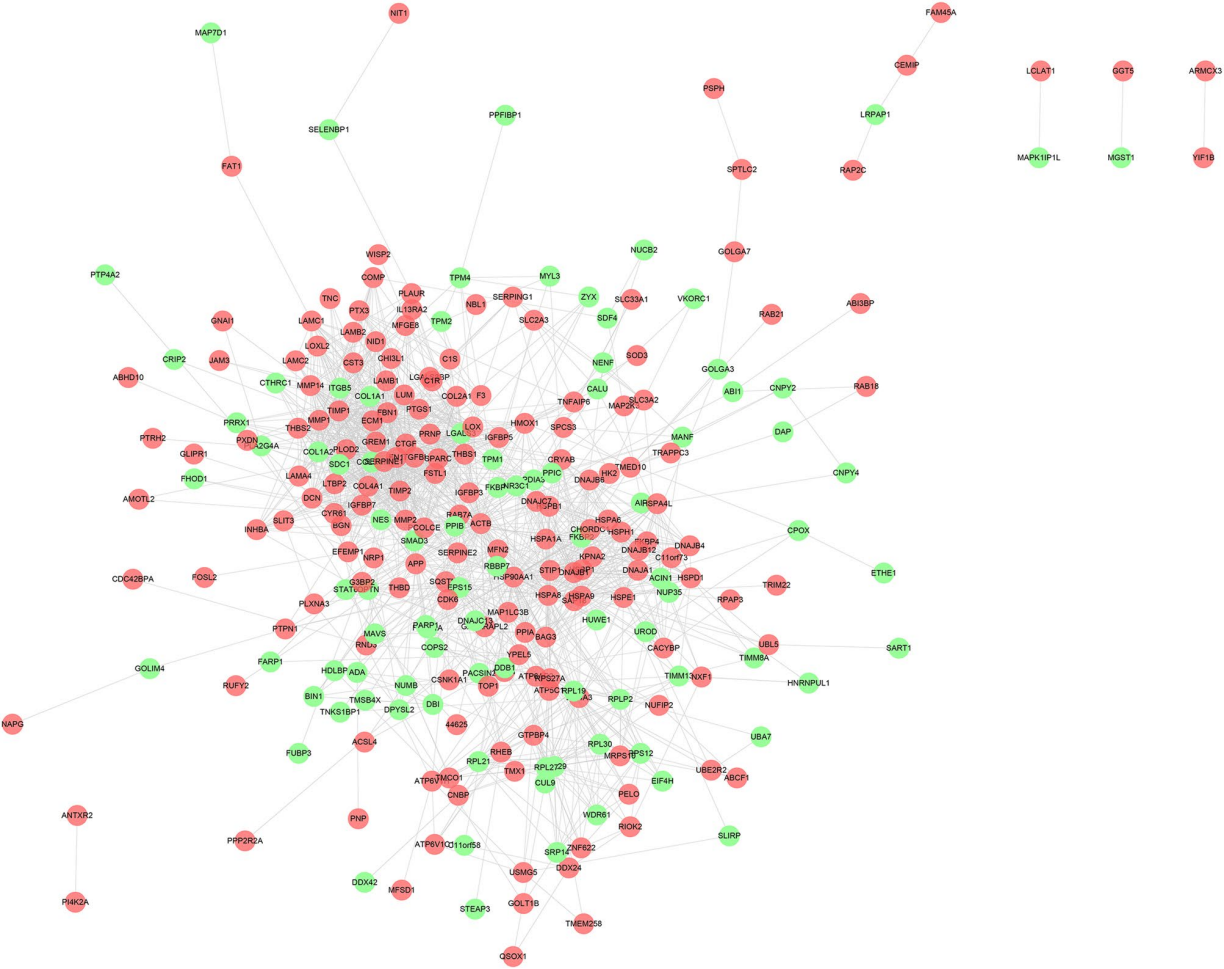
Differentially expressed proteins PPI network (Red node stands for up-regulated genes; green node stands for down-regulated genes).

**Figure 15:**
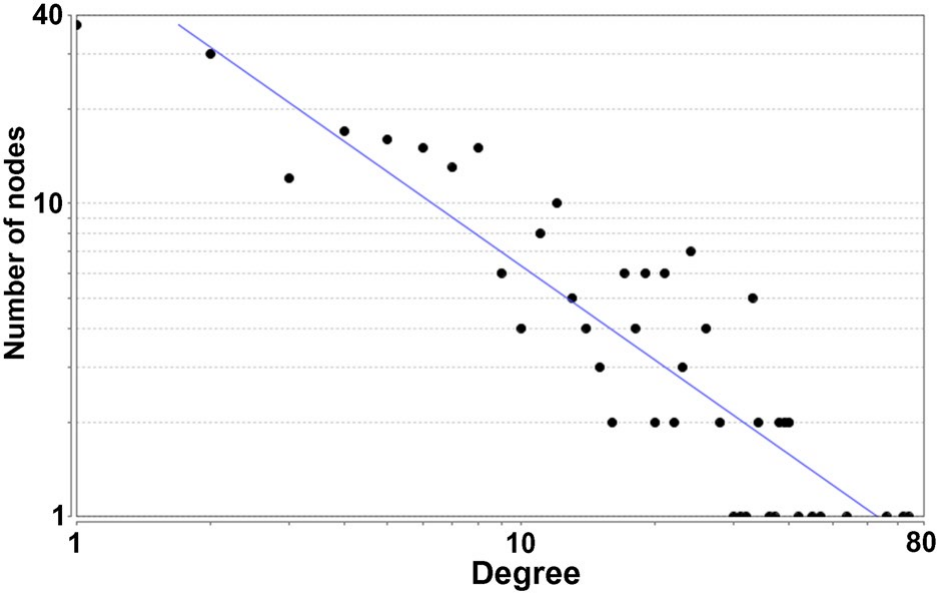
Node degree distribution of Differentially expressed proteins PPI network.

**Figure 16:**
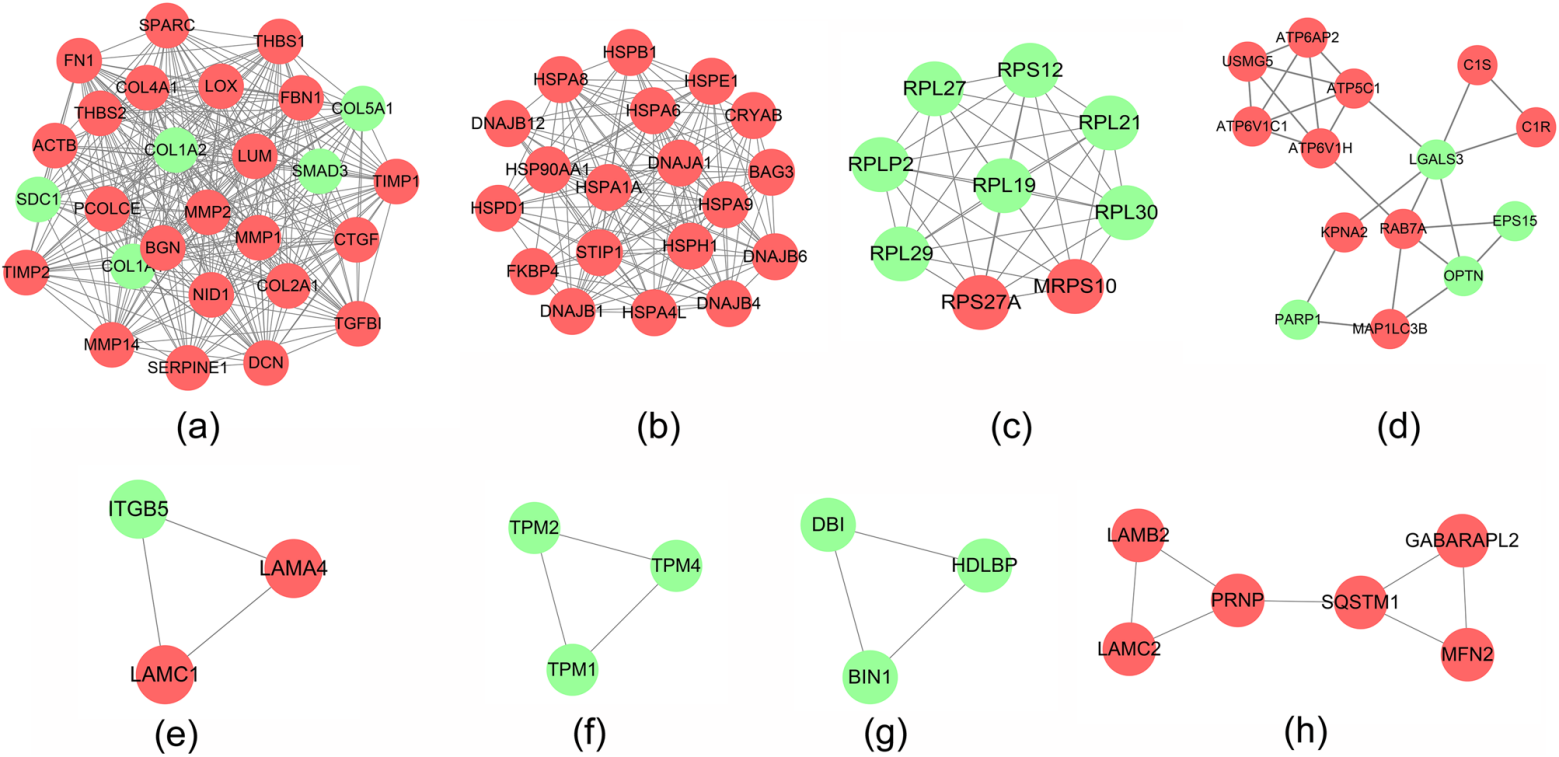
Clusters of differentially expressed proteins PPI network (Red node stands for up-regulated genes; green node stands for down-regulated genes).

The results showed that there were 294 differential proteins with log2FC > 1 and < -1 and *P* < 0.05, with 188 up-regulated proteins and 106 down-regulated proteins. Those differentially expressed proteins were input into Cytoscape to construct differentially expressed genes PPI network. The top 20 targets in up-regulated genes sets were: ACTB (74 edges), FN1 (72 edges), HSP90AA1 (66 edges), HSPA8 (54 edges), MMP2 (45 edges), COL4A1 (40 edges), CTGF (40 edges), THBS1 (39 edges), APP (39 edges), HSPA9 (38 edges), TIMP1 (37 edges), LOX (36 edges), RPS27A (34 edges), HSPA1A (34 edges), FBN1 (33 edges), BGN (33 edges), SPARC (33 edges), SERPINE1 (33 edges), MMP14 (32 edges), LUM (31 edges). The top 22 targets in down-regulated genes sets were: COL1A1 (47 edges), COL1A2 (42 edges), COL5A1 (38 edges), SMAD3 (33 edges), ITGB5 (24 edges), SDC1 (24 edges), PDIA3 (23 edges), LGALS3 (23 edges), RPL27 (22 edges), RPL19 (21 edges), RPL30 (21 edges), RPL29 (19 edges), PPIB (19 edges), RPL21 (16 edges), RPS12 (15 edges), RPLP2 (14 edges), FKBP1A (13 edges), PARP1 (12 edges), OPTN (12 edges), EPS15 (11 edges), DDB1 (11 edges), NES (11 edges) (Fig. 14). The topological property of this network was assessed by network analyzer tool, and the result demonstrates this PPI network meets the power-law distribution (R^2^ = 0.812, y = 62.594x^−0.998^) (Fig. 15). Cluster analysis showed that the Celastrol-RA target PPI network was divided into 8 clusters (Fig. 16).

#### Up-regulated proteins

**Figure 17:**
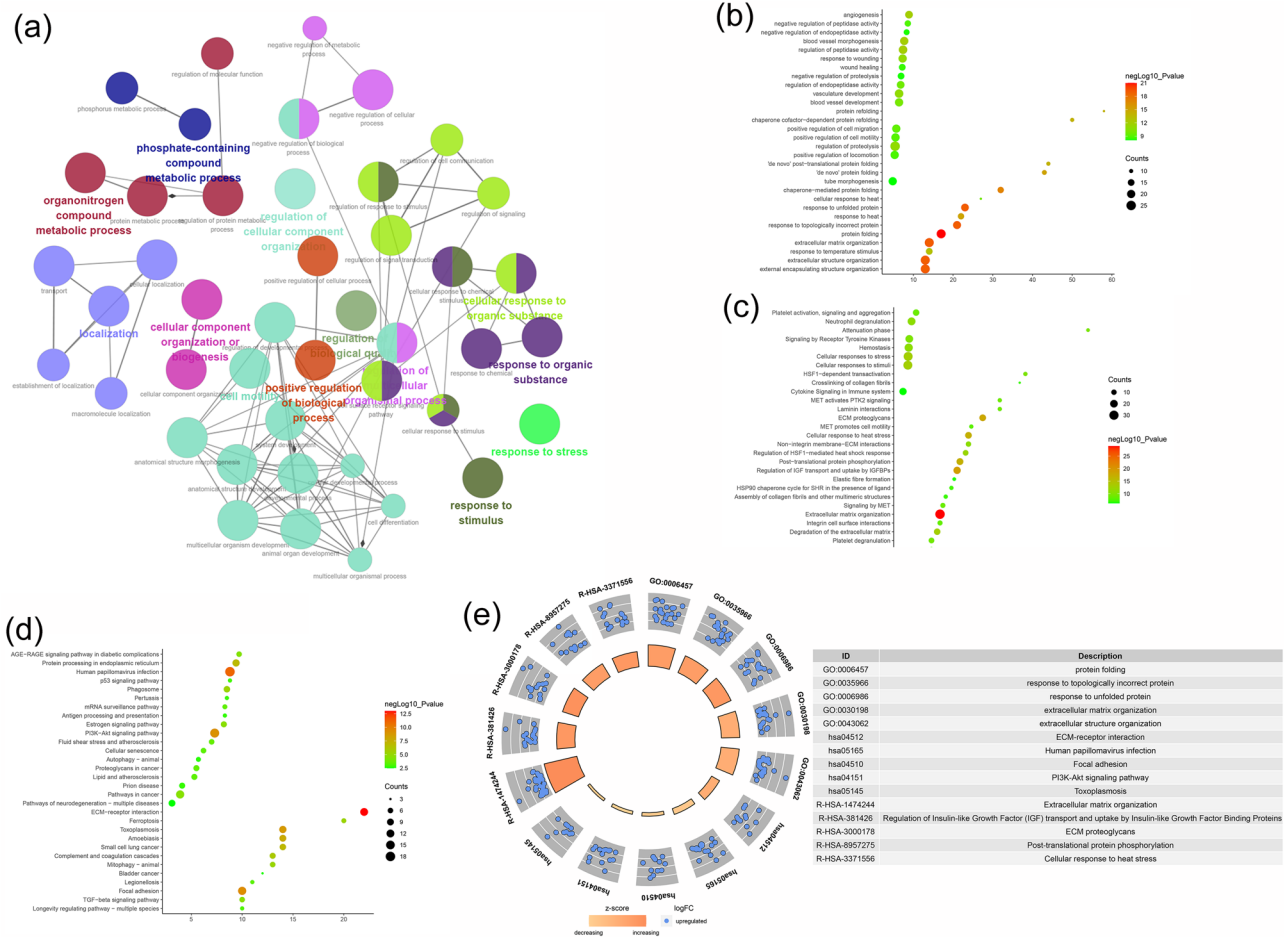
Enrichment analysis of up-regulated genes (**a**: primary enrichment analysis results; **b**: biological processes; **c**: signaling pathways; **d**: Reactome pathway. X-axis stands for fold enrichment. **e**: top 5 enrichment results).

The biological processes, signaling pathways, Reactome pathways were shown in Fig. 17.

#### Down-regulated proteins

**Figure 18:**
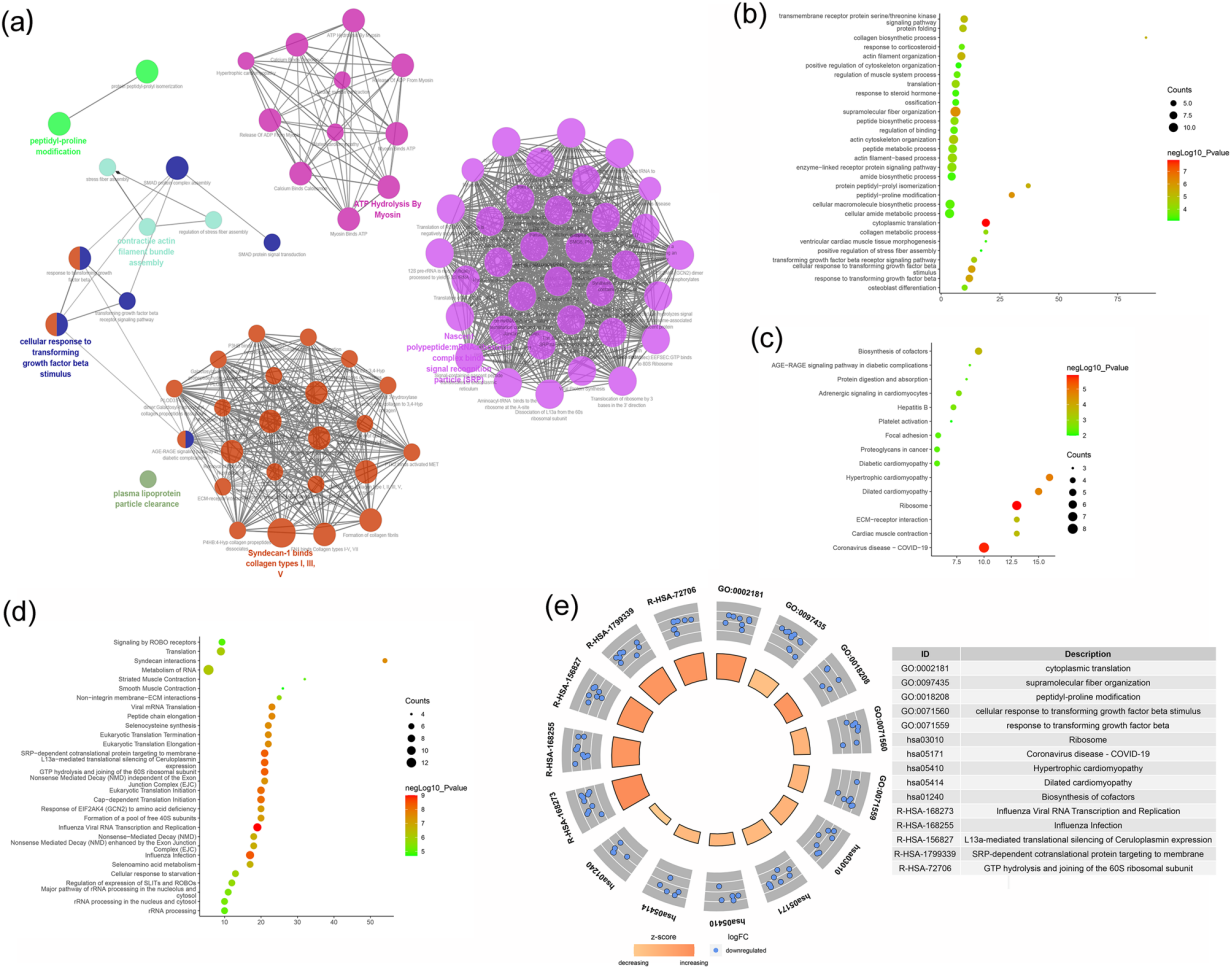
Enrichment analysis of down-regulated genes (**a**: primary enrichment analysis results; **b**: biological processes; **c**: signaling pathways; **d**: Reactome pathway. X-axis stands for fold enrichment. **e**: top 5 enrichment results).

The biological processes, signaling pathways, Reactome pathways were shown in Fig. 18.

#### All proteins

**Figure 19:**
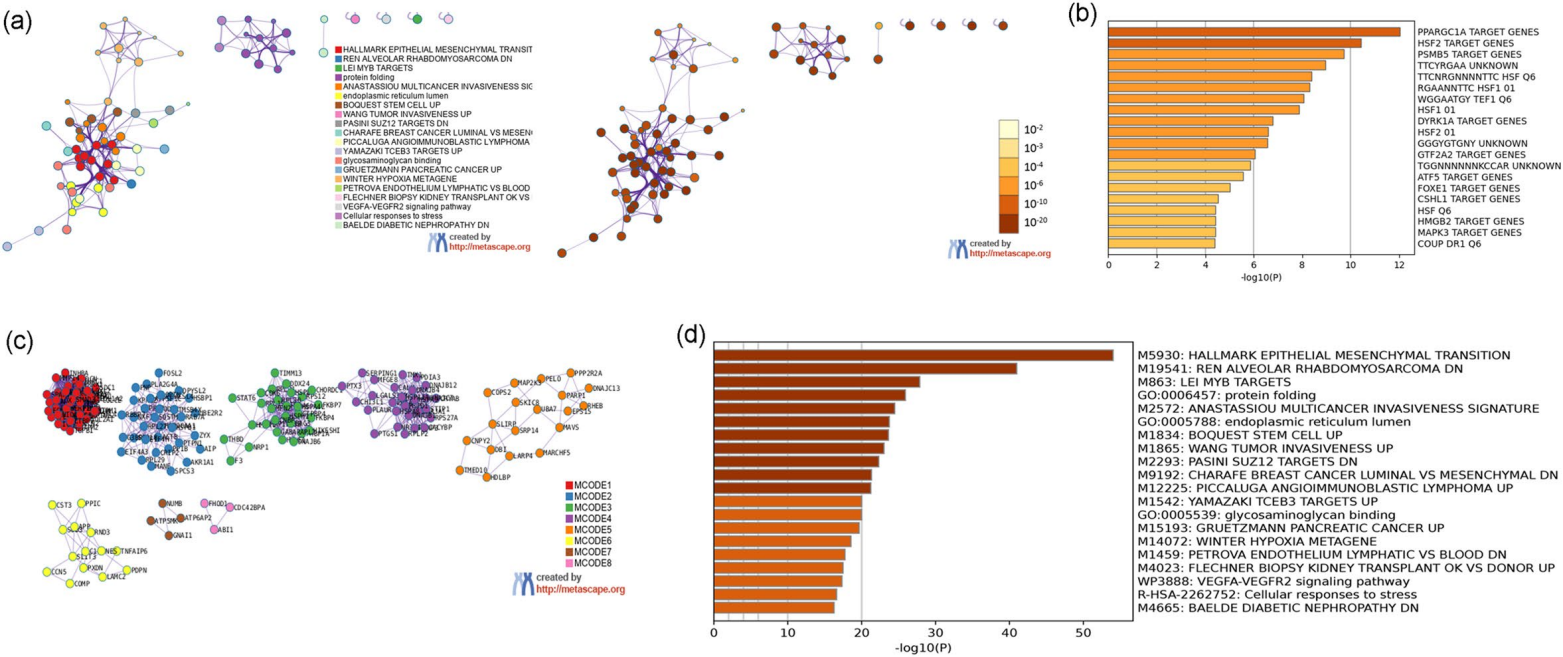
Primary enrichment results (**a**: PPI network colored by cluster and *P*-value; **b**: Heatmap of transcription factor; **c**: Heatmap of enrichment results).

**Figure 20:**
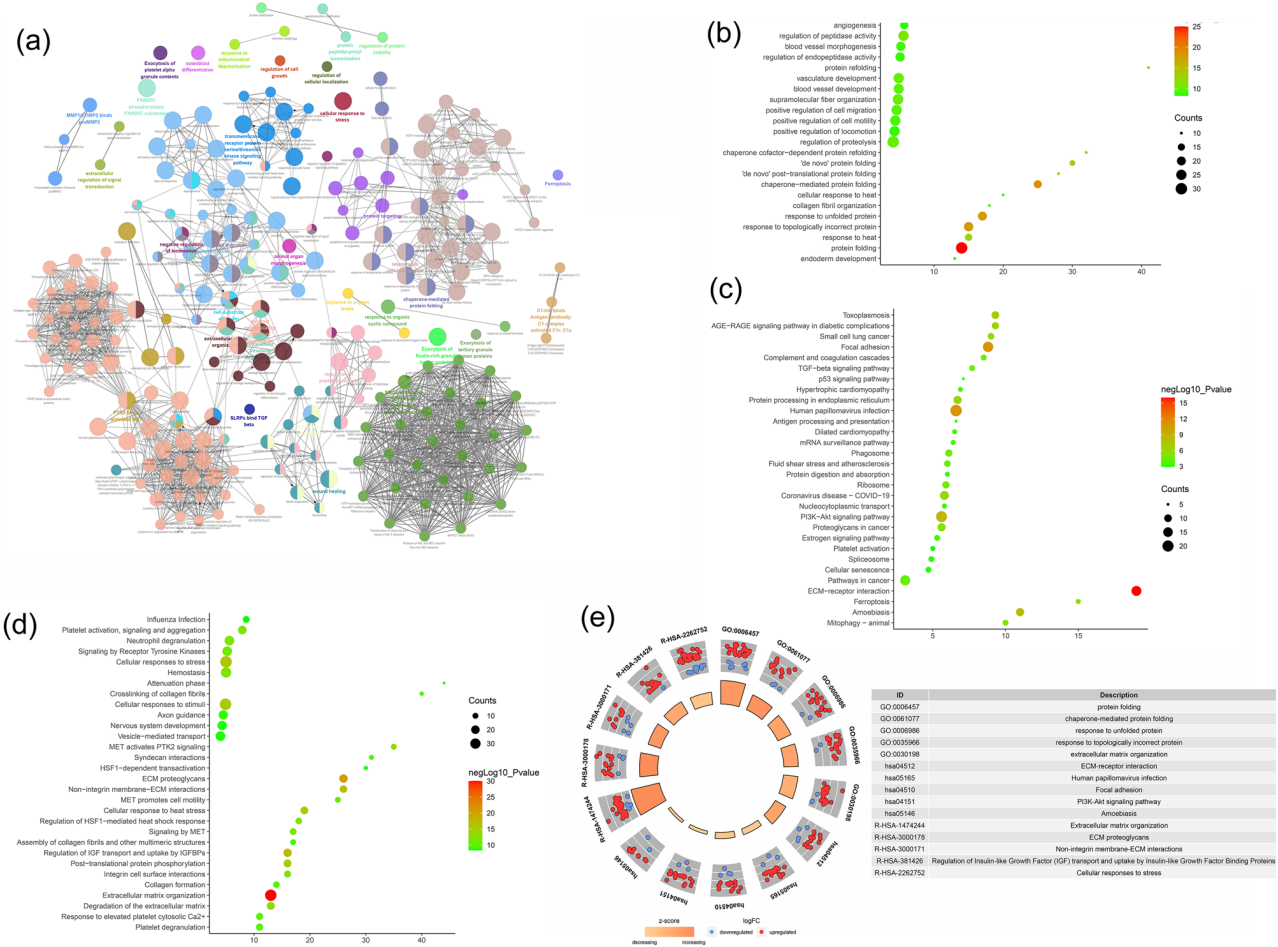
Enrichment analysis of all genes (**a**: primary enrichment analysis results; **b**: biological processes; **c**: signaling pathways; **d**: Reactome pathway. X-axis stands for fold enrichment. **e**: top 5 enrichment results).

The biological processes, signaling pathways, Reactome pathways were shown in Figs. 19 and 20, and The details were shown in Table S3.

The enrichment analysis of proteomics data showed that the biological processes and pathways mainly focused on supramolecular fiber organization, peptide-palmitoylation, response to transforming growth factor-beta (TGF-β) stimulation, TGF-β response, TGF-β receptor signaling pathway, ECM-receptor interaction, PI3K-Akt signaling pathway, protein processing in the endoplasmic reticulum, ferroptosis, mitochondrial autophagy, phagosome, TGF-β signaling pathway, extracellular matrix organization, ECM proteoglycans, integrin-ECM interaction, IGFBP regulation of IGF transport and uptake, response to cellular stress, response to heat stress, post-translational protein phosphorylation, response to stimulus, extracellular matrix degradation, neutrophil degranulation, integrin-cell surface interaction, regulation of HSF1-mediated heat shock response, MET promoting cell motility, receptor tyrosine kinase signaling, platelet degranulation, and response to cytosolic Ca2+elevation. Studies have demonstrated that the destruction of cartilage and non-osseous supporting structures in RA is primarily caused by FLS ^56^. In RA, FLS-mediated inflammatory response, angiogenesis, and bone destruction play crucial pathological roles in the development of rheumatoid arthritis ^57^. Additionally, new inflammatory mediators and their associated cell signaling events have been found to be involved in RA, including IL-21, IL-22, IL-23, IL-27, IL-32, IL-33, IL-34, IL-35, IL-36, IL-37, IL-38, etc. ^58,59^. Among these, IL-21 induces the activation of PI3K, STAT-3, and ERK 1/2 pathways, promoting FLS secretion of MMP-2, MMP-3, MMP-9, and MMP-13 ^59^, while antimicrobial peptide LL-37 can inhibit the secretion of MMP-1 by chondrocytes ^60^.

MMPs are highly expressed in the synovium, synovial fluid, and peripheral blood of RA patients, and play a significant role in the process of joint inflammation and cartilage destruction. MMP-1 and MMP-3 are involved in joint damage, with MMP-3 being a key protease causing joint injury ^61^, while MMP-2 and MMP-9 participate in bone erosion, and MMP-13 can degrade collagen in the cartilage matrix, leading to cartilage destruction ^62^. Certain pro-inflammatory cytokines can influence the invasiveness of synovium, such as IL-21, which can promote the migration and invasion of FLS in RA, as well as stimulate the secretion of MMPs ^59^. Under the stimulation of inflammatory cytokines such as TNF-α, IL-1, IL-6, and IL-17, FLS exhibit increased expression of receptor activator of nuclear factor-kappa B ligand (RANKL) on their surface. RANKL specifically binds to receptor activator of nuclear factor-kappa B (RANK) on the surface of osteoclast precursors, promoting osteoclast differentiation and bone resorption, ultimately leading to joint bone erosion and destruction ^63,64^.

### Transcriptomics-proteomics integrated analysis

**Figure 21:**
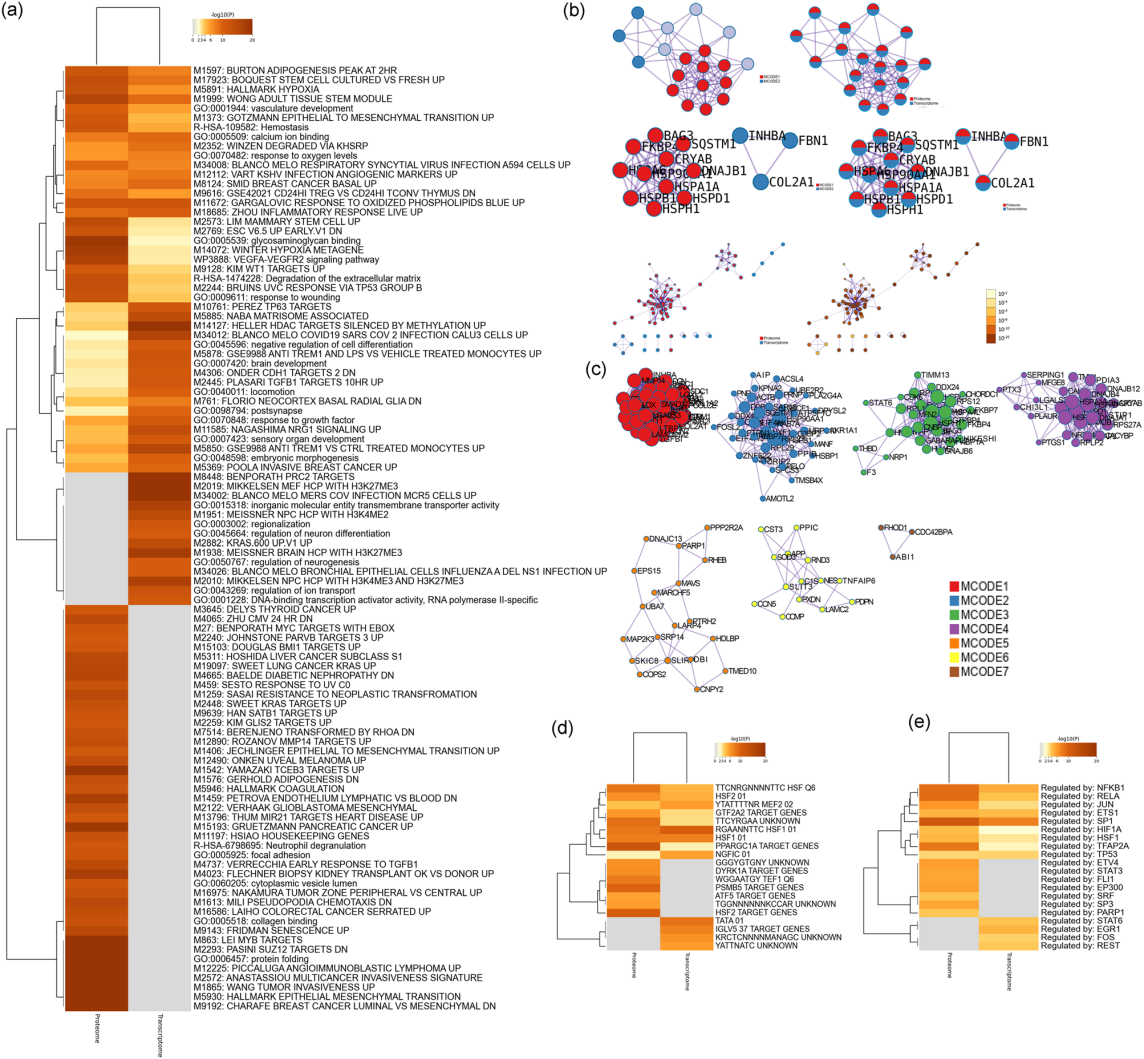
Transcriptomics-Proteomics Integrated Analysis (**a**: Heatmap of top 100 enrichment analysis results; **b**: PPI network colored by counts or clusters; **c**: clusters; **d**: heatmap of transcription factors; **e**: heatmap of TRRUST).

The development of RA is a complex network involving abnormalities in epigenetic and transcriptional gene expression levels. In this study, the mechanism of Celastrol interfering with RA was analyzed through multi-omics integration data, which can provide an in-depth study of biological explanations and provide a new research angle for analyzing the molecular biological network of Celastrol in the treatment of rheumatoid arthritis. The results of transcriptomics-proteomics integrated analysis showed that the top enriched representative signaling pathways were: ECM-receptor interaction, adhesion, PI3K-Akt signaling pathway, ferroptosis, mitophagy, TGF-β signaling pathway, estrogen signaling pathway, NOD-like receptor signaling pathway, cytokine-cytokine receptor interaction, MAPK signaling pathway, systemic lupus erythematosus, neutrophil extracellular trap formation, calcium signaling pathway, PI3K-Akt signaling pathway, chemokine signaling pathway, NF-κB signaling pathway, regulation of actin cytoskeleton, rheumatoid arthritis. The top Reactome pathways include extracellular matrix organization, ECM proteoglycans, non-integrin membrane-ECM interactions, cellular response to heat stress, degradation of extracellular matrix, MET activation of PTK2 signaling, signaling through MET, neutrophil degranulation, Integrin cell surface interaction, HSF1-mediated regulation of heat shock response, MET-promoted cell motility, receptor tyrosine kinase signaling, collagen formation (Fig. 21). In summary, it can be found that Celastrol can directly interfere with the extracellular matrix, inhibit the proliferation and survival of synovial fibroblasts, and inhibit inflammatory chemokines and their mediated immune-inflammatory signal transduction. The multi-omics strategy of this study provides a good case study for future exploration of the interaction mechanism of natural active ingredients in the treatment of rheumatoid bone diseases and autoimmune diseases, and enhances the understanding of the mechanism of Celastrol, a new drug that is expected to be used to treat RA. This has laid a strong theoretical foundation for Celastrol to be developed into a novel type of anti-inflammatory drug, which is widely used in the treatment of autoimmune diseases and many inflammatory diseases. This study has great application value and practical significance for promoting Celastrol to become a safe and effective emerging drug that meets the requirements of modern medicine.

### Single-cell sequencing data analysis

#### Cell cluster identification

**Figure 22:**
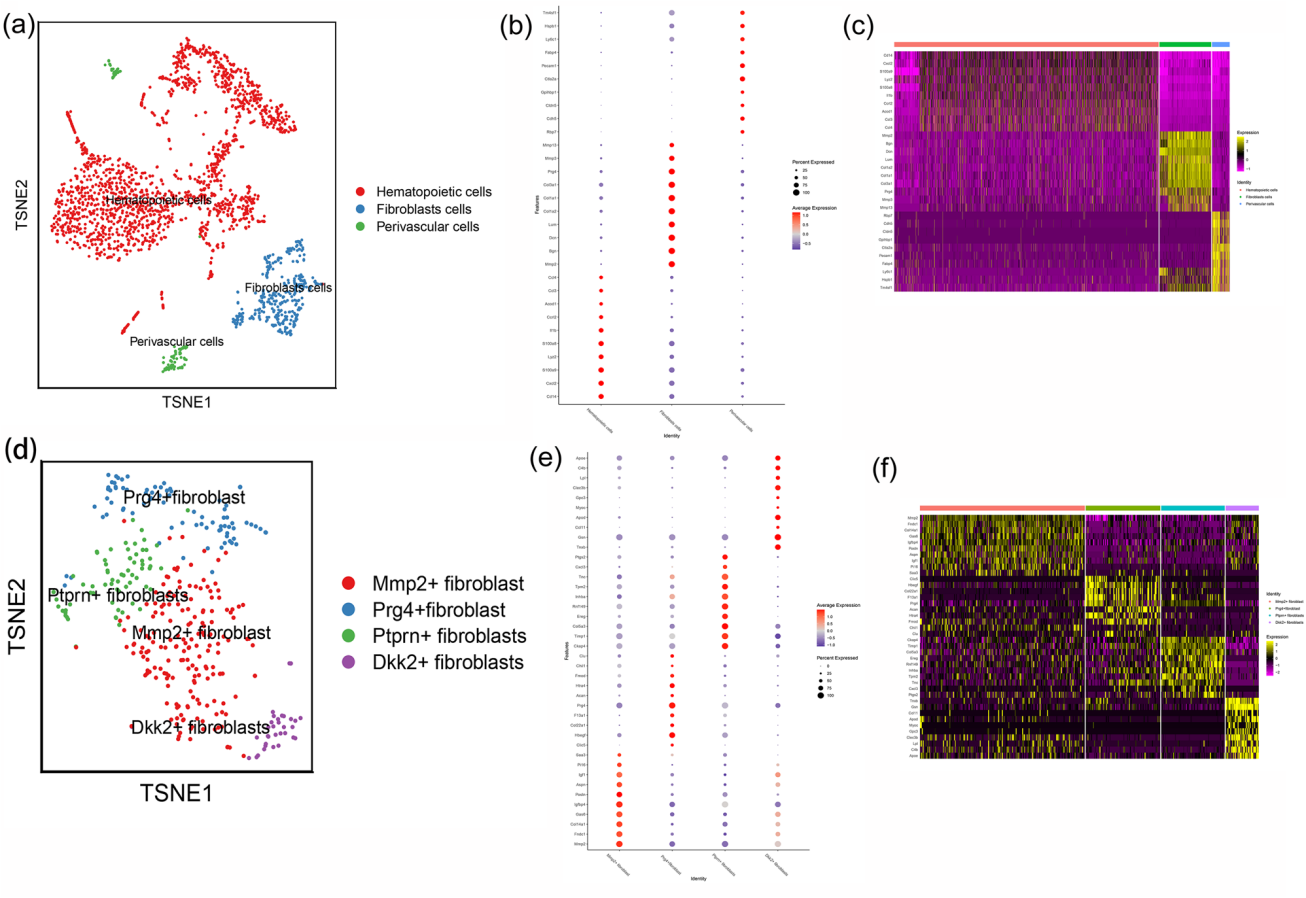
Cell cluster identification (a: identified cell clusters of synovial cells; b: Average expression of top 10 genes of cell clusters; c: Expression of top 10 gene of cell clusters in single cells; d: identified cell clusters of fibroblasts; e: Average expression of top 10 genes of fibroblasts; f: Expression of top 10 gene of cell clusters in fibroblasts).

Cells with nFeature_RNA > 200 and < 2500, and percent.mt < 20% were retained for further analysis. Data after quality control was further processed to generate tSNE cluster plots. These cells were divided into 3 groups. The marker of those synovial cells were shown in Fig. 20. Based on the markers of these cell clusters, a total of 3 cell clusters were identified: Hematopoietic cells, Fibroblasts cells and Perivascular cells. The Fibroblasts cells were further divided into 4 groups, and were identified to: Mmp2+fibroblast, Prg4+fibroblast, Ptprn+fibroblasts, Dkk2+fibroblasts (Fig. 22). The genes in Cluster 1, 2, 3 in PPI networks were mapped to cell clusters to further illustrate that Celastrol may regulate cells by regulating potential targets, thereby improving RA.

#### Distribution of network pharmacology clusters in synovial cells and fibroblasts

**Figure 23:**
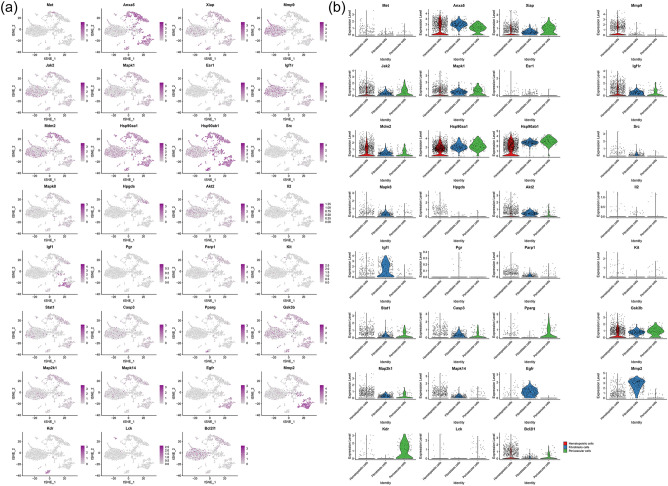
Distribution of network pharmacology cluster 1 in synovial cells (**a**: distribution of genes; **b**: average expression of genes).

**Figure 24:**
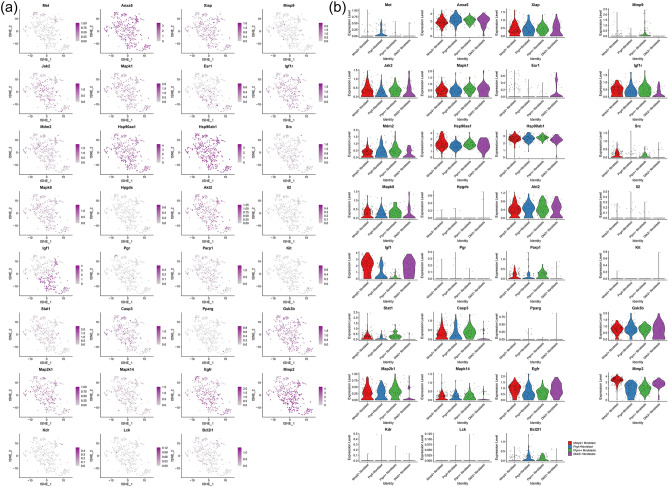
Distribution of network pharmacology cluster 1 in fibroblasts (**a**: distribution of genes; **b**: average expression of genes).

It was found that the genes in these subclusters had obvious distribution differences, for example, Anxa5 was mainly distributed in Hematopoietic cells and Fibroblasts cells; Mmp9 was mainly distributed in Hematopoietic cells and so on (Fig. 23). In Fibroblasts cells, Met is mainly distributed in Prg4+fibroblast, Mmp9 is mainly distributed in Ptprn+fibroblast, etc. (Fig. 24). See "Distribution of other clusters in synovial cells and fibroblasts" for cluster 2 and cluster 3 results.

#### Distribution of transcriptomic clusters in synovial cells and fibroblasts

**Figure 25:**
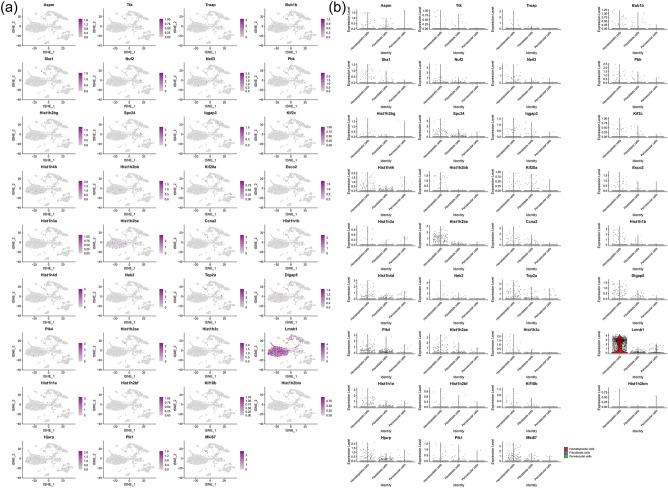
Distribution of transcriptomic cluster 1 in synovial cells (**a**: distribution of genes; **b**: average expression of genes).

**Figure 26:**
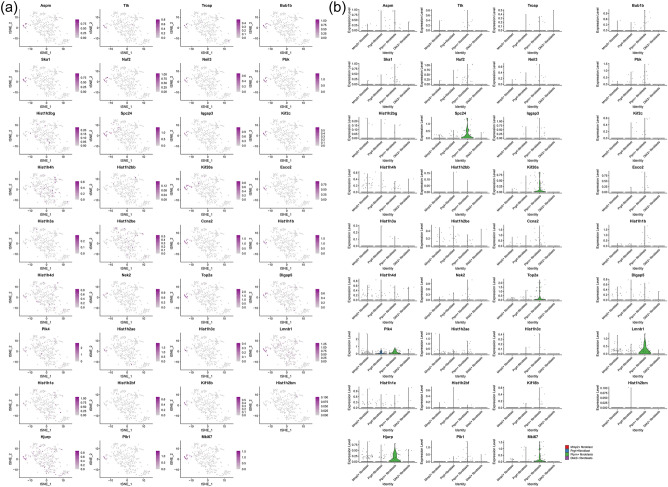
Distribution of transcriptomic cluster 1 in fibroblasts (**a**: distribution of genes; **b**: average expression of genes).

It was found that the genes in these subclusters had obvious distribution differences, for example, Lmnb1 was mainly distributed in Hematopoietic cells and so on (Fig. 25). In Fibroblasts cells, Spc24 is mainly distributed in Ptprn+fibroblast, etc. (Fig. 26). See "Distribution of other clusters in synovial cells and fibroblasts" for cluster 2 and cluster 3 results.

#### Distribution of proteomics clusters in synovial cells and fibroblasts

**Figure 27:**
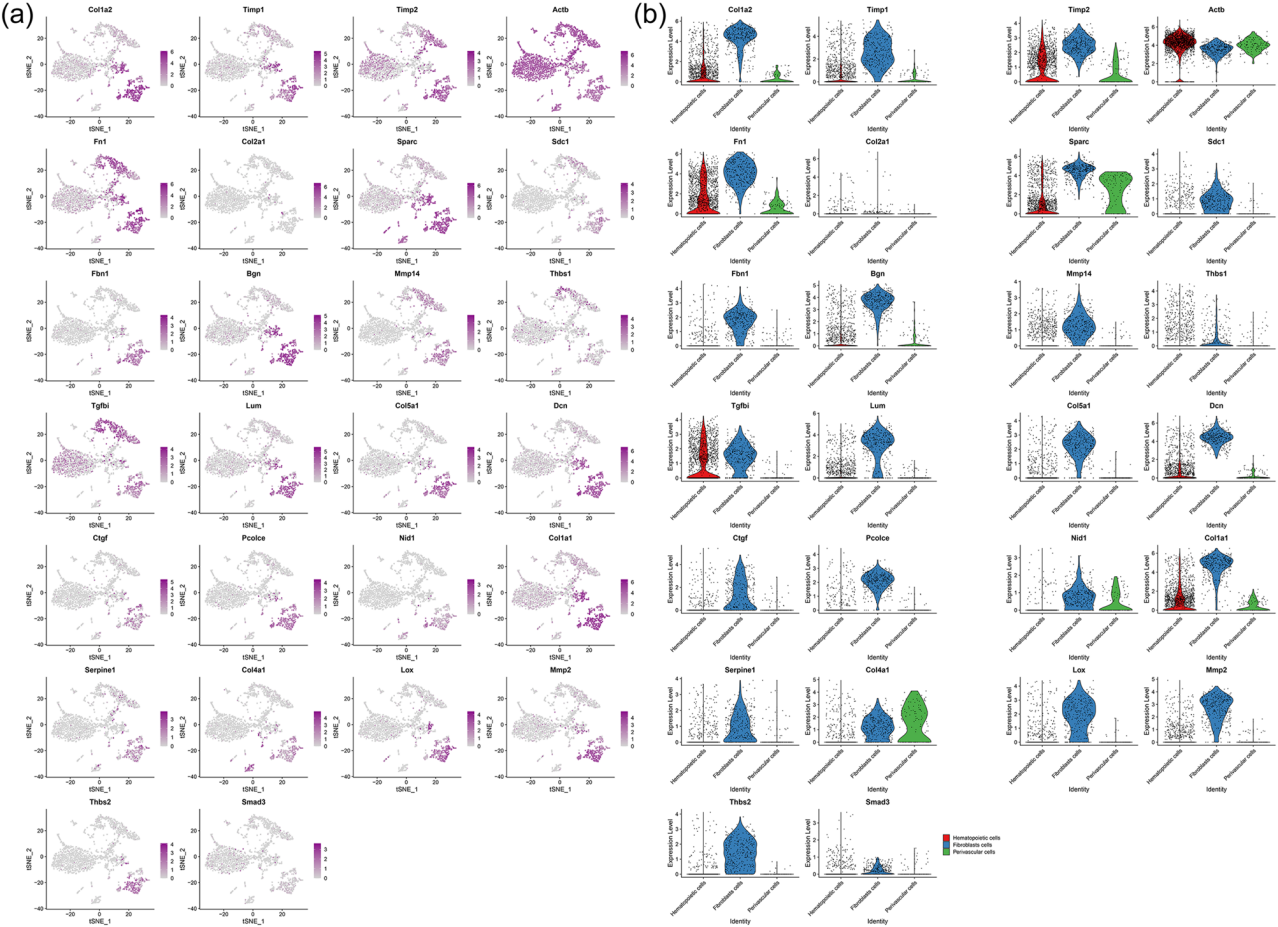
Distribution of proteomics cluster 1 in synovial cells (**a**: distribution of genes; **b**: average expression of genes).

**Figure 28:**
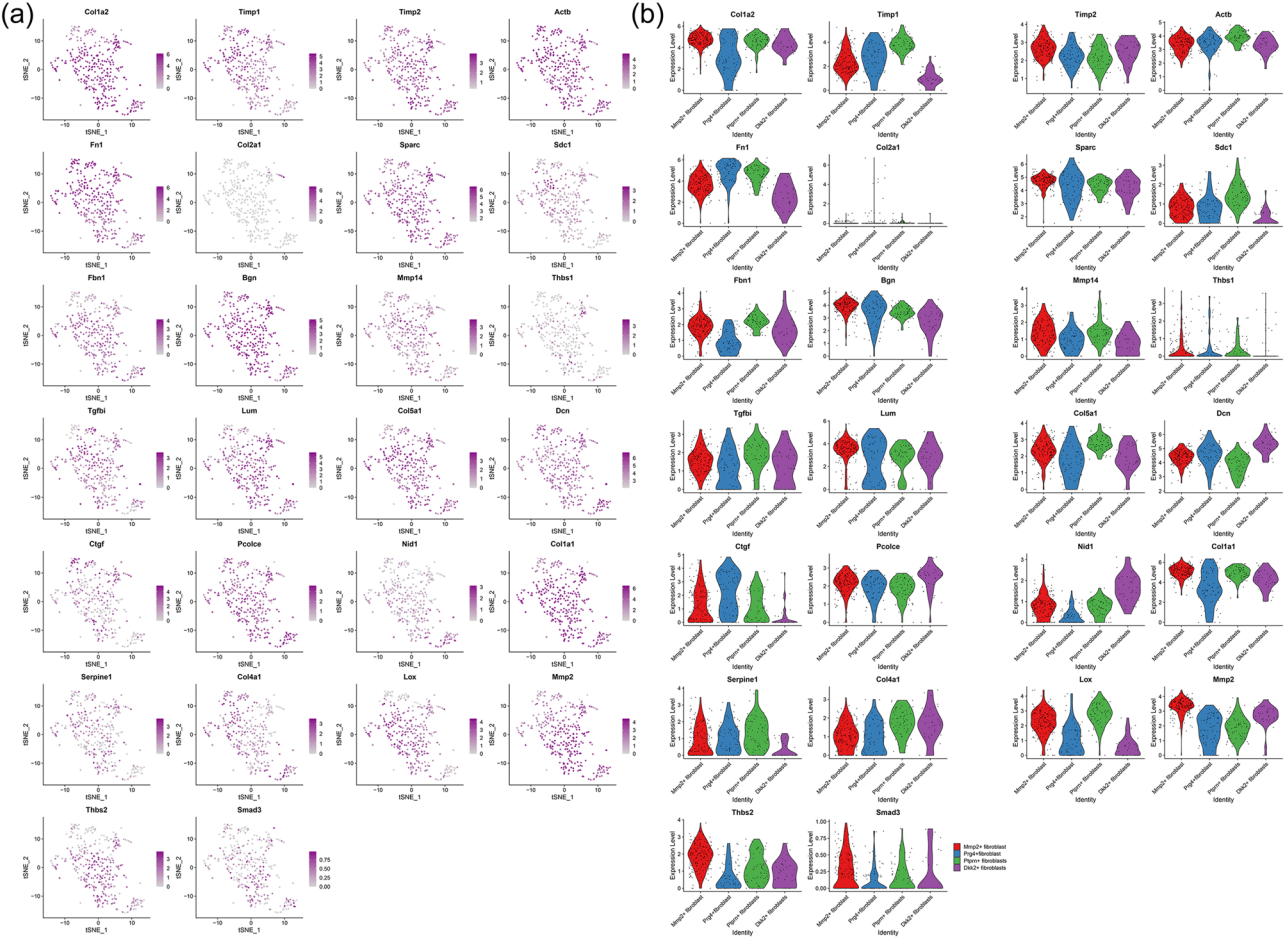
Distribution of proteomics cluster 1 in fibroblasts (a: distribution of genes; b: average expression of genes).

It was found that the genes in these subclusters had obvious distribution differences, for example, Col1a2 and Timp1 was mainly distributed in Fibroblasts cells and so on (Fig. 27). In Fibroblasts cells, Tnbs1 is mainly distributed in Mmp2+fibroblast, Prg4+fibroblast and Ptprn+fibroblast, etc. (Fig. 28). See "Distribution of other clusters in synovial cells and fibroblasts" for cluster 2 and cluster 3 results.

FLS play a significant role in the pathogenesis of rheumatoid arthritis (RA), and they are highly specialized mesenchymal cells found in the synovial membrane of the hip joint ^65^. The synovium is composed of two layers: the intimal lining layer and the sublining layer, with FLS mainly located in the intimal lining layer. In healthy joints, the intimal lining forms a thin, porous barrier between the lining and synovial fluid ^66^. FLS regulate the composition of the extracellular matrix (ECM) and synovial fluid, thus lubricating and nourishing the surface of the cartilage ^67^. In RA, FLS exhibit unique invasive behavior and actively contribute to the pathogenesis and progression of the disease. FLS in RA can not only be activated by inflammation but also function as drivers of inflammatory activation and aggressive phenotypes ^68^. A study analyzing single-cell RNA sequencing of RA synovium identified two major fibroblast phenotypes: lining CD55+population and sublining CD90+population. CD55+fibroblasts were enriched with genes such as HAS1, which encodes hyaluronan synthase, and genes related to endothelial cell proliferation and reactive oxygen species regulation. CD90+fibroblasts were enriched with genes associated with MMP expression and ECM organization ^69^. Integration of RNA sequencing (RNA-seq) data with histopathological data revealed at least three possible synovial patterns in untreated RA patients: a fibroblast pattern lacking immune cell infiltration, a myeloid pattern characterized by enrichment of macrophages or monocytes, and a lymphoid pattern characterized by clustering of B cells and T cells ^70^.

FLS derived from RA patients exhibit autonomous pathogenic features, which can be preserved even after several months of tissue culture or implantation in mice ^71^. In rheumatoid joints, FLS numbers are significantly increased and contribute to the transition of the synovial lining from a delicate structure to an infiltrative proliferative tissue mass known as pannus ^72^. RA FLS proliferate in culture when exposed to platelet-derived growth factor, transforming growth factor-β, TNF, or IL-1β, all of which are produced by immune cells present in inflamed joints ^73^. Furthermore, compared to other cell types, RA FLS demonstrate resistance to endoplasmic reticulum stress-induced apoptosis, possibly due to increased autophagy and proteasome activity ^74^. Evidence suggests that FLS in RA may undergo epithelial-to-mesenchymal transition, an essential developmental process during complex tissue formation that is believed to occur in adult tissues following an epithelial stress ^75,76^. Another source of increased FLS is the recruitment and subsequent differentiation of multipotent mesenchymal stem cells (MSC) from the bone marrow to the synovium ^77^. In a collagen-induced arthritis mouse model, the influx of these blood-derived mesenchymal precursors precedes inflammation, indicating that their massive influx promotes the onset of arthritis ^56^. At the interface between RA synovium and cartilage, FLS-mediated overproduction of matrix metalloproteinases (MMPs) such as MMP1, MMP3, and MMP13 disrupts the collagen-rich structure of joint tissues and facilitates FLS invasion ^57^. Elevated expression of MMPs has been observed in the intimal lining layer of synovial tissue samples from newly diagnosed RA patients (≤ 1 week after symptom onset) ^78^. The extent of FLS expansion in RA is positively correlated with disease duration, the number of macrophage infiltrations in the synovium, and the severity of cartilage erosion ^79^. Studies in mice also demonstrate the essential role of FLS activation, as they are indispensable in certain models such as human TNF transgenic mice and mice induced with collagen antibody-induced arthritis ^80^. In RA, FLS exhibit crucial immunoregulatory functions, including the secretion of IL-6. These cells actively promote the influx, proliferation, survival of immune cells, and joint neovascularization by producing cytokines, chemokines, and angiogenic factors ^81^. FLS in RA prolong the lifespan of B cells by generating IL-6, vascular cell adhesion molecule 1 (VCAM1), CXC chemokine ligand 12 (CXCL12), B cell-activating factor (BAFF; also known as TNFSF13B), and a proliferation-inducing ligand (APRIL; also known as TNFSF13) ^82^. Additionally, FLS in RA can act as antigen-presenting cells for T cells ^83^. They attract monocytes from the vascular system through the secretion of chemokines, such as chemokine ligand 2 (CCL2; also known as MCP1), CCL5, CCL8, CXCL5, and CXCL10. FLS in RA also modulate the influx of inflammatory infiltrates through crosstalk with adjacent vascular endothelial cells ^84,85^.

### Network pharmacology core target validation

#### Distribution of top genes of Celastrol-RA target PPI network in PBMC

**Figure 29:**
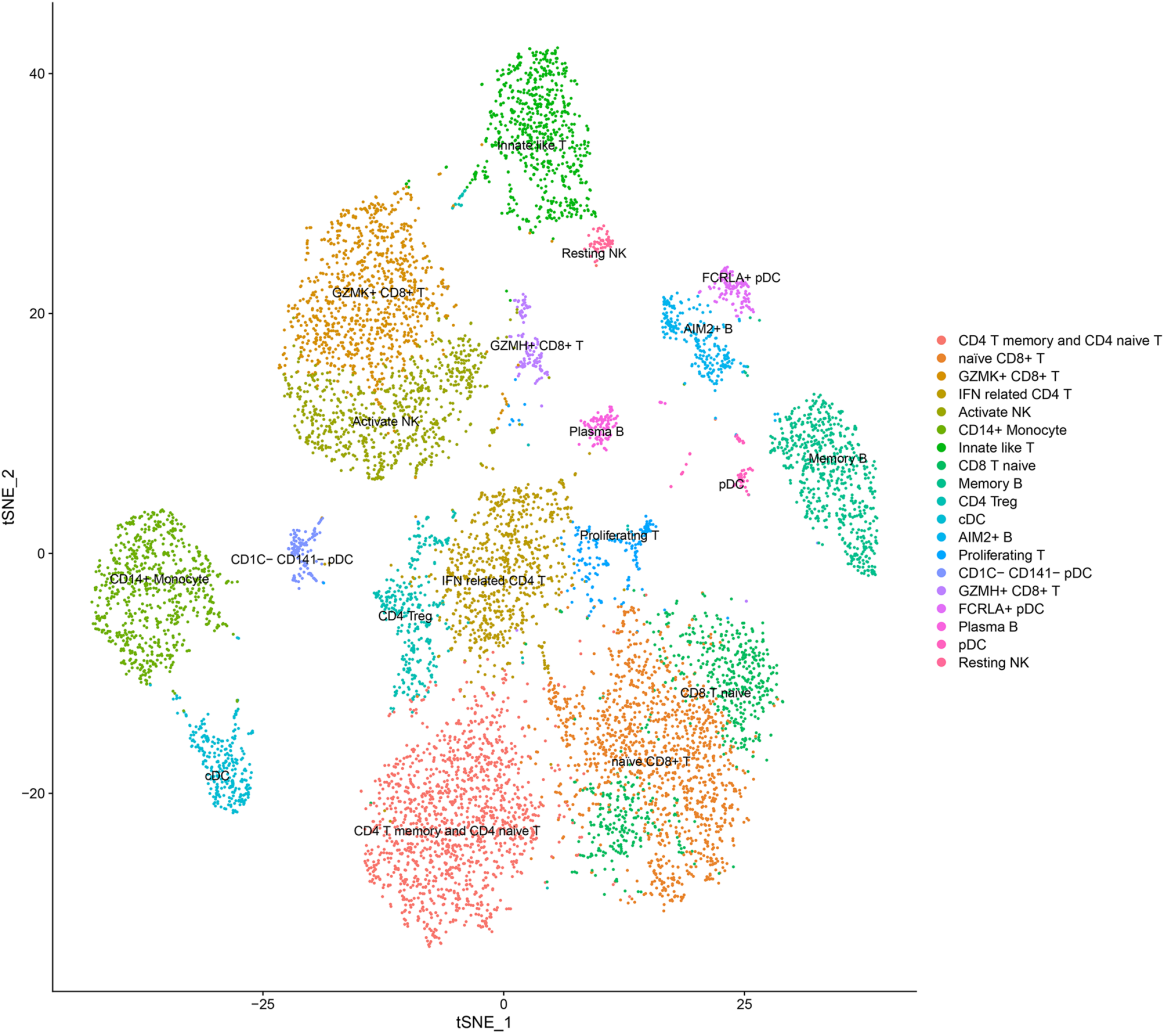
Identified cell clusters of synovial cells.

**Figure 30:**
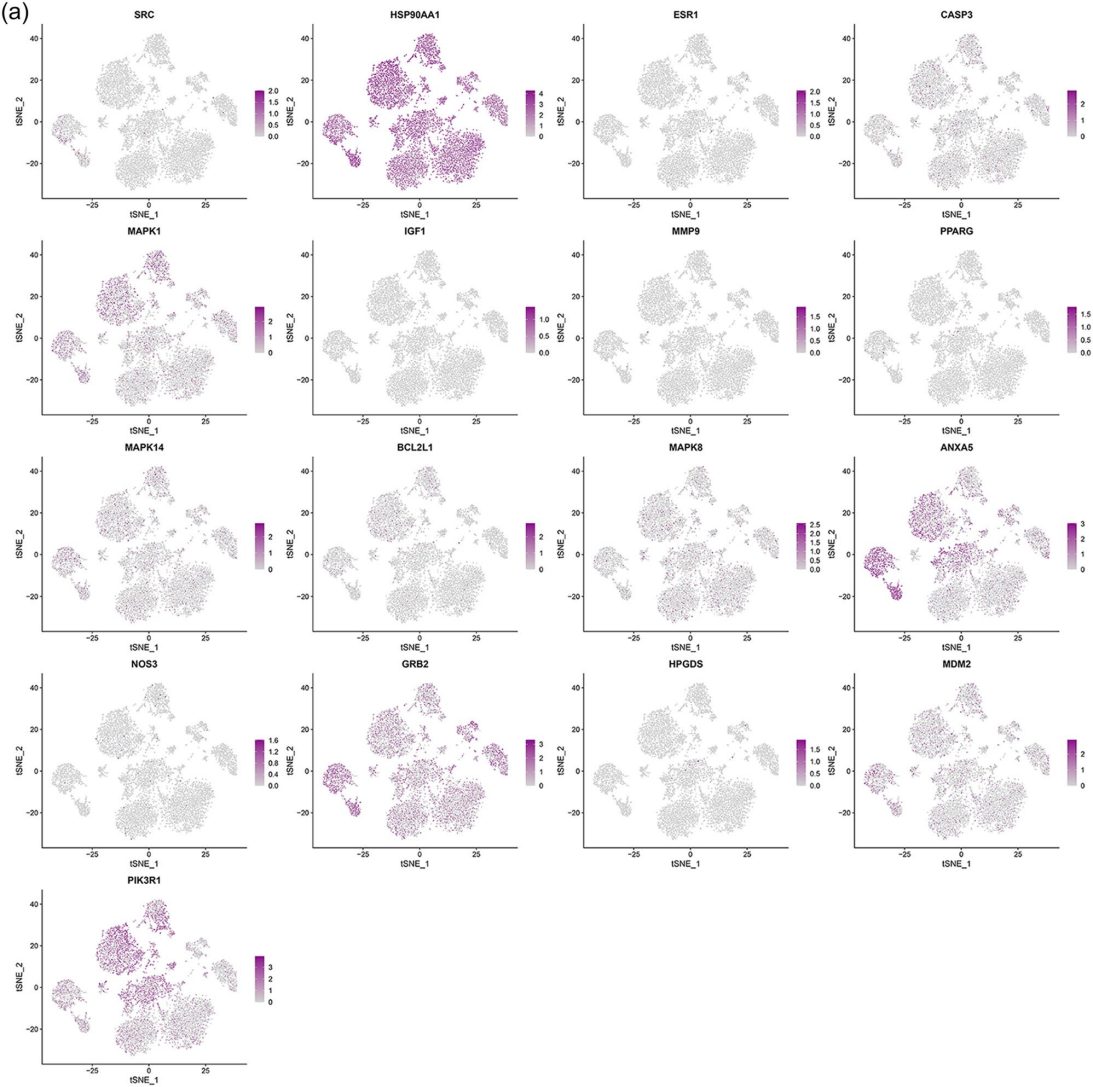

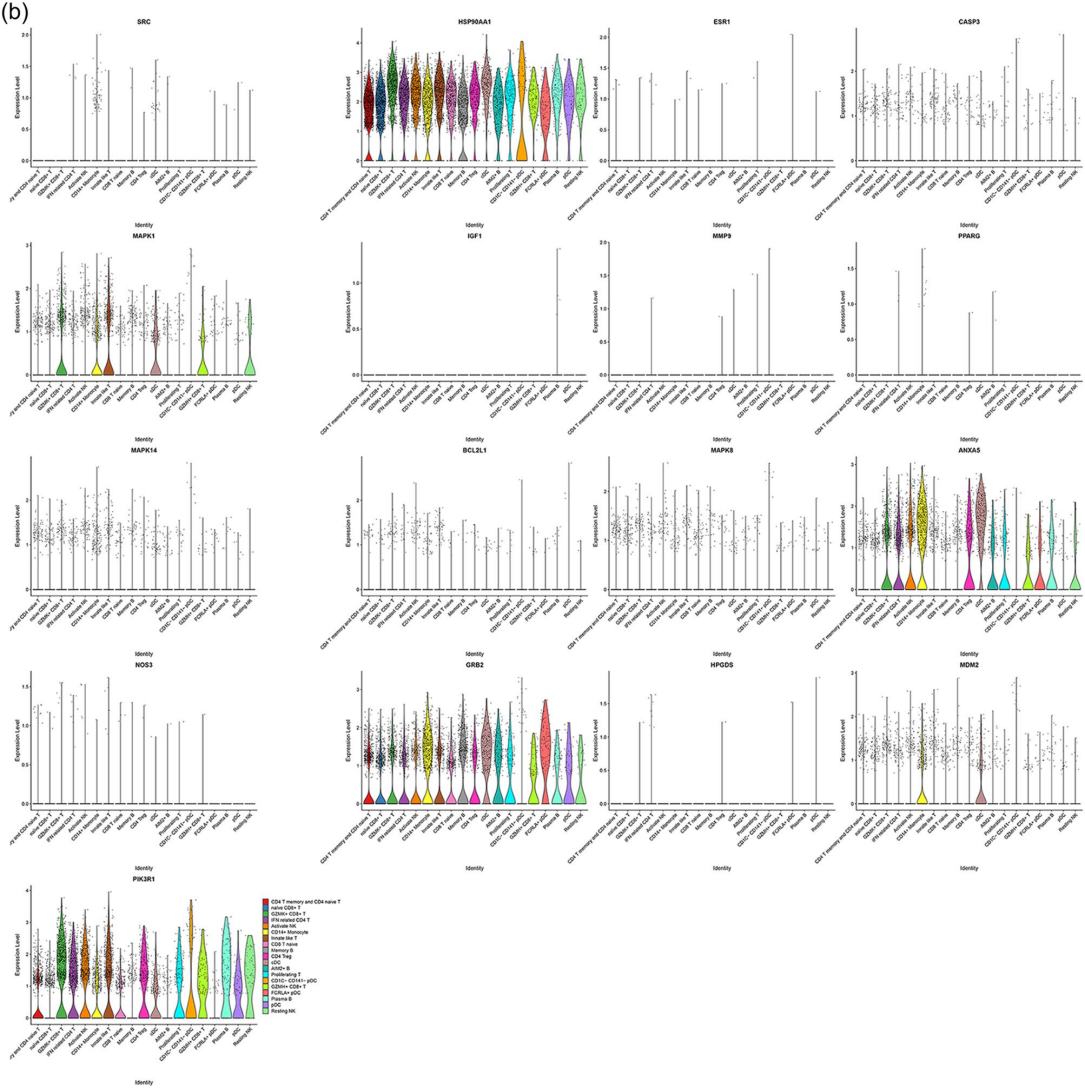
Distribution of top 20 genes of Celastrol-RA target PPI network in PBMC (a: distribution of genes; b: average expression of genes).

The single-cell sequencing data GSE159117 was processed in the same way. This data set is the PBMC data of RA patients, and its grouping is shown in Fig. 29. After mapping, it can be found that HSP90AA1 exists in all cells. MAPK1 is mainly present in GZMK+CD8+T, CD14+monocyte, Innate like T, cDC, GZMH+CD8+T, Resting NK cells. ANXA5 is mainly present in GZMK+CD8+T, IFN related CD4 T, Activate NK, CD14+monocyte, CD4 Treg, cDC, AIM2+B, proliferating T, GZMH+CD8+T, FCRLA+pDC, Plasma B, Resting NK cells. GRB2 was more distributed in cells other than CD1C-CD141-pDC. MDM2 is mainly distributed in CD14+monocyte and cDC cells. PIK3R1 was more distributed in cells other than Memory B, AIM2+B and FCRLA+pDC (Fig. 30).

#### Molecular docking results

**Figure 31:**
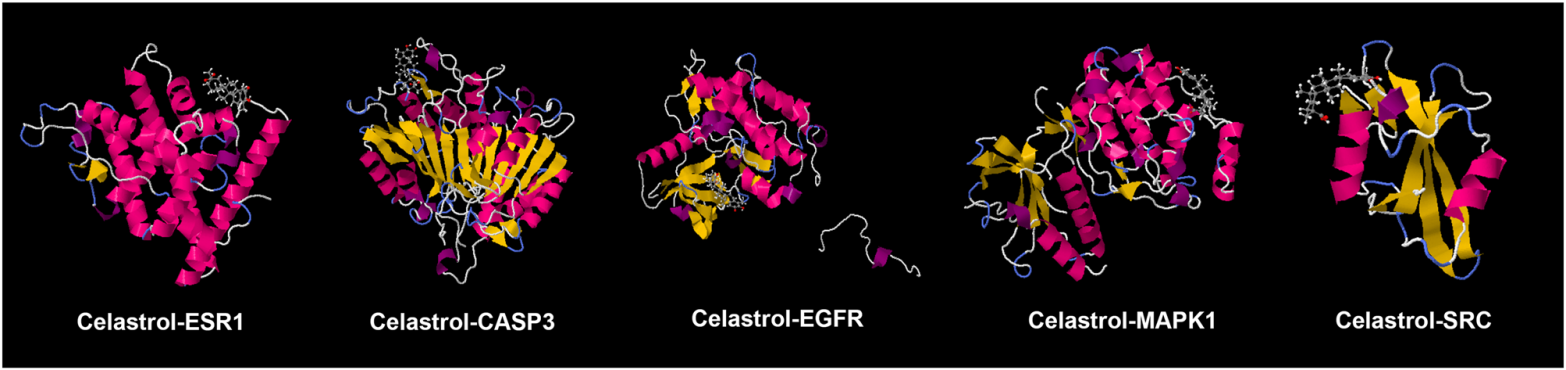
Molecular Docking Results.

The results of molecular docking showed that the binding energy of Celastrol and SCR is -6.39 kcal/mol; that the binding energy of Celastrol and EGFR is -7.86 kcal/mol; that the binding energy of Celastrol and ESR1 is -6.24 kcal/mol; that the binding energy of Celastrol and CASP3 is -7.58 kcal/mol; that the binding energy of Celastrol and MAPK1 is -6.07 kcal/mol (Fig. 31). This suggests that Celastrol can stably combine with these molecules.

## Discussion

Systems pharmacology starts from the integrity and systematicness of the interaction between drug targets and diseases, and studies the biological basis of the whole drug action on the basis of the multi-level network of disease-gene-drug. It provides a new idea for the study of pharmacological effects and mechanisms of natural compounds, and has become a powerful tool for the modernization of traditional Chinese medicine ^86^. In this study, the Celastrol-RA target PPI network was constructed by systematic pharmacology method, and TNF, EGFR, HSP90AA1, CASP3, ESR1, IGF1, MAPK1, MMP9, PPARG, MAPK14, BCL2L1, MAPK8, ANXA5, GRB2, NOS3 were predicted. HPGDS, STAT1, PIK3R1, MMP2, MDM2, PPARA, MAP2K1, IL2, PTPN11, JAK2, IGF1R, KDR, GSK3B, ARIL6 and MAPK14 are potential key targets for Celastrol in the treatment of RA. A number of current studies have shown that the above key genes mainly mediate multiple stages of pathological processes in the development and pathogenesis of RA ^65,87–89^. The inflammatory targets that mediate rheumatoid arthritis mainly include (TNF, PTPN11, IL6, etc.); the main targets that mediate RA oxidative stress are NOS3; the targets that mediate the destruction of extracellular matrix tissue in RA mainly include (MMP9, MMP2, ANXA5, etc.). In particular, MMP9, which is an important predictor of RA disease activity, is the most important protease leading to cartilage degradation, is a systemic inflammatory marker, and can be used as an indicator of synovial damage and prognosis in RA patients ^90^. In addition, the main targets of immune and inflammatory factors that mediate RA are (IL2, JAK2, STAT1); The main genes that mediate the excessive activation of RA immune cells, the inflammatory hyperplasia of synovial tissue and the destruction of articular cartilage tissue are (MAPK1, MAPK14, MAPK8, MAP2K1, etc.) ^91,92^.

The pathway enrichment analysis results of Celastrol-RA targets showed that the pathways involved in the treatment of RA by Celastrol include (hsa04151) PI3K-Akt signaling pathway, (hsa04659) Th17 cell differentiation, (hsa04010) MAPK signaling pathway; hsa04068) FoxO signaling pathway, (hsa01521) Epidermal growth factor receptor tyrosine kinase inhibitor resistance, (hsa04657) IL-17 signaling pathway, (hsa04660) T cell receptor signaling pathway, (hsa04668) TNF signaling pathway; hsa04658) Th1 and Th2 cell differentiation, (hsa04370) vascular endothelial growth factor signaling pathway, (hsa04062) Chemokine signaling pathway, (hsa04613) Neutrophil extracellular trap formation, (hsa04150) mTOR signaling pathway, (hsa04620) Toll-like receptor signaling pathway, (hsa04621) NOD-like receptor signaling pathway, (hsa04630) JAK-STAT signaling pathway. Among them, PI3K-Akt signaling pathway is an important pathway of apoptosis. Abnormal activation of PI3K-Akt pathway can lead to decreased apoptosis of synovial fibroblasts and promote synovial hyperplasia, synovial inflammation, bone and subchondral bone destruction ^93,94^. For example, when studying the effect of the inflammatory factor interleukin-22 (IL-22) on fibroblast-like synoviocytes (FLS), it was found that IL-22 was dependent on the PI3K/Akt pathway to induce significant proliferation of FLS ^95^. IL-17 can be detected in pathological biopsies of RA patients. IL-17 stimulates RA-FLS to secrete IL-6, IL-8 and vascular endothelial growth factor receptor (VEGFR) through PI3K/Akt and NF-κB pathways ^96^. In addition, IL-21 further promotes the inflammatory proliferation of RA-FLS by inducing and triggering PI3K. Moreover, the expression level of PI3K/Akt signal in FLS was significantly increased under IL-21 stimulation, and the Akt level was also higher than the threshold. This shows that IL-21 has the ability to activate PI3K/Akt signaling, and use this as a link to intervene in the development of RA ^97,98^. Wu et al. found that blocking the PI3K/Akt pathway can significantly inhibit the expression of TNF-α-induced B lymphocyte-induced maturation protein 1 (Blimp 1), suggesting that the PI3K/Akt pathway is involved in the regulation of TNF-α-induced Blimp 1 expression ^99^.

IL-17A is a pro-inflammatory cytokine and a regulator of inflammatory response, which directly or indirectly promotes and aggravates joint inflammation through a series of reactions ^100^. IL-17A also acts on a variety of cells (such as Th17 cells, macrophages, dendritic cells, fibroblasts, endothelial cells, epithelial cells, keratinocytes and lymphocytes). The release of pro-inflammatory cytokines TNF-a, IL-1, IL-6, G-CSF and granulocyte–macrophage colony stimulating factor (GM-CSFI), C-X-C motif chemokine ligand 1 (CXCL1), CXCL5, IL-8, CCL2 and CCL7, antimicrobial peptides (defensins and S100 proteins) are involved in acute inflammatory response ^101–103^. Under the action of IL-17A, endothelial cells can also release tissue factor and promote thrombus formation ^104^. In the late stage of inflammation, IL-17A causes chronic inflammation by prolonging the survival time of immune cells in RA-FLS and germinal centers, induces synovial hyperplasia, promotes the formation of osteoclasts and collagen degradation and cartilage destruction, causes local bone damage and promotes joint destruction ^105,106^. At present, IL-17-related inhibitors are constantly emerging in new clinical drugs for RA. Common IL-17 inhibitors include secukinumab, ixekizumab, brodalumab, and CNTO6785 ^50,107^. In RA, p38 MAPK plays a key role as it can regulate the production of pathogenic cytokines such as IL-1 and TNF-α through various transcriptional and translational mechanisms ^108^. In the synovial tissue of RA, p38 is highly expressed and activated, and the commonly used p38 MAPK inhibitor SB203580 reduces the production of pro-inflammatory cytokines by monocytes/macrophages, neutrophils and T lymphocytes ^109^. In addition, in a rodent model of RA, p38 MAPK inhibitors can suppress inflammation and bone destruction ^110^.

TNF signaling pathway is the classic cytokine pathway of RA. TNF is involved in the functional regulation of RA-related synovial cells, chondrocytes, osteoclasts, osteoblasts and other cells. It accelerates the progress of RA by stimulating the production of inflammatory cytokines, synovial proliferation, pannus hyperplasia, bone and cartilage destruction, which is an important target pathway for RA treatment ^111,112^. Toll-like receptor signaling pathway plays an important role in both innate immunity and acquired immunity. The activation of Toll-like receptor signaling pathway can achieve immune regulation function, and use myeloid differentiation factor-dependent and non-dependent ways to activate NF-κB or MAPK signaling pathway, activate downstream transcription factors, and then up-regulate the expression of inflammatory cytokines such as IL-1β, IL-6 and TNF-α, and mediate inflammation ^113,114^. The inflammatory chemokine signaling pathway plays an important role in the pathogenesis of RA, and inhibiting the inflammatory chemokine signaling pathway can effectively treat RA ^115^. TNF is expressed on the surface of monocytes, T cells, B cells, natural killer (NK) cells, synovial fibroblasts and other cells. It can mediate the activation, adhesion and migration of leukocytes, promote the activation of endothelial cells and angiogenesis, and is also related to the expression of chemokines and the activation of osteoclasts ^10,116^. The effect of IL-6 on the joint synovium is similar to that of TNF, and it directly drives the increase of acute phase reactants ^117^. NOD-like receptor signaling pathway, T cell receptor signaling pathway and B cell receptor signaling pathway are all important signaling pathways for immune regulation ^118^. Dysfunctional JAK-STAT signaling pathway can lead to excessive proliferation of RA-FLS, thereby promoting synovitis, cartilage degradation and bone destruction. Pathogenic factors such as IL-1, IL-6, IL-10 and interferon (IFN) activate the JAK-STAT signaling pathway through different ways, thus playing a biological role in the progression of RA disease ^119,120^.

In summary, this study found that Celastrol mainly by regulating immune inflammatory cells, inhibiting immune inflammatory chemokines, inhibiting immune inflammatory chemokine signaling pathways, inhibiting osteoclast differentiation, promoting synovial cell apoptosis and other ways to intervene in the pathological process of RA.

Subsequently, we analyzed the transcriptomic results of Celastrol treatment of RA, and found that the down-regulated genes mainly included TNF, FOS, ITGAM, CXCR4, HSP90AA1, PAX6, SPI1, PTGS2, ASCL1, GAD1, KCNA1, FOXG1, IRF4, LCK, EGR2, EOMES, SPP1, CCL3, BMP2, FLT3 and other targets. The signaling pathways include neuroactive ligand-receptor interactions, MAPK signaling pathway, cytokine-cytokine receptor interaction, systemic lupus erythematosus, calcium signaling pathway, neutrophil extracellular trap formation, cytokine and cytokine receptor interaction, Rap1 signaling pathway, PI3K- Akt signaling pathway and other signaling pathways. In summary, combined with the results of transcriptomics, it can be found that Celastrol can regulate inflammatory factors and their mediated signaling pathways. The results of proteomics showed that the core proteins regulated by Celastrol include ACBB, FN1, HSP90AA1, HSPA8, MMP2, COL4A1, CTGF, THBS1, APP, HSPA9, TIMP1, LOX, RPS27A, HSPA1A, FBN1, BGN, SPARC, SERPINE1, MMP14, LUM. The results of enrichment analysis showed that biological processes include protein folding, response to topologically incorrect protein, response to unfolded protein, extracellular matrix organization, extracellular structure organization, etc. The signaling pathways include ECM-receptor interaction, Human papillomavirus infection, Focal adhesion, PI3K-Akt signaling pathway, etc. The Reactome pathway include Extracellular matrix organization, Regulation of IGF transport and uptake by IGFBPs, ECM proteoglycans, Post-translational protein phosphorylation, Cellular response to heat stress, etc. This indicates that Celastrol regulates RA-FLS synovial proteomics mainly in extracellular matrix organization, TGF-β signaling pathway, regulation of IGF transport and uptake by IGFBPs, ECM proteoglycan and other mechanisms to regulate TGF-β pathway.

Subsequently, this study further analyzed the distribution of key targets in the synoviocytes of Celastrol-RA PPI network clusters in RA patients’ synoviocytes through single-cell transcriptomics to determine the main cells regulated by Celastrol. The results showed that Anxa5 was mainly distributed in Hematopoietic cells and Fibroblasts cells; Mmp9 was mainly distributed in Hematopoietic cells and so on; In Fibroblasts cells, Met is mainly distributed in Prg4+fibroblast, Mmp9 is mainly distributed in Ptprn+fibroblast, etc. The results of gene mapping of transcriptomic clusters showed that Lmnb1 was mainly distributed in Hematopoietic cells and so on; In Fibroblasts cells, Spc24 is mainly distributed in Ptprn+fibroblast, etc. The results of gene mapping of proteomic clusters showed that Col1a2 and Timp1 was mainly distributed in Fibroblasts cells and so on; In Fibroblasts cells, Tnbs1 is mainly distributed in Mmp2+fibroblast, Prg4+fibroblast and Ptprn+fibroblast, etc. These suggest that future research can focus on observing the intervention of Celastrol on the above cells.

**Figure 32:**
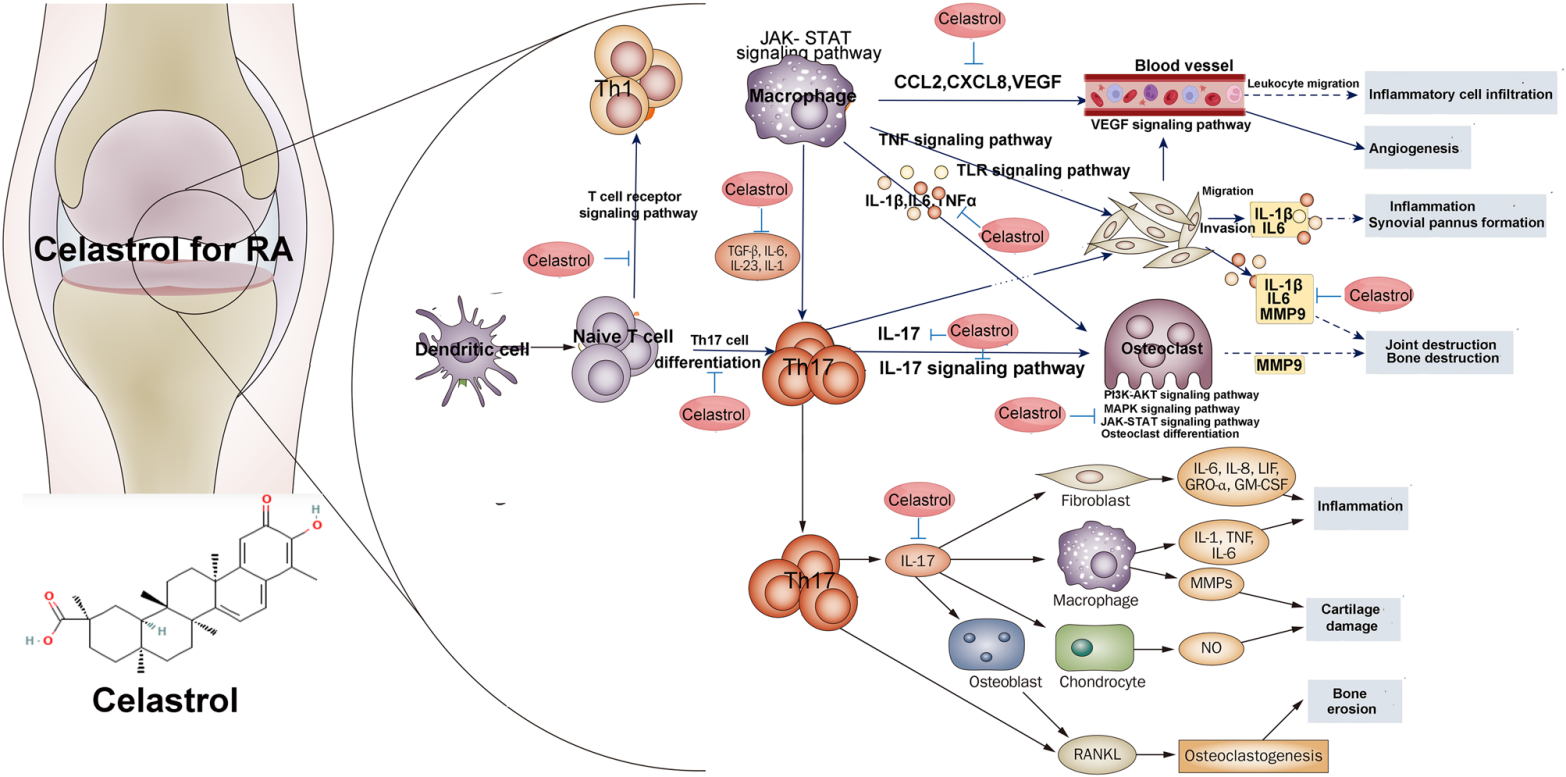
The mechanism of celastrol in the treatment of RA (*Th* T helper cell, *IL* Interleukin, *VRGF* vascular endothelial growth factor, *CCL2* chemokine ligand 2).

The mechanism of celastrol in treating RA was summarized in Fig. 32.

## Materials and methods

### Celastrol targets prediction and RA genes collection

The database used in this study is public. The structure of Celastrol was searched from Pubchem (https://pubchem.ncbi.nlm.nih.gov/) and saved as “sdf” format file. The “sdf” file were then input into Pharmmapper to predict the potential targets of Celastrol ^121^. In the GeneCards database (https://www.genecards.org/) ^122^, OMIM database (https://omim.org/) ^123^, DisGeNet database (https://www.disgenet.org/) ^124^, and DrugBank database (http://www.drugbank.ca) ^125^, “Rheumatoid Arthritis” was used as the key word to search for RA genes, and the results were combined and deduplicated to obtain the RA-related gene set. The Celastrol target set and RA-related gene set were intersected, and the targets in the intersection set were considered as the target of Celastrol in the treatment of RA (that is Celastrol-RA targets).

### Transcriptomics and Proteomics data collection and processing

The transcriptomics and proteomics data from fibroblasts of Celastrol in the treatment of RA were from reference ^126^. The screening criteria for differentially expressed genes and differentially expressed proteins were Log2FC ≥ 1 or ≤  − 1, and FDR < 0.05.

### Single cell sequencing data collection and processing

Single-cell sequencing data for synovial fibroblasts were obtained from the GEO database (GSE192504 and GSE159117). The dataset included synovial cells isolated from collagen-induced arthritis (CIA) mice. The Seurat 4.0 package in R software was used to normalize single-cell data and remove low-quality cells in the data set ^127^. Then, the "FindVariableFeatures" function was used to find highly variable genes (HVGs) for subsequent analysis. Afterwards, the data is centrally processed using the "ScaleData" function, and a linear transformation is calculated to ensure that individuals in the sample are given the same weight in the downstream principal component (PCA) analysis. After PCA analysis, the "tSNE" function is used to map the data in the high-dimensional space to the low-dimensional space while preserving the local features of the dataset. Finally, the "FindAllMarkers" function was used to detect markers of all cell populations and labeled all cell populations according to the CellMarker database (http://biocc.hrbmu.edu.cn/CellMarker/).

### Network construction and analysis

Celastrol-RA targets were input into STRING (https://string-db.org/), the species was limited to “Homo sapiens” or “Mus musculus”, and the minimum interaction threshold was set to “medium confidence” (≥ 0.4) ^128^. The result file was imported into Cytoscape 3.9.1 software to construct a protein–protein interaction (PPI) network and visualize the results ^129^. Then Celastrol-RA targets were imported into the Metascape database (https://metascape.org/gp/index.html) ^130^. “Input as species” and “Analysis as species” were set to Homo sapiens or Mus musculus, and the threshold was set to *P* < 0.05 for gene ontology (GO) and Kyoto encyelopedia of genes and genomes (KEGG) enrichment analysis., so as to analyze the possible mechanism of Celastrol in the treatment of RA.

## Conclusion

In this study, multi-omics strategies such as system pharmacology, transcriptomics, proteomics, and single-cell transcriptomics were used to reveal that Celastrol may regulate PI3K/AKT signaling pathway, Th17 cell differentiation, MAPK signaling pathway, TNF signaling pathway, Toll signaling pathway, TGF-β/SMAD signaling pathway, IL-17 signaling pathway, JAK-STAT signaling pathway and other major signaling pathways by acting on key targets such as TNF and IL6 to exert immunomodulatory functions. Inhibiting inflammatory response, inhibiting RA-FLS proliferation, migration and osteoclast differentiation, promoting apoptosis and other ways to show anti-RA effect. This reveals the characteristics and advantages of Celastrol in anti-RA through multi-component, multi-target and multi-pathway synergy, and provides a basis for further experimental research and clinical application.

### Supplementary Information

Supplementary Figures.

Supplementary Table S1.

Supplementary Table S2.

Supplementary Table S3.

## Data Availability

The data used to support the findings of this study are included within the article and the supplementary information files. The database used in our study is public.
