# Supplementary material for: Exploring the mechanism of Celastrol in the treatment of rheumatoid arthritis based on systems pharmacology and multi-omics
Source: Sci Rep. 2024 Jan 18;14:1604. doi: 10.1038/s41598-023-48248-5 (PMC10796403; doi:10.1038/s41598-023-48248-5)
Supplement: Supplementary file 1 — Supplementary Figures. [file 41598_2023_48248_MOESM1_ESM.docx]

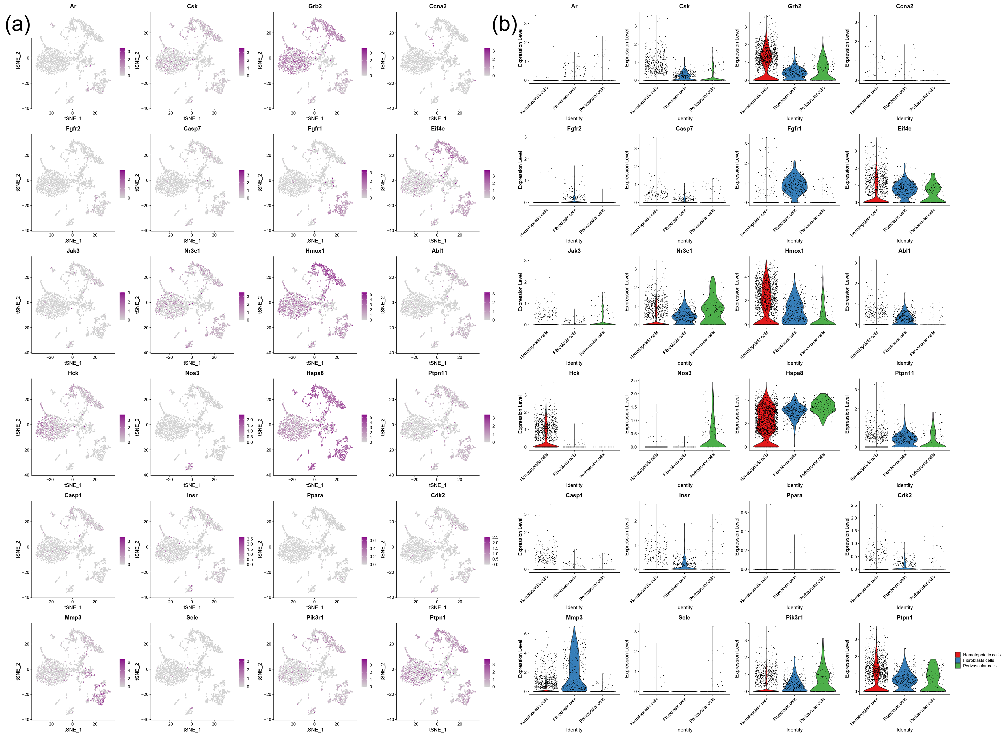


Figure S1 Distribution of network pharmacology cluster 2 in synovial cells (a: distribution of genes; b: average expression of genes)


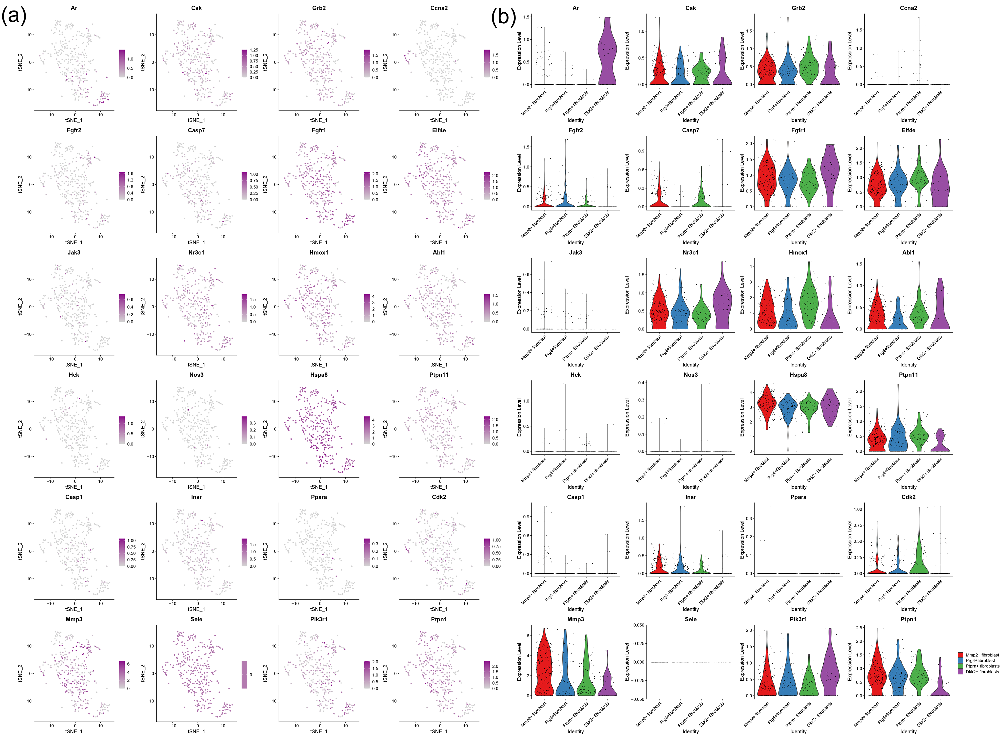


Figure S2 Distribution of network pharmacology cluster 2 in fibroblasts (a: distribution of genes; b: average expression of genes)


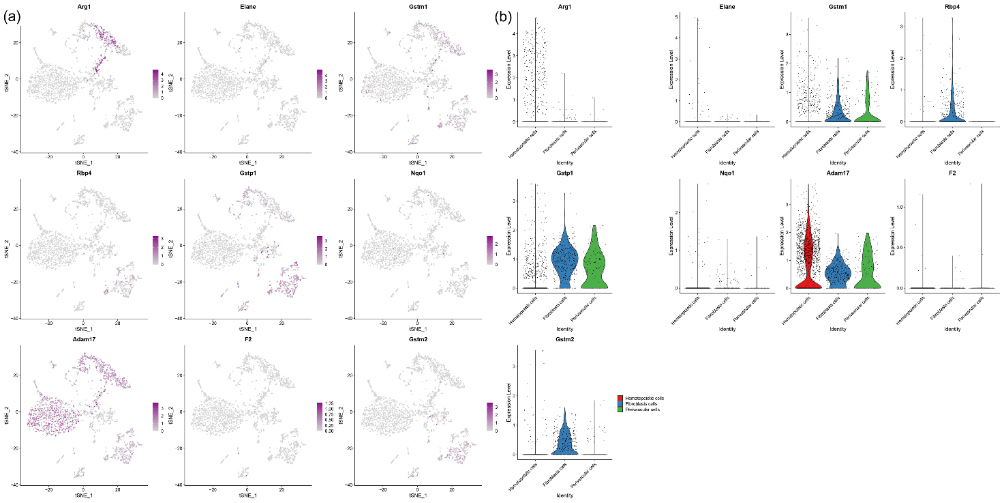


Figure S3 Distribution of network pharmacology cluster 3 in synovial cells (a: distribution of genes; b: average expression of genes)


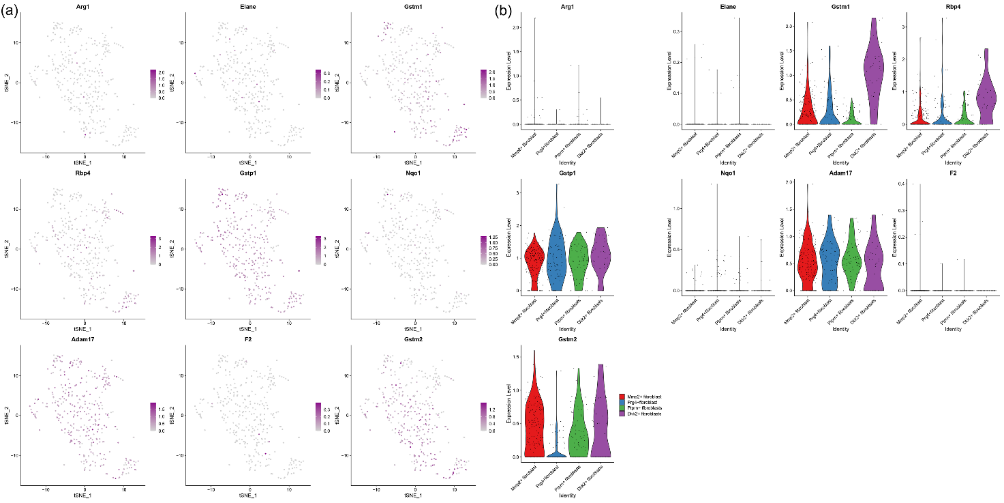


Figure S4 Distribution of network pharmacology cluster 3 in fibroblasts (a: distribution of genes; b: average expression of genes)


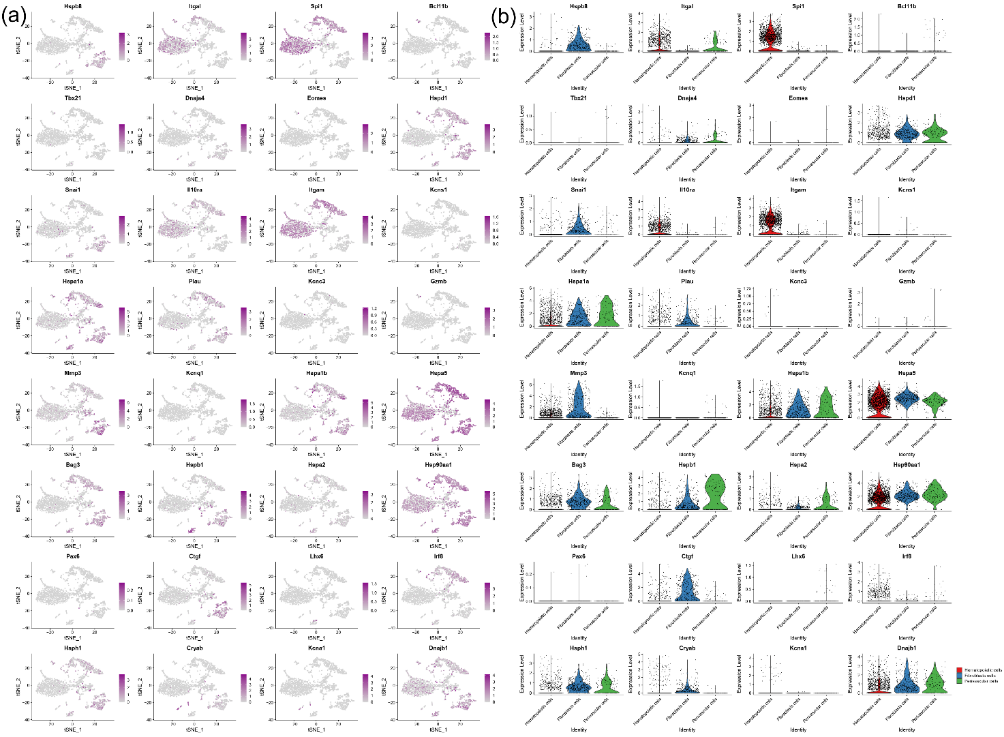


Figure S5 Distribution of transcriptomic cluster 2 in synovial cells (a: distribution of genes; b: average expression of genes)


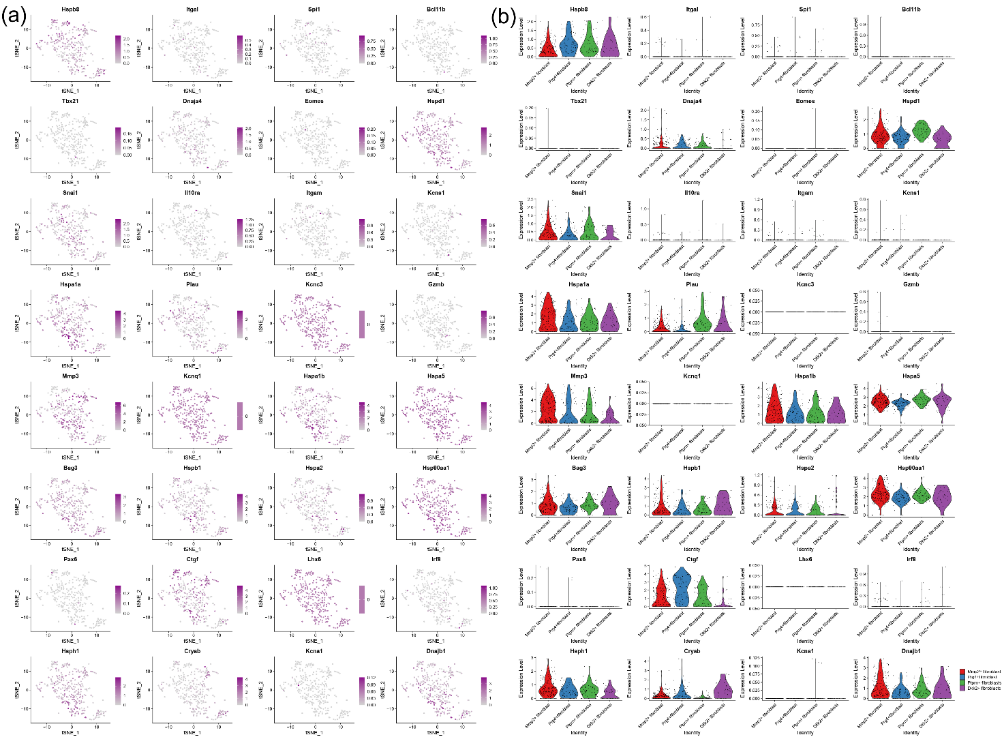


Figure S6 Distribution of transcriptomic cluster 2 in fibroblasts (a: distribution of genes; b: average expression of genes)


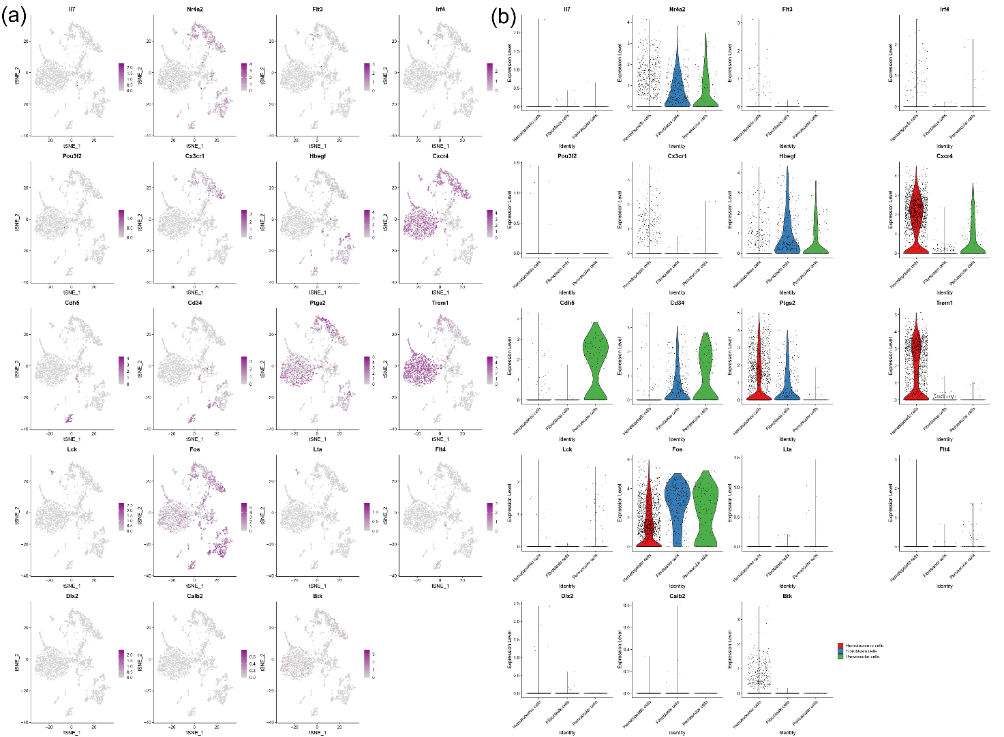


Figure S7 Distribution of transcriptomic cluster 3 in synovial cells (a: distribution of genes; b: average expression of genes)


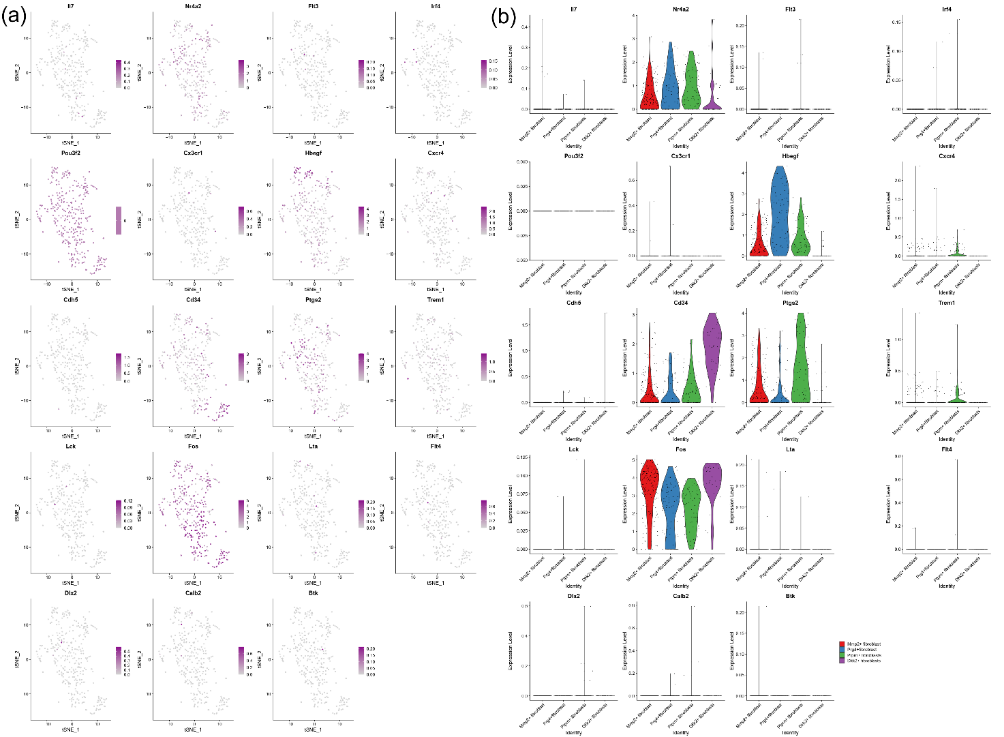


Figure S8 Distribution of transcriptomic cluster 3 in fibroblasts (a: distribution of genes; b: average expression of genes)


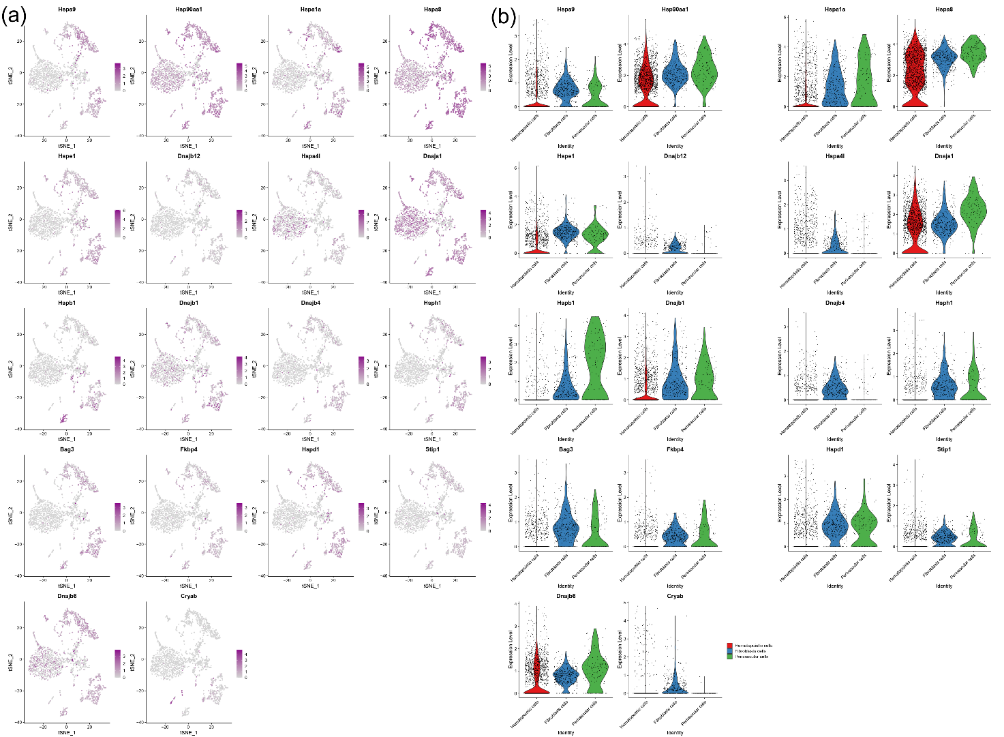


Figure S9 Distribution of proteomics cluster 2 in synovial cells (a: distribution of genes; b: average expression of genes)


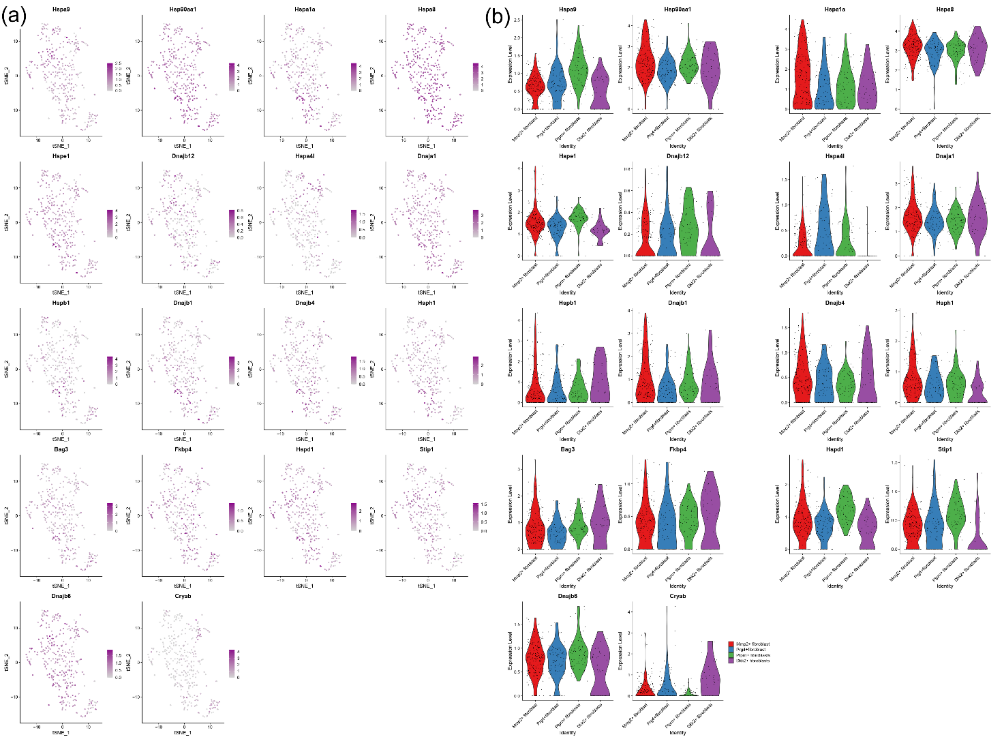


Figure S10 Distribution of proteomics cluster 2 in fibroblasts (a: distribution of genes; b: average expression of genes)


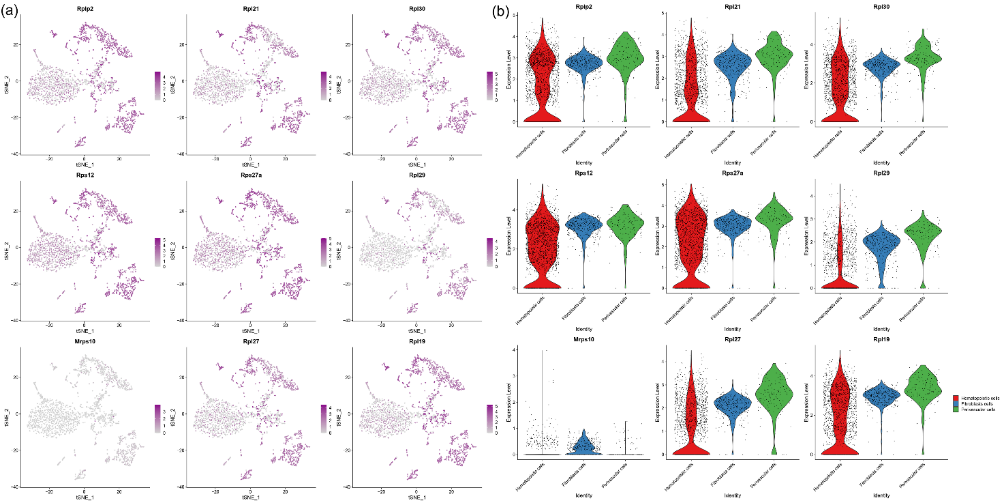


Figure S11 Distribution of proteomics cluster 3 in synovial cells (a: distribution of genes; b: average expression of genes)


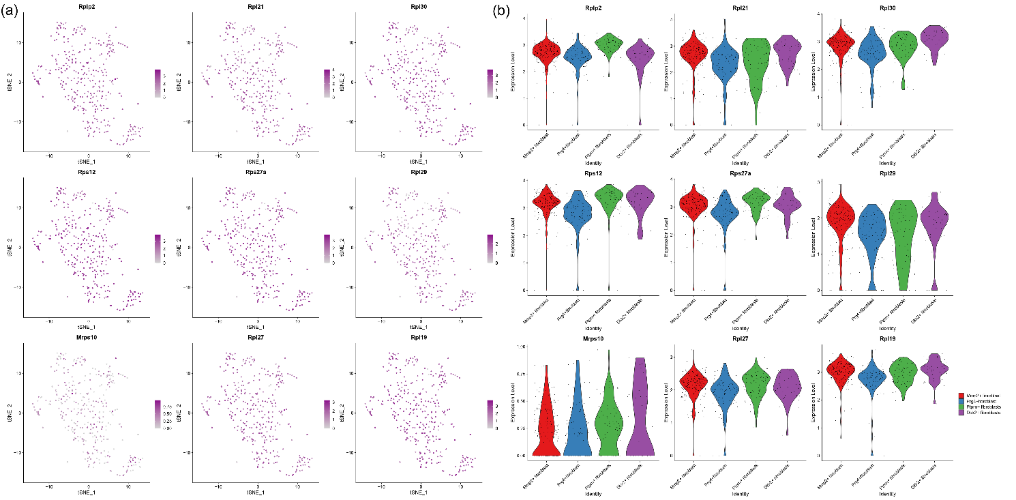


Figure S12 Distribution of proteomics cluster 3 in fibroblasts (a: distribution of genes; b: average expression of genes)
