# Supplementary material for: Exploring the mechanism of Celastrol in the treatment of rheumatoid arthritis based on systems pharmacology and multi-omics
Source: Sci Rep. 2024 Jan 18;14:1604. doi: 10.1038/s41598-023-48248-5 (PMC10796403; doi:10.1038/s41598-023-48248-5)
Supplement: Supplementary file 2 — Supplementary Table S1. [file 41598_2023_48248_MOESM2_ESM.docx]

Table S1 Enrichment analysis of triptolide-RA target PPI network

| Category | GO | Description | Pvalue | Enrichment | Z-score | Counts | Targets and Genes |
| --- | --- | --- | --- | --- | --- | --- | --- |
| GO Biological Processes | GO:0009725 | response to hormone | 1E-46 | 12 | 25 | 59 | PARP1\|AKT2\|APOA2\|AR\|ARG1\|BCHE\|CA2\|CALM3\|CASP3\|CCNA2\|MAPK14\|CSK\|CTSB\|CTSK\|CTSS\|ACE\|NQO1\|EIF4E\|ESR1\|ESR2\|ESRRA\|GCK\|GPI\|GRB2\|NR3C1\|GSK3B\|HMOX1\|HSPA8\|HSP90AA1\|IGF1R\|INSR\|JAK2\|JAK3\|KIT\|LTA4H\|NR3C2\|MMP2\|NOS2\|NOS3\|PDPK1\|PGF\|PGR\|PIK3R1\|PPARA\|PPARG\|PTPN1\|PTPN11\|RARA\|RARB\|RARG\|REN\|RXRA\|RXRB\|SRC\|STAT1\|TGFB2\|TYMS\|KAT2B\|NR1H3 |
|  | GO:0032870 | cellular response to hormone stimulus | 1E-35 | 14 | 23 | 43 | PARP1\|AKT2\|AR\|ARG1\|CA2\|CCNA2\|CSK\|CTSB\|CTSS\|ACE\|EIF4E\|ESR1\|ESR2\|ESRRA\|GCK\|GRB2\|NR3C1\|GSK3B\|HSPA8\|IGF1R\|INSR\|JAK2\|JAK3\|KIT\|NR3C2\|PDPK1\|PGF\|PGR\|PIK3R1\|PPARA\|PPARG\|PTPN1\|PTPN11\|RARA\|RARB\|RARG\|REN\|RXRA\|RXRB\|SRC\|STAT1\|KAT2B\|NR1H3 |
|  | GO:0006468 | protein phosphorylation | 1E-32 | 10 | 20 | 46 | ABL1\|AKT2\|BMP2\|BRAF\|BTK\|CCNT1\|CDK2\|CDK7\|MAPK14\|CSK\|CTSG\|EGFR\|EPHB4\|FGFR1\|FGFR2\|GSK3B\|HCK\|IGF1R\|IL2\|INSR\|JAK2\|JAK3\|KDR\|KIT\|LCK\|MET\|PDPK1\|PIM1\|PIK3CG\|PIK3R1\|PRKCQ\|MAPK1\|MAPK8\|MAPK10\|MAP2K1\|RARA\|MAPK12\|SRC\|AURKA\|SYK\|TEK\|TGFB2\|TGFBR1\|ZAP70\|CDK5R1\|MAPKAPK2 |
|  | GO:0007169 | transmembrane receptor protein tyrosine kinase signaling pathway | 1E-30 | 14 | 21 | 37 | ABL1\|AKT2\|AR\|BRAF\|CASP3\|MAPK14\|EGFR\|EPHB4\|FGFR1\|FGFR2\|GRB2\|GSK3B\|HCK\|IGF1\|IGF1R\|INSR\|JAK2\|JAK3\|KDR\|KIT\|LCK\|MET\|MMP2\|MMP9\|PDPK1\|PGF\|PIK3R1\|MAPK1\|PTPN1\|PTPN11\|SRC\|SYK\|ADAM17\|TEK\|ZAP70\|CDK5R1\|MAPKAPK2 |
|  | GO:0071396 | cellular response to lipid | 1E-30 | 12 | 20 | 40 | ABL1\|AR\|ARG1\|CASP1\|CCNA2\|MAPK14\|CTSG\|ACE\|EGFR\|EIF4E\|ESR1\|ESR2\|ESRRA\|FGFR2\|NR3C1\|GSK3B\|GSTP1\|HCK\|HSPA8\|IGF1R\|JAK2\|NR3C2\|MMP2\|NOS2\|NOS3\|PGR\|PIM1\|PPARA\|MAPK1\|MAPK8\|RARA\|RARG\|RORA\|RXRA\|RXRB\|SRC\|SYK\|VDR\|NR1H4\|NR1H3 |
|  | GO:0007167 | enzyme-linked receptor protein signaling pathway | 1E-29 | 10 | 19 | 42 | ABL1\|PARP1\|AKT2\|AR\|BMP2\|BRAF\|CASP3\|MAPK14\|EGFR\|EPHB4\|FGFR1\|FGFR2\|GRB2\|GSK3B\|HCK\|IGF1\|IGF1R\|INSR\|JAK2\|JAK3\|KDR\|KIT\|LCK\|MET\|MMP2\|MMP9\|PDPK1\|PGF\|PIK3R1\|PPARG\|MAPK1\|PTPN1\|PTPN11\|SRC\|SYK\|ADAM17\|TEK\|TGFB2\|TGFBR1\|ZAP70\|CDK5R1\|MAPKAPK2 |
|  | GO:0043549 | regulation of kinase activity | 1E-29 | 8.9 | 18 | 45 | ABL1\|BMP2\|CALM1\|CALM2\|CALM3\|CASP3\|CCNA2\|CCNT1\|CDK7\|CSK\|ACE\|EGFR\|ELANE\|EPHB4\|F2\|FABP4\|FGFR1\|FGFR2\|GSTP1\|HMGCR\|HSP90AA1\|HSP90AB1\|IGF1\|IGF1R\|IL2\|INSR\|JAK2\|KDR\|KIT\|MET\|PDPK1\|PIK3CG\|PIK3R1\|PPARG\|PPIA\|MAP2K1\|PTPN1\|SRC\|SYK\|ADAM17\|TEK\|TGFB2\|PDE5A\|KAT2B\|CDK5R1 |
|  | GO:0051347 | positive regulation of transferase activity | 1E-27 | 10 | 18 | 39 | ABL1\|APOA2\|BMP2\|CALM1\|CALM2\|CALM3\|CCNT1\|CSK\|ACE\|EGFR\|ELANE\|EPHB4\|F2\|FGFR1\|FGFR2\|HSP90AA1\|HSP90AB1\|IGF1\|IGF1R\|IL2\|INSR\|JAK2\|KDR\|KIT\|MET\|PDPK1\|PIK3CG\|PPIA\|PRKCQ\|MAPK1\|MAP2K1\|PTPN1\|SRC\|SYK\|ADAM17\|TEK\|TGFB2\|PDE5A\|CDK5R1 |
|  | GO:0033674 | positive regulation of kinase activity | 1E-27 | 11 | 19 | 36 | ABL1\|BMP2\|CALM1\|CALM2\|CALM3\|CCNT1\|CSK\|ACE\|EGFR\|ELANE\|EPHB4\|F2\|FGFR1\|FGFR2\|HSP90AA1\|HSP90AB1\|IGF1\|IGF1R\|IL2\|INSR\|JAK2\|KDR\|KIT\|MET\|PDPK1\|PIK3CG\|PPIA\|MAP2K1\|PTPN1\|SRC\|SYK\|ADAM17\|TEK\|TGFB2\|PDE5A\|CDK5R1 |
|  | GO:0030522 | intracellular receptor signaling pathway | 1E-26 | 25 | 24 | 24 | AR\|ESR1\|ESR2\|ESRRA\|NR3C1\|JAK2\|NR3C2\|PGR\|PIM1\|PPARA\|PPARG\|RARA\|RARB\|RARG\|RORA\|RXRA\|RXRB\|SRC\|NR1H2\|VDR\|NR1I2\|NR1I3\|NR1H4\|NR1H3 |
|  | GO:0031347 | regulation of defense response | 1E-25 | 8.9 | 17 | 39 | PARP1\|XIAP\|ARG1\|BST1\|BTK\|CASP1\|CMA1\|MAPK14\|ACE\|ELANE\|ESR1\|FABP4\|GSTP1\|HCK\|HSP90AA1\|IGF1\|IL2\|JAK2\|MMP3\|MMP8\|MMP9\|MMP12\|PIK3CG\|PLA2G2A\|PPARA\|PPARG\|PTPN1\|PTPN11\|RORA\|S100A9\|SELE\|SRC\|STAT1\|SYK\|TEK\|NR1H2\|PLA2G10\|NR1H4\|NR1H3 |
|  | GO:0001934 | positive regulation of protein phosphorylation | 1E-24 | 8.3 | 16 | 40 | ABL1\|AKT2\|BMP2\|BRAF\|CALM1\|CALM2\|CALM3\|CCNT1\|CSK\|ACE\|EGFR\|ELANE\|F2\|FGFR1\|HSP90AA1\|HSP90AB1\|IGF1\|IL2\|INSR\|JAK2\|KDR\|KIT\|MMP9\|PDPK1\|PGF\|PIK3CG\|PPARG\|PPIA\|MAPK1\|MAP2K1\|PTPN1\|PTPN11\|SRC\|SYK\|ADAM17\|TEK\|TGFB2\|TGFBR1\|PDE5A\|CDK5R1 |
|  | GO:0009410 | response to xenobiotic stimulus | 1E-24 | 12 | 18 | 32 | ABL1\|APOA2\|ARG1\|BCHE\|BRAF\|CASP3\|CCNT1\|CYP2C9\|ACE\|NQO1\|DPEP1\|DUSP6\|GSTM1\|GSTM2\|GSTP1\|HMOX1\|HSP90AA1\|HSP90AB1\|LCK\|MMP2\|NOS2\|PGF\|REN\|RORA\|SOD2\|SRC\|STAT1\|SULT2A1\|ADAM17\|TGFB2\|TYMS\|NR1I2 |
|  | GO:0071407 | cellular response to organic cyclic compound | 1E-24 | 10 | 17 | 34 | ABL1\|AR\|ARG1\|BMP2\|CASP3\|CASP7\|CCNA2\|ACE\|EGFR\|EIF4E\|ESR1\|ESR2\|ESRRA\|NR3C1\|GSK3B\|GSTM2\|HSPA8\|HSP90AB1\|IGF1R\|JAK2\|NR3C2\|MMP2\|PGR\|PIM1\|PIK3CG\|PPARA\|MAPK1\|RORA\|RXRA\|RXRB\|SRC\|STAT1\|VDR\|NR1H4 |
|  | GO:0050727 | regulation of inflammatory response | 1E-23 | 12 | 17 | 31 | XIAP\|BST1\|BTK\|CASP1\|CMA1\|MAPK14\|ACE\|ELANE\|ESR1\|FABP4\|GSTP1\|HCK\|IGF1\|IL2\|JAK2\|MMP3\|MMP8\|MMP9\|PIK3CG\|PLA2G2A\|PPARA\|PPARG\|RORA\|S100A9\|SELE\|SRC\|SYK\|TEK\|PLA2G10\|NR1H4\|NR1H3 |
|  | GO:1901699 | cellular response to nitrogen compound | 1E-23 | 8.6 | 16 | 36 | ABL1\|PARP1\|AKT2\|ARG1\|BCL2L1\|CA2\|CASP3\|CASP7\|CCNA2\|CDK2\|CSK\|DPEP1\|EGFR\|GCK\|GRB2\|GSK3B\|GSTM2\|IGF1\|IGF1R\|INSR\|JAK2\|JAK3\|MDM2\|MMP2\|MMP3\|PDPK1\|PIK3CG\|PIK3R1\|PPARG\|MAPK1\|PTPN1\|SRC\|STAT1\|KAT2B\|CDK5R1\|NR1H4 |
|  | GO:0018108 | peptidyl-tyrosine phosphorylation | 1E-22 | 21 | 21 | 22 | ABL1\|BTK\|CSK\|EGFR\|EPHB4\|FGFR1\|FGFR2\|HCK\|IGF1R\|IL2\|INSR\|JAK2\|JAK3\|KDR\|KIT\|LCK\|MET\|MAP2K1\|SRC\|SYK\|TEK\|ZAP70 |
|  | GO:0046777 | protein autophosphorylation | 1E-22 | 19 | 20 | 23 | ABL1\|BTK\|CSK\|EGFR\|EPHB4\|FGFR1\|FGFR2\|GSK3B\|HCK\|IGF1R\|INSR\|JAK2\|KDR\|KIT\|LCK\|PDPK1\|PIM1\|SRC\|AURKA\|SYK\|TEK\|ZAP70\|MAPKAPK2 |
|  | GO:0018212 | peptidyl-tyrosine modification | 1E-22 | 21 | 21 | 22 | ABL1\|BTK\|CSK\|EGFR\|EPHB4\|FGFR1\|FGFR2\|HCK\|IGF1R\|IL2\|INSR\|JAK2\|JAK3\|KDR\|KIT\|LCK\|MET\|MAP2K1\|SRC\|SYK\|TEK\|ZAP70 |
|  | GO:1901652 | response to peptide | 1E-22 | 11 | 17 | 31 | PARP1\|AKT2\|ARG1\|CA2\|CCNA2\|MAPK14\|CSK\|CTSK\|GCK\|GRB2\|GSK3B\|IGF1\|IGF1R\|INSR\|JAK2\|JAK3\|LTA4H\|MDM2\|MMP2\|MMP3\|MMP9\|MMP12\|MMP13\|PDPK1\|PIK3R1\|PPARA\|PPARG\|PTPN1\|SRC\|STAT1\|KAT2B |
|  | GO:0045859 | regulation of protein kinase activity | 1E-22 | 8.3 | 15 | 36 | ABL1\|BMP2\|CALM1\|CALM2\|CALM3\|CASP3\|CCNA2\|CCNT1\|CDK7\|CSK\|ACE\|EGFR\|ELANE\|FABP4\|FGFR1\|GSTP1\|HMGCR\|HSP90AA1\|HSP90AB1\|IGF1\|INSR\|JAK2\|KIT\|PDPK1\|PIK3CG\|PPARG\|PPIA\|MAP2K1\|PTPN1\|SRC\|SYK\|ADAM17\|TGFB2\|PDE5A\|KAT2B\|CDK5R1 |
|  | GO:0048545 | response to steroid hormone | 1E-22 | 14 | 18 | 26 | PARP1\|APOA2\|AR\|ARG1\|BCHE\|CALM3\|CASP3\|ACE\|EIF4E\|ESR1\|ESR2\|ESRRA\|GPI\|NR3C1\|HSPA8\|IGF1R\|JAK2\|NR3C2\|PGR\|PPARA\|RXRA\|RXRB\|SRC\|TGFB2\|TYMS\|NR1H3 |
|  | GO:0043408 | regulation of MAPK cascade | 1E-21 | 8 | 15 | 36 | ABL1\|XIAP\|AR\|BMP2\|BRAF\|CSK\|DUSP6\|EGFR\|ELANE\|FGFR1\|FGFR2\|GRB2\|GSTP1\|HMGCR\|IGF1\|IGF1R\|INSR\|JAK2\|KDR\|KIT\|MMP8\|PIK3CG\|PLA2G2A\|PPARG\|PPIA\|MAPK1\|MAP2K1\|PTPN1\|PTPN11\|REN\|SRC\|SYK\|TEK\|TGFB2\|TGFBR1\|PDE5A |
|  | GO:0032496 | response to lipopolysaccharide | 1E-21 | 13 | 17 | 27 | ABL1\|ARG1\|BTK\|CASP1\|CASP3\|MAPK14\|CTSG\|ACE\|ELANE\|FGFR2\|GSTP1\|HCK\|JAK2\|NOS2\|NOS3\|MAPK1\|MAPK8\|RARA\|REN\|S100A9\|SELE\|SOD2\|SRC\|ADAM17\|MAPKAPK2\|NR1H4\|NR1H3 |
|  | GO:0009617 | response to bacterium | 1E-21 | 7.6 | 15 | 37 | ABL1\|ARG1\|BMP2\|BTK\|CASP1\|CASP3\|CHIT1\|MAPK14\|CTSG\|ACE\|CFD\|ELANE\|F2\|FABP4\|FGFR2\|GSTP1\|HCK\|JAK2\|LCN2\|LYZ\|NOS2\|NOS3\|PLA2G2A\|MAPK1\|MAPK8\|RARA\|REN\|RNASE3\|S100A9\|SELE\|SOD2\|SRC\|SYK\|ADAM17\|MAPKAPK2\|NR1H4\|NR1H3 |
|  | GO:0002237 | response to molecule of bacterial origin | 1E-21 | 12 | 17 | 27 | ABL1\|ARG1\|BTK\|CASP1\|CASP3\|MAPK14\|CTSG\|ACE\|ELANE\|FGFR2\|GSTP1\|HCK\|JAK2\|NOS2\|NOS3\|MAPK1\|MAPK8\|RARA\|REN\|S100A9\|SELE\|SOD2\|SRC\|ADAM17\|MAPKAPK2\|NR1H4\|NR1H3 |
|  | GO:0071900 | regulation of protein serine/threonine kinase activity | 1E-20 | 11 | 16 | 28 | ABL1\|BMP2\|CALM1\|CALM2\|CALM3\|CASP3\|CCNA2\|CCNT1\|CDK7\|CSK\|EGFR\|ELANE\|FGFR1\|GSTP1\|HMGCR\|HSP90AB1\|INSR\|KIT\|PIK3CG\|PPARG\|MAP2K1\|PTPN1\|SRC\|SYK\|ADAM17\|PDE5A\|KAT2B\|CDK5R1 |
|  | GO:0009611 | response to wounding | 1E-20 | 10 | 16 | 29 | ANXA5\|ARG1\|CASP3\|MAPK14\|CTSG\|DHFR\|F2\|F10\|FGFR2\|HMOX1\|IGF1\|JAK2\|KDR\|LCK\|MMP2\|MMP12\|SERPINA1\|PIK3CG\|PPARA\|PPIA\|SOD2\|SRC\|AURKA\|SYK\|ADAM17\|TGFB2\|TGFBR1\|WAS\|PROCR |
|  | GO:0009755 | hormone-mediated signaling pathway | 1E-20 | 23 | 20 | 19 | AR\|ESR1\|ESR2\|ESRRA\|NR3C1\|JAK2\|NR3C2\|PGR\|PPARA\|PPARG\|PTPN11\|RARA\|RARB\|RARG\|REN\|RXRA\|RXRB\|SRC\|NR1H3 |
|  | GO:0010035 | response to inorganic substance | 1E-20 | 9 | 15 | 31 | ABL1\|PARP1\|ARG1\|ATIC\|BRAF\|CALM1\|CALM2\|CALM3\|CASP3\|CCNA2\|CDK2\|NQO1\|DPEP1\|EGFR\|FABP4\|GART\|GPI\|HMOX1\|KDR\|KIT\|LTA4H\|MMP2\|MMP3\|MMP9\|NOS3\|MAPK1\|MAPK8\|SOD2\|SRC\|STAT1\|TNNC1 |
|  | GO:0071417 | cellular response to organonitrogen compound | 1E-20 | 8.4 | 15 | 32 | ABL1\|PARP1\|AKT2\|ARG1\|BCL2L1\|CA2\|CASP3\|CASP7\|CCNA2\|CSK\|EGFR\|GCK\|GRB2\|GSK3B\|GSTM2\|IGF1\|IGF1R\|INSR\|JAK2\|JAK3\|MDM2\|MMP2\|PDPK1\|PIK3CG\|PIK3R1\|PPARG\|MAPK1\|PTPN1\|SRC\|STAT1\|KAT2B\|NR1H4 |
|  | GO:0048732 | gland development | 1E-20 | 10 | 16 | 28 | ABL1\|AKT2\|AR\|ARG1\|BMP2\|BRAF\|EGFR\|ESR1\|FGFR2\|NR3C1\|HMOX1\|INSR\|JAK2\|MET\|MMP2\|PGR\|MAPK1\|MAP2K1\|RARA\|RARG\|SOD2\|SRC\|AURKA\|TGFB2\|TGFBR1\|TPH1\|TYMS\|VDR |
|  | GO:0045860 | positive regulation of protein kinase activity | 1E-20 | 10 | 16 | 28 | ABL1\|BMP2\|CALM1\|CALM2\|CALM3\|CCNT1\|CSK\|ACE\|EGFR\|ELANE\|FGFR1\|HSP90AA1\|HSP90AB1\|IGF1\|INSR\|JAK2\|KIT\|PDPK1\|PIK3CG\|PPIA\|MAP2K1\|PTPN1\|SRC\|SYK\|ADAM17\|TGFB2\|PDE5A\|CDK5R1 |
|  | GO:0043410 | positive regulation of MAPK cascade | 1E-19 | 9 | 15 | 29 | ABL1\|XIAP\|AR\|BMP2\|BRAF\|CSK\|EGFR\|ELANE\|FGFR1\|FGFR2\|IGF1\|IGF1R\|INSR\|JAK2\|KDR\|KIT\|MMP8\|PIK3CG\|PLA2G2A\|PPIA\|MAP2K1\|PTPN1\|PTPN11\|SRC\|SYK\|TEK\|TGFB2\|TGFBR1\|PDE5A |
|  | GO:0030335 | positive regulation of cell migration | 1E-18 | 8.2 | 14 | 30 | ABL1\|AKT2\|BMP2\|EGFR\|F10\|FGFR1\|GPI\|HMOX1\|IGF1\|IGF1R\|INSR\|JAK2\|KDR\|KIT\|LGALS3\|MET\|MMP2\|MMP9\|NOS3\|PDPK1\|PGF\|PIK3CG\|PIK3R1\|MAPK1\|SOD2\|SRC\|ADAM17\|TEK\|TGFB2\|TGFBR1 |
|  | GO:2000147 | positive regulation of cell motility | 1E-18 | 7.8 | 14 | 30 | ABL1\|AKT2\|BMP2\|EGFR\|F10\|FGFR1\|GPI\|HMOX1\|IGF1\|IGF1R\|INSR\|JAK2\|KDR\|KIT\|LGALS3\|MET\|MMP2\|MMP9\|NOS3\|PDPK1\|PGF\|PIK3CG\|PIK3R1\|MAPK1\|SOD2\|SRC\|ADAM17\|TEK\|TGFB2\|TGFBR1 |
|  | GO:0040017 | positive regulation of locomotion | 1E-17 | 7.6 | 13 | 30 | ABL1\|AKT2\|BMP2\|EGFR\|F10\|FGFR1\|GPI\|HMOX1\|IGF1\|IGF1R\|INSR\|JAK2\|KDR\|KIT\|LGALS3\|MET\|MMP2\|MMP9\|NOS3\|PDPK1\|PGF\|PIK3CG\|PIK3R1\|MAPK1\|SOD2\|SRC\|ADAM17\|TEK\|TGFB2\|TGFBR1 |
|  | GO:0048729 | tissue morphogenesis | 1E-17 | 7.9 | 13 | 29 | ABL1\|AR\|BMP2\|CA2\|CASP3\|EGFR\|ESR1\|FGFR2\|GPI\|GRB2\|NR3C1\|KDR\|MDM2\|MET\|MMP2\|MMP12\|MTHFD1\|NOS3\|PGR\|RARA\|RARG\|SRC\|ADAM17\|TGFB2\|TGFBR1\|TGM3\|TNNC1\|VDR\|PLA2G10 |
|  | GO:0001819 | positive regulation of cytokine production | 1E-17 | 8.6 | 14 | 27 | ABL1\|APOA2\|BTK\|CASP1\|MAPK14\|ELANE\|HMOX1\|HSP90AA1\|IL2\|JAK2\|KIT\|MMP8\|MMP12\|NOS2\|PDE4D\|PIK3CG\|PIK3R1\|PRKCQ\|PTPN11\|RARA\|RORA\|SRC\|STAT1\|SYK\|ADAM17\|MAPKAPK2\|NR1H4 |
|  | GO:0043434 | response to peptide hormone | 1E-17 | 10 | 14 | 24 | PARP1\|AKT2\|ARG1\|CA2\|CCNA2\|MAPK14\|CSK\|CTSK\|GCK\|GRB2\|GSK3B\|IGF1R\|INSR\|JAK2\|JAK3\|LTA4H\|PDPK1\|PIK3R1\|PPARA\|PPARG\|PTPN1\|SRC\|STAT1\|KAT2B |
|  | GO:0044057 | regulation of system process | 1E-17 | 7.6 | 13 | 29 | ABL1\|PARP1\|APOA2\|CALM1\|CALM2\|CALM3\|ACE\|FABP5\|GSTM2\|HSP90AA1\|IGF1\|IL2\|JAK2\|KIT\|MDM2\|MMP2\|NOS3\|PDE4D\|PIK3CG\|PPARA\|PPARG\|PTPN11\|REN\|SRC\|TGFB2\|TNNC1\|NR1H2\|PDE5A\|NR1H3 |
|  | GO:0048534 | hematopoietic or lymphoid organ development | 1E-17 | 6.9 | 13 | 31 | ABL1\|PARP1\|BRAF\|BTK\|CASP3\|MAPK14\|CTSK\|ACE\|FGFR2\|IL2\|JAK2\|JAK3\|KDR\|KIT\|LCK\|MMP9\|PIK3R1\|PPARG\|MAPK1\|MAP2K1\|PTPN11\|RORA\|SOD2\|SRC\|SYK\|ADAM17\|TEK\|TGFB2\|TGFBR1\|ZAP70\|PLA2G10 |
|  | GO:0042060 | wound healing | 1E-16 | 11 | 14 | 23 | ANXA5\|CASP3\|MAPK14\|CTSG\|F2\|F10\|FGFR2\|HMOX1\|IGF1\|KDR\|LCK\|MMP12\|SERPINA1\|PIK3CG\|PPARA\|PPIA\|SRC\|SYK\|ADAM17\|TGFB2\|TGFBR1\|WAS\|PROCR |
|  | GO:0032103 | positive regulation of response to external stimulus | 1E-16 | 8.7 | 13 | 26 | ARG1\|BRAF\|BTK\|CASP1\|ACE\|F2\|FABP4\|FGFR1\|HCK\|HSP90AA1\|IGF1R\|IL2\|JAK2\|KDR\|MET\|MMP8\|MMP12\|PGF\|PIK3CG\|PLA2G2A\|MAPK1\|S100A9\|SRC\|SYK\|ADAM17\|NR1H4 |
|  | GO:0071902 | positive regulation of protein serine/threonine kinase activity | 1E-16 | 14 | 15 | 20 | BMP2\|CALM1\|CALM2\|CALM3\|CCNT1\|CSK\|EGFR\|ELANE\|FGFR1\|HSP90AB1\|INSR\|KIT\|PIK3CG\|MAP2K1\|PTPN1\|SRC\|SYK\|ADAM17\|PDE5A\|CDK5R1 |
|  | GO:0050865 | regulation of cell activation | 1E-16 | 6.6 | 12 | 31 | ABL1\|ARG1\|BRAF\|BST1\|BTK\|CASP3\|CSK\|CTSG\|DPP4\|F2\|HMOX1\|IGF1\|IL2\|JAK2\|JAK3\|LCK\|LGALS3\|MMP8\|NOS3\|PDPK1\|PLA2G2A\|PRKCQ\|PTPN11\|RARA\|RORA\|SRC\|SYK\|ZAP70\|PLA2G10\|PDE5A\|NR1H3 |
|  | GO:0048511 | rhythmic process | 1E-16 | 12 | 15 | 21 | CASP3\|ESR1\|GSK3B\|IGF1R\|IMPDH2\|MMP2\|NOS2\|NOS3\|PGR\|PPARA\|PPARG\|PPP1CC\|MAPK8\|MAPK10\|RORA\|SRC\|TGFB2\|TPH1\|TYMS\|KAT2B\|CDK5R1 |
|  | GO:1901653 | cellular response to peptide | 1E-16 | 11 | 14 | 22 | PARP1\|AKT2\|ARG1\|CA2\|CCNA2\|CSK\|GCK\|GRB2\|GSK3B\|IGF1\|IGF1R\|INSR\|JAK2\|JAK3\|MDM2\|PDPK1\|PIK3R1\|PPARG\|PTPN1\|SRC\|STAT1\|KAT2B |
|  | GO:0022617 | extracellular matrix disassembly | 1E-16 | 40 | 22 | 12 | CMA1\|CTSG\|CTSK\|CTSS\|ELANE\|MMP1\|MMP2\|MMP3\|MMP8\|MMP9\|MMP12\|MMP13 |
|  | GO:0071375 | cellular response to peptide hormone stimulus | 1E-16 | 13 | 15 | 20 | PARP1\|AKT2\|ARG1\|CA2\|CCNA2\|CSK\|GCK\|GRB2\|GSK3B\|IGF1R\|INSR\|JAK2\|JAK3\|PDPK1\|PIK3R1\|PPARG\|PTPN1\|SRC\|STAT1\|KAT2B |
|  | GO:0071383 | cellular response to steroid hormone stimulus | 1E-16 | 18 | 16 | 17 | AR\|ARG1\|ACE\|EIF4E\|ESR1\|ESR2\|ESRRA\|NR3C1\|HSPA8\|IGF1R\|JAK2\|NR3C2\|PGR\|PPARA\|RXRA\|RXRB\|SRC |
|  | GO:0002520 | immune system development | 1E-16 | 6.4 | 12 | 31 | ABL1\|PARP1\|BRAF\|BTK\|CASP3\|MAPK14\|CTSK\|ACE\|FGFR2\|IL2\|JAK2\|JAK3\|KDR\|KIT\|LCK\|MMP9\|PIK3R1\|PPARG\|MAPK1\|MAP2K1\|PTPN11\|RORA\|SOD2\|SRC\|SYK\|ADAM17\|TEK\|TGFB2\|TGFBR1\|ZAP70\|PLA2G10 |
|  | GO:0008283 | cell population proliferation | 1E-16 | 6.6 | 12 | 30 | ABL1\|AR\|BCL2L1\|BMP2\|BTK\|ACE\|EGFR\|ESR1\|FGFR2\|GSTP1\|HMOX1\|IGF1\|IL2\|IMPDH2\|KDR\|KIT\|MMP16\|PIK3CG\|PRKCQ\|MAPK1\|MAP2K1\|RARB\|RARG\|RORA\|SRC\|STAT1\|TEK\|TGFB2\|TGFBR1\|PDE5A |
|  | GO:0007507 | heart development | 1E-15 | 7.5 | 13 | 27 | ABL1\|BMP2\|CASP3\|CASP7\|ACE\|EPHB4\|FGFR2\|IGF1R\|INSR\|KDR\|MDM2\|MMP2\|MTHFD1\|NOS3\|PPARA\|MAPK1\|MAP2K1\|PTPN11\|RARA\|RARB\|RBP4\|SOD2\|TEK\|TGFB2\|TGFBR1\|TNNC1\|KAT2B |
|  | GO:0051098 | regulation of binding | 1E-15 | 9.6 | 13 | 23 | ABL1\|PARP1\|BMP2\|CALM1\|CALM2\|CALM3\|ACE\|GSK3B\|HMOX1\|HSP90AB1\|IGF1\|JAK2\|MET\|MMP8\|MMP9\|PPARA\|PPARG\|MAPK8\|RARA\|SRC\|AURKA\|TGFBR1\|DOT1L |
|  | GO:0010942 | positive regulation of cell death | 1E-15 | 7.1 | 12 | 28 | ABL1\|PARP1\|BMP2\|CASP3\|ACE\|NQO1\|DUSP6\|NR3C1\|GSK3B\|HMOX1\|JAK2\|LCK\|MMP2\|MMP3\|MMP9\|NOS2\|PPARG\|MAPK8\|RARB\|RARG\|S100A9\|SOD2\|SRC\|SYK\|TGFB2\|TGFBR1\|VDR\|CDK5R1 |
|  | GO:0002694 | regulation of leukocyte activation | 1E-15 | 6.7 | 12 | 29 | ABL1\|ARG1\|BRAF\|BST1\|BTK\|CASP3\|CSK\|CTSG\|DPP4\|HMOX1\|IGF1\|IL2\|JAK2\|JAK3\|LCK\|LGALS3\|MMP8\|PDPK1\|PLA2G2A\|PRKCQ\|PTPN11\|RARA\|RORA\|SRC\|SYK\|ZAP70\|PLA2G10\|PDE5A\|NR1H3 |
|  | GO:0035239 | tube morphogenesis | 1E-15 | 6.7 | 12 | 29 | ABL1\|AR\|BMP2\|CASP3\|MAPK14\|ACE\|EGFR\|EPHB4\|ESR1\|FGFR2\|NR3C1\|HMOX1\|KDR\|MET\|MMP2\|MTHFD1\|NOS3\|PGF\|PGR\|PIK3CG\|RARA\|RARG\|RORA\|SRC\|SYK\|TEK\|TGFB2\|TGFBR1\|VDR |
|  | GO:0010817 | regulation of hormone levels | 1E-15 | 7.8 | 13 | 26 | ADH1C\|AKR1B1\|BCHE\|BMP2\|BTK\|CMA1\|CRABP2\|CTSB\|CTSG\|CTSK\|CYP2C9\|ACE\|DPP4\|ESR1\|GCK\|NR3C1\|IGF1R\|JAK2\|NOS2\|PPARG\|PTPN11\|RBP4\|REN\|SULT2A1\|TTR\|NR1H4 |
|  | GO:0070848 | response to growth factor | 1E-15 | 7.8 | 13 | 26 | ABL1\|PARP1\|ARG1\|BMP2\|CASP3\|CCNA2\|MAPK14\|CTSK\|DUSP6\|EGFR\|FGFR1\|FGFR2\|GRB2\|NR3C1\|IGF1R\|INSR\|KDR\|PDPK1\|PGF\|PPARG\|MAPK1\|PTPN11\|SRC\|TGFB2\|TGFBR1\|MAPKAPK2 |
| KEGG Pathway | hsa05200 | Pathways in cancer | 1E-51 | 16 | 29 | 56 | ABL1\|AKT2\|XIAP\|AR\|BCL2L1\|BMP2\|BRAF\|CALM1\|CALM2\|CALM3\|CASP3\|CASP7\|CCNA2\|CDK2\|NQO1\|EGFR\|ESR1\|ESR2\|F2\|FGFR1\|FGFR2\|GRB2\|GSK3B\|GSTM1\|GSTM2\|GSTP1\|HMOX1\|HSP90AA1\|HSP90AB1\|IGF1\|IGF1R\|IL2\|JAK2\|JAK3\|KIT\|MDM2\|MET\|MMP1\|MMP2\|MMP9\|NOS2\|PGF\|PIM1\|PIK3R1\|PPARG\|MAPK1\|MAPK8\|MAPK10\|MAP2K1\|RARA\|RARB\|RXRA\|RXRB\|STAT1\|TGFB2\|TGFBR1 |
|  | hsa05417 | Lipid and atherosclerosis | 1E-32 | 22 | 25 | 31 | AKT2\|BCL2L1\|CALM1\|CALM2\|CALM3\|CASP1\|CASP3\|CASP7\|MAPK14\|CYP2C9\|GSK3B\|HSPA8\|HSP90AA1\|HSP90AB1\|JAK2\|MMP1\|MMP3\|MMP9\|NOS3\|PDPK1\|PIK3R1\|PPARG\|MAPK1\|MAPK8\|MAPK10\|RXRA\|RXRB\|MAPK12\|SELE\|SOD2\|SRC |
|  | hsa05215 | Prostate cancer | 1E-26 | 33 | 26 | 21 | AKT2\|AR\|BRAF\|CDK2\|EGFR\|FGFR1\|FGFR2\|GRB2\|GSK3B\|GSTP1\|HSP90AA1\|HSP90AB1\|IGF1\|IGF1R\|MDM2\|MMP3\|MMP9\|PDPK1\|PIK3R1\|MAPK1\|MAP2K1 |
|  | hsa04151 | PI3K-Akt signaling pathway | 1E-25 | 14 | 19 | 31 | AKT2\|BCL2L1\|CDK2\|EGFR\|EIF4E\|FGFR1\|FGFR2\|GRB2\|GSK3B\|HSP90AA1\|HSP90AB1\|IGF1\|IGF1R\|IL2\|INSR\|JAK2\|JAK3\|KDR\|KIT\|MDM2\|MET\|NOS3\|PDPK1\|PGF\|PIK3CG\|PIK3R1\|MAPK1\|MAP2K1\|RXRA\|SYK\|TEK |
|  | hsa04014 | Ras signaling pathway | 1E-25 | 18 | 21 | 27 | ABL1\|AKT2\|BCL2L1\|CALM1\|CALM2\|CALM3\|EGFR\|FGFR1\|FGFR2\|GRB2\|IGF1\|IGF1R\|INSR\|KDR\|KIT\|MET\|PGF\|PIK3R1\|PLA2G2A\|MAPK1\|MAPK8\|MAPK10\|MAP2K1\|PTPN11\|TEK\|ZAP70\|PLA2G10 |
|  | hsa05418 | Fluid shear stress and atherosclerosis | 1E-24 | 24 | 22 | 22 | AKT2\|CALM1\|CALM2\|CALM3\|MAPK14\|NQO1\|GSTM1\|GSTM2\|GSTP1\|HMOX1\|HSP90AA1\|HSP90AB1\|KDR\|MMP2\|MMP9\|NOS3\|PIK3R1\|MAPK8\|MAPK10\|MAPK12\|SELE\|SRC |
|  | hsa05205 | Proteoglycans in cancer | 1E-23 | 18 | 20 | 24 | AKT2\|BRAF\|CASP3\|MAPK14\|EGFR\|ESR1\|FGFR1\|GRB2\|IGF1\|IGF1R\|KDR\|MDM2\|MET\|MMP2\|MMP9\|PDPK1\|PIK3R1\|PPP1CC\|MAPK1\|MAP2K1\|PTPN11\|MAPK12\|SRC\|TGFB2 |
|  | hsa01522 | Endocrine resistance | 1E-22 | 30 | 23 | 19 | AKT2\|BRAF\|MAPK14\|EGFR\|ESR1\|ESR2\|GRB2\|IGF1\|IGF1R\|MDM2\|MMP2\|MMP9\|PIK3R1\|MAPK1\|MAPK8\|MAPK10\|MAP2K1\|MAPK12\|SRC |
|  | hsa04659 | Th17 cell differentiation | 1E-21 | 27 | 22 | 19 | MAPK14\|HSP90AA1\|HSP90AB1\|IL2\|JAK2\|JAK3\|LCK\|PRKCQ\|MAPK1\|MAPK8\|MAPK10\|RARA\|RORA\|RXRA\|RXRB\|MAPK12\|STAT1\|TGFBR1\|ZAP70 |
|  | hsa04010 | MAPK signaling pathway | 1E-21 | 14 | 18 | 26 | AKT2\|BRAF\|CASP3\|MAPK14\|DUSP6\|EGFR\|FGFR1\|FGFR2\|GRB2\|HSPA8\|IGF1\|IGF1R\|INSR\|KDR\|KIT\|MET\|PGF\|MAPK1\|MAPK8\|MAPK10\|MAP2K1\|MAPK12\|TEK\|TGFB2\|TGFBR1\|MAPKAPK2 |
|  | hsa04068 | FoxO signaling pathway | 1E-21 | 24 | 21 | 20 | AKT2\|BRAF\|CDK2\|MAPK14\|EGFR\|GRB2\|IGF1\|IGF1R\|INSR\|MDM2\|PDPK1\|PIK3R1\|MAPK1\|MAPK8\|MAPK10\|MAP2K1\|MAPK12\|SOD2\|TGFB2\|TGFBR1 |
|  | hsa04015 | Rap1 signaling pathway | 1E-21 | 17 | 19 | 23 | AKT2\|BRAF\|CALM1\|CALM2\|CALM3\|MAPK14\|EGFR\|FGFR1\|FGFR2\|IGF1\|IGF1R\|INSR\|ITGAL\|KDR\|KIT\|MET\|PGF\|PIK3R1\|MAPK1\|MAP2K1\|MAPK12\|SRC\|TEK |
|  | hsa01521 | EGFR tyrosine kinase inhibitor resistance | 1E-21 | 33 | 23 | 17 | AKT2\|BCL2L1\|BRAF\|EGFR\|EIF4E\|FGFR2\|GRB2\|GSK3B\|IGF1\|IGF1R\|JAK2\|KDR\|MET\|PIK3R1\|MAPK1\|MAP2K1\|SRC |
|  | hsa04915 | Estrogen signaling pathway | 1E-21 | 22 | 20 | 20 | AKT2\|CALM1\|CALM2\|CALM3\|EGFR\|ESR1\|ESR2\|GRB2\|HSPA8\|HSP90AA1\|HSP90AB1\|MMP2\|MMP9\|NOS3\|PGR\|PIK3R1\|MAPK1\|MAP2K1\|RARA\|SRC |
|  | hsa04625 | C-type lectin receptor signaling pathway | 1E-20 | 27 | 21 | 18 | AKT2\|CALM1\|CALM2\|CALM3\|CASP1\|MAPK14\|IL2\|MDM2\|PIK3R1\|MAPK1\|MAPK8\|MAPK10\|PTPN11\|MAPK12\|SRC\|STAT1\|SYK\|MAPKAPK2 |
|  | hsa04917 | Prolactin signaling pathway | 1E-20 | 35 | 23 | 16 | AKT2\|MAPK14\|ESR1\|ESR2\|GCK\|GRB2\|GSK3B\|JAK2\|PIK3R1\|MAPK1\|MAPK8\|MAPK10\|MAP2K1\|MAPK12\|SRC\|STAT1 |
|  | hsa05207 | Chemical carcinogenesis - receptor activation | 1E-20 | 16 | 18 | 22 | AKT2\|XIAP\|AR\|EGFR\|ESR1\|ESR2\|GRB2\|GSTM1\|GSTM2\|HSP90AA1\|HSP90AB1\|JAK2\|PGR\|PIK3R1\|PPARA\|MAPK1\|MAP2K1\|RXRA\|RXRB\|SRC\|VDR\|NR1I3 |
|  | hsa05161 | Hepatitis B | 1E-19 | 19 | 19 | 20 | AKT2\|BRAF\|CASP3\|CCNA2\|CDK2\|MAPK14\|GRB2\|JAK2\|JAK3\|MMP9\|PIK3R1\|MAPK1\|MAPK8\|MAPK10\|MAP2K1\|MAPK12\|SRC\|STAT1\|TGFB2\|TGFBR1 |
|  | hsa04722 | Neurotrophin signaling pathway | 1E-19 | 23 | 20 | 18 | ABL1\|AKT2\|BRAF\|CALM1\|CALM2\|CALM3\|MAPK14\|GRB2\|GSK3B\|PDPK1\|PIK3R1\|MAPK1\|MAPK8\|MAPK10\|MAP2K1\|PTPN11\|MAPK12\|MAPKAPK2 |
|  | hsa05208 | Chemical carcinogenesis - reactive oxygen species | 1E-19 | 15 | 17 | 22 | ABL1\|AKT2\|BRAF\|MAPK14\|NQO1\|EGFR\|GRB2\|GSTM1\|GSTM2\|HMOX1\|MET\|PDPK1\|PIK3R1\|MAPK1\|MAPK8\|MAPK10\|MAP2K1\|PTPN1\|PTPN11\|MAPK12\|SOD2\|SRC |
|  | hsa04914 | Progesterone-mediated oocyte maturation | 1E-19 | 26 | 20 | 17 | AKT2\|BRAF\|CCNA2\|CDK2\|MAPK14\|HSP90AA1\|HSP90AB1\|IGF1\|IGF1R\|PGR\|PIK3R1\|MAPK1\|MAPK8\|MAPK10\|MAP2K1\|MAPK12\|AURKA |
|  | hsa04926 | Relaxin signaling pathway | 1E-18 | 22 | 19 | 18 | AKT2\|MAPK14\|EGFR\|GRB2\|MMP1\|MMP2\|MMP9\|MMP13\|NOS2\|NOS3\|PIK3R1\|MAPK1\|MAPK8\|MAPK10\|MAP2K1\|MAPK12\|SRC\|TGFBR1 |
|  | hsa04910 | Insulin signaling pathway | 1E-18 | 20 | 18 | 18 | AKT2\|BRAF\|CALM1\|CALM2\|CALM3\|EIF4E\|GCK\|GRB2\|GSK3B\|INSR\|PDPK1\|PIK3R1\|PPP1CC\|MAPK1\|MAPK8\|MAPK10\|MAP2K1\|PTPN1 |
|  | hsa05167 | Kaposi sarcoma-associated herpesvirus infection | 1E-18 | 16 | 17 | 20 | AKT2\|CALM1\|CALM2\|CALM3\|CASP3\|MAPK14\|GSK3B\|HCK\|JAK2\|PIK3CG\|PIK3R1\|MAPK1\|MAPK8\|MAPK10\|MAP2K1\|MAPK12\|SRC\|STAT1\|SYK\|MAPKAPK2 |
|  | hsa04933 | AGE-RAGE signaling pathway in diabetic complications | 1E-17 | 25 | 19 | 16 | AKT2\|CASP3\|MAPK14\|JAK2\|MMP2\|NOS3\|PIM1\|PIK3R1\|MAPK1\|MAPK8\|MAPK10\|MAPK12\|SELE\|STAT1\|TGFB2\|TGFBR1 |
|  | hsa05415 | Diabetic cardiomyopathy | 1E-17 | 15 | 16 | 20 | PARP1\|AKT2\|CMA1\|MAPK14\|ACE\|GSK3B\|GSR\|INSR\|MMP2\|MMP9\|NOS3\|PIK3R1\|PPARA\|PPP1CC\|MAPK8\|MAPK10\|REN\|MAPK12\|TGFB2\|TGFBR1 |
|  | hsa04380 | Osteoclast differentiation | 1E-17 | 21 | 18 | 17 | AKT2\|BTK\|MAPK14\|CTSK\|GRB2\|LCK\|PIK3R1\|PPARG\|MAPK1\|MAPK8\|MAPK10\|MAP2K1\|MAPK12\|STAT1\|SYK\|TGFB2\|TGFBR1 |
|  | hsa05145 | Toxoplasmosis | 1E-17 | 22 | 18 | 16 | AKT2\|XIAP\|BCL2L1\|CASP3\|MAPK14\|HSPA8\|JAK2\|NOS2\|PDPK1\|PIK3CG\|MAPK1\|MAPK8\|MAPK10\|MAPK12\|STAT1\|TGFB2 |
|  | hsa04657 | IL-17 signaling pathway | 1E-16 | 25 | 19 | 15 | CASP3\|MAPK14\|GSK3B\|HSP90AA1\|HSP90AB1\|LCN2\|MMP1\|MMP3\|MMP9\|MMP13\|MAPK1\|MAPK8\|MAPK10\|S100A9\|MAPK12 |
|  | hsa05225 | Hepatocellular carcinoma | 1E-16 | 17 | 16 | 18 | AKT2\|BCL2L1\|BRAF\|NQO1\|EGFR\|GRB2\|GSK3B\|GSTM1\|GSTM2\|GSTP1\|HMOX1\|IGF1R\|MET\|PIK3R1\|MAPK1\|MAP2K1\|TGFB2\|TGFBR1 |
|  | hsa04510 | Focal adhesion | 1E-16 | 15 | 16 | 19 | AKT2\|XIAP\|BRAF\|EGFR\|GRB2\|GSK3B\|IGF1\|IGF1R\|KDR\|MET\|PDPK1\|PGF\|PIK3R1\|PPP1CC\|MAPK1\|MAPK8\|MAPK10\|MAP2K1\|SRC |
|  | hsa05152 | Tuberculosis | 1E-16 | 15 | 16 | 18 | AKT2\|CALM1\|CALM2\|CALM3\|CASP3\|MAPK14\|CTSS\|JAK2\|NOS2\|MAPK1\|MAPK8\|MAPK10\|MAPK12\|SRC\|STAT1\|SYK\|TGFB2\|VDR |
|  | hsa04660 | T cell receptor signaling pathway | 1E-16 | 22 | 18 | 15 | AKT2\|MAPK14\|GRB2\|GSK3B\|IL2\|LCK\|PDPK1\|PIK3R1\|PRKCQ\|MAPK1\|MAPK8\|MAPK10\|MAP2K1\|MAPK12\|ZAP70 |
|  | hsa04931 | Insulin resistance | 1E-15 | 21 | 17 | 15 | AKT2\|GSK3B\|INSR\|NOS3\|PDPK1\|PIK3R1\|PPARA\|PPP1CC\|PRKCQ\|MAPK8\|MAPK10\|PTPN1\|PTPN11\|NR1H2\|NR1H3 |
|  | hsa05223 | Non-small cell lung cancer | 1E-15 | 28 | 18 | 13 | AKT2\|BRAF\|EGFR\|GRB2\|JAK3\|MET\|PDPK1\|PIK3R1\|MAPK1\|MAP2K1\|RARB\|RXRA\|RXRB |
|  | hsa05214 | Glioma | 1E-15 | 27 | 18 | 13 | AKT2\|BRAF\|CALM1\|CALM2\|CALM3\|EGFR\|GRB2\|IGF1\|IGF1R\|MDM2\|PIK3R1\|MAPK1\|MAP2K1 |
|  | hsa05226 | Gastric cancer | 1E-15 | 17 | 15 | 16 | AKT2\|BRAF\|CDK2\|EGFR\|FGFR2\|GRB2\|GSK3B\|MET\|PIK3R1\|MAPK1\|MAP2K1\|RARB\|RXRA\|RXRB\|TGFB2\|TGFBR1 |
|  | hsa04218 | Cellular senescence | 1E-14 | 16 | 15 | 16 | AKT2\|CALM1\|CALM2\|CALM3\|CCNA2\|CDK2\|MAPK14\|MDM2\|PIK3R1\|PPP1CC\|MAPK1\|MAP2K1\|MAPK12\|TGFB2\|TGFBR1\|MAPKAPK2 |
|  | hsa05210 | Colorectal cancer | 1E-14 | 23 | 17 | 13 | AKT2\|BRAF\|CASP3\|EGFR\|GRB2\|GSK3B\|PIK3R1\|MAPK1\|MAPK8\|MAPK10\|MAP2K1\|TGFB2\|TGFBR1 |
|  | hsa04210 | Apoptosis | 1E-14 | 17 | 15 | 15 | PARP1\|AKT2\|XIAP\|BCL2L1\|CASP3\|CASP7\|CTSB\|CTSK\|CTSS\|PDPK1\|PIK3R1\|MAPK1\|MAPK8\|MAPK10\|MAP2K1 |
|  | hsa05135 | Yersinia infection | 1E-14 | 17 | 15 | 15 | AKT2\|CASP1\|MAPK14\|GSK3B\|IL2\|LCK\|PIK3R1\|MAPK1\|MAPK8\|MAPK10\|MAP2K1\|MAPK12\|SRC\|WAS\|ZAP70 |
|  | hsa04664 | Fc epsilon RI signaling pathway | 1E-14 | 27 | 18 | 12 | AKT2\|BTK\|MAPK14\|GRB2\|PDPK1\|PIK3R1\|MAPK1\|MAPK8\|MAPK10\|MAP2K1\|MAPK12\|SYK |
|  | hsa05235 | PD-L1 expression and PD-1 checkpoint pathway in cancer | 1E-14 | 23 | 16 | 13 | AKT2\|MAPK14\|EGFR\|JAK2\|LCK\|PIK3R1\|PRKCQ\|MAPK1\|MAP2K1\|PTPN11\|MAPK12\|STAT1\|ZAP70 |
|  | hsa04912 | GnRH signaling pathway | 1E-13 | 22 | 16 | 13 | CALM1\|CALM2\|CALM3\|MAPK14\|EGFR\|GRB2\|MMP2\|MAPK1\|MAPK8\|MAPK10\|MAP2K1\|MAPK12\|SRC |
|  | hsa05224 | Breast cancer | 1E-13 | 16 | 14 | 15 | AKT2\|BRAF\|EGFR\|ESR1\|ESR2\|FGFR1\|GRB2\|GSK3B\|IGF1\|IGF1R\|KIT\|PGR\|PIK3R1\|MAPK1\|MAP2K1 |
|  | hsa05133 | Pertussis | 1E-13 | 24 | 16 | 12 | CALM1\|CALM2\|CALM3\|CASP1\|CASP3\|CASP7\|MAPK14\|NOS2\|MAPK1\|MAPK8\|MAPK10\|MAPK12 |
|  | hsa05212 | Pancreatic cancer | 1E-13 | 24 | 16 | 12 | AKT2\|BCL2L1\|BRAF\|EGFR\|PIK3R1\|MAPK1\|MAPK8\|MAPK10\|MAP2K1\|STAT1\|TGFB2\|TGFBR1 |
|  | hsa05220 | Chronic myeloid leukemia | 1E-13 | 24 | 16 | 12 | ABL1\|AKT2\|BCL2L1\|BRAF\|GRB2\|MDM2\|PIK3R1\|MAPK1\|MAP2K1\|PTPN11\|TGFB2\|TGFBR1 |
|  | hsa05166 | Human T-cell leukemia virus 1 infection | 1E-13 | 12 | 13 | 17 | AKT2\|XIAP\|BCL2L1\|CCNA2\|CDK2\|IL2\|ITGAL\|JAK3\|LCK\|PIK3R1\|MAPK1\|MAPK8\|MAPK10\|MAP2K1\|TGFB2\|TGFBR1\|KAT2B |
|  | hsa05202 | Transcriptional misregulation in cancer | 1E-13 | 13 | 13 | 16 | BCL2L1\|CCNA2\|CCNT1\|DUSP6\|ELANE\|IGF1\|IGF1R\|MDM2\|MET\|MMP3\|MMP9\|PPARG\|RARA\|RXRA\|RXRB\|DOT1L |
|  | hsa04114 | Oocyte meiosis | 1E-13 | 17 | 14 | 14 | AR\|CALM1\|CALM2\|CALM3\|CDK2\|MAPK14\|IGF1\|IGF1R\|PGR\|PPP1CC\|MAPK1\|MAP2K1\|MAPK12\|AURKA |
|  | hsa05171 | Coronavirus disease - COVID-19 | 1E-13 | 11 | 13 | 17 | CASP1\|MAPK14\|ACE\|CFD\|EGFR\|F2\|IL2\|MMP1\|MMP3\|PIK3R1\|MAPK1\|MAPK8\|MAPK10\|MAPK12\|STAT1\|SYK\|ADAM17 |
|  | hsa04066 | HIF-1 signaling pathway | 1E-13 | 18 | 15 | 13 | AKT2\|EGFR\|EIF4E\|HMOX1\|IGF1\|IGF1R\|INSR\|NOS2\|NOS3\|PIK3R1\|MAPK1\|MAP2K1\|TEK |
|  | hsa04012 | ErbB signaling pathway | 1E-12 | 22 | 16 | 12 | ABL1\|AKT2\|BRAF\|EGFR\|GRB2\|GSK3B\|PIK3R1\|MAPK1\|MAPK8\|MAPK10\|MAP2K1\|SRC |
|  | hsa04668 | TNF signaling pathway | 1E-12 | 18 | 15 | 13 | AKT2\|CASP3\|CASP7\|MAPK14\|MMP3\|MMP9\|PIK3R1\|MAPK1\|MAPK8\|MAPK10\|MAP2K1\|MAPK12\|SELE |
|  | hsa05221 | Acute myeloid leukemia | 1E-12 | 25 | 16 | 11 | AKT2\|BRAF\|CCNA2\|DUSP6\|GRB2\|KIT\|PIM1\|PIK3R1\|MAPK1\|MAP2K1\|RARA |
|  | hsa04550 | Signaling pathways regulating pluripotency of stem cells | 1E-12 | 15 | 14 | 14 | AKT2\|MAPK14\|FGFR1\|FGFR2\|GRB2\|GSK3B\|IGF1\|IGF1R\|JAK2\|JAK3\|PIK3R1\|MAPK1\|MAP2K1\|MAPK12 |
|  | hsa04658 | Th1 and Th2 cell differentiation | 1E-12 | 20 | 15 | 12 | MAPK14\|IL2\|JAK2\|JAK3\|LCK\|PRKCQ\|MAPK1\|MAPK8\|MAPK10\|MAPK12\|STAT1\|ZAP70 |
|  | hsa05120 | Epithelial cell signaling in Helicobacter pylori infection | 1E-12 | 24 | 16 | 11 | CASP3\|MAPK14\|CSK\|EGFR\|MET\|MAPK8\|MAPK10\|PTPN11\|MAPK12\|SRC\|ADAM17 |
|  | hsa04935 | Growth hormone synthesis, secretion and action | 1E-12 | 17 | 14 | 13 | AKT2\|MAPK14\|GRB2\|GSK3B\|IGF1\|JAK2\|PIK3R1\|MAPK1\|MAPK8\|MAPK10\|MAP2K1\|MAPK12\|STAT1 |
| Reactome Pathways | R-HSA-383280 | Nuclear Receptor transcription pathway | 1E-32 | 62 | 36 | 21 | AR\|ESR1\|ESR2\|ESRRA\|NR3C1\|NR3C2\|PGR\|PPARA\|PPARG\|RARA\|RARB\|RARG\|RORA\|RXRA\|RXRB\|NR1H2\|VDR\|NR1I2\|NR1I3\|NR1H4\|NR1H3 |
|  | R-HSA-9006934 | Signaling by Receptor Tyrosine Kinases | 1E-32 | 12 | 21 | 41 | AKT2\|BRAF\|CALM1\|CALM2\|CALM3\|CMA1\|MAPK14\|CSK\|DUSP6\|EGFR\|ESR1\|FGFR1\|FGFR2\|GRB2\|HSP90AA1\|IGF1\|IGF1R\|INSR\|JAK2\|JAK3\|KDR\|KIT\|LCK\|MET\|MMP9\|NOS3\|PDPK1\|PGF\|PGR\|PIK3R1\|MAPK1\|MAP2K1\|PTPN1\|PTPN11\|MAPK12\|SRC\|STAT1\|ADAM17\|TPH1\|CDK5R1\|MAPKAPK2 |
|  | R-HSA-9006931 | Signaling by Nuclear Receptors | 1E-27 | 16 | 21 | 31 | ADH1C\|AKT2\|CALM1\|CALM2\|CALM3\|CCNT1\|CRABP2\|EGFR\|ESR1\|ESR2\|FABP5\|HSP90AA1\|HSP90AB1\|IGF1R\|MMP2\|MMP3\|MMP9\|NOS3\|PDPK1\|PGR\|PIK3R1\|MAPK1\|RARA\|RARB\|RARG\|RXRA\|RXRB\|SRC\|NR1H2\|KAT2B\|NR1H3 |
|  | R-HSA-5663202 | Diseases of signal transduction by growth factor receptors and second messengers | 1E-27 | 13 | 19 | 35 | AKT2\|ATIC\|BCL2L1\|BRAF\|CALM1\|CALM2\|CALM3\|CSK\|DUSP6\|EGFR\|ESR1\|ESR2\|FGFR1\|FGFR2\|GRB2\|GSK3B\|HSP90AA1\|JAK2\|KDR\|KIT\|LCK\|MDM2\|MET\|PDPK1\|PIM1\|PIK3R1\|PPP1CC\|MAPK1\|MAP2K1\|PTPN11\|SRC\|STAT1\|ADAM17\|TGFBR1\|KAT2B |
|  | R-HSA-449147 | Signaling by Interleukins | 1E-27 | 12 | 19 | 36 | BCL2L1\|CA1\|CASP1\|CASP3\|MAPK14\|CTSG\|DUSP6\|GRB2\|HCK\|HMOX1\|HSPA8\|HSP90AA1\|IL2\|JAK2\|JAK3\|LCK\|LCN2\|MAOA\|MMP1\|MMP2\|MMP3\|MMP9\|NOS2\|PIM1\|PIK3R1\|PPIA\|MAPK1\|MAPK8\|MAPK10\|MAP2K1\|PTPN11\|RORA\|SOD2\|STAT1\|SYK\|MAPKAPK2 |
|  | R-HSA-1280215 | Cytokine Signaling in Immune system | 1E-26 | 8.7 | 17 | 41 | AKT2\|BCL2L1\|CA1\|CASP1\|CASP3\|MAPK14\|CSK\|CTSG\|DUSP6\|EIF4E\|GRB2\|HCK\|HMOX1\|HSPA8\|HSP90AA1\|IL2\|JAK2\|JAK3\|LCK\|LCN2\|MAOA\|MMP1\|MMP2\|MMP3\|MMP9\|NOS2\|PIM1\|PIK3R1\|PPIA\|MAPK1\|MAPK8\|MAPK10\|MAP2K1\|PTPN1\|PTPN11\|RORA\|SOD2\|STAT1\|SYK\|ADAM17\|MAPKAPK2 |
|  | R-HSA-4090294 | SUMOylation of intracellular receptors | 1E-25 | 77 | 34 | 15 | AR\|ESR1\|NR3C1\|NR3C2\|PGR\|PPARA\|PPARG\|RARA\|RORA\|RXRA\|NR1H2\|VDR\|NR1I2\|NR1H4\|NR1H3 |
|  | R-HSA-109582 | Hemostasis | 1E-23 | 9 | 16 | 36 | ABL1\|ALB\|ANXA5\|CALM1\|CALM2\|CALM3\|CDK2\|MAPK14\|CSK\|CFD\|F2\|F10\|GRB2\|IGF1\|ITGAL\|JAK2\|LCK\|MMP1\|NOS2\|NOS3\|PDPK1\|SERPINA1\|PIK3CG\|PIK3R1\|PPIA\|PRKCQ\|MAPK1\|PTPN1\|PTPN11\|SELE\|SRC\|SYK\|TEK\|TGFB2\|PDE5A\|PROCR |
|  | R-HSA-6798695 | Neutrophil degranulation | 1E-21 | 10 | 16 | 31 | ARG1\|BST1\|CHIT1\|MAPK14\|CTSB\|CTSG\|CTSS\|CFD\|ELANE\|FABP5\|GPI\|GSTP1\|HEXB\|HSPA8\|HSP90AA1\|HSP90AB1\|IMPDH2\|ITGAL\|LCN2\|LGALS3\|LTA4H\|LYZ\|MMP8\|MMP9\|SERPINA1\|PPIA\|MAPK1\|RNASE2\|RNASE3\|S100A9\|TTR |
|  | R-HSA-9009391 | Extra-nuclear estrogen signaling | 1E-21 | 34 | 23 | 17 | AKT2\|CALM1\|CALM2\|CALM3\|EGFR\|ESR1\|ESR2\|HSP90AA1\|IGF1R\|MMP2\|MMP3\|MMP9\|NOS3\|PDPK1\|PIK3R1\|MAPK1\|SRC |
|  | R-HSA-5684996 | MAPK1/MAPK3 signaling | 1E-20 | 14 | 17 | 25 | BCL2L1\|BRAF\|CALM1\|CALM2\|CALM3\|CSK\|DUSP6\|EGFR\|FGFR1\|FGFR2\|GRB2\|IL2\|JAK2\|JAK3\|KIT\|MET\|PIK3R1\|PPP1CC\|PRKCQ\|MAPK1\|MAP2K1\|PTPN11\|MAPK12\|SRC\|TEK |
|  | R-HSA-76002 | Platelet activation, signaling and aggregation | 1E-20 | 14 | 17 | 24 | ALB\|ANXA5\|CALM1\|CALM2\|CALM3\|MAPK14\|CSK\|CFD\|F2\|GRB2\|IGF1\|LCK\|PDPK1\|SERPINA1\|PIK3CG\|PIK3R1\|PPIA\|PRKCQ\|MAPK1\|PTPN1\|PTPN11\|SRC\|SYK\|TGFB2 |
|  | R-HSA-5673001 | RAF/MAP kinase cascade | 1E-19 | 13 | 17 | 24 | BCL2L1\|BRAF\|CALM1\|CALM2\|CALM3\|CSK\|DUSP6\|EGFR\|FGFR1\|FGFR2\|GRB2\|IL2\|JAK2\|JAK3\|KIT\|MET\|PIK3R1\|PPP1CC\|PRKCQ\|MAPK1\|MAP2K1\|MAPK12\|SRC\|TEK |
|  | R-HSA-5683057 | MAPK family signaling cascades | 1E-19 | 12 | 16 | 25 | BCL2L1\|BRAF\|CALM1\|CALM2\|CALM3\|CSK\|DUSP6\|EGFR\|FGFR1\|FGFR2\|GRB2\|IL2\|JAK2\|JAK3\|KIT\|MET\|PIK3R1\|PPP1CC\|PRKCQ\|MAPK1\|MAP2K1\|PTPN11\|MAPK12\|SRC\|TEK |
|  | R-HSA-6785807 | Interleukin-4 and Interleukin-13 signaling | 1E-18 | 24 | 20 | 17 | BCL2L1\|HMOX1\|HSPA8\|HSP90AA1\|JAK2\|JAK3\|LCN2\|MAOA\|MMP1\|MMP2\|MMP3\|MMP9\|NOS2\|PIM1\|PIK3R1\|RORA\|STAT1 |
|  | R-HSA-8939211 | ESR-mediated signaling | 1E-18 | 15 | 16 | 21 | AKT2\|CALM1\|CALM2\|CALM3\|CCNT1\|EGFR\|ESR1\|ESR2\|HSP90AA1\|HSP90AB1\|IGF1R\|MMP2\|MMP3\|MMP9\|NOS3\|PDPK1\|PGR\|PIK3R1\|MAPK1\|SRC\|KAT2B |
|  | R-HSA-9006925 | Intracellular signaling by second messengers | 1E-17 | 12 | 15 | 23 | AKT2\|XIAP\|CALM1\|CALM2\|CALM3\|EGFR\|ESR1\|ESR2\|FGFR1\|FGFR2\|GRB2\|GSK3B\|INSR\|KIT\|LCK\|MDM2\|MET\|PDPK1\|PIK3R1\|PPARG\|MAPK1\|PTPN11\|SRC |
|  | R-HSA-2219528 | PI3K/AKT Signaling in Cancer | 1E-17 | 24 | 19 | 16 | AKT2\|EGFR\|ESR1\|ESR2\|FGFR1\|FGFR2\|GRB2\|GSK3B\|KIT\|LCK\|MDM2\|MET\|PDPK1\|PIK3R1\|PTPN11\|SRC |
|  | R-HSA-556833 | Metabolism of lipids | 1E-17 | 6.7 | 13 | 32 | ALB\|AKR1B1\|APOA2\|STS\|BCHE\|CYP2C9\|DPEP1\|ESRRA\|FABP4\|FABP5\|GC\|HEXB\|HMGCR\|HSD11B1\|LTA4H\|PIK3CG\|PIK3R1\|PLA2G2A\|PPARA\|PPARG\|PPP1CC\|RORA\|RXRA\|RXRB\|SULT2A1\|NR1H2\|VDR\|PLA2G10\|MAPKAPK2\|NR1H4\|NR1H3\|HPGDS |
|  | R-HSA-1592389 | Activation of Matrix Metalloproteinases | 1E-16 | 52 | 23 | 11 | CMA1\|CTSG\|CTSK\|ELANE\|MMP1\|MMP2\|MMP3\|MMP8\|MMP9\|MMP13\|MMP16 |
|  | R-HSA-3108232 | SUMO E3 ligases SUMOylate target proteins | 1E-16 | 15 | 16 | 18 | PARP1\|AR\|ESR1\|NR3C1\|MDM2\|NR3C2\|PGR\|PPARA\|PPARG\|RARA\|RORA\|RXRA\|AURKA\|NR1H2\|VDR\|NR1I2\|NR1H4\|NR1H3 |
|  | R-HSA-2990846 | SUMOylation | 1E-15 | 15 | 15 | 18 | PARP1\|AR\|ESR1\|NR3C1\|MDM2\|NR3C2\|PGR\|PPARA\|PPARG\|RARA\|RORA\|RXRA\|AURKA\|NR1H2\|VDR\|NR1I2\|NR1H4\|NR1H3 |
|  | R-HSA-1474244 | Extracellular matrix organization | 1E-15 | 11 | 14 | 21 | BMP2\|CASP3\|CMA1\|CTSB\|CTSG\|CTSK\|CTSS\|ELANE\|ITGAL\|KDR\|MMP1\|MMP2\|MMP3\|MMP8\|MMP9\|MMP12\|MMP13\|MMP16\|ADAM17\|TGFB2\|TTR |
|  | R-HSA-199418 | Negative regulation of the PI3K/AKT network | 1E-15 | 21 | 17 | 15 | AKT2\|EGFR\|ESR1\|ESR2\|FGFR1\|FGFR2\|GRB2\|INSR\|KIT\|LCK\|MET\|PIK3R1\|MAPK1\|PTPN11\|SRC |
|  | R-HSA-1257604 | PIP3 activates AKT signaling | 1E-15 | 12 | 14 | 20 | AKT2\|XIAP\|EGFR\|ESR1\|ESR2\|FGFR1\|FGFR2\|GRB2\|GSK3B\|INSR\|KIT\|LCK\|MDM2\|MET\|PDPK1\|PIK3R1\|PPARG\|MAPK1\|PTPN11\|SRC |
|  | R-HSA-1474228 | Degradation of the extracellular matrix | 1E-15 | 18 | 16 | 16 | CASP3\|CMA1\|CTSB\|CTSG\|CTSK\|CTSS\|ELANE\|MMP1\|MMP2\|MMP3\|MMP8\|MMP9\|MMP12\|MMP13\|MMP16\|ADAM17 |
|  | R-HSA-9658195 | Leishmania infection | 1E-14 | 12 | 14 | 19 | ABL1\|BTK\|CALM1\|CALM2\|CALM3\|CASP1\|MAPK14\|CTSG\|DPEP1\|GRB2\|HCK\|HMOX1\|HSP90AB1\|MAPK1\|MAPK8\|SRC\|SYK\|ADAM17\|WAS |
|  | R-HSA-6811558 | PI5P, PP2A and IER3 Regulate PI3K/AKT Signaling | 1E-14 | 20 | 16 | 14 | EGFR\|ESR1\|ESR2\|FGFR1\|FGFR2\|GRB2\|INSR\|KIT\|LCK\|MET\|PIK3R1\|MAPK1\|PTPN11\|SRC |
|  | R-HSA-194138 | Signaling by VEGF | 1E-14 | 20 | 16 | 14 | AKT2\|CALM1\|CALM2\|CALM3\|MAPK14\|HSP90AA1\|KDR\|NOS3\|PDPK1\|PGF\|PIK3R1\|MAPK12\|SRC\|MAPKAPK2 |
|  | R-HSA-4420097 | VEGFA-VEGFR2 Pathway | 1E-13 | 20 | 16 | 13 | AKT2\|CALM1\|CALM2\|CALM3\|MAPK14\|HSP90AA1\|KDR\|NOS3\|PDPK1\|PIK3R1\|MAPK12\|SRC\|MAPKAPK2 |
|  | R-HSA-2219530 | Constitutive Signaling by Aberrant PI3K in Cancer | 1E-13 | 24 | 16 | 12 | EGFR\|ESR1\|ESR2\|FGFR1\|FGFR2\|GRB2\|KIT\|LCK\|MET\|PIK3R1\|PTPN11\|SRC |
|  | R-HSA-1433557 | Signaling by SCF-KIT | 1E-13 | 36 | 19 | 10 | CMA1\|GRB2\|JAK2\|KIT\|LCK\|MMP9\|PIK3R1\|PTPN11\|SRC\|STAT1 |
|  | R-HSA-1442490 | Collagen degradation | 1E-12 | 27 | 17 | 11 | CTSB\|CTSK\|ELANE\|MMP1\|MMP2\|MMP3\|MMP8\|MMP9\|MMP12\|MMP13\|ADAM17 |
|  | R-HSA-8878166 | Transcriptional regulation by RUNX2 | 1E-12 | 17 | 14 | 13 | ABL1\|AKT2\|AR\|BMP2\|ESR1\|ESRRA\|NR3C1\|GSK3B\|LGALS3\|MMP13\|MAPK1\|SRC\|STAT1 |
|  | R-HSA-2262752 | Cellular responses to stress | 1E-12 | 5.3 | 9.9 | 27 | AKT2\|ALB\|AR\|BLVRB\|CCNA2\|CDK2\|MAPK14\|NQO1\|NR3C1\|GSK3B\|GSR\|GSTP1\|HMOX1\|HSPA8\|HSP90AA1\|HSP90AB1\|MDM2\|NR3C2\|PGR\|PPARA\|MAPK1\|MAPK8\|MAPK10\|RORA\|RXRA\|SOD2\|MAPKAPK2 |
|  | R-HSA-8953897 | Cellular responses to stimuli | 1E-12 | 5.2 | 9.8 | 27 | AKT2\|ALB\|AR\|BLVRB\|CCNA2\|CDK2\|MAPK14\|NQO1\|NR3C1\|GSK3B\|GSR\|GSTP1\|HMOX1\|HSPA8\|HSP90AA1\|HSP90AB1\|MDM2\|NR3C2\|PGR\|PPARA\|MAPK1\|MAPK8\|MAPK10\|RORA\|RXRA\|SOD2\|MAPKAPK2 |
|  | R-HSA-9675108 | Nervous system development | 1E-11 | 6.2 | 10 | 23 | ABL1\|MAPK14\|EGFR\|EPHB4\|FGFR1\|GRB2\|GSK3B\|HMGCR\|HSPA8\|HSP90AA1\|HSP90AB1\|MET\|MMP2\|MMP9\|PIK3R1\|PRKCQ\|MAPK1\|MAPK8\|MAP2K1\|PTPN11\|MAPK12\|SRC\|CDK5R1 |
|  | R-HSA-166520 | Signaling by NTRKs | 1E-11 | 15 | 13 | 13 | BRAF\|MAPK14\|DUSP6\|GRB2\|PIK3R1\|MAPK1\|MAP2K1\|PTPN11\|MAPK12\|SRC\|TPH1\|CDK5R1\|MAPKAPK2 |
|  | R-HSA-6802957 | Oncogenic MAPK signaling | 1E-11 | 20 | 14 | 11 | BRAF\|CALM1\|CALM2\|CALM3\|CSK\|DUSP6\|JAK2\|PPP1CC\|MAPK1\|MAP2K1\|SRC |
|  | R-HSA-9656223 | Signaling by RAF1 mutants | 1E-11 | 32 | 17 | 9 | BRAF\|CALM1\|CALM2\|CALM3\|CSK\|JAK2\|MAPK1\|MAP2K1\|SRC |
|  | R-HSA-2029480 | Fcgamma receptor (FCGR) dependent phagocytosis | 1E-11 | 20 | 14 | 11 | ABL1\|BTK\|GRB2\|HCK\|HSP90AA1\|HSP90AB1\|PIK3R1\|MAPK1\|SRC\|SYK\|WAS |
|  | R-HSA-422475 | Axon guidance | 1E-11 | 6.2 | 9.9 | 22 | ABL1\|MAPK14\|EGFR\|EPHB4\|FGFR1\|GRB2\|GSK3B\|HSPA8\|HSP90AA1\|HSP90AB1\|MET\|MMP2\|MMP9\|PIK3R1\|PRKCQ\|MAPK1\|MAPK8\|MAP2K1\|PTPN11\|MAPK12\|SRC\|CDK5R1 |
|  | R-HSA-187037 | Signaling by NTRK1 (TRKA) | 1E-11 | 16 | 13 | 12 | BRAF\|MAPK14\|DUSP6\|GRB2\|PIK3R1\|MAPK1\|MAP2K1\|MAPK12\|SRC\|TPH1\|CDK5R1\|MAPKAPK2 |
|  | R-HSA-1989781 | PPARA activates gene expression | 1E-11 | 16 | 13 | 12 | APOA2\|ESRRA\|HMGCR\|PPARA\|PPARG\|RORA\|RXRA\|RXRB\|SULT2A1\|NR1H2\|NR1H4\|NR1H3 |
|  | R-HSA-6802946 | Signaling by moderate kinase activity BRAF mutants | 1E-11 | 30 | 16 | 9 | BRAF\|CALM1\|CALM2\|CALM3\|CSK\|JAK2\|MAPK1\|MAP2K1\|SRC |
|  | R-HSA-6802955 | Paradoxical activation of RAF signaling by kinase inactive BRAF | 1E-11 | 30 | 16 | 9 | BRAF\|CALM1\|CALM2\|CALM3\|CSK\|JAK2\|MAPK1\|MAP2K1\|SRC |
|  | R-HSA-6802949 | Signaling by RAS mutants | 1E-11 | 30 | 16 | 9 | BRAF\|CALM1\|CALM2\|CALM3\|CSK\|JAK2\|MAPK1\|MAP2K1\|SRC |
|  | R-HSA-9649948 | Signaling downstream of RAS mutants | 1E-11 | 30 | 16 | 9 | BRAF\|CALM1\|CALM2\|CALM3\|CSK\|JAK2\|MAPK1\|MAP2K1\|SRC |
|  | R-HSA-400206 | Regulation of lipid metabolism by PPARalpha | 1E-11 | 16 | 13 | 12 | APOA2\|ESRRA\|HMGCR\|PPARA\|PPARG\|RORA\|RXRA\|RXRB\|SULT2A1\|NR1H2\|NR1H4\|NR1H3 |
|  | R-HSA-9670439 | Signaling by phosphorylated juxtamembrane, extracellular and kinase domain KIT mutants | 1E-11 | 54 | 19 | 7 | GRB2\|JAK2\|KIT\|LCK\|PIK3R1\|SRC\|STAT1 |
|  | R-HSA-9669938 | Signaling by KIT in disease | 1E-11 | 54 | 19 | 7 | GRB2\|JAK2\|KIT\|LCK\|PIK3R1\|SRC\|STAT1 |
|  | R-HSA-187687 | Signalling to ERKs | 1E-10 | 36 | 17 | 8 | BRAF\|MAPK14\|GRB2\|MAPK1\|MAP2K1\|MAPK12\|SRC\|MAPKAPK2 |
|  | R-HSA-2428928 | IRS-related events triggered by IGF1R | 1E-10 | 27 | 15 | 9 | AKT2\|FGFR1\|FGFR2\|GRB2\|IGF1\|IGF1R\|PDPK1\|PIK3R1\|PTPN11 |
|  | R-HSA-2428924 | IGF1R signaling cascade | 1E-10 | 26 | 15 | 9 | AKT2\|FGFR1\|FGFR2\|GRB2\|IGF1\|IGF1R\|PDPK1\|PIK3R1\|PTPN11 |
|  | R-HSA-1474151 | Tetrahydrobiopterin (BH4) synthesis, recycling, salvage and regulation | 1E-10 | 77 | 21 | 6 | CALM1\|CALM2\|CALM3\|DHFR\|HSP90AA1\|NOS3 |
|  | R-HSA-2404192 | Signaling by Type 1 Insulin-like Growth Factor 1 Receptor (IGF1R) | 1E-10 | 26 | 15 | 9 | AKT2\|FGFR1\|FGFR2\|GRB2\|IGF1\|IGF1R\|PDPK1\|PIK3R1\|PTPN11 |
|  | R-HSA-74751 | Insulin receptor signalling cascade | 1E-10 | 26 | 15 | 9 | AKT2\|FGFR1\|FGFR2\|GRB2\|INSR\|PDPK1\|PIK3R1\|MAPK1\|PTPN11 |
|  | R-HSA-5673000 | RAF activation | 1E-10 | 34 | 16 | 8 | BRAF\|CALM1\|CALM2\|CALM3\|JAK2\|PPP1CC\|MAP2K1\|SRC |
|  | R-HSA-2454202 | Fc epsilon receptor (FCERI) signaling | 1E-10 | 14 | 12 | 12 | BTK\|CALM1\|CALM2\|CALM3\|GRB2\|PDPK1\|PIK3R1\|PRKCQ\|MAPK1\|MAPK8\|MAPK10\|SYK |
|  | R-HSA-168898 | Toll-like Receptor Cascades | 1E-10 | 12 | 12 | 13 | BTK\|MAPK14\|CTSB\|CTSK\|CTSS\|DUSP6\|MAPK1\|MAPK8\|MAPK10\|MAP2K1\|PTPN11\|S100A9\|MAPKAPK2 |
