# Supplementary material for: Exploring the mechanism of Celastrol in the treatment of rheumatoid arthritis based on systems pharmacology and multi-omics
Source: Sci Rep. 2024 Jan 18;14:1604. doi: 10.1038/s41598-023-48248-5 (PMC10796403; doi:10.1038/s41598-023-48248-5)
Supplement: Supplementary file 3 — Supplementary Table S2. [file 41598_2023_48248_MOESM3_ESM.docx]

Table S2 Enrichment analysis of all differentially expressed genes

| Category | GO | Description | Pvalue | Enrichment | Z-score | Counts | Genes |
| --- | --- | --- | --- | --- | --- | --- | --- |
| GO Biological Processes | GO:0098660 | inorganic ion transmembrane transport | 1E-14 | 2.5 | 8.9 | 83 | FASLG\|SHROOM2\|ATP1A4\|ATP1B2\|CACNA1B\|CACNA1E\|CHRNA7\|CCN2\|GABRR2\|GRIN2C\|KCNA1\|KCNC3\|KCND2\|KCNJ4\|KCNJ6\|KCNN2\|KCNN3\|KCNQ1\|KCNQ2\|KCNS1\|KCNS2\|KEL\|LCK\|ATP6\|ATP8\|COX1\|COX2\|COX3\|CYTB\|RYR1\|RYR2\|SCN1A\|SCN2A\|SCN4A\|SCN10A\|CCL3\|SLC1A7\|SLC3A2\|SLC4A1\|SLC5A5\|SLC6A3\|SLC6A4\|SLC6A7\|SLC6A12\|SLC6A13\|SLC8A2\|SLC8A3\|SLC12A1\|UCP1\|SLC30A3\|GLRA3\|KCNK5\|CACNA1H\|HCN4\|KCNK7\|KCNH3\|KCNE4\|SLC24A2\|KCNH5\|TRPM5\|RHCG\|KCNK10\|BEST2\|TRPV6\|ASIC4\|SLC30A10\|GABRQ\|RHBG\|JPH3\|ANO3\|SLC12A8\|OTOP2\|SLC26A9\|SLC5A10\|BEST3\|ATP6V0E2\|PKD1L1\|KCNG3\|ANO5\|ATP6V0D2\|SCARA5\|PKD1L3\|OTOP3 |
|  | GO:0043269 | regulation of ion transport | 1E-12 | 2.4 | 8.4 | 82 | ACTN2\|ADRA2B\|ATP1B2\|CA2\|CACNA1B\|CACNA1E\|CHRM1\|CHRNA4\|CNR1\|NKX2-5\|EPHB2\|F2\|GDNF\|GEM\|HES1\|HSPA2\|CXCL10\|KCNA1\|KCNC3\|KCND2\|KCNJ4\|KCNJ6\|KCNN2\|KCNQ1\|KCNQ2\|KCNS1\|KCNS2\|KEL\|CXCL9\|OPRK1\|P2RX5\|P2RY1\|P2RY6\|ABCB1\|PIK3CG\|PTAFR\|PTGS2\|PTPN6\|RASGRF1\|RGS2\|RGS4\|RRAD\|RYR2\|SCN1A\|SCN2A\|SCN4A\|SCN10A\|CCL3\|SLC6A4\|SPINK1\|STC1\|TNF\|VIP\|CXCR4\|PLA2G10\|KCNK5\|GALR2\|CACNA1H\|CHRNA6\|HAP1\|HCN4\|KCNK7\|ARC\|KCNH3\|KCNE4\|TSPAN13\|KCNH5\|TRPM5\|GAL\|KCNK10\|SLC30A10\|CABP4\|JPH3\|WNK2\|JSRP1\|BEST3\|GSG1L\|SYT6\|SIK1\|KCNG3\|NKAIN3\|STAC2 |
|  | GO:0098662 | inorganic cation transmembrane transport | 1E-12 | 2.5 | 8.5 | 73 | FASLG\|SHROOM2\|ATP1A4\|ATP1B2\|CACNA1B\|CACNA1E\|CHRNA7\|CCN2\|GRIN2C\|KCNA1\|KCNC3\|KCND2\|KCNJ4\|KCNJ6\|KCNN2\|KCNN3\|KCNQ1\|KCNQ2\|KCNS1\|KCNS2\|KEL\|LCK\|ATP6\|ATP8\|COX1\|COX2\|COX3\|CYTB\|RYR1\|RYR2\|SCN1A\|SCN2A\|SCN4A\|SCN10A\|CCL3\|SLC3A2\|SLC5A5\|SLC6A3\|SLC6A4\|SLC6A7\|SLC6A12\|SLC6A13\|SLC8A2\|SLC8A3\|SLC12A1\|UCP1\|SLC30A3\|KCNK5\|CACNA1H\|HCN4\|KCNK7\|KCNH3\|KCNE4\|SLC24A2\|KCNH5\|TRPM5\|RHCG\|KCNK10\|TRPV6\|ASIC4\|SLC30A10\|RHBG\|JPH3\|SLC12A8\|OTOP2\|SLC5A10\|ATP6V0E2\|PKD1L1\|KCNG3\|ATP6V0D2\|SCARA5\|PKD1L3\|OTOP3 |
|  | GO:0098655 | cation transmembrane transport | 1E-12 | 2.4 | 8.3 | 78 | FASLG\|SHROOM2\|ATP1A4\|ATP1B2\|CACNA1B\|CACNA1E\|CHRNA7\|CNGA1\|CCN2\|GRIN2C\|KCNA1\|KCNC3\|KCND2\|KCNJ4\|KCNJ6\|KCNN2\|KCNN3\|KCNQ1\|KCNQ2\|KCNS1\|KCNS2\|KEL\|LCK\|ATP6\|ATP8\|COX1\|COX2\|COX3\|CYTB\|P2RX5\|RYR1\|RYR2\|SCN1A\|SCN2A\|SCN4A\|SCN10A\|CCL3\|SLC3A2\|SLC5A5\|SLC6A3\|SLC6A4\|SLC6A7\|SLC6A12\|SLC6A13\|SLC8A2\|SLC8A3\|SLC12A1\|UCP1\|SLC30A3\|SLC7A5\|KCNK5\|CACNA1H\|HCN4\|KCNK7\|KCNH3\|KCNE4\|SLC24A2\|KCNH5\|TRPM5\|RHCG\|KCNK10\|TRPV6\|ASIC4\|SLC30A10\|RHBG\|JPH3\|SLC12A8\|SLC7A3\|SLC22A16\|OTOP2\|SLC5A10\|ATP6V0E2\|PKD1L1\|KCNG3\|ATP6V0D2\|SCARA5\|PKD1L3\|OTOP3 |
|  | GO:0007420 | brain development | 1E-12 | 2.3 | 8.1 | 84 | SHROOM2\|ASCL1\|ATP1B2\|AVPR2\|BMP2\|BMP7\|CTNNA2\|CX3CR1\|DLX2\|EFNA2\|CELSR2\|EGR2\|EN2\|EPHB2\|FOXG1\|FOXJ1\|FUT1\|GATA2\|GBX2\|GDF10\|GRIN2C\|HES1\|HSPA5\|DNAJB1\|INHBA\|ITGAM\|KCNA1\|LHX1\|MT3\|COX1\|NEFL\|NEUROD1\|NR4A2\|PAX6\|PFKFB3\|PITX3\|POU3F1\|POU3F2\|PROX1\|RGS2\|RGS4\|ROBO2\|RORA\|SALL1\|SHH\|SIX3\|SLC2A1\|SLC6A3\|SLC6A4\|SOX1\|SOX3\|SRD5A2\|SSTR2\|SSTR3\|ZIC2\|CXCR4\|EOMES\|UNC5C\|SPHK1\|HAP1\|BAG3\|AATK\|MAFB\|OLIG2\|BAIAP2\|SEMA6B\|ZNF365\|MYO16\|TTLL1\|CNTNAP2\|LHX6\|DLL1\|PCDH19\|BCAN\|DMRTA2\|BCL11B\|TMEM108\|MFSD2A\|KNDC1\|DMBX1\|GDF7\|ASPM\|NANOS1\|HMX3 |
|  | GO:0003002 | regionalization | 1E-12 | 3.1 | 8.6 | 50 | ASCL1\|BMP2\|BMPR1B\|NKX2-5\|DLX2\|CELSR2\|EGR2\|FOXG1\|FOXF1\|FOXJ1\|GBX2\|HHEX\|FOXA1\|HOXB9\|HOXC11\|HOXC13\|HES1\|ITGAM\|LHX1\|LMX1B\|NEUROD1\|NKX3-1\|PAX6\|PCDH8\|SHH\|SIX3\|SNAI1\|SOX1\|TBX1\|EOMES\|MAFB\|DLL3\|ARC\|COBL\|HEY1\|DLL1\|DLL4\|HES2\|HES6\|BARX1\|HES4\|DMRT3\|DMRTA2\|GREM2\|HES7\|PIFO\|RIPPLY2\|TDRD5\|PKD1L1\|NRARP |
|  | GO:0007389 | pattern specification process | 1E-11 | 2.7 | 8.2 | 59 | ASCL1\|BMP2\|BMP7\|BMPR1B\|NKX2-5\|DLX2\|CELSR2\|EGR2\|FOXG1\|FOXF1\|FOXJ1\|GBX2\|HHEX\|FOXA1\|HOXB9\|HOXC11\|HOXC13\|HES1\|IHH\|ITGAM\|LHX1\|LMX1B\|NEUROD1\|NKX3-1\|PAX6\|PCDH8\|SHH\|SIX3\|SNAI1\|SOX1\|STC1\|TBX1\|WNT6\|EOMES\|HAND1\|MAFB\|DLL3\|ARC\|COBL\|HEY1\|DLL1\|IRX4\|ASB2\|SOX18\|DLL4\|HES2\|HES6\|BARX1\|HES4\|DMRT3\|DMRTA2\|GREM2\|HES7\|MMP21\|PIFO\|RIPPLY2\|TDRD5\|PKD1L1\|NRARP |
|  | GO:0070848 | response to growth factor | 1E-11 | 2.6 | 8.1 | 64 | ANGPT2\|FASLG\|ASCL1\|BMP2\|BMP7\|BMPR1B\|CALCA\|RUNX3\|CCNA2\|CDH5\|COL2A1\|CCN2\|CX3CR1\|EGR3\|FBN1\|FGFR3\|FGFR4\|FLT1\|FLT3\|FLT4\|FOS\|GCNT2\|GDF9\|GDF10\|HAS1\|NR4A1\|HES1\|HSPA5\|HSPB1\|LHX1\|MT1G\|NGFR\|P2RY1\|PAX9\|PDE2A\|PDGFA\|PITX3\|SPI1\|TAC1\|TBX1\|VIL1\|GDF5\|FGF17\|FGF16\|SPHK1\|HAP1\|ONECUT2\|GDF15\|FGF19\|SPRY2\|BAIAP2\|STMN2\|BAMBI\|MXRA5\|ANKRD1\|ERRFI1\|DLL4\|TMEM108\|PDGFD\|DCSTAMP\|PLK5\|TRIM71\|GDF7\|SCX |
|  | GO:0007423 | sensory organ development | 1E-11 | 2.5 | 7.9 | 67 | ACHE\|FASLG\|SHROOM2\|AQP5\|ASCL1\|ASCL2\|BMP2\|BMP7\|BMPR1B\|FOXL2\|CCNA2\|CEBPA\|COL2A1\|CRYAB\|CRYBA2\|CYP1A1\|DIO3\|DSCAM\|EPHB2\|ESRRB\|FBN1\|FOXG1\|FLT1\|GATA2\|GBX2\|HOXC13\|HES1\|IHH\|INHBA\|KCNQ1\|LHX1\|MYO7A\|NEUROD1\|PAX6\|PDGFA\|PITX3\|PLS1\|PROX1\|RP1\|SHH\|SIX3\|SLC6A3\|SOX1\|SOX3\|SOX11\|TBX1\|WNT6\|MAFB\|NR2E3\|SPRY2\|MERTK\|CECR2\|DLL1\|SOX8\|CYTL1\|CABP4\|BCL11B\|ADGRV1\|MFSD2A\|NKD1\|IGFN1\|USH1G\|TMEM132E\|ADAMTS18\|OPN5\|TMIE\|HMX3 |
|  | GO:0045664 | regulation of neuron differentiation | 1E-11 | 3.7 | 8.6 | 35 | ADRA2B\|ASCL1\|BMP2\|BMP7\|NKX2-5\|DLX2\|DTX1\|FOXG1\|GATA2\|FOXA1\|HES1\|NAP1L2\|NEUROD1\|NKX6-1\|PAX6\|PROX1\|SH3GL3\|SHH\|SIX3\|SLC6A4\|SOX3\|SOX11\|TP73\|GDF5\|GPR37L1\|OLIG2\|HEY1\|DLL1\|SOX8\|CASZ1\|DISP3\|BCL11B\|GDF7\|ASPM\|VWC2 |
|  | GO:0045596 | negative regulation of cell differentiation | 1E-11 | 2.3 | 7.7 | 78 | ASCL1\|ASCL2\|BMP2\|BMP7\|RUNX3\|NKX2-5\|DLX2\|DTX1\|EFNA1\|EPHB2\|EREG\|ESRRB\|F2\|FBN1\|FOXG1\|FOXJ1\|GATA2\|GDF10\|HLA-G\|FOXA1\|HES1\|IFRD1\|IGF1\|IHH\|INHBA\|CXCL10\|MT3\|NKX6-1\|NPPC\|PAX6\|PITX3\|PROX1\|RGS2\|RGS4\|RORA\|CCL3\|SHH\|SIX3\|SLC6A4\|SNAI1\|SOX3\|SOX11\|SPP1\|TBX1\|TNF\|TP73\|GDF5\|H4C4\|H4C8\|H4C5\|GPR37L1\|GPR55\|MAFB\|OLIG2\|TRIB1\|LRRC17\|SPRY2\|SEMA6B\|DLL3\|FGL2\|FSTL4\|HEY1\|BAMBI\|NMRK2\|DLL1\|TBX21\|SOX8\|TENT5C\|DISP3\|MIXL1\|ADGRV1\|MYOCD\|ASPM\|PRAMEF17\|NRARP\|TRIM72\|WEE2\|PRAMEF19 |
|  | GO:0048568 | embryonic organ development | 1E-11 | 2.7 | 7.9 | 57 | ASCL2\|BMP7\|FOXL2\|CEBPA\|COL2A1\|NKX2-5\|DLX2\|EFNA1\|EN2\|EPHB2\|FBN1\|FOXG1\|FOXF1\|FOXE1\|GATA2\|GBX2\|GDNF\|HOXB9\|HOXC11\|HES1\|IHH\|KCNQ1\|LHX1\|KITLG\|MYO7A\|NEUROD1\|PAX6\|PDGFA\|PLS1\|PROX1\|RYR2\|SALL1\|SHH\|SIX3\|SNAI1\|SOX11\|ST14\|TBX1\|TNF\|TPO\|EOMES\|GCM1\|HAND1\|MAFB\|SPRY2\|PLK4\|COBL\|HEY1\|DLL1\|ASB2\|SOX18\|VASH2\|USH1G\|PIFO\|TMIE\|HMX3\|TUBB2B |
|  | GO:0030001 | metal ion transport | 1E-11 | 2.4 | 7.7 | 71 | FASLG\|SHROOM2\|ATP1A4\|ATP1B2\|CACNA1B\|CACNA1E\|CHRNA4\|CHRNA7\|CCN2\|FKBP4\|GRIN2C\|KCNA1\|KCNC3\|KCND2\|KCNJ4\|KCNJ6\|KCNN2\|KCNN3\|KCNQ1\|KCNQ2\|KCNS1\|KCNS2\|KEL\|LCK\|MT3\|PRKCB\|RYR1\|RYR2\|SCN1A\|SCN2A\|SCN4A\|SCN10A\|CCL3\|CCL8\|SLC3A2\|SLC5A5\|SLC6A3\|SLC6A4\|SLC6A7\|SLC6A12\|SLC6A13\|SLC8A2\|SLC8A3\|SLC12A1\|SLC30A3\|KCNK5\|CACNA1H\|HCN4\|KCNK7\|RAMP1\|KCNH3\|KCNE4\|SLC24A2\|KCNH5\|TRPM5\|KCNK10\|TRPV6\|ASIC4\|SLC30A10\|JPH3\|STEAP4\|SLC12A8\|PLCZ1\|SLC5A10\|PKD1L1\|KCNG3\|SLC5A9\|SLC10A4\|SLC13A5\|SCARA5\|PKD1L3 |
|  | GO:0050767 | regulation of neurogenesis | 1E-11 | 2.8 | 8 | 51 | ASCL1\|ASCL2\|ASPA\|BMP2\|BMP7\|CX3CR1\|DLX2\|DSCAM\|EGR2\|EPHB2\|F2\|FOXG1\|HES1\|IFRD1\|LTA\|MT3\|NEFL\|NKX6-1\|PAX6\|PITX3\|PROX1\|ROBO2\|SHH\|SOX11\|SPP1\|TNF\|TP73\|CXCR4\|SLC7A5\|HAP1\|GPR37L1\|OLIG2\|BAIAP2\|SEMA6B\|DLL3\|IL1RAPL1\|ZNF365\|FSTL4\|CUX2\|HEY1\|DLL1\|SOX8\|DLL4\|HES2\|HES6\|ISLR2\|DMRTA2\|HES7\|IL34\|ASPM\|RASSF10 |
|  | GO:0042391 | regulation of membrane potential | 1E-10 | 2.7 | 7.8 | 55 | ACTN2\|ATP1A4\|ATP1B2\|CHRM1\|CHRNA2\|CHRNA4\|CHRNA5\|CHRNA7\|CHRNB4\|CNR1\|CNR2\|CXADR\|DSC2\|GABRR2\|GRIK1\|GRIK5\|GRIN2C\|KCNA1\|KCND2\|KCNN2\|KCNQ1\|P2RX5\|PMAIP1\|RGS4\|RYR2\|SCN1A\|SCN2A\|SCN4A\|SCN10A\|SLC1A7\|SLC8A2\|SLC8A3\|TNF\|GLRA3\|FZD9\|KCNK5\|CCN6\|CACNA1H\|CHRNA6\|HCN4\|KCNK7\|BAIAP2\|MLLT11\|CUX2\|KCNH3\|KCNE4\|KCNH5\|KCNK10\|BEST2\|GABRQ\|TMEM108\|MYH14\|CLDN19\|NPAS4\|ZACN |
|  | GO:0071363 | cellular response to growth factor stimulus | 1E-10 | 2.5 | 7.6 | 59 | ANGPT2\|BMP2\|BMP7\|BMPR1B\|CALCA\|CCNA2\|CDH5\|COL2A1\|CCN2\|CX3CR1\|EGR3\|FBN1\|FGFR3\|FGFR4\|FLT1\|FLT3\|FLT4\|FOS\|GCNT2\|GDF9\|GDF10\|HAS1\|NR4A1\|HES1\|HSPA5\|HSPB1\|LHX1\|MT1G\|NGFR\|PAX9\|PDE2A\|PDGFA\|PITX3\|SPI1\|TAC1\|TBX1\|VIL1\|GDF5\|FGF17\|FGF16\|SPHK1\|HAP1\|ONECUT2\|GDF15\|FGF19\|SPRY2\|BAIAP2\|STMN2\|BAMBI\|ANKRD1\|ERRFI1\|DLL4\|TMEM108\|PDGFD\|DCSTAMP\|PLK5\|TRIM71\|GDF7\|SCX |
|  | GO:0048598 | embryonic morphogenesis | 1E-10 | 2.4 | 7.4 | 66 | BMP7\|FOXL2\|COL2A1\|NKX2-5\|DLX2\|DUSP1\|DUSP2\|DUSP5\|EFNA1\|EPHB2\|FBN1\|FOXG1\|FOXF1\|FOXE1\|FLT1\|GATA2\|GBX2\|GDNF\|HOXB9\|HOXC11\|HES1\|IHH\|INHBA\|ITGA2\|KCNQ1\|LHX1\|MYO7A\|NEUROD1\|PAX6\|PCDH8\|PLS1\|PROX1\|RYR2\|SALL1\|SHH\|SIX3\|SNAI1\|SOX11\|ST14\|TBX1\|WNT6\|GDF5\|EOMES\|RECK\|GCM1\|PLPP3\|HAND1\|MAFB\|SPRY2\|COBL\|CECR2\|DLL1\|SOX8\|ASB2\|SOX18\|DLL4\|GREM2\|SOX7\|MIXL1\|USH1G\|TRIM71\|GDF7\|AMOT\|TMIE\|HMX3\|SCX |
|  | GO:0040011 | locomotion | 1E-10 | 2.4 | 7.5 | 62 | BMP7\|BMPR1B\|CALCA\|CHGA\|CCR4\|CNR2\|CX3CR1\|CXADR\|DSCAM\|RCAN1\|HBEGF\|GPR183\|EFNA1\|EFNA2\|EFNB2\|EGR2\|EGR3\|EPHA8\|EPHB2\|FOXG1\|FLT1\|GBX2\|GDNF\|GRIN2C\|NR4A1\|HOXB9\|CXCR1\|CXCL10\|LHX1\|LSP1\|CXCL9\|NEFL\|NGFR\|PAX6\|PDGFA\|PIK3CG\|PLAU\|PTAFR\|ROBO2\|CCL3\|CCL7\|CCL8\|CCL24\|SHH\|VAV1\|CXCR4\|PLA2G10\|UNC5C\|TNFSF14\|CXCL14\|CCL26\|SEMA6B\|DPYSL4\|ACKR4\|TREM1\|JPH3\|BCL11B\|GDF7\|AMOT\|EPHA6\|DRGX\|CD300H |
|  | GO:0048663 | neuron fate commitment | 2E-10 | 5.9 | 9 | 19 | ASCL1\|DLX2\|FOXG1\|GATA2\|FOXA1\|IHH\|PAX6\|POU3F2\|PROX1\|SHH\|SOX1\|OLIG2\|MYT1L\|DLL1\|DLL4\|FEV\|DMRT3\|DMRTA2\|BCL11B |
|  | GO:0060284 | regulation of cell development | 2.51E-10 | 2.4 | 7.3 | 60 | ACTN3\|ASCL1\|ASCL2\|ASPA\|BMP2\|BMP7\|CDH5\|CX3CR1\|DLX2\|DSCAM\|EGR2\|EPHB2\|F2\|FBN1\|FOXG1\|HES1\|IFRD1\|IGF1\|LTA\|MT3\|NEFL\|NKX6-1\|NPPC\|PAX6\|PITX3\|PROX1\|ROBO2\|SHH\|SOX11\|SPP1\|TNF\|TP73\|CXCR4\|SLC7A5\|RECK\|HAP1\|GPR37L1\|OLIG2\|TRIB1\|BAIAP2\|SEMA6B\|CSPG5\|DLL3\|IL1RAPL1\|ZNF365\|FSTL4\|CUX2\|HEY1\|DLL1\|SOX8\|DLL4\|HES2\|HES6\|ISLR2\|DMRTA2\|HES7\|IL34\|ASPM\|WEE2\|RASSF10 |
|  | GO:0045165 | cell fate commitment | 3.16E-10 | 3.2 | 7.7 | 38 | ASCL1\|BMP2\|NKX2-5\|DLX2\|FOXG1\|GATA2\|FOXA1\|HES1\|IFRD1\|IHH\|IL7\|IRF4\|NEUROD1\|PAX6\|POU3F2\|PROX1\|SHH\|SOX1\|TBX1\|WNT6\|EOMES\|GCM1\|ONECUT2\|OLIG2\|SPRY2\|MYT1L\|DLL1\|TBX21\|SOX8\|DHH\|SOX18\|DLL4\|FEV\|DMRT3\|DMRTA2\|BCL11B\|SLAMF6\|GDF7 |
|  | GO:0030900 | forebrain development | 5.01E-10 | 2.7 | 7.4 | 49 | ASCL1\|ATP1B2\|AVPR2\|BMP2\|DLX2\|EFNA2\|EPHB2\|FOXG1\|FUT1\|GATA2\|GBX2\|HES1\|DNAJB1\|INHBA\|ITGAM\|KCNA1\|LHX1\|NEFL\|NEUROD1\|NR4A2\|PAX6\|POU3F1\|POU3F2\|PROX1\|ROBO2\|SALL1\|SHH\|SIX3\|SLC2A1\|SLC6A3\|SOX1\|SOX3\|SRD5A2\|SSTR2\|SSTR3\|CXCR4\|EOMES\|HAP1\|OLIG2\|SEMA6B\|CNTNAP2\|LHX6\|BCAN\|DMRTA2\|BCL11B\|TMEM108\|MFSD2A\|GDF7\|ASPM |
|  | GO:0030902 | hindbrain development | 7.94E-10 | 3.9 | 7.9 | 28 | ASCL1\|ATP1B2\|BMP7\|CTNNA2\|EGR2\|EN2\|EPHB2\|GATA2\|GBX2\|GDF10\|HES1\|HSPA5\|LHX1\|COX1\|NEUROD1\|PROX1\|RORA\|SHH\|SSTR2\|SSTR3\|HAP1\|MAFB\|ZNF365\|MYO16\|TTLL1\|DLL1\|KNDC1\|NANOS1 |
|  | GO:0090596 | sensory organ morphogenesis | 1E-09 | 3 | 7.4 | 39 | FASLG\|SHROOM2\|AQP5\|BMP7\|FOXL2\|COL2A1\|DIO3\|DSCAM\|EPHB2\|FBN1\|FOXG1\|GATA2\|GBX2\|HOXC13\|IHH\|KCNQ1\|LHX1\|MYO7A\|PAX6\|PITX3\|PLS1\|PROX1\|RP1\|SIX3\|SOX1\|SOX11\|TBX1\|MAFB\|NR2E3\|SPRY2\|DLL1\|SOX8\|CABP4\|MFSD2A\|NKD1\|IGFN1\|USH1G\|TMIE\|HMX3 |
|  | GO:0034765 | regulation of ion transmembrane transport | 1E-09 | 2.4 | 7 | 59 | ACTN2\|ATP1B2\|CACNA1B\|CACNA1E\|EPHB2\|F2\|GEM\|HSPA2\|CXCL10\|KCNA1\|KCNC3\|KCND2\|KCNJ4\|KCNJ6\|KCNN2\|KCNQ1\|KCNQ2\|KCNS1\|KCNS2\|KEL\|CXCL9\|OPRK1\|P2RX5\|P2RY6\|ABCB1\|PIK3CG\|PTAFR\|PTPN6\|RASGRF1\|RGS2\|RGS4\|RRAD\|RYR2\|SCN1A\|SCN2A\|SCN4A\|SCN10A\|TNF\|KCNK5\|GALR2\|CACNA1H\|HAP1\|HCN4\|KCNK7\|ARC\|KCNH3\|KCNE4\|TSPAN13\|KCNH5\|TRPM5\|GAL\|KCNK10\|CABP4\|JPH3\|WNK2\|JSRP1\|GSG1L\|KCNG3\|STAC2 |
|  | GO:0045665 | negative regulation of neuron differentiation | 1E-09 | 5.4 | 8.5 | 19 | ASCL1\|BMP7\|DLX2\|DTX1\|FOXG1\|HES1\|PAX6\|SHH\|SIX3\|SLC6A4\|SOX3\|TP73\|GPR37L1\|OLIG2\|HEY1\|DLL1\|SOX8\|DISP3\|ASPM |
|  | GO:0051960 | regulation of nervous system development | 1.26E-09 | 2.5 | 7.1 | 54 | ASCL1\|ASCL2\|ASPA\|BMP2\|BMP7\|CX3CR1\|DLX2\|DSCAM\|EGR2\|EPHB2\|F2\|FOXG1\|GBX2\|HES1\|IFRD1\|LTA\|MT3\|NEFL\|NKX6-1\|PAX6\|PITX3\|PROX1\|ROBO2\|SHH\|SOX11\|SPP1\|TNF\|TP73\|CXCR4\|SLC7A5\|HAP1\|GPR37L1\|OLIG2\|BAIAP2\|SEMA6B\|DLL3\|IL1RAPL1\|ZNF365\|FSTL4\|CUX2\|HEY1\|DLL1\|SOX8\|DLL4\|HES2\|HES6\|ISLR2\|DMRTA2\|HES7\|LRRC4B\|IL34\|ELAPOR2\|ASPM\|RASSF10 |
|  | GO:0006935 | chemotaxis | 1.26E-09 | 2.4 | 7 | 58 | BMP7\|BMPR1B\|CALCA\|CHGA\|CCR4\|CNR2\|CX3CR1\|CXADR\|DSCAM\|HBEGF\|GPR183\|EFNA1\|EFNA2\|EFNB2\|EGR2\|EGR3\|EPHA8\|EPHB2\|FOXG1\|FLT1\|GBX2\|GDNF\|NR4A1\|HOXB9\|CXCR1\|CXCL10\|LHX1\|LSP1\|CXCL9\|NGFR\|PAX6\|PDGFA\|PIK3CG\|PLAU\|PTAFR\|ROBO2\|CCL3\|CCL7\|CCL8\|CCL24\|SHH\|VAV1\|CXCR4\|PLA2G10\|UNC5C\|TNFSF14\|CXCL14\|CCL26\|SEMA6B\|DPYSL4\|ACKR4\|TREM1\|BCL11B\|GDF7\|AMOT\|EPHA6\|DRGX\|CD300H |
|  | GO:0042330 | taxis | 1.58E-09 | 2.4 | 7 | 58 | BMP7\|BMPR1B\|CALCA\|CHGA\|CCR4\|CNR2\|CX3CR1\|CXADR\|DSCAM\|HBEGF\|GPR183\|EFNA1\|EFNA2\|EFNB2\|EGR2\|EGR3\|EPHA8\|EPHB2\|FOXG1\|FLT1\|GBX2\|GDNF\|NR4A1\|HOXB9\|CXCR1\|CXCL10\|LHX1\|LSP1\|CXCL9\|NGFR\|PAX6\|PDGFA\|PIK3CG\|PLAU\|PTAFR\|ROBO2\|CCL3\|CCL7\|CCL8\|CCL24\|SHH\|VAV1\|CXCR4\|PLA2G10\|UNC5C\|TNFSF14\|CXCL14\|CCL26\|SEMA6B\|DPYSL4\|ACKR4\|TREM1\|BCL11B\|GDF7\|AMOT\|EPHA6\|DRGX\|CD300H |
|  | GO:0019233 | sensory perception of pain | 1.58E-09 | 5.3 | 8.3 | 19 | CALCA\|CHRNA4\|CNR1\|CNR2\|HOXD1\|ITGA2\|KCNA1\|KCND2\|OPRK1\|P2RY1\|PTGS2\|SCN1A\|SCN10A\|TAC1\|TNF\|CXCR4\|PHF24\|PRDM12\|ALOXE3 |
|  | GO:0043408 | regulation of MAPK cascade | 2E-09 | 2.1 | 6.8 | 71 | ADRA2B\|ALOX12B\|ATF3\|BMP2\|BMP7\|CHRNA7\|CCN2\|DUSP1\|DUSP2\|DUSP5\|DUSP8\|GPR183\|EFNA1\|ELANE\|EPHA8\|EPHB2\|MECOM\|FGFR3\|FGFR4\|FLT1\|FLT3\|FLT4\|GCNT2\|IGF1\|IL11\|INHBA\|KITLG\|MT3\|GADD45B\|OPRK1\|OSM\|P2RY1\|P2RY6\|PDGFA\|PIK3CG\|SERPINF2\|PPEF2\|PTPN6\|PTPRR\|RGS2\|CCL3\|CCL7\|CCL8\|CCL24\|SPI1\|TBX1\|TNF\|TP73\|SPHK1\|GPR37L1\|GPR55\|GDF15\|FGF19\|SPRY2\|CCL26\|GRAP\|GADD45G\|FZD10\|CD300A\|PIK3R5\|ERRFI1\|SLC30A10\|INAVA\|PBK\|WNK2\|DUSP26\|PDGFD\|IQGAP3\|IL34\|EPGN\|ALKAL1 |
|  | GO:0001775 | cell activation | 2E-09 | 2.1 | 6.7 | 71 | ADRA2B\|AIRE\|BTK\|CD1D\|CHGA\|CHRNA4\|CX3CR1\|CXADR\|DGKB\|DGKG\|GPR183\|EPHB2\|F2\|FLT3\|HHEX\|HES1\|HSPB1\|HSPD1\|IRF8\|IGF1\|IL7\|IL11\|CXCL10\|IRF4\|ITGAL\|ITGAM\|LCK\|DNAJB9\|KITLG\|MT1G\|P2RY1\|PDGFA\|PIK3CG\|PRKCB\|PTPN6\|RORA\|CCL3\|SHH\|SPI1\|TNF\|TPD52\|VAV1\|ZP2\|EOMES\|FZD9\|PLA2G10\|TNFSF14\|MAFB\|DHRS2\|MERTK\|FGL2\|IKZF3\|ICOSLG\|DLL1\|TBX21\|DLL4\|ENTPD7\|PELI1\|WDFY4\|SLAMF7\|BCL11B\|ULBP1\|DCSTAMP\|CARD11\|MEGF10\|PLCZ1\|SLAMF9\|RSAD2\|SLAMF6\|ASTL\|NRARP |
|  | GO:0010817 | regulation of hormone levels | 2E-09 | 2.3 | 6.9 | 58 | ADCY8\|ADH1B\|BMP2\|FOXL2\|BTK\|CHGA\|CNR1\|CRYM\|CYP1A1\|CYP2C8\|DIO3\|FOXE1\|GDF9\|GIPR\|GPR27\|FOXA1\|IL11\|INHBA\|KCNQ1\|NEUROD1\|NKX3-1\|NKX6-1\|OPRK1\|OSM\|P2RY1\|PTPRN\|SHH\|SLC5A5\|SOX11\|SPINK1\|SPP1\|SRD5A2\|AKR1D1\|HNF1A\|TNF\|TPO\|UGT2B4\|VGF\|VIP\|SLC7A5\|DOC2B\|CACNA1H\|BAIAP3\|NR1H4\|DHRS9\|DHRS2\|CPLX1\|PCLO\|SOX8\|RDH8\|GAL\|TRPV6\|CELA2A\|CHST8\|SLC16A10\|RDH12\|ILDR2\|MAFA |
|  | GO:0021700 | developmental maturation | 2.51E-09 | 3 | 7.2 | 37 | ASCL1\|BMP2\|BTK\|CDH5\|CEBPA\|CX3CR1\|EPHA8\|EREG\|FGFR3\|GATA2\|FOXA1\|HES1\|IGF1\|IHH\|NEFL\|NKX6-1\|NR4A2\|PTPRN\|RYR1\|SIX3\|SPINK1\|PLA2G10\|RECK\|BAIAP3\|DMC1\|SEZ6L\|CNTNAP2\|LHX6\|RND1\|TFCP2L1\|SOX8\|SOX18\|FEV\|BCAN\|SRRM4\|TDRD5\|CATSPERD |
|  | GO:0034762 | regulation of transmembrane transport | 3.16E-09 | 2.2 | 6.7 | 65 | ACTN2\|ATP1B2\|CA2\|CACNA1B\|CACNA1E\|EPHB2\|F2\|GEM\|HSPA2\|IGF1\|CXCL10\|KCNA1\|KCNC3\|KCND2\|KCNJ4\|KCNJ6\|KCNN2\|KCNQ1\|KCNQ2\|KCNS1\|KCNS2\|KEL\|CXCL9\|OPRK1\|P2RX5\|P2RY6\|ABCB1\|PIK3CG\|PRKCB\|PTAFR\|PTPN6\|RASGRF1\|RGS2\|RGS4\|RRAD\|RYR2\|SCN1A\|SCN2A\|SCN4A\|SCN10A\|TNF\|SLC7A5\|KCNK5\|GALR2\|CACNA1H\|HAP1\|FGF19\|HCN4\|KCNK7\|ARC\|KCNH3\|KCNE4\|TSPAN13\|KCNH5\|TRPM5\|GAL\|KCNK10\|ITLN1\|CABP4\|JPH3\|WNK2\|JSRP1\|GSG1L\|KCNG3\|STAC2 |
|  | GO:0002520 | immune system development | 3.16E-09 | 2.1 | 6.6 | 74 | AIRE\|BTK\|CBFA2T3\|RUNX3\|CD1D\|CD34\|CEBPA\|CCR4\|NKX2-5\|GPR183\|EFNA2\|MECOM\|FOXJ1\|FOXE1\|FLT1\|FLT3\|FLT4\|FOS\|GATA2\|HHEX\|HLA-G\|HES1\|HSPD1\|IRF8\|IL7\|IL11\|INHBA\|IRF4\|KCNQ1\|LCK\|LTA\|DNAJB9\|KITLG\|MT1G\|PTPN6\|RORA\|SHH\|SLC4A1\|SLC8A3\|SPI1\|TBX1\|TNF\|TOP2A\|TPD52\|TPO\|VAV1\|EOMES\|FZD9\|PLA2G10\|KRT75\|MAFB\|DHRS2\|LRRC17\|MERTK\|IKZF3\|DLL1\|TBX21\|DLL4\|BARX1\|ENTPD7\|CD248\|BCL11B\|DCSTAMP\|MIXL1\|CARD11\|RSAD2\|SLAMF6\|MMP21\|IL31RA\|TMEM190\|ESCO2\|DACT2\|WDR38\|NRARP |
|  | GO:0051046 | regulation of secretion | 3.98E-09 | 2.2 | 6.6 | 65 | ACHE\|ADCY8\|ADRA2B\|ALOX12B\|AVPR2\|BMP2\|FOXL2\|CHGA\|CHRNA4\|CHRNA5\|CHRNB4\|CNR1\|FOXF1\|GATA2\|GDF9\|GDNF\|GIPR\|GPR27\|IGF1\|IL11\|INHBA\|ITGAM\|KCNQ1\|NEUROD1\|NKX3-1\|NKX6-1\|OPRK1\|OSM\|P2RY1\|SEPTIN4\|PRKCB\|PRKCG\|PTAFR\|PTGER3\|PTPRN\|RAB27B\|SLC6A4\|SOX11\|SPI1\|SPINK1\|SPP1\|STC1\|TNF\|VIP\|PLA2G10\|DOC2B\|CACNA1H\|BAIAP3\|CHRNA6\|HAP1\|NR1H4\|CSPG5\|CPLX1\|IL1RAPL1\|CD300A\|ANKRD1\|PCLO\|GAL\|TRPV6\|CELA2A\|MCTP1\|OR51E2\|RSAD2\|SYT6\|LGI3 |
|  | GO:0022407 | regulation of cell-cell adhesion | 5.01E-09 | 2.3 | 6.7 | 55 | BMP2\|BMP7\|RUNX3\|CD1D\|CDSN\|EFNB2\|CELSR2\|EGR3\|ELANE\|FOXJ1\|FUT3\|GCNT2\|HLA-DOB\|HLA-G\|FOXA1\|HES1\|HSPD1\|IGF1\|IHH\|IL7\|LCK\|KITLG\|PNP\|PCDH8\|PCK1\|SERPINF2\|PTAFR\|PTPN6\|SHH\|SPI1\|TNF\|VAV1\|PLPP3\|TNFSF14\|TNFSF9\|HSPH1\|FGL2\|CD300A\|ICOSLG\|IL36B\|TBX21\|PELI1\|CELA2A\|VTCN1\|CARD11\|MEGF10\|NLRP3\|ADAMTS18\|CCDC88B\|NEXMIF\|ILDR2\|MIRLET7G\|MIR31\|NRARP\|TARM1 |
|  | GO:0048534 | hematopoietic or lymphoid organ development | 6.31E-09 | 2.1 | 6.5 | 70 | AIRE\|BTK\|CBFA2T3\|RUNX3\|CD1D\|CD34\|CEBPA\|NKX2-5\|GPR183\|EFNA2\|MECOM\|FOXE1\|FLT1\|FLT3\|FLT4\|FOS\|GATA2\|HHEX\|HES1\|IRF8\|IL7\|IL11\|INHBA\|IRF4\|KCNQ1\|LCK\|LTA\|DNAJB9\|KITLG\|MT1G\|PTPN6\|RORA\|SHH\|SLC4A1\|SLC8A3\|SPI1\|TBX1\|TNF\|TOP2A\|TPD52\|TPO\|VAV1\|EOMES\|FZD9\|PLA2G10\|KRT75\|MAFB\|DHRS2\|LRRC17\|MERTK\|IKZF3\|DLL1\|TBX21\|DLL4\|BARX1\|ENTPD7\|CD248\|BCL11B\|DCSTAMP\|MIXL1\|CARD11\|RSAD2\|SLAMF6\|MMP21\|IL31RA\|TMEM190\|ESCO2\|DACT2\|WDR38\|NRARP |
|  | GO:0042026 | protein refolding | 7.94E-09 | 9.2 | 9.2 | 11 | CRYAB\|HSPA1A\|HSPA1B\|HSPA2\|HSPA5\|HSPA6\|HSPA7\|HSPB1\|HSP90AA1\|HSPD1\|DNAJA4 |
|  | GO:0007610 | behavior | 1E-08 | 2.2 | 6.5 | 62 | ADCY8\|ATP1B2\|BRS3\|CALCA\|CCK\|CHRNA4\|CHRNA5\|CHRNA7\|CHRNB4\|CNR1\|CNTFR\|CX3CR1\|DSCAM\|RCAN1\|EGR2\|EPHB2\|FOS\|GAD1\|GATM\|GDNF\|KCND2\|KCNQ1\|NPTX2\|NR4A2\|OPRK1\|P2RY1\|PITX3\|PRKCG\|PTGS2\|RASGRF1\|SCN1A\|SCN2A\|SLC2A4\|SLC6A3\|SLC6A4\|SLC8A2\|SLC8A3\|TAC1\|TBX1\|VGF\|VIP\|FZD9\|GALR2\|GDF15\|GPR83\|ARC\|CUX2\|RASD2\|CNTNAP2\|B3GAT1\|PCDH17\|GAL\|KCNK10\|ASIC4\|LPAR5\|JPH3\|DMRT3\|DMRTA1\|MYH14\|MFSD2A\|DMBX1\|NPAS4 |
|  | GO:0071695 | anatomical structure maturation | 1.26E-08 | 3.1 | 6.9 | 32 | ASCL1\|BMP2\|BTK\|CDH5\|CEBPA\|CX3CR1\|EPHA8\|EREG\|FGFR3\|GATA2\|FOXA1\|HES1\|IGF1\|IHH\|NKX6-1\|NR4A2\|RYR1\|SIX3\|SPINK1\|PLA2G10\|RECK\|DMC1\|CNTNAP2\|LHX6\|RND1\|TFCP2L1\|SOX8\|SOX18\|FEV\|SRRM4\|TDRD5\|CATSPERD |
|  | GO:0036293 | response to decreased oxygen levels | 1.26E-08 | 2.7 | 6.7 | 39 | ANGPT2\|ASCL2\|BMP2\|BMP7\|CBFA2T3\|CCNA2\|CHRNA4\|CHRNA7\|CRYAB\|CCN2\|CYP1A1\|DIO3\|FOS\|ITGA2\|KCND2\|LTA\|MT3\|NKX3-1\|NPPC\|NR4A2\|PDGFA\|PLAU\|PMAIP1\|PTGS2\|RORA\|RYR1\|RYR2\|SCN2A\|SLC2A1\|SLC2A4\|SLC6A4\|SLC8A3\|STC1\|TERC\|TNF\|CXCR4\|CPEB4\|MYOCD\|EGLN3 |
|  | GO:0070482 | response to oxygen levels | 2E-08 | 2.6 | 6.6 | 41 | ANGPT2\|ASCL2\|BMP2\|BMP7\|CBFA2T3\|CCNA2\|CHRNA4\|CHRNA7\|CRYAB\|CCN2\|CYP1A1\|DIO3\|FOS\|ITGA2\|KCND2\|LTA\|MT3\|ATP6\|NKX3-1\|NPPC\|NR4A2\|PDGFA\|PLAU\|PMAIP1\|PTGS2\|RORA\|RYR1\|RYR2\|SCN2A\|SLC2A1\|SLC2A4\|SLC6A4\|SLC8A3\|STC1\|TERC\|TNF\|CXCR4\|SLC7A5\|CPEB4\|MYOCD\|EGLN3 |
|  | GO:0009266 | response to temperature stimulus | 2E-08 | 3.3 | 7 | 28 | CALCA\|CRYAB\|FOS\|HSPA1A\|HSPA1B\|HSPA2\|HSPA6\|HSPB1\|HSP90AA1\|HSPD1\|DNAJB1\|IGF1\|CXCL10\|COX2\|NGFR\|PTGS2\|UCP1\|VGF\|CXCR4\|FGF16\|SLC25A27\|BAG3\|TFEC\|DNAJA4\|PRDM12\|ANO3\|SCARA5\|DRGX |
|  | GO:0030855 | epithelial cell differentiation | 2E-08 | 2.1 | 6.3 | 62 | ASCL1\|BMP2\|BMP7\|FOXL2\|CD34\|CDSN\|CEBPA\|NKX2-5\|CYP1A1\|DMRT1\|EREG\|FOXF1\|FOXJ1\|GATA2\|GDNF\|FOXA1\|HES1\|IHH\|ITGA2\|KCNQ1\|MSI1\|MYO7A\|NEUROD1\|NKX6-1\|NPHS1\|PAX6\|PCK1\|PDE2A\|PITX3\|PLS1\|POU3F1\|POU3F2\|PROX1\|SALL1\|SIX3\|SOX11\|ST14\|STC1\|TBX1\|TGM3\|VIL1\|CXCR4\|KRT75\|ONECUT2\|MAFB\|DHRS9\|PLK4\|HEY1\|TJP3\|DLL1\|TFCP2L1\|SOX8\|RHCG\|SOX18\|BARX1\|CAMSAP3\|BCL11B\|TMEM132E\|IL31RA\|GDF7\|DACT2\|SCX |
|  | GO:1903530 | regulation of secretion by cell | 2.51E-08 | 2.2 | 6.3 | 59 | ACHE\|ADCY8\|ADRA2B\|BMP2\|FOXL2\|CHGA\|CHRNA4\|CHRNA5\|CHRNB4\|CNR1\|FOXF1\|GATA2\|GDF9\|GDNF\|GIPR\|GPR27\|IGF1\|IL11\|INHBA\|ITGAM\|NEUROD1\|NKX3-1\|NKX6-1\|OPRK1\|OSM\|P2RY1\|SEPTIN4\|PRKCB\|PRKCG\|PTAFR\|RAB27B\|SLC6A4\|SOX11\|SPI1\|SPINK1\|SPP1\|TNF\|VIP\|PLA2G10\|DOC2B\|CACNA1H\|BAIAP3\|CHRNA6\|HAP1\|NR1H4\|CSPG5\|CPLX1\|IL1RAPL1\|CD300A\|ANKRD1\|PCLO\|GAL\|TRPV6\|CELA2A\|MCTP1\|OR51E2\|RSAD2\|SYT6\|LGI3 |
|  | GO:0021537 | telencephalon development | 2.51E-08 | 2.9 | 6.7 | 35 | ASCL1\|ATP1B2\|AVPR2\|BMP2\|DLX2\|EFNA2\|EPHB2\|FOXG1\|FUT1\|HES1\|INHBA\|KCNA1\|LHX1\|NEFL\|NEUROD1\|PAX6\|POU3F2\|PROX1\|ROBO2\|SALL1\|SHH\|SIX3\|SLC2A1\|SRD5A2\|CXCR4\|EOMES\|SEMA6B\|CNTNAP2\|LHX6\|BCAN\|DMRTA2\|BCL11B\|TMEM108\|MFSD2A\|ASPM |
|  | GO:0007517 | muscle organ development | 2.51E-08 | 2.7 | 6.6 | 39 | ATF3\|BMP2\|FOXL2\|CNTFR\|CRYAB\|NKX2-5\|RCAN1\|HBEGF\|EGR2\|EGR3\|FOS\|H1-5\|NR4A1\|IGF1\|CXCL10\|KEL\|NPHS1\|PROX1\|RYR1\|RYR2\|SHH\|SOX11\|TBX1\|CSRP3\|EOMES\|CACNA1H\|HAND1\|ANKRD1\|DLL1\|SOX8\|ASB2\|DLL4\|DMRTA2\|MYH14\|MEGF10\|XIRP2\|KY\|TRIM72\|SCX |
|  | GO:0048562 | embryonic organ morphogenesis | 2.51E-08 | 2.7 | 6.6 | 38 | BMP7\|FOXL2\|COL2A1\|NKX2-5\|DLX2\|EFNA1\|EPHB2\|FBN1\|FOXG1\|FOXF1\|FOXE1\|GATA2\|GBX2\|HOXB9\|HOXC11\|HES1\|IHH\|KCNQ1\|LHX1\|MYO7A\|NEUROD1\|PAX6\|PLS1\|PROX1\|RYR2\|SHH\|SIX3\|SOX11\|TBX1\|HAND1\|MAFB\|SPRY2\|DLL1\|ASB2\|SOX18\|USH1G\|TMIE\|HMX3 |
|  | GO:0008283 | cell population proliferation | 3.16E-08 | 2 | 6.2 | 68 | ASCL1\|BMP2\|BMP7\|BTK\|CD34\|CEBPA\|NKX2-5\|CCN2\|CXADR\|CYP1A1\|DIO3\|DMRT1\|EFNB2\|EREG\|MECOM\|FGFR3\|FOXG1\|FLT3\|FOS\|GATA2\|GBX2\|HES1\|HSPD1\|IGF1\|IHH\|IL7\|IL11\|ITGA2\|KCNA1\|KITLG\|NGFR\|NKX3-1\|NKX6-1\|NPPC\|PAX6\|ABCB1\|PIK3CG\|POU3F2\|PROX1\|PTPN6\|RASGRF1\|RORA\|SHH\|SIX3\|SOX11\|STC1\|TBX1\|TNF\|FZD9\|TNFSF14\|SPHK1\|SQSTM1\|NR2E3\|CNTNAP2\|EAF2\|CD248\|PELI1\|DMRTA2\|OR51E2\|CARD11\|MEGF10\|IQGAP3\|TRIM71\|IL34\|DIPK2A\|FAM83B\|ASPM\|NRARP |
|  | GO:2000179 | positive regulation of neural precursor cell proliferation | 3.16E-08 | 5.7 | 7.8 | 15 | ASCL1\|CX3CR1\|FOXG1\|LHX1\|PAX6\|PITX3\|PROX1\|SHH\|FZD9\|GPR37L1\|DLL4\|DISP3\|DMRTA2\|ASPM\|RASSF10 |
|  | GO:0014013 | regulation of gliogenesis | 3.16E-08 | 4.1 | 7.2 | 21 | ASCL2\|ASPA\|BMP2\|DLX2\|EGR2\|F2\|HES1\|LTA\|NKX6-1\|PITX3\|SHH\|SOX11\|TNF\|TP73\|CXCR4\|SLC7A5\|GPR37L1\|OLIG2\|ZNF365\|SOX8\|IL34 |
|  | GO:0048592 | eye morphogenesis | 3.98E-08 | 3.4 | 6.9 | 26 | FASLG\|SHROOM2\|AQP5\|BMP7\|FOXL2\|DIO3\|DSCAM\|EPHB2\|FBN1\|IHH\|LHX1\|MYO7A\|PAX6\|PITX3\|PROX1\|RP1\|SIX3\|SOX1\|SOX11\|NR2E3\|DLL1\|SOX8\|CABP4\|MFSD2A\|NKD1\|IGFN1 |
|  | GO:0001666 | response to hypoxia | 3.98E-08 | 2.7 | 6.5 | 37 | ANGPT2\|ASCL2\|BMP2\|BMP7\|CBFA2T3\|CCNA2\|CHRNA4\|CHRNA7\|CRYAB\|CYP1A1\|DIO3\|FOS\|ITGA2\|KCND2\|LTA\|MT3\|NKX3-1\|NPPC\|NR4A2\|PDGFA\|PLAU\|PMAIP1\|PTGS2\|RORA\|RYR1\|RYR2\|SCN2A\|SLC2A1\|SLC2A4\|SLC6A4\|SLC8A3\|STC1\|TERC\|TNF\|CXCR4\|MYOCD\|EGLN3 |
|  | GO:2000177 | regulation of neural precursor cell proliferation | 3.98E-08 | 4.4 | 7.2 | 19 | ASCL1\|CX3CR1\|FOXG1\|GATA2\|LHX1\|PAX6\|PITX3\|PROX1\|SHH\|SIX3\|SLC6A4\|FZD9\|GPR37L1\|DLL4\|DISP3\|DMRTA2\|TRIM71\|ASPM\|RASSF10 |
|  | GO:0001934 | positive regulation of protein phosphorylation | 5.01E-08 | 2 | 6.1 | 71 | ADCY8\|ADRA2B\|BMP2\|BMP7\|BMPR1B\|CALCA\|CHRNA7\|CCN2\|CX3CR1\|HBEGF\|EFNA1\|ELANE\|EREG\|ERCC6\|F2\|FCGR1A\|FGFR3\|FLT1\|FLT3\|FLT4\|GDF9\|GDF10\|HES1\|HSPA2\|HSP90AA1\|IGF1\|IL11\|INHBA\|INHBC\|KITLG\|MT3\|NKX3-1\|OSM\|P2RY1\|PDGFA\|PIK3CG\|PLK1\|PROX1\|PTGS2\|SLC8A2\|TBX1\|TNF\|TTK\|GDF5\|PLPP3\|FGF17\|FGF16\|SPHK1\|SQSTM1\|GPRC5A\|GDF15\|FGF19\|SPRY2\|IL24\|FZD10\|PIK3R5\|MRNIP\|FAM20A\|GPRC5D\|ITLN1\|PDGFD\|KNDC1\|IQGAP3\|IL31RA\|IL34\|CLDN19\|GDF7\|DIPK2A\|EPGN\|IRGM\|ALKAL1 |
|  | GO:0043410 | positive regulation of MAPK cascade | 5.01E-08 | 2.2 | 6.2 | 53 | ADRA2B\|ALOX12B\|BMP2\|CHRNA7\|CCN2\|GPR183\|EFNA1\|ELANE\|EPHA8\|FGFR3\|FGFR4\|FLT1\|FLT3\|FLT4\|GCNT2\|IGF1\|IL11\|INHBA\|KITLG\|MT3\|GADD45B\|OPRK1\|OSM\|P2RY1\|P2RY6\|PDGFA\|PIK3CG\|SERPINF2\|CCL3\|CCL7\|CCL8\|CCL24\|SPI1\|TBX1\|TNF\|TP73\|SPHK1\|GPR37L1\|GPR55\|GDF15\|FGF19\|SPRY2\|CCL26\|GADD45G\|FZD10\|PIK3R5\|SLC30A10\|INAVA\|PDGFD\|IQGAP3\|IL34\|EPGN\|ALKAL1 |
|  | GO:0021953 | central nervous system neuron differentiation | 6.31E-08 | 3.3 | 6.7 | 27 | ASCL1\|BMPR1B\|DLX2\|EPHB2\|FOXG1\|GATA2\|GBX2\|HES1\|HSP90AA1\|INHBA\|LHX1\|NKX6-1\|NR4A2\|PAX6\|PROX1\|RORA\|SHH\|SOX1\|EOMES\|OLIG2\|TTLL1\|LHX6\|DLL4\|DMRT3\|BCL11B\|KNDC1\|GDF7 |
|  | GO:0035725 | sodium ion transmembrane transport | 6.31E-08 | 3.7 | 6.8 | 23 | SHROOM2\|ATP1A4\|ATP1B2\|SCN1A\|SCN2A\|SCN4A\|SCN10A\|SLC3A2\|SLC5A5\|SLC6A3\|SLC6A4\|SLC6A7\|SLC6A12\|SLC6A13\|SLC8A2\|SLC8A3\|SLC12A1\|CACNA1H\|HCN4\|SLC24A2\|TRPM5\|ASIC4\|SLC5A10 |
| KEGG Pathway | hsa04060 | Cytokine-cytokine receptor interaction | 1E-10 | 3 | 7.9 | 43 | FASLG\|BMP2\|BMP7\|BMPR1B\|CCR4\|CNTFR\|CX3CR1\|GDF9\|GDF10\|IL7\|CXCR1\|IL10RA\|IL11\|IL12RB2\|INHBA\|INHBC\|CXCL10\|LTA\|CXCL9\|NGFR\|OSM\|CCL3\|CCL7\|CCL8\|CCL24\|TNF\|CXCR4\|GDF5\|TNFSF14\|TNFSF9\|IL18RAP\|IL1RL1\|GDF15\|CXCL14\|CCL26\|IL24\|IL36RN\|IL36B\|ACKR4\|RELT\|IL31RA\|IL34\|GDF7 |
|  | hsa04080 | Neuroactive ligand-receptor interaction | 2E-09 | 2.6 | 7.1 | 46 | ADRA2B\|AVPR2\|BRS3\|CALCA\|CCK\|CHRM1\|CHRM4\|CHRNA2\|CHRNA4\|CHRNA5\|CHRNA7\|CHRNB4\|CNR1\|CNR2\|F2\|GABRR2\|GCGR\|GIPR\|MLNR\|GRIK1\|GRIK5\|GRIN2C\|GRM8\|MC5R\|OPRK1\|P2RX5\|P2RY1\|P2RY6\|PTAFR\|PTGER3\|SSTR2\|SSTR3\|TAC1\|TAC3\|VGF\|VIP\|VIPR2\|GLRA3\|GALR2\|CHRNA6\|GPR50\|GABBR2\|GPR83\|GAL\|GABRQ\|UCN2 |
|  | hsa04010 | MAPK signaling pathway | 3.16E-09 | 2.8 | 7.1 | 40 | ANGPT2\|FASLG\|CACNA1B\|CACNA1E\|DUSP1\|DUSP2\|DUSP5\|DUSP8\|EFNA1\|EFNA2\|EREG\|MECOM\|FGFR3\|FGFR4\|FLT1\|FLT3\|FLT4\|FOS\|NR4A1\|HSPA1A\|HSPA1B\|HSPA2\|HSPA6\|HSPB1\|IGF1\|KITLG\|GADD45B\|NGFR\|PDGFA\|PRKCB\|PRKCG\|PTPRR\|RASGRF1\|TNF\|FGF17\|FGF16\|CACNA1H\|FGF19\|GADD45G\|PDGFD |
|  | hsa04061 | Viral protein interaction with cytokine and cytokine receptor | 5.01E-08 | 4.2 | 7.1 | 20 | CCR4\|CX3CR1\|CXCR1\|IL10RA\|CXCL10\|LTA\|CXCL9\|CCL3\|CCL7\|CCL8\|CCL24\|TNF\|CXCR4\|TNFSF14\|IL18RAP\|CXCL14\|CCL26\|IL24\|ACKR4\|IL34 |
|  | hsa05322 | Systemic lupus erythematosus | 1.26E-07 | 3.5 | 6.6 | 23 | C7\|ELANE\|FCGR1A\|H2AC8\|H2BC5\|H2BC3\|HLA-DOB\|TNF\|H2AC16\|H2AC18\|H2BC14\|H2BC17\|H3C1\|H3C3\|H3C12\|H4C4\|H4C8\|H4C5\|H3C14\|H3C15\|H2BC18\|H3C13\|H2AC19 |
|  | hsa05202 | Transcriptional misregulation in cancer | 6.31E-07 | 2.9 | 6 | 27 | CCNA2\|CEBPA\|ELANE\|FCGR1A\|FLT1\|FLT3\|GZMB\|HHEX\|IGF1\|ITGAM\|MMP3\|GADD45B\|NGFR\|PDGFA\|PLAU\|SPI1\|H3C1\|H3C3\|H3C12\|CCNA1\|BAIAP3\|GADD45G\|FEV\|BCL11B\|H3C14\|H3C15\|H3C13 |
|  | hsa04613 | Neutrophil extracellular trap formation | 1.58E-06 | 2.8 | 5.7 | 26 | ELANE\|FCGR1A\|H2AC8\|H2BC5\|H2BC3\|ITGAL\|ITGAM\|NCF2\|PRKCB\|PRKCG\|H2AC16\|H2AC18\|H2BC14\|H2BC17\|H3C1\|H3C3\|H3C12\|H4C4\|H4C8\|H4C5\|SIGLEC9\|H3C14\|H3C15\|H2BC18\|H3C13\|H2AC19 |
|  | hsa04020 | Calcium signaling pathway | 1.58E-06 | 2.6 | 5.6 | 30 | ADCY8\|CACNA1B\|CACNA1E\|CHRM1\|CHRNA7\|FGFR3\|FGFR4\|FLT1\|FLT4\|GDNF\|GNAL\|GRIN2C\|P2RX5\|PDGFA\|PRKCB\|PRKCG\|PTAFR\|PTGER3\|RYR1\|RYR2\|SLC8A2\|SLC8A3\|CXCR4\|FGF17\|FGF16\|SPHK1\|CACNA1H\|FGF19\|PDGFD\|PLCZ1 |
|  | hsa04725 | Cholinergic synapse | 2E-06 | 3.5 | 6 | 19 | ACHE\|ADCY8\|CACNA1B\|CHRM1\|CHRM4\|CHRNA4\|CHRNA7\|CHRNB4\|FOS\|GNG4\|KCNJ4\|KCNJ6\|KCNQ1\|KCNQ2\|PIK3CG\|PRKCB\|PRKCG\|CHRNA6\|PIK3R5 |
|  | hsa04950 | Maturity onset diabetes of the young | 2E-06 | 7.2 | 7.1 | 9 | HHEX\|FOXA3\|HNF4G\|HES1\|NEUROD1\|NKX6-1\|PAX6\|HNF1A\|MAFA |
|  | hsa05200 | Pathways in cancer | 5.01E-06 | 2 | 5 | 50 | ADCY8\|FASLG\|BMP2\|CCNA2\|CEBPA\|CTNNA2\|MECOM\|F2\|FGFR3\|FGFR4\|FLT3\|FLT4\|FOS\|GNG4\|HES1\|HSP90AA1\|IGF1\|IL7\|IL12RB2\|ITGA2\|KITLG\|MMP1\|GADD45B\|NKX3-1\|PDGFA\|PMAIP1\|PRKCB\|PRKCG\|PTGER3\|PTGS2\|SHH\|SLC2A1\|SPI1\|TERC\|WNT6\|CXCR4\|FZD9\|FGF17\|FGF16\|CCNA1\|FGF19\|DLL3\|GADD45G\|FZD10\|FRAT2\|HEY1\|DLL1\|DLL4\|LPAR5\|EGLN3 |
|  | hsa04015 | Rap1 signaling pathway | 3.16E-05 | 2.5 | 4.8 | 25 | ADCY8\|ANGPT2\|CNR1\|EFNA1\|EFNA2\|FGFR3\|FGFR4\|FLT1\|FLT4\|IGF1\|ITGAL\|ITGAM\|KITLG\|NGFR\|P2RY1\|PDGFA\|PRKCB\|PRKCG\|VAV1\|FGF17\|FGF16\|RAPGEF5\|FGF19\|LPAR5\|PDGFD |
|  | hsa05034 | Alcoholism | 0.0001 | 2.4 | 4.5 | 22 | FOSB\|GNG4\|GRIN2C\|H2AC8\|H2BC5\|H2BC3\|SLC6A3\|H2AC16\|H2AC18\|H2BC14\|H2BC17\|H3C1\|H3C3\|H3C12\|H4C4\|H4C8\|H4C5\|H3C14\|H3C15\|H2BC18\|H3C13\|H2AC19 |
|  | hsa04151 | PI3K-Akt signaling pathway | 0.0001 | 2 | 4.2 | 34 | ANGPT2\|FASLG\|CHRM1\|COL2A1\|COL9A1\|EFNA1\|EFNA2\|EREG\|FGFR3\|FGFR4\|FLT1\|FLT3\|FLT4\|GNG4\|NR4A1\|HSP90AA1\|IGF1\|IL7\|ITGA2\|KITLG\|NGFR\|OSM\|PCK1\|PDGFA\|PIK3CG\|PPP2R2C\|SPP1\|FGF17\|FGF16\|FGF19\|PIK3R5\|LPAR5\|PDGFD\|COL6A6 |
|  | hsa04974 | Protein digestion and absorption | 0.000126 | 3 | 4.6 | 15 | ATP1A4\|ATP1B2\|COL2A1\|COL9A1\|KCNQ1\|SLC3A2\|SLC8A2\|SLC8A3\|KCNK5\|COL20A1\|CELA2A\|SLC16A10\|COL6A6\|COL26A1\|CTRB2 |
|  | hsa04940 | Type I diabetes mellitus | 0.000158 | 4.4 | 4.9 | 9 | FASLG\|GAD1\|GZMB\|HLA-DOB\|HLA-G\|HSPD1\|LTA\|PTPRN\|TNF |
|  | hsa04014 | Ras signaling pathway | 0.0002 | 2.2 | 4.2 | 25 | ANGPT2\|FASLG\|EFNA1\|EFNA2\|FGFR3\|FGFR4\|FLT1\|FLT3\|FLT4\|GNG4\|IGF1\|KITLG\|NGFR\|PDGFA\|PRKCB\|PRKCG\|RASGRF1\|PLA2G10\|FGF17\|FGF16\|RAPGEF5\|FGF19\|PDGFD\|PLA2G12B\|KSR2 |
|  | hsa04727 | GABAergic synapse | 0.000316 | 3 | 4.3 | 13 | ADCY8\|CACNA1B\|GABRR2\|GAD1\|GNG4\|KCNJ6\|PRKCB\|PRKCG\|SLC6A12\|SLC6A13\|HAP1\|GABBR2\|GABRQ |
|  | hsa04972 | Pancreatic secretion | 0.000398 | 2.9 | 4.2 | 14 | ADCY8\|ATP1A4\|ATP1B2\|CA2\|CCK\|KCNQ1\|PRKCB\|PRKCG\|RAB27B\|RYR2\|PLA2G10\|CELA2A\|PLA2G12B\|CTRB2 |
|  | hsa05224 | Breast cancer | 0.000794 | 2.4 | 3.8 | 17 | FLT4\|FOS\|HES1\|IGF1\|GADD45B\|WNT6\|FZD9\|FGF17\|FGF16\|FGF19\|DLL3\|GADD45G\|FZD10\|FRAT2\|HEY1\|DLL1\|DLL4 |
|  | hsa04062 | Chemokine signaling pathway | 0.002512 | 2.1 | 3.3 | 19 | ADCY8\|CCR4\|CX3CR1\|GNG4\|CXCR1\|CXCL10\|CXCL9\|PIK3CG\|PRKCB\|CCL3\|CCL7\|CCL8\|CCL24\|VAV1\|CXCR4\|CXCL14\|CCL26\|PIK3R5\|GRK7 |
|  | hsa05033 | Nicotine addiction | 0.002512 | 3.6 | 3.8 | 7 | CACNA1B\|CHRNA4\|CHRNA7\|GABRR2\|GRIN2C\|CHRNA6\|GABRQ |
|  | hsa04911 | Insulin secretion | 0.002512 | 2.7 | 3.5 | 11 | ADCY8\|ATP1A4\|ATP1B2\|CCK\|KCNN2\|KCNN3\|PRKCB\|PRKCG\|RYR2\|SLC2A1\|PCLO |
|  | hsa04670 | Leukocyte transendothelial migration | 0.003162 | 2.4 | 3.3 | 13 | CDH5\|CTNNA2\|ITGAL\|ITGAM\|NCF2\|CLDN11\|PRKCB\|PRKCG\|TXK\|VAV1\|CXCR4\|CLDN14\|CLDN19 |
|  | hsa04971 | Gastric acid secretion | 0.003162 | 2.7 | 3.4 | 10 | ADCY8\|ATP1A4\|ATP1B2\|CA2\|GAST\|KCNQ1\|PRKCB\|PRKCG\|SSTR2\|KCNK10 |
|  | hsa04721 | Synaptic vesicle cycle | 0.003981 | 2.7 | 3.3 | 10 | CACNA1B\|SLC1A7\|SLC6A3\|SLC6A4\|SLC6A7\|SLC6A12\|SLC6A13\|CPLX1\|ATP6V0E2\|ATP6V0D2 |
|  | hsa04064 | NF-kappa B signaling pathway | 0.003981 | 2.4 | 3.2 | 12 | BTK\|LCK\|LTA\|GADD45B\|PLAU\|PRKCB\|PTGS2\|TNF\|TNFSF14\|GADD45G\|CARD11\|EDARADD |
|  | hsa04810 | Regulation of actin cytoskeleton | 0.003981 | 1.9 | 3 | 20 | CHRM1\|CHRM4\|F2\|FGFR3\|FGFR4\|INSRR\|ITGA2\|ITGAL\|ITGAM\|PDGFA\|VAV1\|CXCR4\|FGF17\|FGF16\|FGF19\|BAIAP2\|LPAR5\|MYH14\|PDGFD\|IQGAP3 |
|  | hsa05323 | Rheumatoid arthritis | 0.005012 | 2.5 | 3.2 | 11 | FLT1\|FOS\|HLA-DOB\|IL11\|ITGAL\|MMP1\|MMP3\|CCL3\|TNF\|ATP6V0E2\|ATP6V0D2 |
|  | hsa04072 | Phospholipase D signaling pathway | 0.005012 | 2.1 | 3 | 15 | ADCY8\|AVPR2\|DGKB\|DGKG\|F2\|GRM8\|CXCR1\|KITLG\|PDGFA\|PIK3CG\|PLPP3\|SPHK1\|PIK3R5\|LPAR5\|PDGFD |
|  | hsa04350 | TGF-beta signaling pathway | 0.005012 | 2.4 | 3.1 | 11 | BMP2\|BMP7\|BMPR1B\|FBN1\|INHBA\|INHBC\|TNF\|GDF5\|BAMBI\|GREM2\|GDF7 |
|  | hsa04713 | Circadian entrainment | 0.00631 | 2.4 | 3 | 11 | ADCY8\|FOS\|GNG4\|GRIN2C\|KCNJ6\|PRKCB\|PRKCG\|RYR1\|RYR2\|CACNA1H\|RASD1 |
|  | hsa05143 | African trypanosomiasis | 0.007943 | 3.4 | 3.2 | 6 | FASLG\|HBA1\|HBA2\|PRKCB\|PRKCG\|TNF |
|  | hsa04640 | Hematopoietic cell lineage | 0.007943 | 2.3 | 2.9 | 11 | CD1D\|CD34\|FCGR1A\|FLT3\|HLA-DOB\|IL7\|IL11\|ITGA2\|ITGAM\|KITLG\|TNF |
|  | hsa05417 | Lipid and atherosclerosis | 0.007943 | 1.8 | 2.8 | 19 | FASLG\|CYP1A1\|CYP2C8\|FOS\|HSPA1A\|HSPA1B\|HSPA2\|HSPA5\|HSPA6\|HSP90AA1\|HSPD1\|MMP1\|MMP3\|NCF2\|CCL3\|TNF\|VAV1\|POU2F3\|NLRP3 |
|  | hsa05022 | Pathways of neurodegeneration - multiple diseases | 0.007943 | 1.5 | 2.6 | 35 | FASLG\|CACNA1B\|CHRM1\|CHRNA7\|GRIN2C\|HSPA5\|ATP6\|ATP8\|COX1\|COX2\|COX3\|CYTB\|NEFM\|NEFH\|NEFL\|PRKCB\|PRKCG\|PTGS2\|RYR1\|RYR2\|SLC6A3\|TNF\|WNT6\|FZD9\|SQSTM1\|HAP1\|TUBB4A\|FZD10\|FRAT2\|DKK4\|DNAH3\|COX6B2\|DNAH2\|KLC3\|TUBB2B |
|  | hsa04390 | Hippo signaling pathway | 0.007943 | 2 | 2.8 | 15 | BMP2\|BMP7\|BMPR1B\|CCN2\|CTNNA2\|PPP2R2C\|TP73\|WNT6\|GDF5\|FZD9\|FZD10\|NKD1\|GDF7\|AMOT\|RASSF6 |
|  | hsa04260 | Cardiac muscle contraction | 0.007943 | 2.4 | 2.9 | 10 | ATP1A4\|ATP1B2\|COX1\|COX2\|COX3\|CYTB\|RYR2\|SLC8A2\|SLC8A3\|COX6B2 |
|  | hsa04726 | Serotonergic synapse | 0.01 | 2.2 | 2.8 | 12 | ALOX12B\|CACNA1B\|CYP2C8\|DUSP1\|GNG4\|KCND2\|KCNJ6\|KCNN2\|PRKCB\|PRKCG\|PTGS2\|SLC6A4 |
|  | hsa04918 | Thyroid hormone synthesis | 0.01 | 2.5 | 2.9 | 9 | ADCY8\|ASGR2\|ATP1A4\|ATP1B2\|HSPA5\|PRKCB\|PRKCG\|SLC5A5\|TPO |
| Reactome pathways | R-HSA-112316 | Neuronal System | 1E-11 | 2.7 | 8 | 54 | ACHE\|ACTN2\|ADCY8\|CACNA1B\|CACNA1E\|CHRNA2\|CHRNA4\|CHRNA5\|CHRNA7\|CHRNB4\|GABRR2\|GAD1\|GNAL\|GNG4\|GRIK1\|GRIK5\|GRIN2C\|KCNA1\|KCNC3\|KCND2\|KCNJ4\|KCNJ6\|KCNN2\|KCNN3\|KCNQ1\|KCNQ2\|KCNS1\|KCNS2\|NEFL\|PRKCB\|PRKCG\|RASGRF1\|SLC1A7\|SLC6A3\|SLC6A4\|SLC6A12\|SLC6A13\|GLRA3\|CHRNA6\|GABBR2\|HCN4\|KCNK7\|TUBB4A\|CPLX1\|IL1RAPL1\|KCNH3\|GRIP1\|KCNH5\|KCNK10\|GABRQ\|PANX2\|LRRC4B\|KCNG3\|TUBB2B |
|  | R-HSA-372790 | Signaling by GPCR | 1.58E-10 | 2.2 | 7.3 | 75 | ADCY8\|ADRA2B\|AVPR2\|BRS3\|BTK\|CALCA\|CCK\|CHRM1\|CHRM4\|CCR4\|CNR1\|CNR2\|CX3CR1\|DGKB\|DGKG\|HBEGF\|GPR183\|ADGRE1\|F2\|GAST\|GIPR\|GNAL\|GNG4\|GPR27\|MLNR\|GRM8\|IHH\|CXCR1\|CXCL10\|KEL\|MC5R\|CXCL9\|MMP3\|OPRK1\|P2RY1\|P2RY6\|PDE2A\|PIK3CG\|PRKCB\|PRKCG\|PTAFR\|PTGER3\|RGS2\|RGS4\|RGS16\|CCL3\|CCL7\|SHH\|SSTR2\|SSTR3\|TAC1\|TAC3\|VAV1\|VIP\|VIPR2\|WNT6\|CXCR4\|FZD9\|GALR2\|GPR37L1\|GPR55\|GABBR2\|RAMP1\|GPR83\|FZD10\|PIK3R5\|RGS22\|GPR132\|DHH\|GAL\|ACKR4\|LPAR5\|TAS1R1\|UCN2\|OPN5 |
|  | R-HSA-500792 | GPCR ligand binding | 1E-09 | 2.5 | 7.1 | 55 | ADRA2B\|AVPR2\|BRS3\|CALCA\|CCK\|CHRM1\|CHRM4\|CCR4\|CNR1\|CNR2\|CX3CR1\|GPR183\|ADGRE1\|F2\|GIPR\|GNG4\|MLNR\|GRM8\|IHH\|CXCR1\|CXCL10\|KEL\|MC5R\|CXCL9\|OPRK1\|P2RY1\|P2RY6\|PTAFR\|PTGER3\|CCL3\|CCL7\|SHH\|SSTR2\|SSTR3\|TAC1\|TAC3\|VIP\|VIPR2\|WNT6\|CXCR4\|FZD9\|GALR2\|GPR37L1\|GPR55\|GABBR2\|RAMP1\|FZD10\|GPR132\|DHH\|GAL\|ACKR4\|LPAR5\|TAS1R1\|UCN2\|OPN5 |
|  | R-HSA-73728 | RNA Polymerase I Promoter Opening | 3.16E-08 | 5.3 | 7.6 | 16 | H2AC8\|H2BC5\|H2BC3\|H2AC18\|H2BC14\|H2BC17\|H3C1\|H3C3\|H3C12\|H4C4\|H4C8\|H4C5\|H3C14\|H3C15\|H3C13\|H2AC19 |
|  | R-HSA-112315 | Transmission across Chemical Synapses | 3.16E-08 | 2.8 | 6.6 | 36 | ACHE\|ACTN2\|ADCY8\|CACNA1B\|CACNA1E\|CHRNA2\|CHRNA4\|CHRNA5\|CHRNA7\|CHRNB4\|GABRR2\|GAD1\|GNAL\|GNG4\|GRIK1\|GRIK5\|GRIN2C\|KCNJ4\|KCNJ6\|NEFL\|PRKCB\|PRKCG\|RASGRF1\|SLC1A7\|SLC6A3\|SLC6A4\|SLC6A12\|SLC6A13\|GLRA3\|CHRNA6\|GABBR2\|TUBB4A\|CPLX1\|GRIP1\|GABRQ\|TUBB2B |
|  | R-HSA-9616222 | Transcriptional regulation of granulopoiesis | 3.98E-08 | 4.4 | 7.2 | 19 | CEBPA\|GATA2\|H2AC8\|H2BC5\|H2BC3\|SPI1\|H2AC18\|H2BC14\|H2BC17\|H3C1\|H3C3\|H3C12\|H4C4\|H4C8\|H4C5\|H3C14\|H3C15\|H3C13\|H2AC19 |
|  | R-HSA-388396 | GPCR downstream signalling | 3.98E-08 | 2.1 | 6.1 | 63 | ADCY8\|ADRA2B\|AVPR2\|BRS3\|BTK\|CALCA\|CCK\|CHRM1\|CHRM4\|CCR4\|CNR1\|CNR2\|CX3CR1\|DGKB\|DGKG\|HBEGF\|GPR183\|F2\|GAST\|GIPR\|GNAL\|GNG4\|GPR27\|MLNR\|GRM8\|CXCR1\|CXCL10\|MC5R\|CXCL9\|MMP3\|OPRK1\|P2RY1\|P2RY6\|PDE2A\|PIK3CG\|PRKCB\|PRKCG\|PTAFR\|PTGER3\|RGS2\|RGS4\|RGS16\|SSTR2\|SSTR3\|TAC1\|TAC3\|VAV1\|VIP\|VIPR2\|CXCR4\|GALR2\|GPR37L1\|GPR55\|GABBR2\|RAMP1\|GPR83\|PIK3R5\|RGS22\|GPR132\|GAL\|LPAR5\|TAS1R1\|OPN5 |
|  | R-HSA-5334118 | DNA methylation | 5.01E-08 | 5.1 | 7.5 | 16 | H2AC8\|H2BC5\|H2BC3\|H2AC18\|H2BC14\|H2BC17\|H3C1\|H3C3\|H3C12\|H4C4\|H4C8\|H4C5\|H3C14\|H3C15\|H3C13\|H2AC19 |
|  | R-HSA-2299718 | Condensation of Prophase Chromosomes | 6.31E-08 | 4.8 | 7.3 | 17 | H2AC8\|H2BC5\|H2BC3\|PLK1\|H2AC18\|H2BC14\|H2BC17\|H3C1\|H3C3\|H3C12\|H4C4\|H4C8\|H4C5\|H3C14\|H3C15\|H3C13\|H2AC19 |
|  | R-HSA-5625886 | Activated PKN1 stimulates transcription of AR (androgen receptor) regulated genes KLK2 and KLK3 | 7.94E-08 | 5 | 7.3 | 16 | H2AC8\|H2BC5\|H2BC3\|H2AC18\|H2BC14\|H2BC17\|H3C1\|H3C3\|H3C12\|H4C4\|H4C8\|H4C5\|H3C14\|H3C15\|H3C13\|H2AC19 |
|  | R-HSA-427389 | ERCC6 (CSB) and EHMT2 (G9a) positively regulate rRNA expression | 1E-07 | 4.7 | 7.2 | 17 | ERCC6\|H2AC8\|H2BC5\|H2BC3\|H2AC18\|H2BC14\|H2BC17\|H3C1\|H3C3\|H3C12\|H4C4\|H4C8\|H4C5\|H3C14\|H3C15\|H3C13\|H2AC19 |
|  | R-HSA-427359 | SIRT1 negatively regulates rRNA expression | 1E-07 | 4.9 | 7.2 | 16 | H2AC8\|H2BC5\|H2BC3\|H2AC18\|H2BC14\|H2BC17\|H3C1\|H3C3\|H3C12\|H4C4\|H4C8\|H4C5\|H3C14\|H3C15\|H3C13\|H2AC19 |
|  | R-HSA-68616 | Assembly of the ORC complex at the origin of replication | 1.26E-07 | 4.8 | 7.1 | 16 | H2AC8\|H2BC5\|H2BC3\|H2AC18\|H2BC14\|H2BC17\|H3C1\|H3C3\|H3C12\|H4C4\|H4C8\|H4C5\|H3C14\|H3C15\|H3C13\|H2AC19 |
|  | R-HSA-9710421 | Defective pyroptosis | 3.16E-07 | 4.6 | 6.8 | 16 | H2AC8\|H2BC5\|H2BC3\|H2AC18\|H2BC14\|H2BC17\|H3C1\|H3C3\|H3C12\|H4C4\|H4C8\|H4C5\|H3C14\|H3C15\|H3C13\|H2AC19 |
|  | R-HSA-212300 | PRC2 methylates histones and DNA | 3.16E-07 | 4.6 | 6.8 | 16 | H2AC8\|H2BC5\|H2BC3\|H2AC18\|H2BC14\|H2BC17\|H3C1\|H3C3\|H3C12\|H4C4\|H4C8\|H4C5\|H3C14\|H3C15\|H3C13\|H2AC19 |
|  | R-HSA-1296071 | Potassium Channels | 3.98E-07 | 3.8 | 6.5 | 19 | GNG4\|KCNA1\|KCNC3\|KCND2\|KCNJ4\|KCNJ6\|KCNN2\|KCNN3\|KCNQ1\|KCNQ2\|KCNS1\|KCNS2\|GABBR2\|HCN4\|KCNK7\|KCNH3\|KCNH5\|KCNK10\|KCNG3 |
|  | R-HSA-3214815 | HDACs deacetylate histones | 5.01E-07 | 4 | 6.5 | 18 | H2AC8\|H2BC5\|H2BC3\|H2AC16\|H2AC18\|H2BC14\|H2BC17\|H3C1\|H3C3\|H3C12\|H4C4\|H4C8\|H4C5\|H3C14\|H3C15\|H2BC18\|H3C13\|H2AC19 |
|  | R-HSA-912446 | Meiotic recombination | 6.31E-07 | 4.1 | 6.5 | 17 | H2AC8\|H2BC5\|H2BC3\|H2AC18\|H2BC14\|H2BC17\|H3C1\|H3C3\|H3C12\|H4C4\|H4C8\|H4C5\|DMC1\|H3C14\|H3C15\|H3C13\|H2AC19 |
|  | R-HSA-373076 | Class A/1 (Rhodopsin-like receptors) | 0.000001 | 2.4 | 5.6 | 38 | ADRA2B\|AVPR2\|BRS3\|CCK\|CHRM1\|CHRM4\|CCR4\|CNR1\|CNR2\|CX3CR1\|GPR183\|F2\|MLNR\|CXCR1\|CXCL10\|KEL\|MC5R\|CXCL9\|OPRK1\|P2RY1\|P2RY6\|PTAFR\|PTGER3\|CCL3\|CCL7\|SSTR2\|SSTR3\|TAC1\|TAC3\|CXCR4\|GALR2\|GPR37L1\|GPR55\|GPR132\|GAL\|ACKR4\|LPAR5\|OPN5 |
|  | R-HSA-5617472 | Activation of anterior HOX genes in hindbrain development during early embryogenesis | 1.26E-06 | 3.4 | 6 | 20 | EGR2\|H2AC8\|H2BC5\|H2BC3\|HOXD1\|PAX6\|H2AC18\|H2BC14\|H2BC17\|H3C1\|H3C3\|H3C12\|H4C4\|H4C8\|H4C5\|MAFB\|H3C14\|H3C15\|H3C13\|H2AC19 |
|  | R-HSA-5619507 | Activation of HOX genes during differentiation | 1.26E-06 | 3.4 | 6 | 20 | EGR2\|H2AC8\|H2BC5\|H2BC3\|HOXD1\|PAX6\|H2AC18\|H2BC14\|H2BC17\|H3C1\|H3C3\|H3C12\|H4C4\|H4C8\|H4C5\|MAFB\|H3C14\|H3C15\|H3C13\|H2AC19 |
|  | R-HSA-201722 | Formation of the beta-catenin:TCF transactivating complex | 1.26E-06 | 3.9 | 6.2 | 17 | RUNX3\|H2AC8\|H2BC5\|H2BC3\|H2AC18\|H2BC14\|H2BC17\|H3C1\|H3C3\|H3C12\|H4C4\|H4C8\|H4C5\|H3C14\|H3C15\|H3C13\|H2AC19 |
|  | R-HSA-5250924 | B-WICH complex positively regulates rRNA expression | 1.26E-06 | 3.9 | 6.2 | 17 | ERCC6\|H2AC8\|H2BC5\|H2BC3\|H2AC18\|H2BC14\|H2BC17\|H3C1\|H3C3\|H3C12\|H4C4\|H4C8\|H4C5\|H3C14\|H3C15\|H3C13\|H2AC19 |
|  | R-HSA-425407 | SLC-mediated transmembrane transport | 1.58E-06 | 2.6 | 5.6 | 31 | SLC1A7\|SLC2A1\|SLC2A4\|SLC3A2\|SLC4A1\|SLC5A5\|SLC6A3\|SLC6A7\|SLC6A12\|SLC6A13\|SLC8A2\|SLC8A3\|SLC12A1\|SLC14A1\|SLC30A3\|SLC7A5\|SLCO2B1\|SLC24A2\|RHCG\|SLC30A10\|SLC7A10\|RHBG\|SLC7A3\|SLC22A16\|SLC26A9\|SLC16A10\|SLC5A10\|SLC2A14\|SLC5A9\|SLC44A5\|SLC13A5 |
|  | R-HSA-2559582 | Senescence-Associated Secretory Phenotype (SASP) | 1.58E-06 | 3.5 | 6 | 19 | CCNA2\|FOS\|H2AC8\|H2BC5\|H2BC3\|H2AC18\|H2BC14\|H2BC17\|H3C1\|H3C3\|H3C12\|H4C4\|H4C8\|H4C5\|CCNA1\|H3C14\|H3C15\|H3C13\|H2AC19 |
|  | R-HSA-9645723 | Diseases of programmed cell death | 2E-06 | 3.6 | 6 | 18 | FASLG\|H2AC8\|H2BC5\|H2BC3\|LMNB1\|H2AC18\|H2BC14\|H2BC17\|H3C1\|H3C3\|H3C12\|H4C4\|H4C8\|H4C5\|H3C14\|H3C15\|H3C13\|H2AC19 |
|  | R-HSA-9609690 | HCMV Early Events | 2E-06 | 3.2 | 5.9 | 21 | DYNC1I1\|H2AC8\|H2BC5\|H2BC3\|H2AC16\|H2AC18\|H2BC14\|H2BC17\|H3C1\|H3C3\|H3C12\|H4C4\|H4C8\|H4C5\|TUBB4A\|H3C14\|H3C15\|TUBB2B\|H2BC18\|H3C13\|H2AC19 |
|  | R-HSA-5625740 | RHO GTPases activate PKNs | 2.51E-06 | 3.7 | 6 | 17 | H2AC8\|H2BC5\|H2BC3\|H2AC18\|H2BC14\|H2BC17\|H3C1\|H3C3\|H3C12\|H4C4\|H4C8\|H4C5\|MYH14\|H3C14\|H3C15\|H3C13\|H2AC19 |
|  | R-HSA-9610379 | HCMV Late Events | 2.51E-06 | 3.4 | 5.8 | 19 | H2AC8\|H2BC5\|H2BC3\|H2AC16\|H2AC18\|H2BC14\|H2BC17\|H3C1\|H3C3\|H3C12\|H4C4\|H4C8\|H4C5\|H3C14\|H3C15\|H2BC18\|H3C13\|H2AC19\|RNF103-CHMP3 |
|  | R-HSA-1500620 | Meiosis | 3.16E-06 | 3.4 | 5.7 | 19 | H2AC8\|H2BC5\|H2BC3\|HSPA2\|LMNB1\|H2AC18\|H2BC14\|H2BC17\|H3C1\|H3C3\|H3C12\|H4C4\|H4C8\|H4C5\|DMC1\|H3C14\|H3C15\|H3C13\|H2AC19 |
|  | R-HSA-8939211 | ESR-mediated signaling | 3.16E-06 | 2.6 | 5.4 | 28 | HBEGF\|EREG\|FKBP4\|FOS\|FOSB\|GNG4\|H2AC8\|H2BC5\|H2BC3\|FOXA1\|HSPB1\|HSP90AA1\|MMP3\|H2AC18\|H2BC14\|H2BC17\|H3C1\|H3C3\|H3C12\|H4C4\|H4C8\|H4C5\|SPHK1\|H3C14\|EPGN\|H3C15\|H3C13\|H2AC19 |
|  | R-HSA-1474165 | Reproduction | 5.01E-06 | 3.1 | 5.5 | 21 | H2AC8\|H2BC5\|H2BC3\|HSPA2\|LMNB1\|ZP2\|H2AC18\|H2BC14\|H2BC17\|H3C1\|H3C3\|H3C12\|H4C4\|H4C8\|H4C5\|DMC1\|H3C14\|CATSPERD\|H3C15\|H3C13\|H2AC19 |
|  | R-HSA-109582 | Hemostasis | 5.01E-06 | 1.9 | 5 | 56 | ACTN2\|ADRA2B\|ANGPT2\|ATP1B2\|KIF1A\|CXADR\|DGKB\|DGKG\|F2\|GATA2\|GNG4\|HSPA5\|IGF1\|ITGA2\|ITGAL\|ITGAM\|LCK\|MMP1\|P2RX5\|P2RY1\|PDE2A\|PDGFA\|PIK3CG\|PLAU\|SERPINF2\|PRKCB\|PRKCG\|PSG7\|PTPN6\|RAB27B\|SLC3A2\|SLC8A2\|SLC8A3\|VAV1\|SLC7A5\|H3C1\|H3C3\|H3C12\|DOK2\|KIF20A\|IRAG1\|TUBB4A\|MERTK\|KIF2C\|KIF21B\|PIK3R5\|KIF4A\|TREM1\|SLC7A10\|PCYOX1L\|H3C14\|KIF18B\|KLC3\|H3C15\|TUBB2B\|H3C13 |
|  | R-HSA-2559586 | DNA Damage/Telomere Stress Induced Senescence | 5.01E-06 | 3.9 | 5.8 | 15 | CCNA2\|H1-4\|H1-5\|H2AC8\|H2BC5\|H2BC3\|LMNB1\|H2AC18\|H2BC14\|H2BC17\|H4C4\|H4C8\|H4C5\|CCNA1\|H2AC19 |
|  | R-HSA-73772 | RNA Polymerase I Promoter Escape | 6.31E-06 | 3.7 | 5.7 | 16 | H2AC8\|H2BC5\|H2BC3\|H2AC18\|H2BC14\|H2BC17\|H3C1\|H3C3\|H3C12\|H4C4\|H4C8\|H4C5\|H3C14\|H3C15\|H3C13\|H2AC19 |
|  | R-HSA-112314 | Neurotransmitter receptors and postsynaptic signal transmission | 6.31E-06 | 2.6 | 5.3 | 26 | ACTN2\|ADCY8\|CHRNA2\|CHRNA4\|CHRNA5\|CHRNA7\|CHRNB4\|GABRR2\|GNAL\|GNG4\|GRIK1\|GRIK5\|GRIN2C\|KCNJ4\|KCNJ6\|NEFL\|PRKCB\|PRKCG\|RASGRF1\|GLRA3\|CHRNA6\|GABBR2\|TUBB4A\|GRIP1\|GABRQ\|TUBB2B |
|  | R-HSA-1912408 | Pre-NOTCH Transcription and Translation | 7.94E-06 | 3.6 | 5.6 | 16 | H2AC8\|H2BC5\|H2BC3\|H2AC18\|H2BC14\|H2BC17\|H3C1\|H3C3\|H3C12\|H4C4\|H4C8\|H4C5\|H3C14\|H3C15\|H3C13\|H2AC19 |
|  | R-HSA-629594 | Highly calcium permeable postsynaptic nicotinic acetylcholine receptors | 7.94E-06 | 10 | 7.3 | 6 | CHRNA2\|CHRNA4\|CHRNA5\|CHRNA7\|CHRNB4\|CHRNA6 |
|  | R-HSA-9609646 | HCMV Infection | 0.00001 | 2.9 | 5.3 | 22 | DYNC1I1\|H2AC8\|H2BC5\|H2BC3\|H2AC16\|H2AC18\|H2BC14\|H2BC17\|H3C1\|H3C3\|H3C12\|H4C4\|H4C8\|H4C5\|TUBB4A\|H3C14\|H3C15\|TUBB2B\|H2BC18\|H3C13\|H2AC19\|RNF103-CHMP3 |
|  | R-HSA-3371571 | HSF1-dependent transactivation | 0.00001 | 6.9 | 6.5 | 8 | CRYAB\|FKBP4\|HSPA1A\|HSPA1B\|HSPA2\|HSP90AA1\|DNAJB1\|HSPB8 |
|  | R-HSA-9018519 | Estrogen-dependent gene expression | 0.00001 | 2.9 | 5.3 | 21 | FKBP4\|FOS\|FOSB\|H2AC8\|H2BC5\|H2BC3\|FOXA1\|HSP90AA1\|H2AC18\|H2BC14\|H2BC17\|H3C1\|H3C3\|H3C12\|H4C4\|H4C8\|H4C5\|H3C14\|H3C15\|H3C13\|H2AC19 |
|  | R-HSA-9031628 | NGF-stimulated transcription | 1.26E-05 | 5.3 | 6.1 | 10 | ASCL1\|EGR2\|EGR3\|FOS\|FOSB\|RRAD\|SH3GL3\|VGF\|TRIB1\|ARC |
|  | R-HSA-5250913 | Positive epigenetic regulation of rRNA expression | 1.26E-05 | 3.3 | 5.4 | 17 | ERCC6\|H2AC8\|H2BC5\|H2BC3\|H2AC18\|H2BC14\|H2BC17\|H3C1\|H3C3\|H3C12\|H4C4\|H4C8\|H4C5\|H3C14\|H3C15\|H3C13\|H2AC19 |
|  | R-HSA-8936459 | RUNX1 regulates genes involved in megakaryocyte differentiation and platelet function | 1.58E-05 | 3.4 | 5.4 | 16 | H2AC8\|H2BC5\|H2BC3\|H2AC18\|H2BC14\|H2BC17\|H3C1\|H3C3\|H3C12\|H4C4\|H4C8\|H4C5\|H3C14\|H3C15\|H3C13\|H2AC19 |
|  | R-HSA-8939236 | RUNX1 regulates transcription of genes involved in differentiation of HSCs | 1.58E-05 | 3 | 5.2 | 19 | GATA2\|H2AC8\|H2BC5\|H2BC3\|SPI1\|TP73\|H2AC18\|H2BC14\|H2BC17\|H3C1\|H3C3\|H3C12\|H4C4\|H4C8\|H4C5\|H3C14\|H3C15\|H3C13\|H2AC19 |
|  | R-HSA-5576891 | Cardiac conduction | 2E-05 | 3 | 5.2 | 19 | ATP1A4\|ATP1B2\|NKX2-5\|KCND2\|KCNJ4\|KCNQ1\|NPPC\|RYR1\|RYR2\|SCN1A\|SCN2A\|SCN4A\|SCN10A\|SLC8A2\|SLC8A3\|KCNK5\|KCNK7\|KCNE4\|KCNK10 |
|  | R-HSA-73854 | RNA Polymerase I Promoter Clearance | 2E-05 | 3.2 | 5.2 | 17 | ERCC6\|H2AC8\|H2BC5\|H2BC3\|H2AC18\|H2BC14\|H2BC17\|H3C1\|H3C3\|H3C12\|H4C4\|H4C8\|H4C5\|H3C14\|H3C15\|H3C13\|H2AC19 |
|  | R-HSA-195258 | RHO GTPase Effectors | 2E-05 | 2.2 | 4.8 | 34 | BTK\|BUB1B\|DYNC1I1\|H2AC8\|H2BC5\|H2BC3\|NCF2\|PLK1\|PRKCB\|H2AC18\|H2BC14\|H2BC17\|H3C1\|H3C3\|H3C12\|H4C4\|H4C8\|H4C5\|TUBB4A\|BAIAP2\|KIF2C\|KNL1\|MYH14\|NUF2\|RHPN2\|H3C14\|IQGAP3\|KLC3\|SPC24\|SKA1\|H3C15\|TUBB2B\|H3C13\|H2AC19 |
|  | R-HSA-977225 | Amyloid fiber formation | 2E-05 | 3.2 | 5.2 | 17 | CALCA\|H2AC8\|H2BC5\|H2BC3\|H2AC18\|H2BC14\|H2BC17\|H3C1\|H3C3\|H3C12\|H4C4\|H4C8\|H4C5\|H3C14\|H3C15\|H3C13\|H2AC19 |
|  | R-HSA-198933 | Immunoregulatory interactions between a Lymphoid and a non-Lymphoid cell | 2.51E-05 | 3 | 5.1 | 19 | CD1D\|CD34\|COL2A1\|CXADR\|FCGR1A\|HLA-G\|ITGAL\|CD300C\|CD300A\|SIGLEC9\|TREM1\|SLAMF7\|ULBP1\|SIGLEC11\|SLAMF6\|CD300LB\|CD200R1\|CD300LG\|TREML4 |
|  | R-HSA-73864 | RNA Polymerase I Transcription | 2.51E-05 | 3.2 | 5.1 | 17 | ERCC6\|H2AC8\|H2BC5\|H2BC3\|H2AC18\|H2BC14\|H2BC17\|H3C1\|H3C3\|H3C12\|H4C4\|H4C8\|H4C5\|H3C14\|H3C15\|H3C13\|H2AC19 |
|  | R-HSA-3371568 | Attenuation phase | 2.51E-05 | 8.9 | 6.7 | 6 | FKBP4\|HSPA1A\|HSPA1B\|HSPA2\|HSP90AA1\|DNAJB1 |
|  | R-HSA-629597 | Highly calcium permeable nicotinic acetylcholine receptors | 2.51E-05 | 12 | 7.1 | 5 | CHRNA2\|CHRNA4\|CHRNA5\|CHRNB4\|CHRNA6 |
|  | R-HSA-1296072 | Voltage gated Potassium channels | 3.16E-05 | 4.8 | 5.7 | 10 | KCNA1\|KCNC3\|KCND2\|KCNQ1\|KCNQ2\|KCNS1\|KCNS2\|KCNH3\|KCNH5\|KCNG3 |
|  | R-HSA-2559580 | Oxidative Stress Induced Senescence | 3.16E-05 | 3 | 5 | 18 | FOS\|H2AC8\|H2BC5\|H2BC3\|H2AC18\|H2BC14\|H2BC17\|H3C1\|H3C3\|H3C12\|H4C4\|H4C8\|H4C5\|CBX4\|H3C14\|H3C15\|H3C13\|H2AC19 |
|  | R-HSA-210745 | Regulation of gene expression in beta cells | 3.98E-05 | 6.9 | 6.1 | 7 | FOXA3\|HNF4G\|NEUROD1\|NKX6-1\|PAX6\|HNF1A\|MAFA |
|  | R-HSA-375276 | Peptide ligand-binding receptors | 3.98E-05 | 2.5 | 4.7 | 24 | AVPR2\|BRS3\|CCK\|CCR4\|CX3CR1\|F2\|MLNR\|CXCR1\|CXCL10\|KEL\|MC5R\|CXCL9\|OPRK1\|CCL3\|CCL7\|SSTR2\|SSTR3\|TAC1\|TAC3\|CXCR4\|GALR2\|GPR37L1\|GAL\|ACKR4 |
|  | R-HSA-181431 | Acetylcholine binding and downstream events | 3.98E-05 | 8.3 | 6.4 | 6 | CHRNA2\|CHRNA4\|CHRNA5\|CHRNA7\|CHRNB4\|CHRNA6 |
|  | R-HSA-622327 | Postsynaptic nicotinic acetylcholine receptors | 3.98E-05 | 8.3 | 6.4 | 6 | CHRNA2\|CHRNA4\|CHRNA5\|CHRNA7\|CHRNB4\|CHRNA6 |
|  | R-HSA-5578749 | Transcriptional regulation by small RNAs | 5.01E-05 | 3.1 | 5 | 16 | H2AC8\|H2BC5\|H2BC3\|H2AC18\|H2BC14\|H2BC17\|H3C1\|H3C3\|H3C12\|H4C4\|H4C8\|H4C5\|H3C14\|H3C15\|H3C13\|H2AC19 |
