# Supplementary material for: Exploring the mechanism of Celastrol in the treatment of rheumatoid arthritis based on systems pharmacology and multi-omics
Source: Sci Rep. 2024 Jan 18;14:1604. doi: 10.1038/s41598-023-48248-5 (PMC10796403; doi:10.1038/s41598-023-48248-5)
Supplement: Supplementary file 4 — Supplementary Table S3. [file 41598_2023_48248_MOESM4_ESM.docx]

Table S3 Enrichment analysis of all differentially expressed proteins

| Category | GO | Description | Pvalue | Enrichment | Z-score | Counts | Proteins |
| --- | --- | --- | --- | --- | --- | --- | --- |
| GO Biological Processes | GO:0006457 | protein folding | 1E-25 | 14 | 19 | 30 | CRYAB\|FKBP1A\|FKBP2\|FKBP4\|PDIA3\|DNAJA1\|HSPA1A\|HSPA6\|HSPA8\|HSPA9\|HSPB1\|HSP90AA1\|HSPD1\|HSPE1\|DNAJB1\|PPIA\|PPIB\|PPIC\|QSOX1\|DNAJC7\|AIP\|MPDU1\|BAG3\|DNAJB6\|HSPH1\|STIP1\|DNAJB4\|HSPA4L\|CHORDC1\|DNAJB12 |
|  | GO:0061077 | chaperone-mediated protein folding | 1E-19 | 25 | 20 | 17 | FKBP1A\|FKBP2\|FKBP4\|HSPA1A\|HSPA6\|HSPA8\|HSPA9\|HSPB1\|HSPE1\|DNAJB1\|PPIB\|DNAJC7\|DNAJB6\|HSPH1\|DNAJB4\|CHORDC1\|DNAJB12 |
|  | GO:0006986 | response to unfolded protein | 1E-18 | 17 | 17 | 20 | COMP\|DNAJA1\|HSPA1A\|HSPA6\|HSPA8\|HSPA9\|HSPB1\|HSP90AA1\|HSPD1\|HSPE1\|DNAJB1\|PTPN1\|THBS1\|MANF\|BAG3\|MFN2\|OPTN\|HSPH1\|DNAJB4\|HSPA4L |
|  | GO:0035966 | response to topologically incorrect protein | 1E-18 | 15 | 17 | 21 | COMP\|DNAJA1\|HSPA1A\|HSPA6\|HSPA8\|HSPA9\|HSPB1\|HSP90AA1\|HSPD1\|HSPE1\|DNAJB1\|PTPN1\|THBS1\|MANF\|BAG3\|MFN2\|OPTN\|HSPH1\|DNAJB4\|HSPA4L\|DNAJB12 |
|  | GO:0030198 | extracellular matrix organization | 1E-18 | 9.8 | 15 | 26 | APP\|COL1A1\|COL1A2\|COL2A1\|COL4A1\|COL5A1\|COMP\|CCN2\|CCN1\|LAMB1\|LAMB2\|LAMC1\|LOX\|LOXL2\|LUM\|MMP1\|MMP2\|MMP14\|NID1\|QSOX1\|PTX3\|TGFBI\|PXDN\|DNAJB6\|ABI3BP\|GREM1 |
|  | GO:0043062 | extracellular structure organization | 1E-18 | 9.8 | 14 | 26 | APP\|COL1A1\|COL1A2\|COL2A1\|COL4A1\|COL5A1\|COMP\|CCN2\|CCN1\|LAMB1\|LAMB2\|LAMC1\|LOX\|LOXL2\|LUM\|MMP1\|MMP2\|MMP14\|NID1\|QSOX1\|PTX3\|TGFBI\|PXDN\|DNAJB6\|ABI3BP\|GREM1 |
|  | GO:0045229 | external encapsulating structure organization | 1E-17 | 9.7 | 14 | 26 | APP\|COL1A1\|COL1A2\|COL2A1\|COL4A1\|COL5A1\|COMP\|CCN2\|CCN1\|LAMB1\|LAMB2\|LAMC1\|LOX\|LOXL2\|LUM\|MMP1\|MMP2\|MMP14\|NID1\|QSOX1\|PTX3\|TGFBI\|PXDN\|DNAJB6\|ABI3BP\|GREM1 |
|  | GO:0006458 | 'de novo' protein folding | 1E-14 | 30 | 19 | 12 | FKBP1A\|HSPA1A\|HSPA6\|HSPA8\|HSPA9\|HSPD1\|HSPE1\|DNAJB1\|DNAJC7\|HSPH1\|DNAJB4\|DNAJB12 |
|  | GO:0042026 | protein refolding | 1E-14 | 41 | 20 | 10 | CRYAB\|FKBP1A\|DNAJA1\|HSPA1A\|HSPA6\|HSPA8\|HSPA9\|HSPB1\|HSP90AA1\|HSPD1 |
|  | GO:0009408 | response to heat | 1E-13 | 15 | 14 | 15 | CRYAB\|HMOX1\|HSBP1\|DNAJA1\|HSPA1A\|HSPA6\|HSPB1\|HSP90AA1\|DNAJB1\|IGFBP7\|RBBP7\|THBS1\|BAG3\|DNAJB4\|HIKESHI |
|  | GO:0009611 | response to wounding | 1E-13 | 6.1 | 11 | 26 | ACTB\|SERPING1\|COL1A1\|COL5A1\|COMP\|CCN2\|F3\|FN1\|HMOX1\|HSPB1\|TNC\|CCN1\|LAMB2\|LOX\|SMAD3\|MMP2\|SERPINE2\|PLAUR\|PPIA\|SDC1\|THBD\|TIMP1\|TPM1\|NRP1\|PDPN\|VKORC1 |
|  | GO:0051085 | chaperone cofactor-dependent protein refolding | 1E-12 | 32 | 18 | 10 | HSPA1A\|HSPA6\|HSPA8\|HSPA9\|HSPE1\|DNAJB1\|DNAJC7\|HSPH1\|DNAJB4\|DNAJB12 |
|  | GO:0051084 | 'de novo' post-translational protein folding | 1E-12 | 28 | 16 | 10 | HSPA1A\|HSPA6\|HSPA8\|HSPA9\|HSPE1\|DNAJB1\|DNAJC7\|HSPH1\|DNAJB4\|DNAJB12 |
|  | GO:0052547 | regulation of peptidase activity | 1E-11 | 5.6 | 9.9 | 25 | BIN1\|APP\|SERPING1\|CRYAB\|CST3\|CCN2\|DAP\|ECM1\|F3\|FN1\|HSPD1\|HSPE1\|CCN1\|SMAD3\|SERPINE1\|PCOLCE\|SERPINE2\|PLAUR\|PRNP\|THBS1\|TIMP1\|TIMP2\|DNAJB6\|TMED10\|RCN3 |
|  | GO:0042060 | wound healing | 1E-11 | 6.5 | 10 | 21 | ACTB\|SERPING1\|COL1A1\|COL5A1\|COMP\|F3\|FN1\|HMOX1\|HSPB1\|CCN1\|LOX\|SMAD3\|SERPINE2\|PLAUR\|PPIA\|SDC1\|THBD\|TIMP1\|TPM1\|PDPN\|VKORC1 |
|  | GO:0009266 | response to temperature stimulus | 1E-11 | 9.5 | 11 | 16 | CRYAB\|HMOX1\|HSBP1\|DNAJA1\|HSPA1A\|HSPA6\|HSPB1\|HSP90AA1\|HSPD1\|DNAJB1\|IGFBP7\|RBBP7\|THBS1\|BAG3\|DNAJB4\|HIKESHI |
|  | GO:0001503 | ossification | 1E-10 | 7 | 9.9 | 19 | COL1A1\|COL1A2\|COL2A1\|COMP\|CCN2\|ECM1\|HSPE1\|TNC\|IGFBP3\|IGFBP5\|CCN1\|LOX\|SMAD3\|MMP2\|MMP14\|TPM4\|DNAJC13\|GTPBP4\|CTHRC1 |
|  | GO:0030162 | regulation of proteolysis | 1E-10 | 4.1 | 8.6 | 30 | BIN1\|APP\|SERPING1\|CRYAB\|CSNK1A1\|CST3\|CCN2\|DAP\|ECM1\|F3\|FN1\|HSPA1A\|HSPD1\|HSPE1\|CCN1\|SMAD3\|MMP14\|SERPINE1\|PCOLCE\|SERPINE2\|PLAUR\|PRNP\|THBS1\|TIMP1\|TIMP2\|DNAJB6\|TMED10\|GABARAPL2\|RCN3\|SH3D19 |
|  | GO:0097435 | supramolecular fiber organization | 1E-10 | 4.8 | 8.9 | 26 | APP\|RND3\|COL1A1\|COL1A2\|COL2A1\|COL5A1\|COMP\|CST3\|FAT1\|FKBP1A\|ITGB5\|LOX\|LOXL2\|LTBP2\|LUM\|TMSB4X\|TNFAIP6\|TPM1\|TPM2\|TPM4\|ZYX\|PXDN\|ABI1\|DNAJB6\|GREM1\|FHOD1 |
|  | GO:0001944 | vasculature development | 1E-10 | 4.9 | 8.9 | 25 | COL1A1\|COL1A2\|COL4A1\|COL5A1\|COMP\|CCN2\|ECM1\|FN1\|HMOX1\|CCN1\|LOX\|LOXL2\|MFGE8\|MMP2\|MMP14\|SERPINE1\|PRRX1\|TGFBI\|THBS1\|PXDN\|NRP1\|PDPN\|GREM1\|AMOTL2\|JAM3 |
|  | GO:0034605 | cellular response to heat | 1E-10 | 20 | 13 | 10 | HMOX1\|HSBP1\|HSPA1A\|HSPA6\|HSP90AA1\|DNAJB1\|RBBP7\|THBS1\|BAG3\|HIKESHI |
|  | GO:0001568 | blood vessel development | 2E-10 | 4.9 | 8.8 | 24 | COL1A1\|COL1A2\|COL4A1\|COL5A1\|COMP\|CCN2\|ECM1\|FN1\|HMOX1\|CCN1\|LOX\|LOXL2\|MFGE8\|MMP2\|MMP14\|SERPINE1\|PRRX1\|TGFBI\|THBS1\|PXDN\|NRP1\|GREM1\|AMOTL2\|JAM3 |
|  | GO:0030199 | collagen fibril organization | 2E-10 | 18 | 13 | 10 | COL1A1\|COL1A2\|COL2A1\|COL5A1\|COMP\|LOX\|LOXL2\|LUM\|PXDN\|GREM1 |
|  | GO:0030335 | positive regulation of cell migration | 3.16E-10 | 4.6 | 8.5 | 25 | APP\|COL1A1\|F3\|FN1\|HMOX1\|HSPB1\|IGFBP5\|CCN1\|LAMB1\|LAMC2\|LGALS3\|SMAD3\|MMP2\|MMP14\|SERPINE1\|MAP2K3\|SPARC\|THBS1\|TMSB4X\|TNFAIP6\|NUMB\|NRP1\|PDPN\|CEMIP\|JAM3 |
|  | GO:2000147 | positive regulation of cell motility | 7.94E-10 | 4.4 | 8.2 | 25 | APP\|COL1A1\|F3\|FN1\|HMOX1\|HSPB1\|IGFBP5\|CCN1\|LAMB1\|LAMC2\|LGALS3\|SMAD3\|MMP2\|MMP14\|SERPINE1\|MAP2K3\|SPARC\|THBS1\|TMSB4X\|TNFAIP6\|NUMB\|NRP1\|PDPN\|CEMIP\|JAM3 |
|  | GO:0048514 | blood vessel morphogenesis | 1E-09 | 5.2 | 8.5 | 21 | COL4A1\|COMP\|CCN2\|ECM1\|FN1\|HMOX1\|CCN1\|LOX\|LOXL2\|MFGE8\|MMP2\|MMP14\|SERPINE1\|PRRX1\|TGFBI\|THBS1\|PXDN\|NRP1\|GREM1\|AMOTL2\|JAM3 |
|  | GO:0040017 | positive regulation of locomotion | 1.58E-09 | 4.3 | 8 | 25 | APP\|COL1A1\|F3\|FN1\|HMOX1\|HSPB1\|IGFBP5\|CCN1\|LAMB1\|LAMC2\|LGALS3\|SMAD3\|MMP2\|MMP14\|SERPINE1\|MAP2K3\|SPARC\|THBS1\|TMSB4X\|TNFAIP6\|NUMB\|NRP1\|PDPN\|CEMIP\|JAM3 |
|  | GO:0052548 | regulation of endopeptidase activity | 1.58E-09 | 5.1 | 8.4 | 21 | BIN1\|APP\|SERPING1\|CRYAB\|CST3\|CCN2\|DAP\|F3\|HSPD1\|HSPE1\|CCN1\|SMAD3\|SERPINE1\|SERPINE2\|PLAUR\|PRNP\|THBS1\|TIMP1\|TIMP2\|DNAJB6\|TMED10 |
|  | GO:0001525 | angiogenesis | 3.16E-09 | 5.7 | 8.5 | 18 | COL4A1\|CCN2\|ECM1\|FN1\|HMOX1\|CCN1\|LOXL2\|MFGE8\|MMP2\|MMP14\|SERPINE1\|TGFBI\|THBS1\|PXDN\|NRP1\|GREM1\|AMOTL2\|JAM3 |
|  | GO:0007492 | endoderm development | 5.01E-09 | 13 | 11 | 10 | FN1\|HSBP1\|INHBA\|ITGB5\|LAMB1\|LAMC1\|SMAD3\|MMP2\|MMP14\|PELO |
|  | GO:0051346 | negative regulation of hydrolase activity | 5.01E-09 | 5.3 | 8.2 | 19 | BIN1\|APP\|SERPING1\|CRYAB\|CST3\|ECM1\|FKBP1A\|LGALS3\|SERPINE1\|SERPINE2\|PLAUR\|PRNP\|PTX3\|THBS1\|TIMP1\|TIMP2\|AIP\|DNAJB6\|TMED10 |
|  | GO:0033365 | protein localization to organelle | 7.94E-09 | 3.8 | 7.5 | 26 | COL1A1\|TIMM8A\|HK2\|DNAJA1\|HSPA8\|HSP90AA1\|HSPD1\|KPNA2\|SRP14\|RAB7A\|NRP1\|SQSTM1\|AIP\|MFN2\|DNAJB6\|OPTN\|TMED10\|PACSIN2\|GABARAPL2\|TIMM13\|HIKESHI\|MARCHF5\|EMC7\|SPCS3\|MFSD1\|NUP35 |
|  | GO:0009725 | response to hormone | 7.94E-09 | 3.7 | 7.4 | 27 | PARP1\|COL1A1\|CCN2\|FBN1\|FKBP4\|GNAI1\|NR3C1\|HMOX1\|DNAJA1\|HSPA1A\|HSPA8\|HSP90AA1\|IGFBP5\|IGFBP7\|INHBA\|LOX\|MMP2\|MMP14\|PTPN1\|RBBP7\|RPL27\|SDC1\|SLIT3\|STAT6\|THBS1\|TIMP1\|TIMP2 |
|  | GO:0001649 | osteoblast differentiation | 1.26E-08 | 9 | 9.3 | 12 | COL1A1\|HSPE1\|TNC\|IGFBP3\|IGFBP5\|CCN1\|LOX\|SMAD3\|TPM4\|DNAJC13\|GTPBP4\|CTHRC1 |
|  | GO:0006886 | intracellular protein transport | 1.58E-08 | 3.8 | 7.3 | 25 | TIMM8A\|HSPA8\|HSPA9\|HSPB1\|HSP90AA1\|HSPD1\|KPNA2\|LTBP2\|SRP14\|RAB7A\|NAPG\|SQSTM1\|AIP\|BAG3\|MFN2\|TMED10\|RAB18\|RAB21\|TIMM13\|GOLGA7\|HIKESHI\|WDR11\|SPCS3\|YIF1B\|NUP35 |
|  | GO:0006979 | response to oxidative stress | 2E-08 | 5.1 | 7.8 | 18 | PARP1\|APP\|COL1A1\|CRYAB\|HMOX1\|HSPA1A\|MGST1\|MMP2\|MMP14\|PPIA\|PRNP\|PTGS1\|SDC1\|SOD3\|STAT6\|TPM1\|PXDN\|ZNF622 |
|  | GO:1900046 | regulation of hemostasis | 3.16E-08 | 13 | 10 | 9 | SERPING1\|COMP\|F3\|SERPINE1\|SERPINE2\|PLAUR\|THBD\|THBS1\|VKORC1 |
|  | GO:0061448 | connective tissue development | 3.16E-08 | 6.6 | 8.2 | 14 | BGN\|CHI3L1\|COL1A1\|COL2A1\|COL5A1\|COMP\|CCN2\|ECM1\|CCN1\|LOX\|PRRX1\|TGFBI\|TIMP1\|SPTLC2 |
|  | GO:0001558 | regulation of cell growth | 3.98E-08 | 4.7 | 7.5 | 19 | CRYAB\|DPYSL2\|FN1\|HSPA1A\|TNC\|IGFBP3\|IGFBP5\|IGFBP7\|INHBA\|SMAD3\|MMP14\|SERPINE2\|RBBP7\|SLIT3\|NRP1\|RAB21\|GREM1\|PLXNA3\|HDGFL2 |
|  | GO:0035239 | tube morphogenesis | 3.98E-08 | 3.7 | 7 | 24 | COL4A1\|COMP\|CCN2\|ECM1\|FN1\|NR3C1\|HMOX1\|CCN1\|LOX\|LOXL2\|SMAD3\|MFGE8\|MMP2\|MMP14\|SERPINE1\|PRRX1\|TGFBI\|THBS1\|PXDN\|NRP1\|GREM1\|AMOTL2\|JAM3\|CTHRC1 |
|  | GO:0010466 | negative regulation of peptidase activity | 5.01E-08 | 5.9 | 7.9 | 15 | BIN1\|APP\|SERPING1\|CRYAB\|CST3\|ECM1\|SERPINE1\|SERPINE2\|PLAUR\|PRNP\|THBS1\|TIMP1\|TIMP2\|DNAJB6\|TMED10 |
|  | GO:0045785 | positive regulation of cell adhesion | 6.31E-08 | 4.3 | 7.2 | 20 | ACTB\|ADA\|CDK6\|FN1\|HSPD1\|CCN1\|LAMB1\|LAMB2\|LAMC1\|SMAD3\|NID1\|PNP\|PLAUR\|TPM1\|NRP1\|SART1\|PDPN\|HSPH1\|ZBTB1\|ABI3BP |
|  | GO:0007167 | enzyme-linked receptor protein signaling pathway | 7.94E-08 | 3.7 | 6.9 | 23 | PARP1\|COL1A1\|COL1A2\|COL4A1\|COMP\|CCN2\|EFEMP1\|FKBP1A\|HSPB1\|IGFBP5\|INHBA\|ITGB5\|LOX\|LTBP2\|SMAD3\|MMP2\|PTPN1\|STAT6\|ZYX\|NRP1\|ABI1\|GREM1\|NCEH1 |
|  | GO:0043086 | negative regulation of catalytic activity | 7.94E-08 | 3.4 | 6.7 | 26 | BIN1\|APP\|SERPING1\|CRYAB\|CST3\|ECM1\|FKBP1A\|DNAJA1\|HSPB1\|LGALS3\|SERPINE1\|SERPINE2\|PLAUR\|PPIA\|PRNP\|PTPN1\|PTX3\|THBS1\|TIMP1\|TIMP2\|AIP\|DNAJB6\|NES\|TMED10\|CHORDC1\|ITPRIP |
|  | GO:0070585 | protein localization to mitochondrion | 1E-07 | 11 | 9.3 | 9 | TIMM8A\|HK2\|DNAJA1\|HSP90AA1\|HSPD1\|AIP\|MFN2\|TIMM13\|MARCHF5 |
|  | GO:0007596 | blood coagulation | 1.26E-07 | 7.2 | 8.1 | 12 | ACTB\|SERPING1\|COMP\|F3\|FN1\|HSPB1\|SERPINE2\|PLAUR\|PPIA\|THBD\|PDPN\|VKORC1 |
|  | GO:0048545 | response to steroid hormone | 1.26E-07 | 5.5 | 7.5 | 15 | PARP1\|COL1A1\|CCN2\|FKBP4\|NR3C1\|DNAJA1\|HSPA1A\|HSPA8\|IGFBP7\|LOX\|RBBP7\|RPL27\|SDC1\|SLIT3\|THBS1 |
|  | GO:0031647 | regulation of protein stability | 1.26E-07 | 5.1 | 7.3 | 16 | CRYAB\|HSPA1A\|HSPA8\|HSP90AA1\|HSPD1\|SMAD3\|PPIB\|PRNP\|NAPG\|BAG3\|RAB21\|GTPBP4\|GOLGA7\|MFSD1\|RPAP3\|DDI2 |
|  | GO:0050817 | coagulation | 1.26E-07 | 7.1 | 8 | 12 | ACTB\|SERPING1\|COMP\|F3\|FN1\|HSPB1\|SERPINE2\|PLAUR\|PPIA\|THBD\|PDPN\|VKORC1 |
|  | GO:0032963 | collagen metabolic process | 1.58E-07 | 13 | 9.6 | 8 | COL1A1\|COL1A2\|COL5A1\|MMP1\|MMP2\|MMP14\|PCOLCE\|RCN3 |
|  | GO:0001501 | skeletal system development | 1.58E-07 | 4.1 | 6.9 | 20 | BGN\|CHI3L1\|COL1A1\|COL1A2\|COL2A1\|COMP\|CCN2\|ECM1\|FBN1\|CCN1\|SMAD3\|MMP2\|MMP14\|PRRX1\|PPIB\|TGFBI\|TIMP1\|EIF4A3\|ABI1\|VKORC1 |
|  | GO:0040013 | negative regulation of locomotion | 1.58E-07 | 4.5 | 7 | 18 | ADA\|DCN\|HMOX1\|IGFBP3\|IGFBP5\|SERPINE1\|RBBP7\|THBS1\|TIMP1\|TNFAIP6\|TPM1\|NRP1\|GTPBP4\|GREM1\|PLXNA3\|RAP2C\|PODN\|MICOS10-NBL1 |
|  | GO:0007599 | hemostasis | 1.58E-07 | 7 | 7.9 | 12 | ACTB\|SERPING1\|COMP\|F3\|FN1\|HSPB1\|SERPINE2\|PLAUR\|PPIA\|THBD\|PDPN\|VKORC1 |
|  | GO:0090092 | regulation of transmembrane receptor protein serine/threonine kinase signaling pathway | 1.58E-07 | 5.4 | 7.4 | 15 | PARP1\|FBN1\|FKBP1A\|HSPA1A\|CCN1\|INHBA\|LOX\|SMAD3\|RBBP7\|THBS1\|TNFAIP6\|FSTL1\|GREM1\|PELO\|MICOS10-NBL1 |
|  | GO:0050821 | protein stabilization | 2E-07 | 6.3 | 7.7 | 13 | CRYAB\|HSPA1A\|HSP90AA1\|HSPD1\|SMAD3\|PPIB\|NAPG\|BAG3\|RAB21\|GTPBP4\|GOLGA7\|MFSD1\|RPAP3 |
|  | GO:0010951 | negative regulation of endopeptidase activity | 2E-07 | 5.8 | 7.5 | 14 | BIN1\|APP\|SERPING1\|CRYAB\|CST3\|SERPINE1\|SERPINE2\|PLAUR\|PRNP\|THBS1\|TIMP1\|TIMP2\|DNAJB6\|TMED10 |
|  | GO:0035987 | endodermal cell differentiation | 2E-07 | 16 | 10 | 7 | FN1\|HSBP1\|INHBA\|ITGB5\|LAMB1\|MMP2\|MMP14 |
|  | GO:0090287 | regulation of cellular response to growth factor stimulus | 2E-07 | 4.9 | 7.1 | 16 | DCN\|FBN1\|FKBP1A\|HSPA1A\|CCN1\|LOX\|SMAD3\|PTPN1\|RBBP7\|THBS1\|TNFAIP6\|NRP1\|FSTL1\|GREM1\|PELO\|MICOS10-NBL1 |
|  | GO:0010810 | regulation of cell-substrate adhesion | 3.16E-07 | 6.1 | 7.5 | 13 | CDK6\|COL1A1\|FN1\|CCN1\|SMAD3\|MMP14\|NID1\|SERPINE1\|THBS1\|NRP1\|PDPN\|ABI3BP\|GREM1 |
|  | GO:0030193 | regulation of blood coagulation | 3.98E-07 | 12 | 9 | 8 | SERPING1\|F3\|SERPINE1\|SERPINE2\|PLAUR\|THBD\|THBS1\|VKORC1 |
| KEGG Pathway | hsa04512 | ECM-receptor interaction | 1E-16 | 19 | 17 | 16 | COL1A1\|COL1A2\|COL2A1\|COL4A1\|COMP\|FN1\|TNC\|ITGB5\|LAMA4\|LAMB1\|LAMB2\|LAMC1\|LAMC2\|SDC1\|THBS1\|THBS2 |
|  | hsa05165 | Human papillomavirus infection | 1E-11 | 6.6 | 10 | 21 | ATP6V1C1\|CDK6\|COL1A1\|COL1A2\|COL2A1\|COL4A1\|COMP\|CSNK1A1\|FN1\|TNC\|ITGB5\|LAMA4\|LAMB1\|LAMB2\|LAMC1\|LAMC2\|PPP2R2A\|RHEB\|THBS1\|THBS2\|ATP6V1H |
|  | hsa04510 | Focal adhesion | 1E-11 | 8.8 | 11 | 17 | ACTB\|COL1A1\|COL1A2\|COL2A1\|COL4A1\|COMP\|FN1\|TNC\|ITGB5\|LAMA4\|LAMB1\|LAMB2\|LAMC1\|LAMC2\|THBS1\|THBS2\|ZYX |
|  | hsa04151 | PI3K-Akt signaling pathway | 2E-09 | 5.6 | 8.5 | 19 | CDK6\|COL1A1\|COL1A2\|COL2A1\|COL4A1\|COMP\|FN1\|HSP90AA1\|TNC\|ITGB5\|LAMA4\|LAMB1\|LAMB2\|LAMC1\|LAMC2\|PPP2R2A\|RHEB\|THBS1\|THBS2 |
|  | hsa05146 | Amoebiasis | 5.01E-09 | 11 | 10 | 11 | COL1A1\|COL1A2\|COL4A1\|FN1\|HSPB1\|LAMA4\|LAMB1\|LAMB2\|LAMC1\|LAMC2\|RAB7A |
|  | hsa05145 | Toxoplasmosis | 1.58E-07 | 9.3 | 8.6 | 10 | GNAI1\|HSPA1A\|HSPA6\|HSPA8\|LAMA4\|LAMB1\|LAMB2\|LAMC1\|LAMC2\|MAP2K3 |
|  | hsa05171 | Coronavirus disease - COVID-19 | 5.01E-07 | 5.8 | 7.3 | 13 | C1R\|C1S\|MMP1\|RPL19\|RPL21\|RPL27\|RPL30\|RPL29\|RPLP2\|RPS12\|RPS27A\|NRP1\|MAVS |
|  | hsa04933 | AGE-RAGE signaling pathway in diabetic complications | 6.31E-07 | 9.3 | 8.2 | 9 | COL1A1\|COL1A2\|COL4A1\|F3\|FN1\|SMAD3\|MMP2\|SERPINE1\|THBD |
|  | hsa04141 | Protein processing in endoplasmic reticulum | 0.000001 | 6.7 | 7.3 | 11 | CRYAB\|PDIA3\|DNAJA1\|HSPA1A\|HSPA6\|HSPA8\|HSP90AA1\|DNAJB1\|HSPH1\|HSPA4L\|DNAJB12 |
|  | hsa04216 | Ferroptosis | 2.51E-06 | 15 | 9 | 6 | ACSL4\|HMOX1\|PRNP\|SLC3A2\|STEAP3\|MAP1LC3B |
|  | hsa05222 | Small cell lung cancer | 3.16E-06 | 9 | 7.6 | 8 | CDK6\|COL4A1\|FN1\|LAMA4\|LAMB1\|LAMB2\|LAMC1\|LAMC2 |
|  | hsa05205 | Proteoglycans in cancer | 5.01E-06 | 5.6 | 6.5 | 11 | ACTB\|COL1A1\|COL1A2\|DCN\|FN1\|ITGB5\|LUM\|MMP2\|PLAUR\|SDC1\|THBS1 |
|  | hsa04137 | Mitophagy - animal | 6.31E-06 | 10 | 7.6 | 7 | RPS27A\|RAB7A\|SQSTM1\|MFN2\|OPTN\|GABARAPL2\|MAP1LC3B |
|  | hsa04145 | Phagosome | 2E-05 | 6.1 | 6.3 | 9 | ACTB\|ATP6V1C1\|C1R\|COMP\|ITGB5\|THBS1\|THBS2\|RAB7A\|ATP6V1H |
|  | hsa04610 | Complement and coagulation cascades | 2E-05 | 8.5 | 6.9 | 7 | SERPING1\|C1R\|C1S\|F3\|SERPINE1\|PLAUR\|THBD |
|  | hsa03010 | Ribosome | 2.51E-05 | 5.9 | 6.1 | 9 | RPL19\|RPL21\|RPL27\|RPL30\|RPL29\|RPLP2\|RPS12\|RPS27A\|MRPS10 |
|  | hsa04350 | TGF-beta signaling pathway | 3.98E-05 | 7.7 | 6.4 | 7 | DCN\|FBN1\|INHBA\|SMAD3\|THBS1\|GREM1\|MICOS10-NBL1 |
|  | hsa05418 | Fluid shear stress and atherosclerosis | 6.31E-05 | 6 | 5.8 | 8 | ACTB\|HMOX1\|HSP90AA1\|MGST1\|MMP2\|SDC1\|THBD\|SQSTM1 |
|  | hsa05200 | Pathways in cancer | 6.31E-05 | 3.1 | 4.9 | 16 | CDK6\|COL4A1\|FN1\|GNAI1\|HMOX1\|HSP90AA1\|LAMA4\|LAMB1\|LAMB2\|LAMC1\|LAMC2\|SMAD3\|MGST1\|MMP1\|MMP2\|STAT6 |
|  | hsa05410 | Hypertrophic cardiomyopathy | 0.000251 | 6.9 | 5.5 | 6 | ACTB\|ITGB5\|MYL3\|TPM1\|TPM2\|TPM4 |
|  | hsa05414 | Dilated cardiomyopathy | 0.000316 | 6.5 | 5.3 | 6 | ACTB\|ITGB5\|MYL3\|TPM1\|TPM2\|TPM4 |
|  | hsa03015 | mRNA surveillance pathway | 0.000398 | 6.4 | 5.3 | 6 | PPP2R2A\|EIF4A3\|SAP18\|NXF1\|ACIN1\|PELO |
|  | hsa04915 | Estrogen signaling pathway | 0.000398 | 5.3 | 4.9 | 7 | FKBP4\|GNAI1\|HSPA1A\|HSPA6\|HSPA8\|HSP90AA1\|MMP2 |
|  | hsa04974 | Protein digestion and absorption | 0.000501 | 6 | 5.1 | 6 | COL1A1\|COL1A2\|COL2A1\|COL4A1\|COL5A1\|SLC3A2 |
|  | hsa03040 | Spliceosome | 0.000631 | 4.9 | 4.7 | 7 | HSPA1A\|HSPA6\|HSPA8\|SART1\|EIF4A3\|DDX42\|ACIN1 |
|  | hsa03013 | Nucleocytoplasmic transport | 0.000631 | 5.8 | 4.9 | 6 | KPNA2\|EIF4A3\|SAP18\|NXF1\|ACIN1\|NUP35 |
|  | hsa04115 | p53 signaling pathway | 0.000794 | 7.1 | 5.2 | 5 | CDK6\|IGFBP3\|SERPINE1\|THBS1\|STEAP3 |
|  | hsa04218 | Cellular senescence | 0.000794 | 4.7 | 4.5 | 7 | CDK6\|IGFBP3\|SMAD3\|SERPINE1\|MAP2K3\|RHEB\|SQSTM1 |
|  | hsa04612 | Antigen processing and presentation | 0.001 | 6.6 | 4.9 | 5 | PDIA3\|HSPA1A\|HSPA6\|HSPA8\|HSP90AA1 |
|  | hsa04611 | Platelet activation | 0.001259 | 5 | 4.4 | 6 | ACTB\|COL1A1\|COL1A2\|GNAI1\|PLA2G4A\|PTGS1 |
|  | hsa05144 | Malaria | 0.001259 | 8.3 | 5.1 | 4 | COMP\|SDC1\|THBS1\|THBS2 |
|  | hsa05164 | Influenza A | 0.001259 | 4.2 | 4.2 | 7 | ACTB\|CDK6\|DNAJB1\|KPNA2\|NXF1\|HNRNPUL1\|MAVS |
|  | hsa04926 | Relaxin signaling pathway | 0.001585 | 4.8 | 4.3 | 6 | COL1A1\|COL1A2\|COL4A1\|GNAI1\|MMP1\|MMP2 |
|  | hsa05134 | Legionellosis | 0.002512 | 7.3 | 4.7 | 4 | HSPA1A\|HSPA6\|HSPA8\|HSPD1 |
|  | hsa04371 | Apelin signaling pathway | 0.002512 | 4.5 | 4.1 | 6 | CCN2\|GNAI1\|SMAD3\|MYL3\|SERPINE1\|MAP1LC3B |
|  | hsa04213 | Longevity regulating pathway - multiple species | 0.003162 | 6.7 | 4.4 | 4 | CRYAB\|HSPA1A\|HSPA6\|HSPA8 |
|  | hsa04144 | Endocytosis | 0.003162 | 3.3 | 3.6 | 8 | BIN1\|EPS15\|HSPA1A\|HSPA6\|HSPA8\|SMAD3\|RAB7A\|RUFY2 |
|  | hsa04261 | Adrenergic signaling in cardiomyocytes | 0.003162 | 4.1 | 3.8 | 6 | GNAI1\|MYL3\|PPP2R2A\|TPM1\|TPM2\|TPM4 |
|  | hsa04217 | Necroptosis | 0.005012 | 3.9 | 3.6 | 6 | PARP1\|HSP90AA1\|PLA2G4A\|PPIA\|STAT6\|SQSTM1 |
|  | hsa05417 | Lipid and atherosclerosis | 0.005012 | 3.4 | 3.4 | 7 | HSPA1A\|HSPA6\|HSPA8\|HSP90AA1\|HSPD1\|MMP1\|MAP2K3 |
|  | hsa05225 | Hepatocellular carcinoma | 0.00631 | 3.7 | 3.5 | 6 | ACTB\|CDK6\|CSNK1A1\|HMOX1\|SMAD3\|MGST1 |
|  | hsa05133 | Pertussis | 0.00631 | 5.5 | 3.8 | 4 | SERPING1\|C1R\|C1S\|GNAI1 |
|  | hsa05022 | Pathways of neurodegeneration - multiple diseases | 0.00631 | 2.4 | 3 | 11 | APP\|ATP5F1C\|CSNK1A1\|MAP2K3\|PRNP\|RPS27A\|UBA7\|SQSTM1\|MFN2\|OPTN\|MAP1LC3B |
|  | hsa05219 | Bladder cancer | 0.007943 | 7.6 | 4.2 | 3 | MMP1\|MMP2\|THBS1 |
|  | hsa00860 | Porphyrin metabolism | 0.007943 | 7.2 | 4 | 3 | CPOX\|HMOX1\|UROD |
|  | hsa04621 | NOD-like receptor signaling pathway | 0.01 | 3.4 | 3.2 | 6 | HSP90AA1\|MFN2\|GABARAPL2\|MAVS\|MAP1LC3B\|ANTXR2 |
| Reactome | R-HSA-1474244 | Extracellular matrix organization | 1E-30 | 13 | 21 | 38 | APP\|BGN\|COL1A1\|COL1A2\|COL2A1\|COL4A1\|COL5A1\|COMP\|DCN\|FBN1\|EFEMP1\|FN1\|TNC\|ITGB5\|LAMA4\|LAMB1\|LAMB2\|LAMC1\|LAMC2\|LOX\|LOXL2\|LTBP2\|LUM\|MMP1\|MMP2\|MMP14\|NID1\|SERPINE1\|PCOLCE\|PLOD2\|PPIB\|SDC1\|SPARC\|THBS1\|TIMP1\|TIMP2\|PXDN\|JAM3 |
|  | R-HSA-3000178 | ECM proteoglycans | 1E-21 | 26 | 21 | 19 | APP\|BGN\|COL1A1\|COL1A2\|COL2A1\|COL4A1\|COL5A1\|COMP\|DCN\|FN1\|TNC\|ITGB5\|LAMA4\|LAMB1\|LAMB2\|LAMC1\|LUM\|SERPINE1\|SPARC |
|  | R-HSA-3000171 | Non-integrin membrane-ECM interactions | 1E-17 | 26 | 19 | 15 | COL1A1\|COL1A2\|COL2A1\|COL4A1\|COL5A1\|FN1\|TNC\|ITGB5\|LAMA4\|LAMB1\|LAMB2\|LAMC1\|LAMC2\|SDC1\|THBS1 |
|  | R-HSA-381426 | Regulation of Insulin-like Growth Factor (IGF) transport and uptake by Insulin-like Growth Factor Binding Proteins (IGFBPs) | 1E-17 | 16 | 16 | 19 | APP\|CALU\|CST3\|FBN1\|FN1\|TNC\|IGFBP3\|IGFBP5\|IGFBP7\|CCN1\|LAMB1\|LAMB2\|LAMC1\|MFGE8\|MMP1\|MMP2\|QSOX1\|TIMP1\|FSTL1 |
|  | R-HSA-2262752 | Cellular responses to stress | 1E-16 | 5 | 11 | 38 | ATP6V1C1\|CDK6\|CRYAB\|FKBP4\|NR3C1\|HMOX1\|HSBP1\|DNAJA1\|HSPA1A\|HSPA6\|HSPA8\|HSPA9\|HSP90AA1\|DNAJB1\|IGFBP7\|MAP2K3\|RBBP7\|RHEB\|RPL19\|RPL21\|RPL27\|RPL30\|RPL29\|RPLP2\|RPS12\|RPS27A\|SOD3\|DNAJC7\|SQSTM1\|BAG3\|DNAJB6\|HSPH1\|STIP1\|HSPA4L\|HIKESHI\|ATP6V1H\|MAP1LC3B\|NUP35 |
|  | R-HSA-3371556 | Cellular response to heat stress | 1E-15 | 19 | 16 | 16 | CRYAB\|FKBP4\|HSBP1\|HSPA1A\|HSPA6\|HSPA8\|HSPA9\|HSP90AA1\|DNAJB1\|DNAJC7\|BAG3\|DNAJB6\|HSPH1\|HSPA4L\|HIKESHI\|NUP35 |
|  | R-HSA-8957275 | Post-translational protein phosphorylation | 1E-15 | 16 | 16 | 17 | APP\|CALU\|CST3\|FBN1\|FN1\|TNC\|IGFBP3\|IGFBP5\|IGFBP7\|CCN1\|LAMB1\|LAMB2\|LAMC1\|MFGE8\|QSOX1\|TIMP1\|FSTL1 |
|  | R-HSA-8953897 | Cellular responses to stimuli | 1E-15 | 4.9 | 11 | 38 | ATP6V1C1\|CDK6\|CRYAB\|FKBP4\|NR3C1\|HMOX1\|HSBP1\|DNAJA1\|HSPA1A\|HSPA6\|HSPA8\|HSPA9\|HSP90AA1\|DNAJB1\|IGFBP7\|MAP2K3\|RBBP7\|RHEB\|RPL19\|RPL21\|RPL27\|RPL30\|RPL29\|RPLP2\|RPS12\|RPS27A\|SOD3\|DNAJC7\|SQSTM1\|BAG3\|DNAJB6\|HSPH1\|STIP1\|HSPA4L\|HIKESHI\|ATP6V1H\|MAP1LC3B\|NUP35 |
|  | R-HSA-1474228 | Degradation of the extracellular matrix | 1E-13 | 13 | 14 | 17 | COL1A1\|COL1A2\|COL2A1\|COL4A1\|COL5A1\|DCN\|FBN1\|FN1\|LAMB1\|LAMC1\|LAMC2\|MMP1\|MMP2\|MMP14\|NID1\|TIMP1\|TIMP2 |
|  | R-HSA-8874081 | MET activates PTK2 signaling | 1E-13 | 35 | 18 | 10 | COL1A1\|COL1A2\|COL2A1\|COL5A1\|FN1\|LAMA4\|LAMB1\|LAMB2\|LAMC1\|LAMC2 |
|  | R-HSA-109582 | Hemostasis | 1E-12 | 5 | 10 | 30 | ACTB\|APP\|SERPING1\|CALU\|COL1A1\|COL1A2\|ECM1\|F3\|FN1\|GNAI1\|LGALS3BP\|MMP1\|SERPINE1\|SERPINE2\|PLA2G4A\|PLAUR\|PPIA\|QSOX1\|PTPN1\|SDC1\|SLC3A2\|SPARC\|THBD\|THBS1\|TIMP1\|TMSB4X\|MANF\|MFN2\|PDPN\|JAM3 |
|  | R-HSA-6806834 | Signaling by MET | 1E-12 | 17 | 14 | 13 | COL1A1\|COL1A2\|COL2A1\|COL5A1\|EPS15\|FN1\|LAMA4\|LAMB1\|LAMB2\|LAMC1\|LAMC2\|PTPN1\|RPS27A |
|  | R-HSA-76002 | Platelet activation, signaling and aggregation | 1E-12 | 7.9 | 11 | 20 | APP\|SERPING1\|CALU\|COL1A1\|COL1A2\|ECM1\|FN1\|GNAI1\|LGALS3BP\|SERPINE1\|PLA2G4A\|PPIA\|QSOX1\|PTPN1\|SPARC\|THBS1\|TIMP1\|TMSB4X\|MANF\|PDPN |
|  | R-HSA-6798695 | Neutrophil degranulation | 1E-12 | 5.6 | 10 | 26 | CHI3L1\|CST3\|HSPA1A\|HSPA6\|HSPA8\|HSP90AA1\|LGALS3\|MGST1\|PNP\|PLAUR\|PPIA\|QSOX1\|PTX3\|SLC2A3\|SRP14\|TIMP2\|TNFAIP6\|RAB7A\|HUWE1\|ATP6AP2\|GLIPR1\|RAB18\|DNAJC13\|GOLGA7\|YPEL5\|RAP2C |
|  | R-HSA-216083 | Integrin cell surface interactions | 1E-12 | 16 | 14 | 13 | COL1A1\|COL1A2\|COL2A1\|COL4A1\|COL5A1\|COMP\|FBN1\|FN1\|TNC\|ITGB5\|LUM\|THBS1\|JAM3 |
|  | R-HSA-3371453 | Regulation of HSF1-mediated heat shock response | 1E-12 | 18 | 14 | 12 | HSPA1A\|HSPA6\|HSPA8\|HSPA9\|DNAJB1\|DNAJC7\|BAG3\|DNAJB6\|HSPH1\|HSPA4L\|HIKESHI\|NUP35 |
|  | R-HSA-8875878 | MET promotes cell motility | 1E-11 | 25 | 15 | 10 | COL1A1\|COL1A2\|COL2A1\|COL5A1\|FN1\|LAMA4\|LAMB1\|LAMB2\|LAMC1\|LAMC2 |
|  | R-HSA-9006934 | Signaling by Receptor Tyrosine Kinases | 1E-11 | 5.2 | 9.5 | 26 | ACTB\|ATP6V1C1\|COL1A1\|COL1A2\|COL2A1\|COL4A1\|COL5A1\|EPS15\|F3\|FN1\|HSPB1\|HSP90AA1\|LAMA4\|LAMB1\|LAMB2\|LAMC1\|LAMC2\|PTPN1\|RPS27A\|SPARC\|STAT6\|THBS1\|THBS2\|NRP1\|ABI1\|ATP6V1H |
|  | R-HSA-114608 | Platelet degranulation | 1E-10 | 11 | 12 | 14 | APP\|SERPING1\|CALU\|ECM1\|FN1\|LGALS3BP\|SERPINE1\|PPIA\|QSOX1\|SPARC\|THBS1\|TIMP1\|TMSB4X\|MANF |
|  | R-HSA-76005 | Response to elevated platelet cytosolic Ca2+ | 1E-10 | 11 | 11 | 14 | APP\|SERPING1\|CALU\|ECM1\|FN1\|LGALS3BP\|SERPINE1\|PPIA\|QSOX1\|SPARC\|THBS1\|TIMP1\|TMSB4X\|MANF |
|  | R-HSA-1474290 | Collagen formation | 1E-10 | 14 | 12 | 12 | COL1A1\|COL1A2\|COL2A1\|COL4A1\|COL5A1\|LAMC2\|LOX\|LOXL2\|PCOLCE\|PLOD2\|PPIB\|PXDN |
|  | R-HSA-3000170 | Syndecan interactions | 1.26E-10 | 31 | 15 | 8 | COL1A1\|COL1A2\|COL5A1\|FN1\|TNC\|ITGB5\|SDC1\|THBS1 |
|  | R-HSA-2243919 | Crosslinking of collagen fibrils | 2E-10 | 40 | 16 | 7 | COL1A1\|COL1A2\|COL4A1\|LOX\|LOXL2\|PCOLCE\|PXDN |
|  | R-HSA-2022090 | Assembly of collagen fibrils and other multimeric structures | 3.16E-10 | 17 | 12 | 10 | COL1A1\|COL1A2\|COL2A1\|COL4A1\|COL5A1\|LAMC2\|LOX\|LOXL2\|PCOLCE\|PXDN |
|  | R-HSA-422475 | Axon guidance | 1E-09 | 4.5 | 8.2 | 24 | ACTB\|COL2A1\|COL4A1\|COL5A1\|DPYSL2\|HSPA8\|HSP90AA1\|LAMB1\|LAMC1\|MMP2\|PRNP\|RPL19\|RPL21\|RPL27\|RPL30\|RPL29\|RPLP2\|RPS12\|RPS27A\|SLIT3\|NUMB\|NRP1\|EIF4A3\|PLXNA3 |
|  | R-HSA-3371568 | Attenuation phase | 2E-09 | 44 | 16 | 6 | FKBP4\|HSBP1\|HSPA1A\|HSPA8\|HSP90AA1\|DNAJB1 |
|  | R-HSA-3371571 | HSF1-dependent transactivation | 2E-09 | 30 | 14 | 7 | CRYAB\|FKBP4\|HSBP1\|HSPA1A\|HSPA8\|HSP90AA1\|DNAJB1 |
|  | R-HSA-5653656 | Vesicle-mediated transport | 2.51E-09 | 4 | 7.8 | 26 | ACTB\|BIN1\|APP\|COL1A1\|COL1A2\|COL4A1\|EPS15\|HSPA8\|HSP90AA1\|PLA2G4A\|RPS27A\|SPARC\|RAB7A\|NAPG\|COPS2\|OPTN\|HSPH1\|TMED10\|PACSIN2\|GABARAPL2\|RAB18\|RAB21\|TRAPPC3\|GOLIM4\|MAP1LC3B\|SH3D19 |
|  | R-HSA-9675108 | Nervous system development | 2.51E-09 | 4.3 | 7.9 | 24 | ACTB\|COL2A1\|COL4A1\|COL5A1\|DPYSL2\|HSPA8\|HSP90AA1\|LAMB1\|LAMC1\|MMP2\|PRNP\|RPL19\|RPL21\|RPL27\|RPL30\|RPL29\|RPLP2\|RPS12\|RPS27A\|SLIT3\|NUMB\|NRP1\|EIF4A3\|PLXNA3 |
|  | R-HSA-168255 | Influenza Infection | 3.98E-09 | 8.6 | 9.4 | 13 | PARP1\|HSPA1A\|HSP90AA1\|KPNA2\|RPL19\|RPL21\|RPL27\|RPL30\|RPL29\|RPLP2\|RPS12\|RPS27A\|NUP35 |
|  | R-HSA-3000157 | Laminin interactions | 1.26E-08 | 24 | 13 | 7 | COL4A1\|LAMA4\|LAMB1\|LAMB2\|LAMC1\|LAMC2\|NID1 |
|  | R-HSA-9006936 | Signaling by TGFB family members | 3.16E-08 | 9.3 | 9.1 | 11 | PARP1\|COL1A2\|FBN1\|FKBP1A\|INHBA\|ITGB5\|LTBP2\|SMAD3\|SERPINE1\|RPS27A\|FSTL1 |
|  | R-HSA-9679506 | SARS-CoV Infections | 3.16E-08 | 5 | 7.6 | 18 | CSNK1A1\|FKBP1A\|FKBP4\|NR3C1\|HSP90AA1\|KPNA2\|RBBP7\|RPS12\|RPS27A\|SDC1\|CNBP\|NRP1\|G3BP2\|SAP18\|GOLGA7\|MAVS\|MAP1LC3B\|NUP35 |
|  | R-HSA-1280215 | Cytokine Signaling in Immune system | 5.01E-08 | 3.6 | 6.9 | 25 | APP\|COL1A2\|FN1\|HMOX1\|HSPA8\|HSPA9\|HSP90AA1\|IL13RA2\|KPNA2\|SMAD3\|MMP1\|MMP2\|PPIA\|MAP2K3\|PTPN1\|RPS27A\|SDC1\|STAT6\|TIMP1\|UBA7\|SQSTM1\|AIP\|EIF4A3\|TRIM22\|NUP35 |
|  | R-HSA-3371497 | HSP90 chaperone cycle for steroid hormone receptors (SHR) in the presence of ligand | 5.01E-08 | 15 | 10 | 8 | FKBP4\|NR3C1\|DNAJA1\|HSPA1A\|HSPA8\|HSP90AA1\|DNAJB1\|STIP1 |
|  | R-HSA-168273 | Influenza Viral RNA Transcription and Replication | 7.94E-08 | 8.4 | 8.6 | 11 | PARP1\|HSP90AA1\|RPL19\|RPL21\|RPL27\|RPL30\|RPL29\|RPLP2\|RPS12\|RPS27A\|NUP35 |
|  | R-HSA-6785807 | Interleukin-4 and Interleukin-13 signaling | 1E-07 | 9.6 | 8.8 | 10 | COL1A2\|FN1\|HMOX1\|HSPA8\|HSP90AA1\|IL13RA2\|MMP1\|MMP2\|STAT6\|TIMP1 |
|  | R-HSA-1799339 | SRP-dependent cotranslational protein targeting to membrane | 1.58E-07 | 9.3 | 8.6 | 10 | RPL19\|RPL21\|RPL27\|RPL30\|RPL29\|RPLP2\|RPS12\|RPS27A\|SRP14\|SPCS3 |
|  | R-HSA-927802 | Nonsense-Mediated Decay (NMD) | 2E-07 | 9 | 8.5 | 10 | PPP2R2A\|RPL19\|RPL21\|RPL27\|RPL30\|RPL29\|RPLP2\|RPS12\|RPS27A\|EIF4A3 |
|  | R-HSA-975957 | Nonsense Mediated Decay (NMD) enhanced by the Exon Junction Complex (EJC) | 2E-07 | 9 | 8.5 | 10 | PPP2R2A\|RPL19\|RPL21\|RPL27\|RPL30\|RPL29\|RPLP2\|RPS12\|RPS27A\|EIF4A3 |
|  | R-HSA-1442490 | Collagen degradation | 2E-07 | 13 | 9.5 | 8 | COL1A1\|COL1A2\|COL2A1\|COL4A1\|COL5A1\|MMP1\|MMP2\|MMP14 |
|  | R-HSA-1566948 | Elastic fibre formation | 2E-07 | 16 | 10 | 7 | FBN1\|EFEMP1\|FN1\|ITGB5\|LOX\|LOXL2\|LTBP2 |
|  | R-HSA-449147 | Signaling by Interleukins | 2E-07 | 4.2 | 6.8 | 19 | APP\|COL1A2\|FN1\|HMOX1\|HSPA8\|HSPA9\|HSP90AA1\|IL13RA2\|SMAD3\|MMP1\|MMP2\|PPIA\|MAP2K3\|RPS27A\|SDC1\|STAT6\|TIMP1\|SQSTM1\|AIP |
|  | R-HSA-1650814 | Collagen biosynthesis and modifying enzymes | 2.51E-07 | 12 | 9.2 | 8 | COL1A1\|COL1A2\|COL2A1\|COL4A1\|COL5A1\|PCOLCE\|PLOD2\|PPIB |
|  | R-HSA-170834 | Signaling by TGF-beta Receptor Complex | 3.16E-07 | 10 | 8.6 | 9 | PARP1\|COL1A2\|FBN1\|FKBP1A\|ITGB5\|LTBP2\|SMAD3\|SERPINE1\|RPS27A |
|  | R-HSA-9711097 | Cellular response to starvation | 3.98E-07 | 7.3 | 7.8 | 11 | ATP6V1C1\|RHEB\|RPL19\|RPL21\|RPL27\|RPL30\|RPL29\|RPLP2\|RPS12\|RPS27A\|ATP6V1H |
|  | R-HSA-156827 | L13a-mediated translational silencing of Ceruloplasmin expression | 1.26E-06 | 8.4 | 7.7 | 9 | RPL19\|RPL21\|RPL27\|RPL30\|RPL29\|RPLP2\|RPS12\|RPS27A\|EIF4H |
|  | R-HSA-72706 | GTP hydrolysis and joining of the 60S ribosomal subunit | 1.58E-06 | 8.3 | 7.7 | 9 | RPL19\|RPL21\|RPL27\|RPL30\|RPL29\|RPLP2\|RPS12\|RPS27A\|EIF4H |
|  | R-HSA-192823 | Viral mRNA Translation | 2.51E-06 | 9.3 | 7.8 | 8 | RPL19\|RPL21\|RPL27\|RPL30\|RPL29\|RPLP2\|RPS12\|RPS27A |
|  | R-HSA-156902 | Peptide chain elongation | 2.51E-06 | 9.3 | 7.8 | 8 | RPL19\|RPL21\|RPL27\|RPL30\|RPL29\|RPLP2\|RPS12\|RPS27A |
|  | R-HSA-72613 | Eukaryotic Translation Initiation | 2.51E-06 | 7.8 | 7.4 | 9 | RPL19\|RPL21\|RPL27\|RPL30\|RPL29\|RPLP2\|RPS12\|RPS27A\|EIF4H |
|  | R-HSA-72737 | Cap-dependent Translation Initiation | 2.51E-06 | 7.8 | 7.4 | 9 | RPL19\|RPL21\|RPL27\|RPL30\|RPL29\|RPLP2\|RPS12\|RPS27A\|EIF4H |
|  | R-HSA-2173782 | Binding and Uptake of Ligands by Scavenger Receptors | 3.16E-06 | 15 | 8.8 | 6 | COL1A1\|COL1A2\|COL4A1\|HSP90AA1\|SPARC\|HSPH1 |
|  | R-HSA-8953854 | Metabolism of RNA | 3.16E-06 | 3.2 | 5.8 | 21 | HSPA1A\|HSPA8\|HSPB1\|PPP2R2A\|RPL19\|RPL21\|RPL27\|RPL30\|RPL29\|RPLP2\|RPS12\|RPS27A\|SART1\|EIF4A3\|NXF1\|HNRNPUL1\|DDX42\|RIOK2\|SKIC8\|TNKS1BP1\|NUP35 |
|  | R-HSA-72764 | Eukaryotic Translation Termination | 3.16E-06 | 8.9 | 7.5 | 8 | RPL19\|RPL21\|RPL27\|RPL30\|RPL29\|RPLP2\|RPS12\|RPS27A |
|  | R-HSA-2408557 | Selenocysteine synthesis | 3.16E-06 | 8.9 | 7.5 | 8 | RPL19\|RPL21\|RPL27\|RPL30\|RPL29\|RPLP2\|RPS12\|RPS27A |
|  | R-HSA-156842 | Eukaryotic Translation Elongation | 3.16E-06 | 8.9 | 7.5 | 8 | RPL19\|RPL21\|RPL27\|RPL30\|RPL29\|RPLP2\|RPS12\|RPS27A |
|  | R-HSA-975956 | Nonsense Mediated Decay (NMD) independent of the Exon Junction Complex (EJC) | 3.98E-06 | 8.7 | 7.4 | 8 | RPL19\|RPL21\|RPL27\|RPL30\|RPL29\|RPLP2\|RPS12\|RPS27A |
|  | R-HSA-199991 | Membrane Trafficking | 3.98E-06 | 3.3 | 5.7 | 20 | ACTB\|BIN1\|APP\|EPS15\|HSPA8\|PLA2G4A\|RPS27A\|RAB7A\|NAPG\|COPS2\|OPTN\|TMED10\|PACSIN2\|GABARAPL2\|RAB18\|RAB21\|TRAPPC3\|GOLIM4\|MAP1LC3B\|SH3D19 |
|  | R-HSA-9694516 | SARS-CoV-2 Infection | 5.01E-06 | 4.7 | 6.2 | 13 | CSNK1A1\|HSP90AA1\|KPNA2\|RPS12\|RPS27A\|SDC1\|CNBP\|NRP1\|G3BP2\|GOLGA7\|MAVS\|MAP1LC3B\|NUP35 |
